# First-line durvalumab and tremelimumab with chemotherapy in RAS-mutated metastatic colorectal cancer: a phase 1b/2 trial

**DOI:** 10.1038/s41591-023-02497-z

**Published:** 2023-08-10

**Authors:** Marion Thibaudin, Jean-David Fumet, Benoist Chibaudel, Jaafar Bennouna, Christophe Borg, Jerome Martin-Babau, Romain Cohen, Marianne Fonck, Julien Taieb, Emeric Limagne, Julie Blanc, Elise Ballot, Léa Hampe, Marjorie Bon, Susy Daumoine, Morgane Peroz, Hugo Mananet, Valentin Derangère, Romain Boidot, Henri-Alexandre Michaud, Caroline Laheurte, Olivier Adotevi, Aurélie Bertaut, Caroline Truntzer, François Ghiringhelli

**Affiliations:** 1 Université Bourgogne Franche-Comté, Dijon, France; 2 Cancer Biology Transfer Platform, Department of Biology and Pathology of Tumors, Georges-François Leclerc Anticancer Center, UNICANCER, Dijon, France; 3 Centre de Recherche INSERM LNC-UMR1231, Dijon, France; 4 Department of Medical Oncology, Centre Georges-François Leclerc, Dijon, France; 5 Genetic and Immunology Medical Institute, Dijon, France; 6 Department of Medical Oncology, Hôpital Franco-Britannique – Fondation Cognacq-Jay, Levallois-Perret, France; 7 Department of Gastroentérology, CHU, Nantes, France; 8 Department of Medical Oncology, CHU, Besançon, France; 9 Department of Medical Oncology, Clinique CARIO, Plerin, France; 10 Department of Medical Oncology, Saint Antoine, Hospital, Paris, France; 11 Department of Medical Oncology, Institut Bergonie, Bordeaux, France; 12 Department of Gastroenterology, Pompidou Hospital, Paris, France; 13 Department of Statistics, Centre Georges-François Leclerc, Dijon, France; 14 Unit of Molecular Biology, Department of Biology and Pathology of Tumors, Georges-François Leclerc Anticancer Center, UNICANCER, Dijon, France; 15 Plateforme de Cytométrie et d’Imagerie de Masse, IRCM, University of Montpellier, ICM, Inserm Montpellier, Montpellier, France; 16 INSERM EFS UMR1098 RIGHT Interactions Hôte-Greffon-Tumeur - Ingénierie Cellulaire et Génique, Université Bourgogne Franche-Comté, Besançon, France

**Keywords:** Cancer immunotherapy, Tumour immunology

## Abstract

Although patients with microsatellite instable metastatic colorectal cancer (CRC) benefit from immune checkpoint blockade, chemotherapy with targeted therapies remains the only therapeutic option for microsatellite stable (MSS) tumors. The single-arm, phase 1b/2 MEDITREME trial evaluated the safety and efficacy of durvalumab plus tremelimumab combined with mFOLFOX6 chemotherapy in first line, in 57 patients with RAS-mutant unresectable metastatic CRC. Safety was the primary objective of phase Ib; no safety issue was observed. The phase 2 primary objective of efficacy in terms of 3-month progression-free survival (PFS) in patients with MSS tumors was met, with 3-month PFS of 90.7% (95% confidence interval (CI): 79.2–96%). For secondary objectives, response rate was 64.5%; median PFS was 8.2 months (95% CI: 5.9–8.6); and overall survival was not reached in patients with MSS tumors. We observed higher tumor mutational burden and lower genomic instability in responders. Integrated transcriptomic analysis underlined that high immune signature and low epithelial–mesenchymal transition were associated with better outcome. Immunomonitoring showed induction of neoantigen and NY-ESO1 and TERT blood tumor-specific T cell response associated with better PFS. The combination of durvalumab–tremelimumab with mFOLFOX6 was tolerable with promising clinical activity in MSS mCRC. Clinicaltrials.gov identifier: NCT03202758.

## Abstract (web summary)

Treatment with anti-PDL1 and anti-CTLA4 in combination with mFOLFOX6 chemotherapy for patients with treatment-naive microsatellite stable metastatic colorectal cancer is safe, shows encouraging progression-free survival and induces a tumor-specific immune response.

## Main

Treatment of metastatic colorectal cancer (CRC) relies mainly on chemotherapy, generally for palliative purposes when metastases cannot be removed. Median overall survival (OS) of CRC has been rising with improvements in chemotherapeutic and targeted therapies^1–6^. CRC is a heterogeneous disease classified by its genetic characteristics, which guide prognosis and therapy^7–9^. One particular genetic subset of CRC is tumors with microsatellite instability (MSI), resulting in high tumor mutation burden (TMB) and large immune infiltrates^10^. For such tumors, immunotherapy using a monoclonal antibody targeting PD-1/PD-L1 has demonstrated efficacy^11^. For other CRC types, termed microsatellite stable (MSS), immunotherapy is ineffective as monotherapy^12^.

Many studies have underlined that the immune system recognizes CRC, and high CD8 T cell infiltrates are associated with better prognosis in localized or metastatic CRC^13,14^. Preclinical data suggest that combining a PD-1/PD-L1 inhibitor with an immunogenic cell death inducer, such as oxaliplatin, could enhance immunotherapy efficacy^15–17^. 5-fluorouracil (5-FU) could eliminate myeloid-derived suppressor cells and limit tumor-induced immunosuppression^18,19^. Thus, combining 5-FU and oxaliplatin could improve anti-tumor immune response. In mouse CRC models, a synergistic effect was observed with an anti-PD-L1 + FOLFOX combination^20^. Based on these data, we designed the phase 1b/2 MEDITREME trial (NCT03202758). In this Article, patients were treated with 3 months of modified mFOLFOX6 regimen (six cycles) combined with durvalumab and tremelimumab as induction therapy, followed by maintenance therapy with durvalumab until progression. The aim was to investigate feasibility and efficacy and to explore the genomic and immunologic features of response in MSS mCRC. To obtain homogenous response rates and progression-free survival (PFS), we focused on patients with RAS-mutated tumours.

## Results

### Patient characteristics

**Extended Data Fig. 1:**
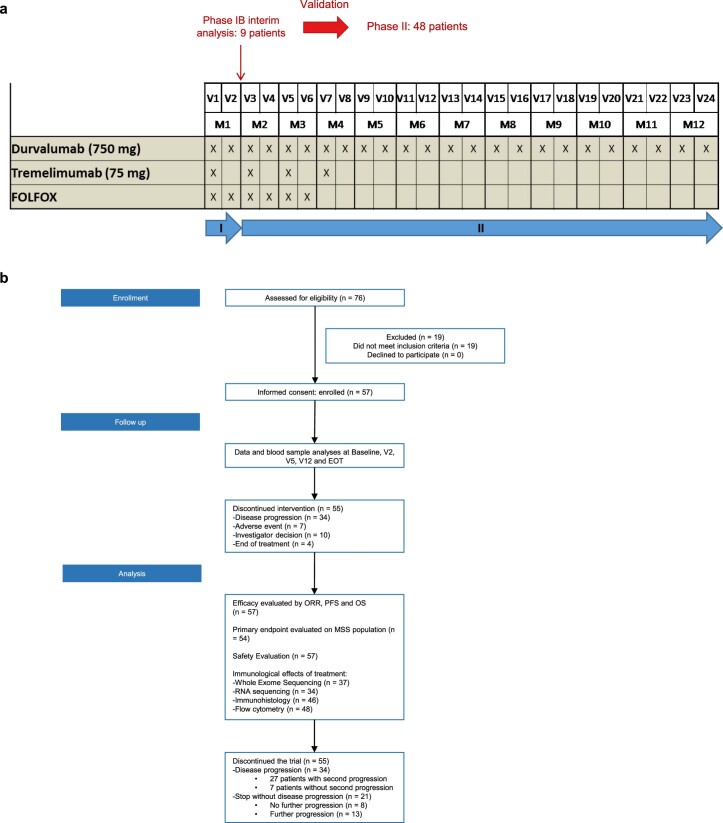
Diagram and flowchart of study cohort. **a**, The diagram shows the study design divided into 2 phases: In phase 1b, 9 patients were included and interim analysis was performed to validate the study design. Then, phase 2 could start, and 48 further patients were included. The diagram shows the treatment regimen tested with 6 consecutive courses of FOLFOX, 4 courses of tremelimumab every other week and durvalumab every week until progression. **b**, A total of 76 patients were assessed for eligibility and 57 were recruited into the study. Data and blood samples were processed from 57 patients and analyzed at baseline, V2, V5, V12 and end of treatment. Treatment efficacy was evaluated by objective response rate (ORR), progression-free survival (PFS) and overall survival (OS) in all 57 patients. The primary endpoint was evaluated in 54 patients comprising the MSS population, and safety evaluation was performed in all 57 patients. Immunological effects of treatment were studied with whole exome sequencing in 37 patients, RNA sequencing in 36 patients, immunohistology in 46 patients and flow cytometry in 48 patients.

Overall, 57 patients with unresectable metastatic RAS-mutated CRC were included from eight French hospitals between 30 August 2017 and 20 December 2019 (Extended Data Fig. 1a,b). Patients received six cycles (3 months) of mFOLFOX6 (oxaliplatin (85 mg m^−2^) and folinic acid (200 mg m^−2^)) intravenously on day 1, followed by 5-FU (400 mg m^−2^) intravenously and then 5-FU (2,400 mg m^−2^) intravenously, preceded by durvalumab (750 mg every 2 weeks) and tremelimumab (75 mg every 4 weeks). Patients with stable or responding tumors after concurrent therapy continued on maintenance durvalumab (750 mg every 2 weeks) for a maximum of 1 year since first study treatment. The first part was a phase Ib study with nine included patients. A protocol-defined safety review was performed. Absent any safety event warning, 48 additional patients were included in phase 2. Median age was 63.6 years (range, 28–80); 33 (58%) patients were female; 17 (30%) patients had left-sided CRC; 13 (23%) patients had rectal cancers; 45 (79%) patients had liver metastases; 23 (40%) patients had lung metastases; and 17 (30%) patients had peritoneal metastases. Ten (17.5%) patients received mFOLFOX6 as adjuvant therapy. Ten (17.5%) patients had metachronous diseases (Extended Data Table 1).

### Feasibility and safety

Adverse events (AEs) occurred in 56 (98%) patients (Extended Data Table 2). Treatment-related grade 3/4 AEs occurred in 38 (67%) patients, leading to treatment discontinuation in seven (12%) patients (two diabetes, two hypophysitis, two infusion reactions and one encephalitis). No grade 5 AEs occurred. Toxicities were mainly related to chemotherapy, with chemotherapy-related grade 3 and grade 4 toxicities observed in 32 (56%) patients, those related to immunotherapy in eight (14%) patients. For chemotherapy-related toxicity, the most common events were diarrhea in 34 (60%) patients, neutropenia in 28 (49%) patients and thrombocytopenia in 20 (35%) patients. For immunotherapy-related toxicity, the most frequent events were skin reaction in 21 (37%) patients, endocrinopathy in 15 (26%) patients and colitis and hepatitis in three (5%) patients. Notably, 90% of grade 3/4 AEs occurred during chemo-immunotherapy.

### Efficacy analyses

**Fig. 1:**
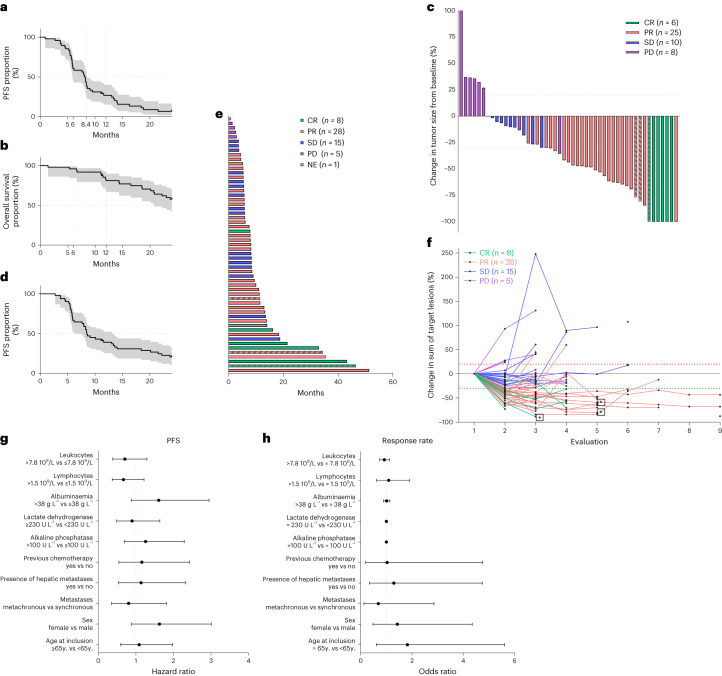
Clinical characterization of patients. **a,b**, Kaplan–Meier curves of PFS (**a**) and OS (**b**) in patients with MSS tumors with the median DOR (*n* = 48). **c**, Waterfall plot for target lesion tumor size for the whole cohort (*n* = 57), colored according to RECIST based on maximal percentage of tumor reduction from baseline. Patients with MSI tumors (*n* = 3) are identified with dashed bars. **d**, Kaplan–Meier curves of DOR in patients with MSS tumors (*n* = 48) with the median DOR. **e**, Swimmer plot of PFS times for the whole cohort (*n* = 57), colored according to RECIST. The length of the bars represent the time from randomization to disease progression. RECIST was not evaluable for one patient. Patients with MSI tumors (*n* = 3) are identified with dashed bars. NE, non-evaluable. **f**, Spider plot showing percent change from baseline in sum of diameter according to the evaluation times for the whole population (*n* = 57). Each line corresponds to one patient and is colored according to RECIST. Patients with MSI tumors are identified with stars. **g**, Forest plot representation of overall HR estimates with 95% CIs for the association of clinical variables with PFS in the MSS cohort (*n* = 48). The circle symbols represent the point estimates, and the whiskers represent the 95% CI. The vertical, dashed line is marking no change (a ratio of 1) compared to the reference level. **h**, Forest plot representation of odds ratio estimates with 95% CIs for the association of clinical variables with objective response rate in the MSS cohort (*n* = 48). The circle symbols represent the point estimates, and the whiskers represent the 95% CI. The vertical, dashed line is marking no change (a ratio of 1) compared to the reference level. Two-sided *P* value with significance level set at 0.05. For each Kaplan–Meier curve, s.d. interval is marked in gray. Source data

Among the 57 patients, MSS status was known for 51 patients; three had MSI status; and 48 had MSS status. Only the 48 patients with MSS tumors were included in the eligible population for efficacy analyses, per protocol. Median follow-up was 36 months (2.5–33.9). The primary objective of phase 2 was met, with 3-month PFS of 90.7% (95% confidence interval (CI): 79.2–96%). Six-month, 12-month and 24-month PFS was, respectively, 60.4% (95% CI: 45.2–72.6%), 26.9% (95% CI: 15.3–39.9%) and 6.7% (95% CI: 1.8–16.5 %). Regarding secondary objectives, median PFS was 8.2 months (95% CI: 5.9–8.6) (Fig. 1a). OS at 6 months, 12 months and 24 months, was respectively, 95.8% (95% CI: 84.3–98.9%), 81.1% (95% CI: 66.8–89.7%) and 57.6% (95% CI: 42.3–70.2%). Median OS was not reached (Fig. 1b). Moreover, 31 (64.5%) patients achieved Response Evaluation Criteria in Solid Tumors (RECIST) objective response; 25 (52%) patients achieved partial response; and six (12.5%) patients achieved complete response. The disease control rate (complete response + partial response + stable disease) was 93.7%.

In the whole population, the estimated percentage of patients alive at 6 months, 12 months and 24 months was 96.5% (95% CI: 86.7–99.1%), 80.6% (95% CI: 67.6–88.8%) and 59.1% (95% CI: 45.1–70.6-%), respectively. The estimated percentages of patients with, respectively, 6 months, 12 months and 24 months of PFS were 63.2% (95% CI: 49.3–74.2%), 38.5% (95% CI: 26–50.9%) and 19.9% (10.6–31.3%). Best response for all patients is shown in a waterfall plot (Fig. 1c). Median treatment duration was 5.4 months (0.9–12 months). Kaplan–Meier, swimmer and spider plots show the duration of response (DOR) (Fig. 1d–f). Median DOR was 8.5 months (95% CI: 6.2–13.4), and 28% of patients were still under durvalumab at 12 months and 61% at 6 months while receiving only 3 months of chemotherapy. Five patients had FOLFOX durvalumab and tremelimumab reintroduction at progression decided by the investigator, and three patients were still responders at database closure. At the time of analysis, two patients were still under treatment; 34 patients discontinued the study due to disease progression; 13 patients stopped for other reasons and, thus, relapsed; and eight patients were in complete remission without relapse.

In a non-predefined subgroup, we analyzed clinical prognostic variables. No significant difference in terms of response rate or PFS was observed for classical prognostic variables (Fig. 1g,h).

### Exploratory analysis of genomic correlates

**Fig. 2:**
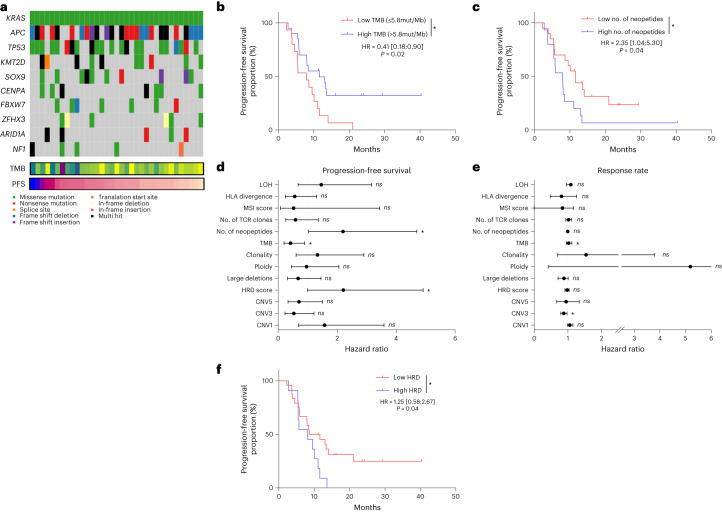
Genomic characterization of patients. **a**, Oncoplot representing genomic landscape of genes most frequently observed in the cohort. TMB and PFS times are also indicated for each patient at the bottom. **b**, Kaplan–Meier curves for PFS with patients stratified according to TMB, with a cutoff of 5.8 mutations per Mb (*n* = 35). Two-sided *P* value with significance level set at 0.05. **c**, Kaplan–Meier curves for PFS with patients stratified according to number of neopeptides with a cutoff on the median (*n* = 35). Two-sided *P* value with significance level set at 0.05. **d**, Forest plot representation of overall HR estimates with 95% CIs for the association of whole-exome-derived variables with PFS. **e**, Forest plot representation of odds ratio estimates with 95% CIs for the association of whole-exome-derived variables with objective response rate. The circle symbols represent the point estimates, and the whiskers represent the 95% CI (*n* = 35 patients (**d**,**e**)). The vertical, dashed line is marking no change (a ratio of 1) compared to the reference level. **f**, Kaplan–Meier curves for PFS with patients stratified according to the HRD score (*n* = 35). Two-sided *P* value with significance level set at 0.05. Survival distributions were compared using the log-rank test (**b**,**c**,**f**). Univariate Cox proportional hazard models were performed to estimate the HR and 95% CI (**d**,**e**). *P < 0.05, assessed using the two-sided Wald test (**d**,**e**). mut/Mb, mutations per megabase. Source data

**Extended Data Fig. 2:**
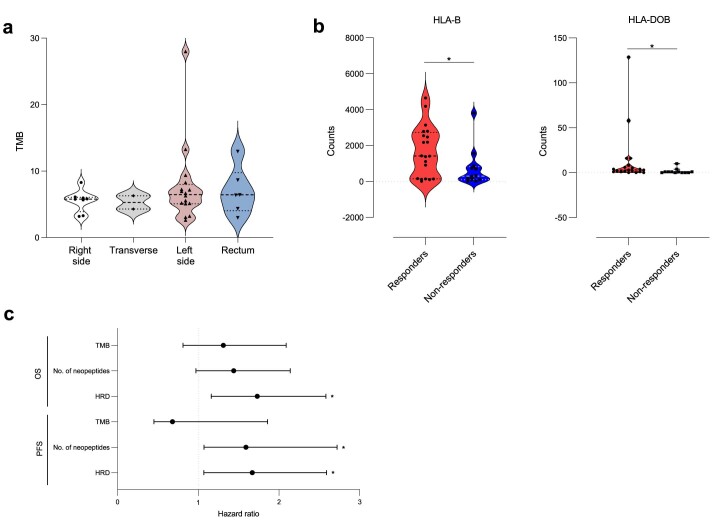
Exploratory analysis of genomic correlates. **a**, Violin plots showing the median, variability, and probability density of TMB for each type of colorectal cancer localization. **b**, Violin plots showing the median, variability, and probability density of HLA-B (left) and HLA-DOB (right) gene counts for responder and non-responder patients. *p < 0.05, data were compared using an unpaired two-sided Mann-Whitney Wilcoxon test. **c**, Forest plots of overall hazard ratio (HR) estimates with 95% confidence intervals for the association of clinical variables with overall and progression-free survival for tumor mutational burden (TMB), number of neopeptides and homologous repair deficiency (HRD) estimated on the TCGA cohort (n = 337 patients). The circles represent the point estimates and the whiskers represent the 95% CI. The vertical, dashed line marks no change (a ratio of one), compared to the reference level. *p < 0.05, assessed using the two-sided Wald test. Source data

Somatic panel to confirm NRAS, KRAS and BRAF mutation and MSI status was performed for all patients. Exome sequencing was performed in 37 patients. The most frequent mutations were APC, KRAS and TP53 (Fig. 2a). No genetic alteration occurring in more than 10% of patients was associated with PFS (Fig. 2a). Median TMB was 6.1 mutations per megabase (Mb) for all patients and did not differ according to tumor sidedness (Extended Data Fig. 2a). Three patients had TMB of more than 10 mutations per Mb; TMB > 5.8 was associated with longer PFS (hazard ratio (HR) = 0.41, 95% CI: 0.18–0.90, *P* = 0.02) (Fig. 2b). When we evaluated non-synonymous sequence alterations associated with putatively immunogenic class I neoantigens (using pVACtools^21^), we found that a low number of neoantigens (<14) was associated with better PFS (HR = 2.35, 95% CI: 1.04–5.30, *P* = 0.04) (Fig. 2c). Maximal germline physiochemical sequence divergence at the human leukocyte antigen (HLA) class I locus was not associated with PFS or objective response rate (Fig. 2d,e). Although HLA divergence was not related to outcome, higher expression of HLA-B and HLA-DOB mRNA was associated with better response rate, suggesting that higher capacity for T cell antigen presentation improves treatment efficacy (Extended Data Fig. 2b). Tumor genomic alterations were characterized by clonality, ploidy, loss of heterozygosity and large chromosomal deletion. Genomic structure alterations were estimated using homologous recombination deficiency (HRD) score, a measure of genomic instability^22^; low HRD score (<29) was associated with better PFS (Fig. 2f).

We analyzed data from tumor whole-exome sequencing from an independent cohort of 341 CRC tumors from The Cancer Genome Atlas (TCGA), which pre-dated the era of immune checkpoint blockade. In the TCGA cohort, TMB was not associated with PFS or OS. HRD score was associated with short PFS and OS, and a high number of neopeptides was associated with short PFS (Extended Data Fig. 2c). Together, these results support that, although TMB seems to be a predictive marker, other genomic markers are more prognostic than predictive.

### Exploratory analysis of transcriptome correlates

**Fig. 3:**
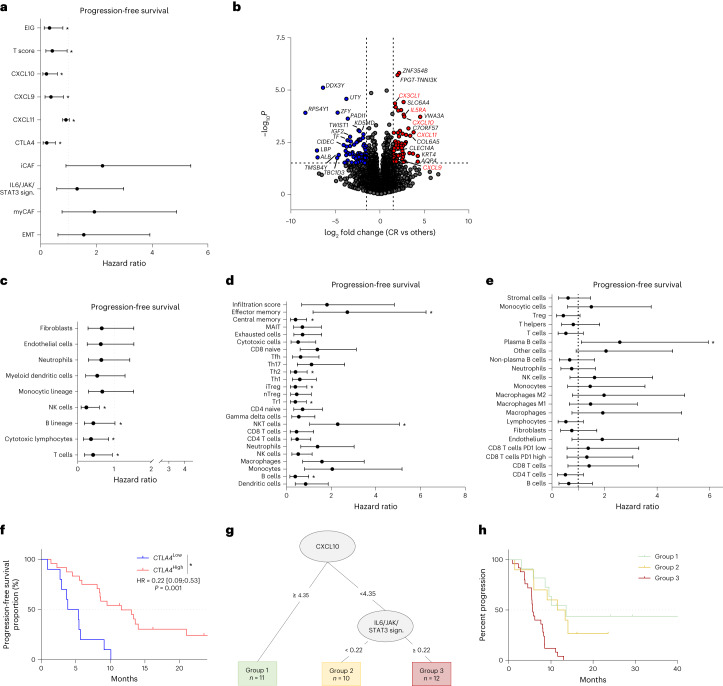
Transcriptomic characterization of patients. **a**, Forest plot of overall HR estimates with 95% CIs for the association of selected RNA-seq-derived signatures related to EMT and T cell infiltration with PFS (*n* = 32). **b**, Volcano plot describing differential analysis performed on RNA-seq data between complete responders and other patients. The log_2_ FC indicates the mean expression level for each gene. Each dot represents one gene. **c**, Forest plots of overall HR estimates with 95% CIs for the association of immune cell populations derived from MCP-counter with PFS (*n* = 32). **d**, Forest plots of overall HR estimates with 95% CIs for the association of immune cell populations derived from ImmuCellAI with PFS (*n* = 32). **e**, Forest plots of overall HR estimates with 95% CIs for the association of immune cell populations derived from Kassandra with PFS (*n* = 32). The circle symbols represent the point estimates, and the whiskers represent the 95% CI (**a**,**c**–**e**). The vertical, dashed line is marking no change (a ratio of 1) compared to the reference level. *P < 0.05, log-rank test (**a**,**c**–**e**). **f**, Kaplan–Meier curves for PFS with patients stratified according to *CTLA4* gene expression level with a cutoff at the median. Two-sided *P* value with significance level set at 0.05. **g**, Decision tree for PFS estimated with stromal and immune-related parameters. **h**, Kaplan–Meier curves with patients stratified according to groups created by the decision tree for PFS. Two-sided *P* value with significance level set at 0.05. Survival distributions were compared using the log-rank test (**f**–**h**). MAIT, mucosal-associated invariant T. Source data

RNA sequencing (RNA-seq) analysis was performed for 36 patients. The relationships between median PFS and expression of individual protein-coding genes were tested using differential gene analysis. Using ssGSEA, EMT signature, IL6/JAK/STAT3 signature and CAF signature showed a borderline relationship with shorter PFS, whereas immune signatures were related to longer PFS (Fig. 3a). We compared the transcriptomic profile of complete responders versus other patients. Using KEGG pathway 2021 on significantly enriched genes, only immune-related pathways were significantly enriched in responders (chemokine signaling pathway, cytokine–cytokine receptor interaction) (Fig. 3b). MCP-counter^23^, ImmuCellAI^24^ and Kassandra^25^ software were used to describe baseline immune infiltration. All software programs showed a significant or borderline association between T cell infiltration and longer PFS (Fig. 3c–e). Durvalumab targets the interaction between PD-1 and PD-L1, and tremelimumab targets the interaction between CTLA-4 and CD80/CD86. We tested the relation between expression of PD-L1 (*CD274*), PD1 (*PDCD1*), CTLA-4, CD80 and CD86 and PFS. For each variable, the best cutoff using the maximum selective rank statistic method was used to divide patients into high and low score groups. Using this strategy, we observed that only high CTLA-4 mRNA expression was related to better PFS (Fig. 3a,f).

To further explore the respective role of stromal and immune content, we used a decision tree to analyze all immune and stromal signatures associated with PFS with *P* < 0.1 by univariate analysis. Low CXCL10 and high IL6/JAK/STAT3 signature expressions were the most important variables to predict outcome (Fig. 3g,h).

Together, these data underline that a TME enriched in immune cells, T cell chemoattractant chemokines and low stromal signatures are predictive factors of better response to chemo-immunotherapy.

### Exploratory analysis of immunological correlates

**Fig. 4:**
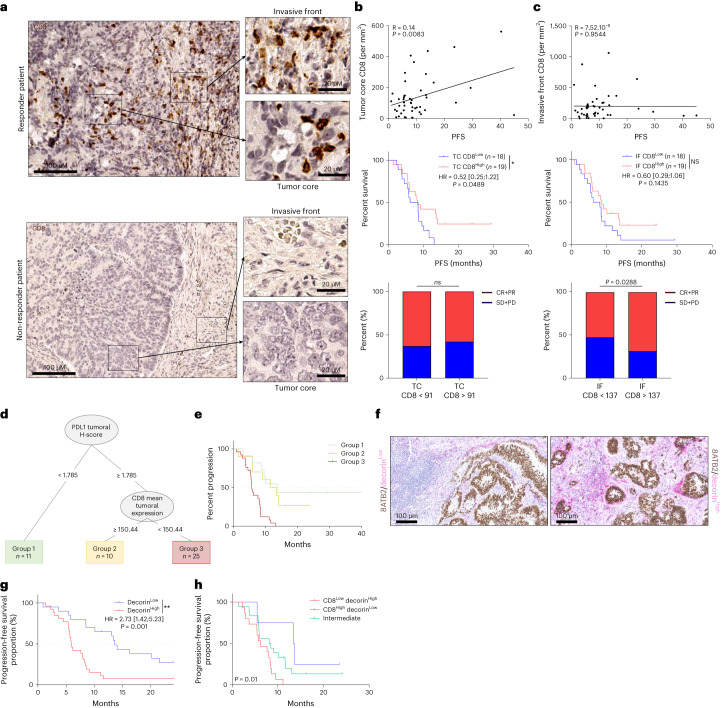
Immunological exploratory analysis. **a**, Representative pictures of CD8 staining of CRC samples from a responder and a non-responder patient (scale bar, 100 µm) focusing on an area of the invasive (IF) front and an area of the tumor core (TC) (scale bar, 20 µm). **b**,**c**, Analysis of CD8^+^ cells in the TC (**b**) and the IF (**c**). Top panel, the correlation between TC (respectively invasive margin) CD8 number per mm^3^ and PFS was determined. Correlation was performed using the Spearman test. Middle panel, Kaplan–Meier curves for PFS with patients stratified according to high or low CD8 number in the TC (respectively invasive margin). The overall median was used as a threshold to distinguish the two groups. Two-sided *P* value with significance level set at 0.05. Bottom panel, bar plots showing the percentage of complete response and partial response (CR + PR) or stable disease and progressive disease (SD + PD) according to the number of CD8 in the tumor core (respectively invasive margin) (*n* = 37). NS, not significant; *P < 0.05, comparison using Fisher’s exact test. **d**, Decision tree for PFS estimated with immunohistochemistry variables. **e**, Kaplan–Meier curves with patients stratified according to groups created by the decision tree for PFS. Two-sided *P* value with significance level set at 0.05. **f**, Representative pictures of decorin/SATB2 staining of CRC samples from a patient with low expression of decorin (left) and a patient with high expression of decorin (right) (scale bar, 100 µm). **g**,**h**, Kaplan–Meier curves for PFS with patients stratified according to decorin protein expression level (**g**) and the combination of CD8 and decorin protein expression level (**h**) (*n* = 47). Two-sided *P* value with significance level set at 0.05. Survival distributions were compared using the log-rank test (**e**,**g**,**h**). Source data

**Extended Data Fig. 3:**
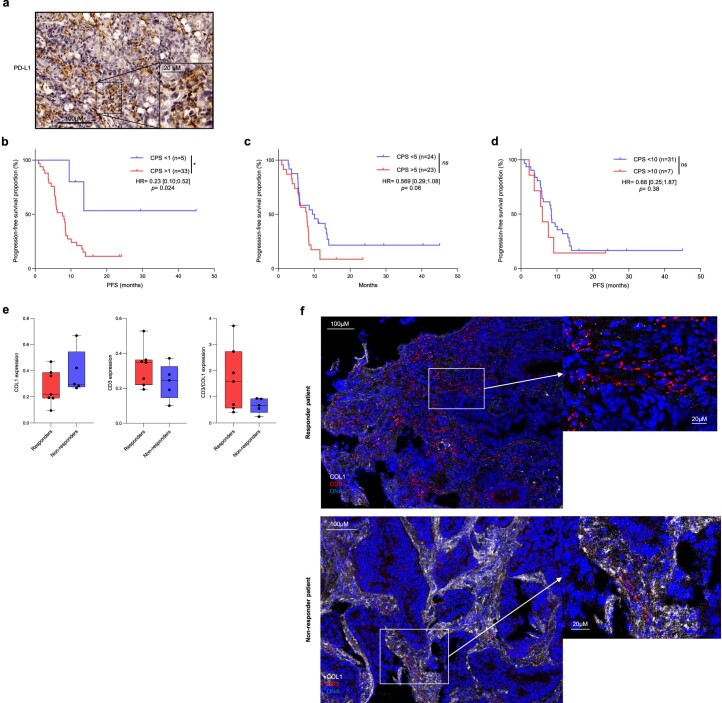
Exploratory analysis of immunological correlates. **a**, Representative pictures of PD-L1 staining of colorectal cancer sample (scale bar 100µM) with a zoom on a part of the blade (scale bar 20 µM). **b**, Kaplan Meier curves for progression-free survival; patients were split into two groups: patients with CPS score < 1 (blue curve) or patients with CPS score > 1 (red curve) (Kaplan-Meier method and log rank tests). **c**, Kaplan Meier curves for progression-free survival; patients were split into two groups: patients with CPS score < 5 (blue curve) or patients with CPS score > 5 (red curve) (Kaplan-Meier method and log rank tests). **d**, Kaplan Meier curves for progression-free survival; patients were split into two groups: patients with CPS score < 10 (blue curve) or patients with CPS score > 10 (red curve) (Kaplan-Meier method and log rank tests). n.s, not significant. **e**, Box plots of COL1 (left), CD3 (middle) protein expression, and CD3/COL1 ratio (right). The center line indicates the median value, lower and upper hinges represent the 25th and 75th percentiles, respectively, and whiskers denote minimum and maximum. Each dot corresponds to one patient (n = 7 responders and n = 5 non-responders). Data were compared using an unpaired two-sided Mann-Whitney Wilcoxon test. **f**, Representative false colour images of COL1 (white) and CD3 (red) markers merged with a nuclear stain (blue) and cropped of colorectal cancer samples from a responder patient (top) and a non-responder patient (bottom) (scale bar 100µM). One area of each sample has been magnified to better appreciate the markers. Source data

PD-L1 CPS expression was not related to outcome (Extended Data Fig. 3a–d). High CD8 infiltration in the tumor core was associated with better PFS, and high CD8 at the invasive margin was associated with a better response rate but not with better PFS (Fig. 4a–c). PD-L1 H-score and CD8 number in the tumor core could be associated to predict PFS. Patients with low PD-L1 H-score and patients with high PD-L1 H-score and high CD8 infiltration in tumor core had longer PFS than other patients (Fig. 4d,e). We tested the expression of decorin, a component of the extracellular matrix highly expressed in inflammatory cancer-associated fibroblasts (iCAFs)^26^. We observed that patients with low decorin had longer PFS (Fig. 4f,g). Combining both CD8 and decorin information, we observed longer PFS in patients with high CD8 and low decorin (Fig. 4h). Using imaging mass cytometry in seven responders and five non-responders, we observed that, whereas responders were enriched in CD3^+^ cells, non-responders have high collagen type 1 (COL1) content in the stromal compartment. The ratio of CD3^+^ to COL1 expression was predictive of objective response and could be a valuable marker of objective response. In non-responders, COL1 appears to represent a protective barrier between cancer cells and immune cells (Extended Data Fig. 3e,f).

**Extended Data Fig. 4:**
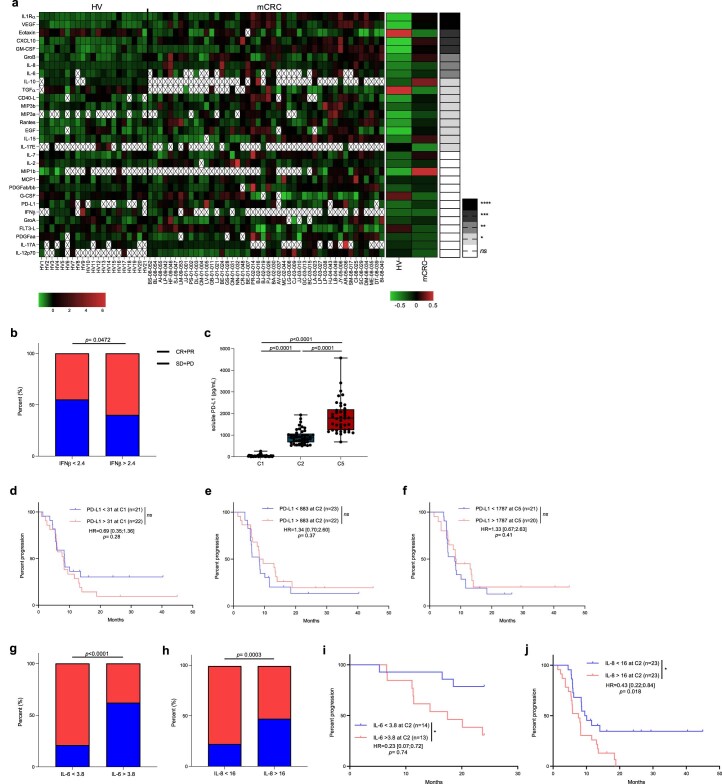
Exploratory analysis of immunological correlates. **a**, Plasma from metastatic colorectal cancer (mCRC) patients was recovered at C1, C2 and C5 and plasma from healthy volunteers (HV) was also recovered. A bioplex assay was performed to analyze the amounts of secreted cytokines. The heat map on the left corresponds to normalized cytokine amount, in the middle is the median of each cytokine between HV and mCRC and on the right is the p-value from statistical analysis. Statistical analysis was performed using the two-sided Wilcoxon t-test. **b**, Bar plots showing the percentage of complete and partial response (CR+PR) or stable and progression disease (SD+PD) according to the amount of IFNβ measured in the patients’ plasma at baseline. n.s, not significant; comparison using two-sided Fisher’s exact test. **c**, Box plots of the soluble PD-L1 assay in the plasma of patients at C1 (baseline), C2 (after one cycle of chemotherapy) and C5 (after 4 cycles of chemotherapy). The center line indicates the median value, lower and upper hinges represent the 25th and 75th percentiles, respectively, and whiskers denote minimum and maximum. Each dot corresponds to one patient (n = 46). Data were compared using an unpaired Mann-Whitney Wilcoxon test. **d-f**, Plasma was recovered at C1, C2 and C5 and a bioplex assay was performed to analyze the amounts of soluble PD-L1 secreted before and during treatment. Kaplan-Meier curves are shown for progression-free survival with patients stratified according to the amount of soluble PD-L1 at C1 (**d**), C2 (**e**) and C5 (**f**). The overall median for each time point was used as a threshold to distinguish the two groups. Survival distributions were compared using the log-rank test (d-f). Two-sided P value with significance level set at 0.05. **g,h**, Bar plots showing the percentage of complete and partial response (CR+PR) or stable and progression disease (SD+PD) according to the amount of IL-6 (**g**) or IL-8 (**h**) measured in the patients’ plasma at C2. Comparison was assessed using Fisher’s exact test. **i,j**, Kaplan-Meier curves for progression-free survival with patients stratified according to IL-6 (**i**) or IL-8 (**j**) secretion level at C2. The overall median was used as a threshold to distinguish the two groups. Two-sided P value with significance level set at 0.05. Source data

Using bioplex assay testing 44 different cytokines (Supplementary File Table 1), we noted that 17 cytokines were highly present in metastatic CRC plasma compared to control patients (Extended Data Fig. 4a). Only high interferon (IFN)-β baseline production was associated with a better response rate (Extended Data Fig. 4b). During therapy, we observed an increased level of soluble PD-L1, albeit unassociated with outcome (Extended Data Fig. 4c–f). After one treatment cycle (C2), high levels of interleukin (IL)-6 and IL-8 were associated with poor response rate (Extended Data Fig. 4g,h) and shorter PFS (Extended Data Fig. 4i,j).

**Extended Data Fig. 5:**
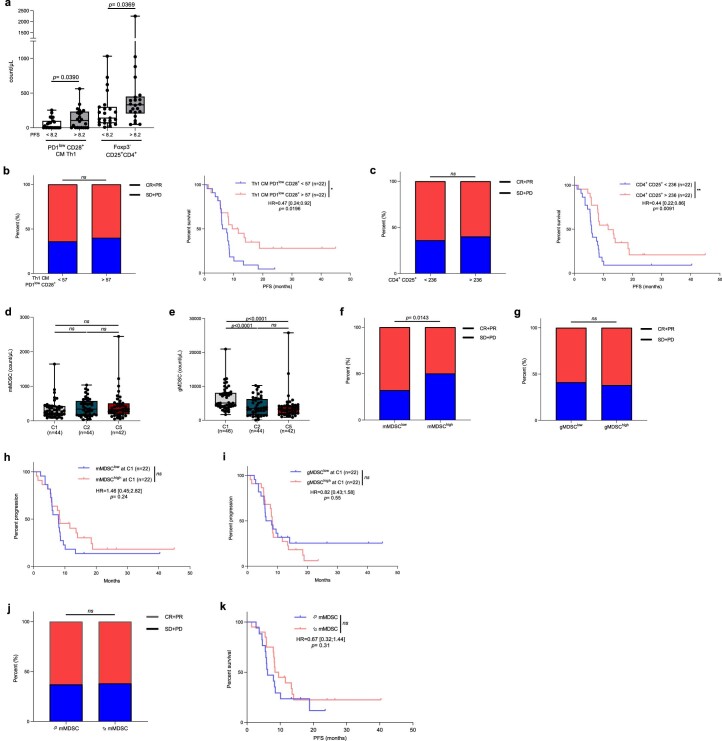
Exploratory analysis of immunological correlates. **a**, Box plots showing the frequency of Th1 central memory (CD4+ CXCR3+ CCR6- CD45RA- CCR7+ PD1low CD28+) and of CD4+ CD25+ Foxp3- cells (count/µL) in patients (n = 45) according to the median of progression-free survival (PFS). **b,c**, Left panel: Bar plots showing the percentage of complete and partial response (CR+PR) or stable and progression disease (SD+PD) according to frequency of Th1 central memory (**b**) or CD4+ CD25+ Foxp3- (**c**). Right panel: Kaplan-Meier curves for progression-free survival with patients stratified according to the frequency of Th1 central memory (**b**) or CD4+ CD25+ Foxp3- (**c**). **d,e**, Box plots showing the frequency of monocytic MDSC (CD45+ CD3- CD19- CD20- CD56- CD15- CD14+ HLA-DRlow) (**d**) and of granulocytic MDSC (CD45+ CD3- CD19- CD20- CD56- CD15+ CD14+) (**e**) (count/µL) at C1, C2 and C5 (n = 46). **f,g**, Bar plots showing the percentage of complete and partial response (CR+PR) or stable and progression disease (SD+PD) according to low or high frequency of mMDSC (**f**) or gMDSC (**g**). **h,i**, Kaplan-Meier curves for progression-free survival with patients stratified according to the frequency of mMDSC (**h**) or gMDSC (**i**) at baseline. **j**, Bar plots showing the percentage of complete and partial response (CR+PR) or stable and progression disease (SD+PD) according to the delta between C5 or C2 and C1 for the frequency of mMDSC measured at baseline. **k**, Kaplan-Meier curves for progression-free survival with patients stratified according to the delta between C5 or C2 and C1 for the frequency of mMDSC. Analysis performed at baseline by flow cytometry in patient’s blood at baseline. For boxplots, center line indicates the median value, lower and upper hinges represent the 25th and 75th percentiles, respectively, and whiskers denote minimum and maximum. Each dot corresponds to one patient. Statistical analysis was performed by unpaired two-sided Mann-Whitney Wilcoxon test (a,d,e) or two-sided Fisher’s exact test (b,c,f,g,j), and Log-rank test (b,c,h, I, k). For continuous variables, the overall median was used as a threshold to distinguish patients into two groups. n.s, not significant. Source data

To understand the systemic immunological effects of treatment, we examined peripheral blood mononuclear cells (PBMCs) in 57 patients with available samples at baseline, at cycle 2 and at cycle 5 (Supplementary File Table 2 and Supplementary File Figs. 1–6); we distinguished a total of 36 immune cell types (Supplementary File Fig. 7). At baseline, only high levels of Foxp3^−^CD4^+^CD25^+^ and of Th1 PD1^low^CD28^+^ central memory cells were associated with better PFS (Extended Data Fig. 5a–c). Monocytic myeloid-derived suppressor cells (mMDSCs) were not affected by the treatment, whereas granulocytic MDSCs (gMDSCs) decreased at C2 and C5 (Extended Data Fig. 5d,e). A high level of mMDSCs, but not gMDSCs, was associated with poor response rate (Extended Data Fig. 5f,g). Neither mMDSCs nor gMDSCs were associated with PFS (Extended Data Fig. 5h,i). Decreased MDSC level during treatment was not associated with either response rate or PFS (Extended Data Fig. 5j,k).

**Fig. 5:**
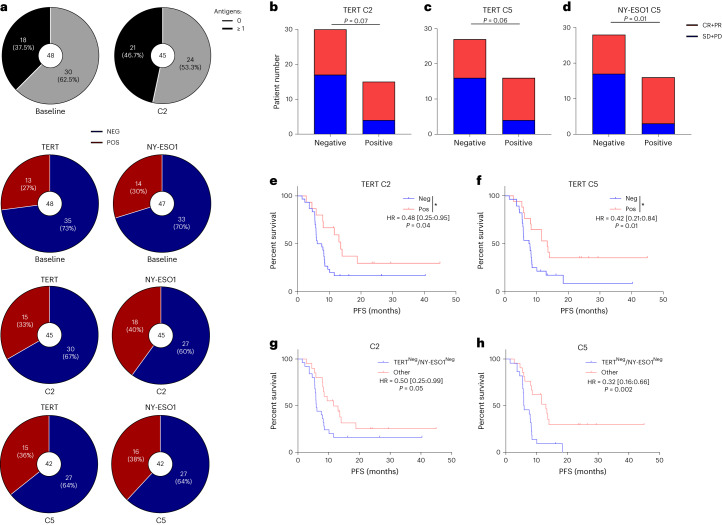
Immunological exploratory analysis. **a**, Upper panel, parts of whole (black and gray) showing the distribution of patients according to their anti-tumor responses against zero or at least one antigen at baseline (*n* = 40) and at C2 (*n* = 45). Lower panel, parts of whole (red and blue) showing the percentage of positive (in red) or negative (in blue) anti-tumor responses against TERT or NY-ESO1 at baseline (*n* = 48), at C2 (*n* = 45) and at C5 (*n* = 42). **b**–**d**, Bar plots showing the number of complete response and partial response (CR + PR) or stable disease and progressive disease (SD + PD) according to TERT-specific T cell responses at C2 (**b**), at C5 (**c**) and NY-ESO1-specific T cell responses at C5 (**d**). Two-sided *P* value with significance level set at 0.05, comparison using Fisher’s exact test. **e**,**f**, Kaplan–Meier curves for PFS with patients stratified according to TERT-specific T cell responses at C2 (**e**) and at C5 (**f**) (*n* = 45). Two-sided *P* value with significance level set at 0.05. **g**,**h** Kaplan–Meier curves for PFS with patients stratified according to the combination of TERT and NY-ESO1-specific T cell responses at C2 (**g**) and at C5 (**h**) (*n* = 45). Two-sided *P* value with significance level set at 0.05. Survival distributions were compared using the log-rank test (**e**–**h**). *P < 0.05. NEG, negative; POS, positive. Source data

**Extended Data Fig. 6:**
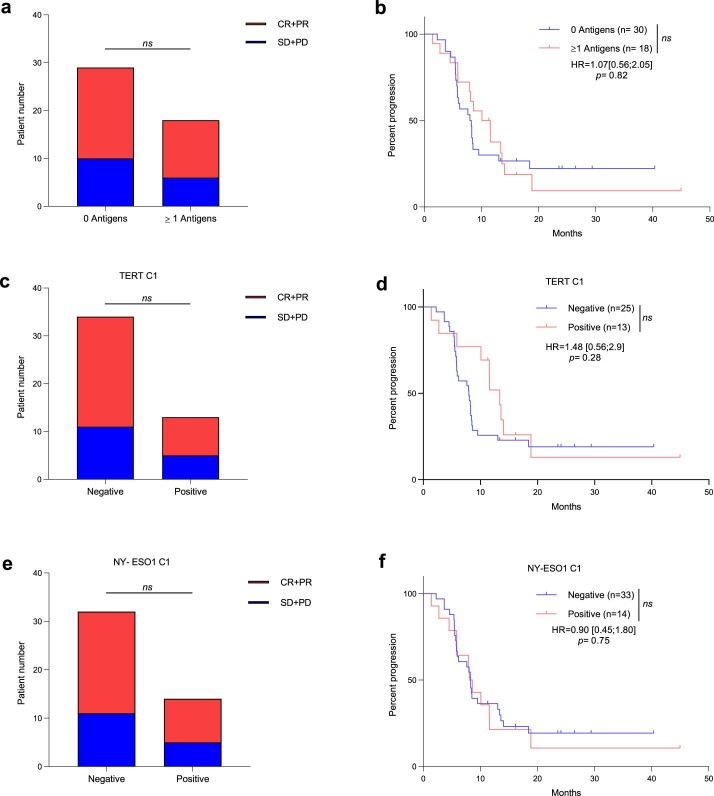
Exploratory analysis of immunological correlates. **a**, Bar plots showing the number of complete and partial response (CR+PR) or stable and progressive disease (SD+PD) according to the antitumor response against 0 or at least 1 antigen at C1 (n = 48). Two-sided P value with significance level set at 0.05, comparison using Fisher’s exact test. **b**, Kaplan-Meier curves for progression-free survival with patients stratified according to the antitumor response against 0 or at least 1 antigen at C1 (n = 48). Two-sided P value with significance level set at 0.05. **c**, Bar plots showing the number of complete and partial response (CR+PR) or stable and progressive disease (SD+PD) according to the antitumor response against TERT-specific T-cell responses at C1 (n = 38). Two-sided P value with significance level set at 0.05, comparison using Fisher’s exact test. **d**, Kaplan-Meier curves for progression-free survival with patients stratified according to the antitumor response against TERT-specific T-cell responses at C1 (n = 38). Two-sided P value with significance level set at 0.05. **e**, Bar plots showing the number of complete and partial response (CR+PR) or stable and progressive disease (SD+PD) according to the antitumor response against NY-ESO1-specific T-cell responses at C1 (n = 47). Two-sided P value with significance level set at 0.05, comparison using Fisher’s exact test. **f**, Kaplan-Meier curves for progression-free survival with patients stratified according to the antitumor response against NY-ESO1-specific T-cell responses at C1 (n = 47). Two-sided P value with significance level set at 0.05. Survival distributions were compared using the log-rank test (b,d,f). Source data

Using ELISpot, we studied anti-tumor-specific T cell responses against shared tumor antigens, telomerase and NY-ESO1 in the blood. We observed that 37.5% of patients presented baseline T cell response against at least one of these two antigens (Fig. 5a). After one treatment cycle, the presence of T cell response against either antigen increased and was detected in 46.7% of patients (Fig. 5a). Baseline responses against these antigens were not associated with objective response or PFS (Extended Data Fig. 6a–f). In contrast, T cell response against telomerase found at C2 and C5 was associated with numerically but non-statistically significantly better objective response and with significantly longer PFS (Fig. 5b,c,e,f). Similarly, induction of T cell response against NY-ESO1 antigen found at C5 was associated with a better response rate (Fig. 5d). Combined analysis of T cell responses against telomerase and NY-ESO-1 showed that the presence of at least one reactivity at C2 or C5 was significantly associated with longer PFS (Fig. 5g,h). These data support that the presence of baseline immune infiltration and induction of immune response against shared tumor antigens is associated with response to therapy.

### Analysis of in situ tumor-specific CD8 response in responders

**Fig. 6:**
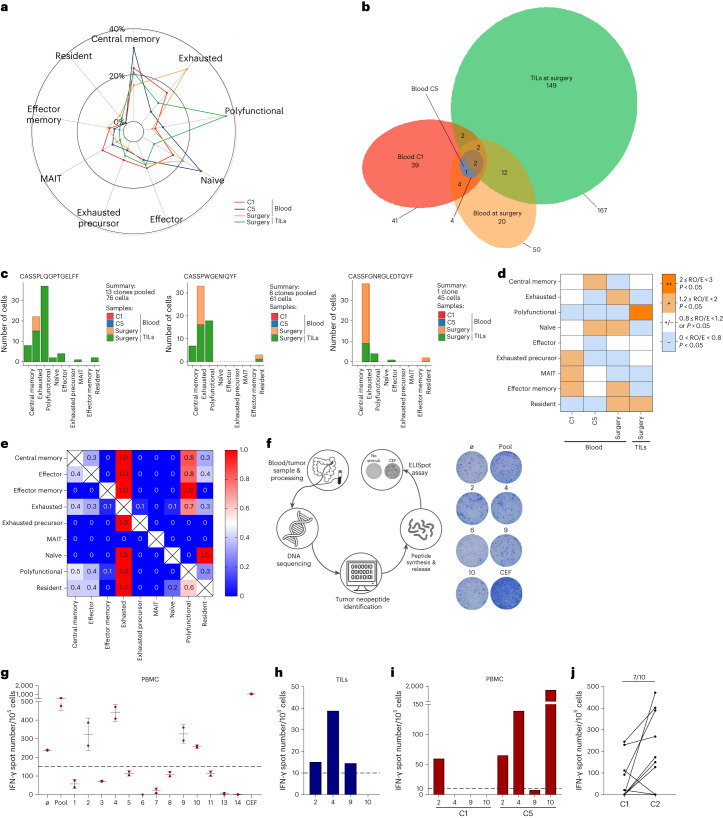
Analysis of in situ tumor-specific CD8 response in responders. **a**, Radar plot showing the proportion of T cell clusters from sampling at C1, at C5 and at the time of surgery in blood and at the time of surgery in TILs. **b**, Venn diagram showing the distribution of TCR clonotypes given the sampling origin. **c**, Bar plots showing the spread of number of cells observed for each T cell population given the sampling origin, for each of the three most frequent TCR clusters, **d**, Sample preference of each cluster estimated by the RO/E index; ++ (2 ≤ RO/E < 3, *P* < 0.05) represents enriched; + (1.2 ≤ RO/E < 2, *P* < 0.05) represents slightly enriched; +/− (0.8 ≤ RO/E < 1.2 or *P* > 0.05) represents non-significant; and − (0 < RO/E < 0.8, *P* < 0.05) represents deletion. **e**, Heat map showing the fraction of T cells with clonotypes belonging to a primary phenotype cluster (rows) that are shared with other secondary phenotype clusters (columns). **f**, Left panel, explanatory diagram of the analysis of the specific T response performed in this responder patient. From a blood and tumor sample, exome sequencing was performed, and, using bioinformatics analysis, neopeptides found only in the tumor were identified. These peptides were synthesized, and the specific T response against these peptides was tested using blood and tumor samples to analyze the appearance of the specific anti-tumor response. Right panel, representative picture of ex vivo IFN-γ ELISpot using PBMCs taken at the time of liver surgery. **g**, Dot plot representing the number of IFN-γ spots for each condition (negative control, peptide pool, single peptide 1–14 and positive control (CEF pool)) in PBMCs at the time of liver surgery. Each number corresponds to the tested neoantigen. Ø, dimethyl sulfoxide; CEF, peptides from cytomegalovirus. Epstein–Barr virus and influenza virus and pool corresponds to the pool of tested neoantigens. Dots represent technical replicates. Data are the mean ± s.d. **h**,**i**, Bar graph representing the number of IFN-γ spots for peptides 2, 4, 9 and 10 in TILs from liver metastasis (**i**) and in PBMCs taken at baseline (C1) and after four cycles of chemotherapy (C5) (**j**). **j**, Dot plot representing the number of IFN-γ spots in PBMCs from patients collected at baseline or after two cycles of chemotherapy (C2) after stimulation with a pool of calculating tumor neoantigens (*n* = 10). MAIT, mucosal-associated invariant T. Source data

**Extended Data Fig. 7:**
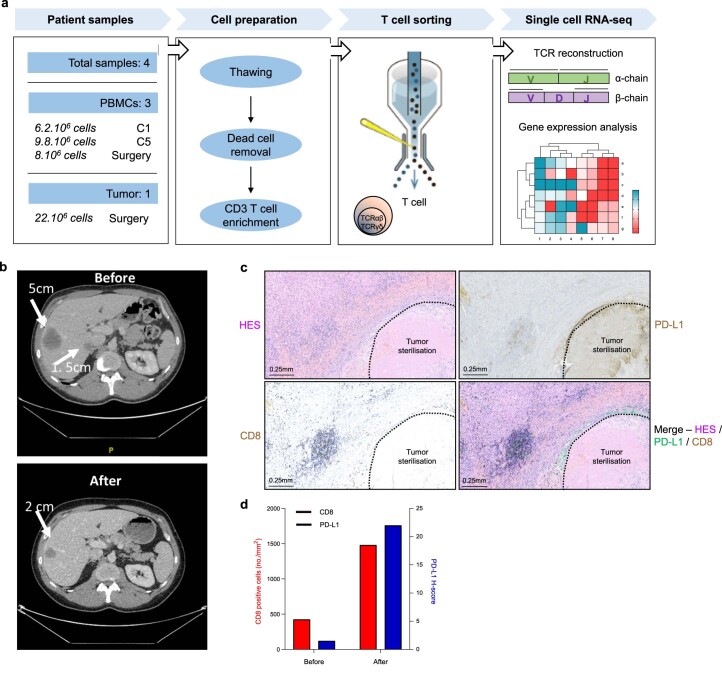
Analysis of in situ tumor specific CD8 response in responders. **a**, Experimental strategy for single cell RNA sequencing on responder patient PBMC and TIL samples. **b**, Contrast enhanced CT-scan showing liver metastases at baseline and before liver surgery. **c**, Upper left: Representative picture of HES staining of a liver metastasis with delimitation of the sterilisation area of the tumour; Upper right: Representative picture of a PD-L1 staining by immunohistochemistry; Lower left: Representative picture of a CD8 staining by immunohistochemistry; Lower right: Merge of the 3 stainings (HES, CD8 and PD-L1) to visualize the spatial distribution of PD-L1 at the tumor sterilisation zone and the presence of a large number of CD8 in the proximity of the tumor. (Scale bar indicates 0.25 mm). **d**, Number of CD8 cells/mm² and H-score of PD-L1 before (in liver biopsy at baseline) and after liver surgery (at the time of surgery). Source data

**Extended Data Fig. 8:**
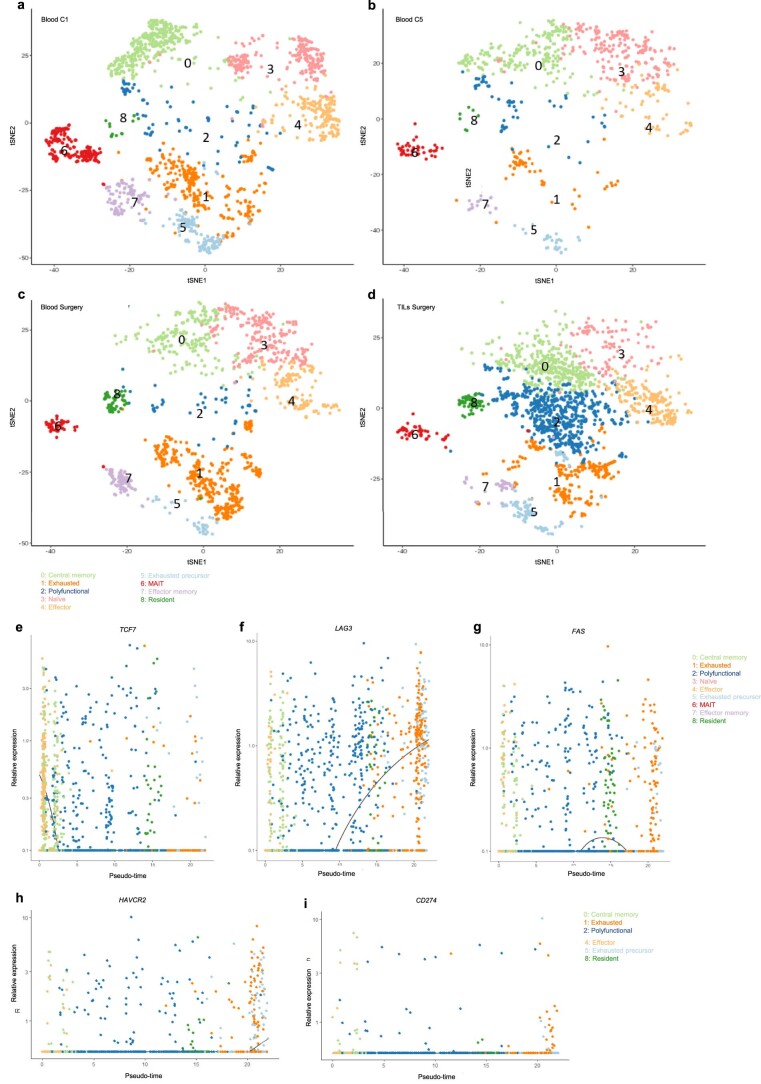
Analysis of in situ tumor specific CD8 response in responders. **a-d**, For each sampling, T-SNE visualization of CD8 T cells analyzed using single-cell RNA-sequencing and colored by cluster identity. Each dot corresponds to one single cell and 9 clusters were selected: 0: Central memory; 1: Exhausted; 2: Polyfunctional; 3: Naïve; 4: Effector; 5: Exhausted precursor; 6: MAIT (Mucosal-Associated Invariant T cells); 7: Effector memory; 8: Resident. **e-i**, Relative expression of TCF7 (**e**), LAG3 (**f**), FAS (**g**), HAVCR2 (**h**) and PDCD1 (**i**) genes according to pseudotimes; points are colored by T-cell clusters for blood surgery sampling. Source data

Among patients with partial response, one patient who underwent resection of remaining liver metastases was monitored using single-cell RNA sequencing (scRNA-seq) and T cell receptor (TCR) sequencing. PBMCs were taken at baseline, at 1 month and at the time of liver surgery. Tumor-infiltrated lymphocytes (TILs) were isolated from liver metastases (Extended Data Fig. 7a). The patient was diagnosed with synchronous rectal cancer with three liver metastases and peri-aortic lymph nodes. The patient received 6 months of therapy. Peri-aortic lymph nodes and one liver metastasis disappeared (Extended Data Fig. 7b). We observed partial response in the two remaining liver metastases. The patient underwent surgery. Histology showed complete necrosis with no remaining live tumor cells (Extended Data Fig. 7c). After more than 3 years of follow-up, the patient did not relapse. We observed a strong accumulation of CD8 T cells and PD-L1^+^ macrophages around tumor necrosis compared to baseline biopsy (Extended Data Fig. 7d). Using scRNA-seq, we analyzed blood and TIL CD8 T cell subsets. After quality control, we obtained the scRNA-seq profiles of 935 T cells with 262 paired TCR sequences (Supplementary File Table 3). Using unsupervised graph-based clustering, we observed nine CD8 T cell clusters (Extended Data Fig. 8a–d). The distributions were heterogeneous between blood and tumor. In the tumor, we observed an accumulation of a cluster of polyfunctional CD8 T cells with high expression of effector cytokine and cytotoxic molecules. In contrast, in the blood, we observed accumulation of naive T cells. During treatment, accumulation of central memory cells was observed at 1 month and accumulation of exhausted T cells after 6 months at the time of liver surgery, suggesting exhaustion of the immune response (Fig. 6a). Most clonal T cells were distributed in TILs (167) in contrast to blood samples (4–50). Only two clones were present in all samples (Fig. 6b). Two clones were shared between baseline blood sample and TILs. In contrast, 12 clonotypes were shared between TILs and blood at the time of surgery, suggesting that these clones were induced during treatment (Fig. 6b). We used GLIPH software^27^, which clusters similar TCRs sharing CDR3 motifs predicted to bind the same major histocompatability complex (MHC)-restricted peptide antigen. Among the seven most frequent clusters, only three pooled different T cell clones (from three to 13) (Supplementary File Table 4). These clones were detected only in TILs and in the blood at the time of liver surgery and were mainly polyfunctional and exhausted (Fig. 6c). Cluster preference analysis underlined that polyfunctional T cell clones were enriched in TILs (Fig. 6d). These data support the rationale for induction of an anti-tumoral immune response in PBMCs and TILs, which induce tumor-specific clones with a polyfunctional phenotype in TILs and an exhausted phenotype in PBMCs.

We then investigated the clonal state transition. Exhausted and polyfunctional phenotypes share various clusters (Fig. 6e), suggesting that such clusters in the TME usually undergo extensive state transitions. To confirm transition states, cells were ordered into a branched pseudotime trajectory using Monocle (version 2.12.0). Pseudotime ordering of CD8 T cells in TILs showed that central memory cells diverge toward either resident or exhausted T cells, whereas polyfunctional cells represent an intermediate state (Extended Data Fig. 8e–i). These results support the rationale that clonal T cells found at the tumor site after treatment initiation are intermediate cells with stemness capability and a low exhaustion profile, prone to mount an anti-tumor immune response.

Using exome sequencing of the tumor, we detected 28 non-synonymous mutations. Using pVAC-Seq software, we predicted 14 strong HLA binder neoantigens (Fig. 6f and Supplementary File Table 5). Using ELISpot, we tested whether blood CD8 taken at the time of liver surgery could respond against each individual neopeptide (Fig. 6f,g). We observed reactivity against four neopeptides that are present in AP2-γ, Trim-17, Jip-4 and Mucin-4 proteins (Fig. 6g). Significant reactivity against three neopeptides was found in TILs (Fig. 6h). In the blood, we observed a response against only one of these four neopeptides at baseline, but a response against three neopeptides occurred after 1 month of treatment (Fig. 6i). We were able to test the peripheral immune response against tumor-derived neopeptides in 10 patients. PBMCs collected at baseline and at C2 were stimulated with neopeptides (one to eight depending on the patient; Supplementary File Table 6). Seven of 10 patients showed enhanced immune response with neopeptides at C2 (Fig. 6j). These results indicate that the chemo-immunotherapy protocol could amplify and generate neoantigen-specific CD8 T cell immune response, which can be detected either in the blood or in the tumor.

## Discussion

This study reports clinical and biological response with first-line chemo-immunotherapy for RAS-mutated metastatic CRC. This study reached its primary objective, with 3-month PFS of 90.7%, 6-month PFS of 60% and median PFS of 8.2 months, whereas the expected median PFS for such a population is 5–6 months with FOLFOX alone. This protocol yielded similar results to those observed with a chemotherapy doublet with bevacizumab, which gave around 8 months of PFS^28^. The different results in term of PFS in metastatic CRC clinical trials must be mitigated by several considerations. First, most studies described above included both wild-type (WT) and RAS-mutated patients, and *RAS* mutant metastatic CRC is known to have poorer prognosis (median survival, 28 months) compared to RAS WT patients, who have median OS of approximately 33 months^29,30^. In a recent trial comparing FOLFOX to FOLFOX-bevacizumab only in KRAS-mutated patients, the FOLFOX group had PFS of only 5.6 months^31^.

In terms of response rate, our study reports an objective response rate of 63%, comparing favorably with the reported 36% in mutant RAS tumors treated with FOLFOX monotherapy (BECOME study). Our trial yielded one of the best objective response rates in the literature to date for bi-chemotherapy treatments^32^ (Supplementary File Fig. 8). Furthermore, the response rate in the MEDITREME trial is similar to that observed in RAS mutant tumors treated with FOLFOXIRI-bevacizumab, which achieved an objective response rate of 65%^28^, suggesting that chemotherapy intensification may yield a similar response to our chemo-immunotherapy protocol. Another particularity is that 15% of our patients had durable complete remission after this treatment compared to 5% using FOLFIRINOX-bevacizumab^28^. This suggests that, contrary to a chemotherapy regimen, chemo-immunotherapy might trigger cure in a small subset of patients. In addition, in most trials, patients received chemotherapy for at least 8–12 cycles or were permanently treated with chemotherapy, whereas, in our study, patients received only six chemotherapy cycles. A short course of chemotherapy might be beneficial in terms of side effects by shortening the period of exposure combined with chemotherapy-free intervals. Consequently, 90% of grade 3/4 side effects observed in our study occurred during the on-chemotherapy period.

MSS CRCs represent 95% of all metastatic CRC and are characterized by low TMB and low immune infiltration compared to MSI CRC^33^. Moreover, KRAS mutation is associated with decreased CD8 T infiltration and HLA expression in CRC, and, consequently, KRAS mutant tumor cells have a lower chance of being recognized by T cells^34^.

Two clinical trials (REGOMUNE and REGONIVO) suggest that immune checkpoint inhibitors may harbor some features of efficacy in patients with MSS tumors, especially in the absence of liver metastases, which are an immune resistance factor^35–37^. Similar results were observed with the combination of an anti-CTLA-4 and an anti-PD1 (botensilimab + balstilimab) in third-line MSS CRC, which showed signs of efficacy but with a detrimental role of liver metastases on the efficacy of anti-PD-1 + anti-CTLA-4 ^38^. Liver metastases are suspected to be a general risk factor for checkpoint inhibitor resistance due to elimination of anti-tumor T cells by intrahepatic macrophages^39^. In contrast to these previous reports, our data support the effectiveness of chemo-immunotherapy in MSS CRC, regardless of clinical characteristics, notably the presence of liver metastases. Our data are congruent with previous literature showing that accumulation of CD3 and CD8 T cells in the invasive margin and in liver metastases of CRC is related to outcome^40–45^.

Our study underlines that CTLA-4 expression at the tumor site is associated with better response. Previous reports testing botensilimab plus balstilimab or durvalumab plus tremelimumab or radiotherapy plus nivolumab and ipilimumab in third-line CRC^38,46,47^ suggest some level of efficacy in contrast to anti-PD-1/PD-L1 alone. Our data reinforce these findings and provide a rationale for this clinical observation.

High immune infiltrate on transcriptomic analysis, plus a combination of high CD8 and high PD-L1, were associated with outcome in our study. These findings mirror those of the GONO group, which observed in AtezoTRIBE that patients with high Immunoscore yielded a benefit from atezolizumab plus FOLFOXIRI^48^. We observed that, in addition to immune signature, low presence of iCAFs and a low decorin level were associated with better response. A previous study reported that a group of MSS mucinous tumors could respond to immunotherapy^49^, suggesting a link between the CRC phenotype and response to immune checkpoint inhibitors. iCAF and decorin are related to poor prognosis, and, by imaging mass cytometry, poor responders present high collagen expression, which may represent a barrier between T cells and tumor cells. These data support the posit that analysis of both baseline immune infiltrate and fibroblastic reaction could be important in predicting the efficacy of chemo-immunotherapy.

NY-ESO1 and telomerase immune response has previously been reported in CRC, with spontaneous response detected in 24% of patients for telomerase^50,51^ and 20% for NY-ESO1, in line with our results. In patients receiving FOLFOX monotherapy, no induction of specific T cell response against shared antigen was reported in these studies. Conversely, in our trial, we observed induction of both NY-ESO1 and telomerase tumor-specific immune response after chemo-immunotherapy. Moreover, although baseline response against shared antigens was not associated with outcome, induction of telomerase or NY-ESO1 immune response was associated with chemo-immunotherapy efficacy. In addition to response against shared antigens, we observed that chemo-immunotherapy triggers a peripheral T cell response against tumor neoantigens. Together, these data support the rationale that chemo-immunotherapy could promote immune response against shared tumor antigens and neoantigens in MSS metastatic CRC and that this immune response is associated with response to therapy. Finally, single-cell and genomic analysis of long responder patients demonstrated that, in addition to generating T cell response against shared neoantigens, this chemo-immunotherapy protocol induced and amplified tumor-specific neopeptide immune response at the tumor site and in the periphery.

This study is limited by its small sample size and the absence of a FOLFOX monotherapy control arm. However, the clinical data compared favorably to previous trials of doublet chemotherapy alone. Because of the lack of control group and small sample size, genomic and transcriptomic data must be taken as exploratory, and the predictive versus prognostic nature of our results warrants confirmation in larger randomized trials. Due to the absence of a control group without oxaliplatin, the role of immunogenic cell death has not been directly proven.

In summary, we report favorable clinical efficacy with first-line chemo-immunotherapy for unresectable MSS metastatic CRC, with in-depth molecular and immune analyses, providing clues for better selection of patients with MSS metastatic CRC for chemo-immunotherapy, with potentially broad clinical implications.

## Methods

### Trial registration

The ‘Evaluation of the Safety and the Tolerability of Durvalumab Plus Tremelimumab Combined with FOLFOX in mCRC (MEDITREME)’ trial was prospectively registered with ClinicalTrials.gov identifier NCT03202758 (EudraCT: 2016-005006-19).

### Inclusion and ethics

The protocol was approved by the Ethics Committee CPP TOURS – Région Centre – Ouest 1 on 27 March 2017 under the number 2017T1-03 and was registered with the French national health products agency (ANSM). The French ethical authorities asked us to include only RAS-mutated metastatic CRC because RAS WT patients must receive an anti-EGFR in first-line of treatment, and it would be unethical to give a protocol without anti-EGFR for these patients. The study was conducted in accordance with the Declaration of Helsinki and International Conference on Harmonization for Good Clinical Practice guidelines and the CONSORT 2010 guidelines. All patients provided written informed consent to study procedures before enrollment. The study protocol was previously published elsewhere^52^. The last version of the protocol is available as supplementary information.

### Patient selection

Patients were enrolled in eight hospitals in France (Georges-François Leclerc Anticancer Center, UNICANCER, Dijon; Hôpital Franco-Britannique – Fondation Cognacq-Jay, Levallois-Perret; CHU, Nantes; CHU, Besançon; Clinique CARIO, Plérin; Saint Antoine, Hospital, Paris; Institut Bergonie, Bordeaux; and Pompidou Hospital, Paris).

The full inclusion and exclusion criteria are listed below.

#### Inclusion criteria

1. Written informed consent and any locally required authorization obtained from the patient before performing any protocol-related procedures, including screening evaluations
2. Male or female age ≥18 years at time of study entry
3. Performance status of 0 or 1 according to the Eastern Cooperative Oncology Group and World Health Organization
4. Histologically confirmed diagnoses of CRC with positive mutated KRas or NRas
5. Patients with metastatic disease
6. First-line metastatic disease or first-line after localized disease treated by local curative treatment, with or without adjuvant chemotherapy by FOLFOX. Reccurence after the last dose of adjuvant chemotherapy should be ≥ 6 months. Previous perioperative chemotherapy for resecable metastasis is not permitted.
7. Life expectancy of more than 12 weeks
8. Adequate normal organ and marrow function as defined below:
  Hemoglobin > 9.0 g dl^−1^
  Absolute neutrophil count (ANC) > 1.5 × 10^9^ per L (>1,500 per mm^3^)
  Platelet count > 100 × 10^9^ per L (≥100,000 per mm^3^)
  Serum bilirubin ≤1.5× the institutional upper limit of normal (ULN)
  AST (SGOT)/ALT (SGPT) ≤ 2.5× the institutional ULN unless liver metastases are present, in which case it must be ≤5× ULN
  PAL ≤ 5× institutional ULN unless liver metastases are present, in which case it must be ≤20× ULN
  Albumin > 30 g L^−1^
  Creatinine < 1.5× institutional ULN
  Serum creatinine CL > 40 ml min^−1^ by the Cockcroft–Gault formula (Cockcroft and Gault, 1976) or by 24-h urine collection for determination of creatinine clearance:

**Table:** 

	Males	Females
Creatinine CL (ml min^−1^)	Weight (kg) × (140–age). 72 × serum creatinine (mg dl^−1^)	Weight (kg) × (140–age) × 0.85 72 × serum creatinine (mg dl^−1^)
9. Tumor evaluation (computed tomography (CT) scan) in the previous 4 weeks with presence of at least one measurable lesion according to RECIST version 1.1
10. At least 4 weeks since the last chemotherapy, immunotherapy or other drug therapy and/or radiotherapy
11. Recovery to grade ≤1 from any AE derived from previous treatment according to National Cancer Institute Common Terminology Criteria for Adverse Events (NCI-CTCAE) version 4.0
12. For principal study: willingness to provide consent for use of archived tissue with sufficient material available for analysis. For ancillary study: metastasis should be accessible to performed biopsy.
13. Female patients must either be of non-reproductive potential (that is, post-menopausal by history: ≥60 years of age and no menses for ≥1 year without an alternative medical cause, or history of hysterectomy, or history of bilateral tubal ligation, or history of bilateral oophorectomy) or must have a negative serum pregnancy test upon study entry
14. Patients must be affiliated with a social security system
15. Patient is willing and able to comply with the protocol for the duration of the study, including undergoing treatment and scheduled visits and examinations, including follow up

#### Exclusion criteria

1. Involvement in the planning and/or conduct of the study (applies to both AstraZeneca staff and/or staff at the study site). Previous enrollment in the present study.
2. Participation in another clinical study with an investigational product during the last 4 weeks
3. Any previous treatment with a PD-1 or PD-L1/CTLA-4 inhibitor, including durvalumab or tremelimumab
4. History of another malignancy within the five previous years with low potential risk for recurrence other than:
  Adequately treated non-melanoma skin cancer or lentigo maligna without evidence of disease
  Adequately treated carcinoma in situ without evidence of disease (for example, cervical cancer in situ)
5. Receipt of the last dose of anti-cancer therapy (chemotherapy, immunotherapy, endocrine therapy, targeted therapy, biologic therapy, tumor embolization, monoclonal antibodies, other investigational agent) 28 d before the first dose of study drug (14 d before the first dose of study drug for patients who have received prior tyrosine kinase inhibitors (TKIs) (for example, erlotinib, gefitinib and crizotinib)) and within 6 weeks for nitrosourea or mitomycin C. (If sufficient wash-out time has not occurred due to the schedule or pharmacokinetics properties of an agent, a longer wash-out period may be required.)
6. Mean QT interval corrected for heart rate (QTc) ≥ 470 ms calculated from three electrocardiograms (ECGs) using Frediricia’s correction
7. Current or prior use of immunosuppressive medication within 28 d before the first dose of durvalumab, with the exceptions of intranasal and inhaled corticosteroids or systemic corticosteroids at physiological doses, which are not to exceed 10 mg per day of prednisone or an equivalent corticosteroid
8. Any history of hypersensitivity to durvalumab or tremelimumab, FOLFOX or their excipients
9. Any unresolved toxicity (CTCAE grade >1) from previous anti-cancer therapy. Patients with irreversible toxicity that is not reasonably expected to be exacerbated by the investigational product may be included (for example, hearing loss and peripherally neuropathy).
10. Any prior grade ≥3 immune-related adverse event (irAE) while receiving any previous immunotherapy agent or any unresolved grade >1 irAE
11. Active or prior documented autoimmune disease within the past 2 years. Note: Patients with vitiligo, Grave’s disease or psoriasis not requiring systemic treatment (within the past 2 years) are not excluded.
12. Active or prior documented inflammatory bowel disease (for example, Crohn’s disease and ulcerative colitis)
13. History of primary immunodeficiency
14. History of organ transplant that requires use of immunosuppressive
15. History of allogeneic organ transplant
16. Uncontrolled intercurrent illness, including, but not limited to, ongoing or active infection. Clinically significant cardiovascular disease, including myocardial infarction within 6 months; symptomatic congestive heart failure; uncontrolled hypertension; unstable angina pectoris; cardiac arrhythmia; history of Mobitz II second degree or third degree heart block without a permanent pacemaker in place; hypotension; rest limb claudication or ischemia within 6 months; active peptic ulcer disease or gastritis; active bleeding diatheses, including any patient known to have evidence of acute or chronic hepatitis B, hepatitis C or HIV; or psychiatric illness/social situations that would limit compliance with study requirements or compromise the ability of the patient to give written informed consent.
17. Severe concurrent disease or comorbidity that, in the judgment of the investigator, would make the patient inappropriate for enrollment
18. Ongoing treatment with CYP3A4 substrates or regular taking of grapefruit juice
19. Known history of active tuberculosis
20. History of leptomeningeal carcinomatosis
21. Brain metastases or spinal cord compression
22. Receipt of live attenuated vaccination within 30 d before study entry or within 30 d of receiving durvalumab
23. Female patients who are pregnant or breast-feeding or male or female patients of reproductive potential who are not employing an effective method of birth control
24. Any condition that, in the opinion of the investigator, would interfere with evaluation of study treatment or interpretation of patient safety or study results
25. Symptomatic or uncontrolled brain metastases requiring concurrent treatment, inclusive of, but not limited to, surgery, radiation and/or corticosteroids
26. Patients with uncontrolled seizures
27. Patients under guardianship, curatorship or judicial protection
28. Known allergy or hypersensitivity to investigational product or any excipient
29. Patients with tumors that invade major vessels, as shown unequivocally by imaging studies
30. Patients with central lung metastases (that is, within 2 cm from the hilum) that are cavitary, as shown unequivocally by imaging studies
31. Patients with any prior history of bleeding related to the current CRC
32. Patients with a history of gross hemoptysis (bright red blood of ½ teaspoon or more per episode of coughing) ≤3 months before enrollment
33. Patients with a recurrence delay less than 6 months after the adjuvant chemotherapy
34. Patients with resecable disease

### Study design and statistical hypothesis

This was a multicenter, single-arm, open-label, phase 1/2 study. The study was performed in two steps (Extended Data Fig. 1a). The primary objective of step 1 was to determine the safety of the combination of durvalumab (anti-PD-L1) + tremelimumab (anti-CTLA-4) + mFOLFOX6. Toxicity was assessed on the first nine patients within two cycles (30 d) after the first administration of durvalumab + tremelimumab + mFOLFOX6. Toxicity was defined as an AE that may be linked to one of the study drugs.

The primary objective of step 2 was to determine efficacy of the combination of durvalumab (anti-PD-L1) + tremelimumab (anti-CTLA-4) + mFOLFOX6 in terms of PFS in patients with colorectal MSS disease. The secondary objectives were:

- to determine efficacy of the combination of durvalumab (anti-PDL1) + tremelimumab (anti-CTLA-4) + mFOLFOX6 in terms of response to treatment and OS in patients with colorectal MSS disease.
- to determine efficacy of the combination of durvalumab (anti-PD-L1) + tremelimumab (anti-CTLA-4) + mFOLFOX6 in terms of PFS, response to treatment and OS in patients with colorectal MSI disease.

Sample size calculation was performed using PASSV13 (NCSS statistical software), using the following null and alternative hypothesis for primary objective of step 2: H0: 50% of PFS at 3 months; H1: 70.7% PFS at 3 months (equivalent to 50% of PFS at 6 months). According to Simon’s design, with α = 10% and β = 10% (90% power), 43 patients with MSS disease are needed for the primary endpoint analysis. Accounting for 20% of non-evaluable patients, 52 patients with MSS disease will be included. Among these 52 patients, the first nine patients were used for the safety population analysis. The prevalence of MSS disease being around 90–95%, five additional patients will be included, leading to 57 patients in the overall study.

### Procedures

Patients received first-line induction with mFOLFOX6 consisting of an intravenous infusion^53^ of 85 mg m^−^^2^ oxaliplatin^53^ and 200 mg m^−^^2^ leucovorin^53^, followed by 400 mg m^−^^2^ 5-FU administered as a bolus injection, followed by 2,400 mg m^−^^2^ 5-FU administered as an intravenous infusion over 46 h. Six cycles of FOLFOX were administrated. During this period, patients received 750 mg of durvalumab via intravenous infusion every 2 weeks for up to eight doses per cycle and 75 mg of tremelimumab via intravenous infusion every 4 weeks for up to four doses per cycle and then continued 750 mg of durvalumab every 2 weeks starting on week 16 for up to 8 months (18 doses). Immunotherapy was injected before chemotherapy. Re-introduction of mFOLFOX6 durvalumab and tremelimumab was authorized after more than 6 months of stable disease under durvalumab at the discretion of the investigator.

Clinical data were collected at the Department of Statistics of the Centre Georges-François Leclerc. Tumor assessments were based on investigator-reported measurements and were performed according to RECIST version 1.1 and repeated every 12 weeks. Safety was monitored continuously throughout the study. All AEs were recorded and classified according to CTCAE version 4.0, regardless of relation to the study drugs. An independent safety monitoring committee periodically reviewed the study safety data.

Peripheral blood samples were collected in 10-ml cell preparation tubes (BD Biosciences) at baseline and at weeks 3, 9 and 23. All samples were processed within 4–6 h after collection.

Blood samples for isolation of PBMCs were collected longitudinally at participating clinical sites, shipped overnight and processed at a central site (on the biomonitoring platform at Besançon) over a Ficoll gradient and cryopreserved. Serum was processed within 2 h of collection at each site and frozen immediately at −80 °C and then batch shipped to a central biorepository. Blood sampling for immune biomarkers occurred during screening, at cycle 1 days 1 and 15, at cycle 3 day 1 and at cycle 6 day 15 and at treatment discontinuation. These samples were shipped overnight and processed at a central site (on the Cancer Biology Transfer Platform at Dijon). If a patient began any new anti-cancer therapy before their end-of-treatment visit, samples were not collected. Baseline or archival as well as post-treatment tumor specimens were collected for biomarker analyses. Fresh tumor biopsies were immediately formalin-fixed and paraffin-embedded (FFPE). For patients who agreed, an on-treatment biopsy (at cycle 3) could be performed and were also FFPE. Additional biopsies were allowed for patients who had prolonged stable disease, defined as more than two consecutive disease assessments demonstrating response by RECIST version 1.1 as well as at the time of disease progression. Ad hoc biopsy collection was permitted with the approval of the medical monitor.

### Plasma collection

After the blood sampling was done at the different times described above, a heparin tube was used to isolate and bank the plasma. For this purpose, after collection, the heparin tube was centrifuged at 1,000*g* for 10 min at room temperature. The plasma was then recovered, aliquoted at a rate of 500 µl per cryotube and stored at −80 °C until use.

### PBMC isolation

After blood sampling in EDTA tubes, PBMCs were isolated from the whole blood by density gradient centrifugation (Lymphocyte Separation Medium, CMSMSL0101, Eurobio) with SepMate tubes (85460, STEMCELL Technologies). Whole blood was transferred into SepMate tubes at a rate of 17 ml of whole blood per tube and then centrifuged at 1,200*g* for 10 min with an acceleration of 5 and the brake off. After removing as much plasma as possible, the phase containing the enriched PBMCs could be recovered. After washing with 45 ml of PBS, centrifugation of 300*g* for 7 min was carried out, and the PBMC pellet was resuspended in 5 ml of PBS 1× for counting. Then, a final wash with 10 ml of PBS 1× was performed before cryopreservation, which consisted of freezing at a rate of 8.10^6^ cells per cryotube in a solution of 50% FBS, 40% RPMI and 10% DMSO until further use.

### Cytometry analysis

At each blood sample, before or during the patient’s treatment, we performed immunophenotyping by flow cytometry.

#### Blood count analysis

Antibodies for blood count analysis: multi-color flow cytometry was performed using Beckman Coulter’s custom design service and its dry coating technology, and custom tubes containing anti-CD16-FITC (clone 3G8), anti-CD56-PE (clone N901), anti-CD19-PE-Cy5.5 (clone J3-119), anti-CD14-PE-Cy7 (clone RMO52), anti-CD4-APC (clone 13B8.2), anti-CD8-Alexa Fluor 700 (clone B9.11), anti-CD3-APC-Alexa Fluor 750 (clone UCTH1), anti-CD15-PacificBlue (clone 80H5) and anti-CD45-KromeOrange (clone J.33) were produced.

Staining protocol: 100 μl of total heparinized blood was added to a DURAClone tube, vortexed immediately for 15 s and incubated for 15 min at room temperature in the dark. Two milliliters of red blood lysis solution (VersaLyse solution, A09777, Beckman Coulter) containing 50 μl of the fixative agent IOTest 3 Fixative Solution (A07800, Beckman Coulter) was added, inverted and incubated for 15 min in the dark. Then, 100 µl of counting beads (Flow-Count Fluorospheres, 7547053, Beckman Coulter) was added before acquisition on a Canto II cytometer (BD Biosciences).

#### Immune cell populations identification

To decipher the peripheral immune system, we performed five panels to identify and characterize the different lymphocyte and myeloid subpopulations.

Antibodies for T cell analysis (first panel): using Beckman Coulter’s custom design service and its dry coating technology, custom tubes containing anti-CD183-FITC (clone G025H7), anti-CD197-PE (clone G043H7), anti-CD196-PE-Cy7 (clone B-R35), anti-CD278-APC (clone ISA-3), anti-CD45RA-Alexa Fluor 700 (clone 2H4LDH11LDB9 (2H4)), anti-HLA-DR-APC-Alexa Fluor 750 (clone Immu-357), anti-CD4-PacificBlue (clone 13B8.2) and anti-CD8-KromeOrange (clone B9.11) were produced. Liquid antibodies were also used: anti-CCR4-PerCP-Cy5.5 (BioLegend, clone L291H4) and anti-CD28-BV605 (BD Biosciences, clone CD28.2).

Antibodies for T cell analysis (second panel): using Beckman Coulter’s custom design service and its dry coating technology, custom tubes containing anti-CD183-FITC (clone G025H7), anti-CD197-PE (clone G043H7), anti-CD196-PE-Cy7 (clone B-R35), anti-PD1-APC (clone PD1.3), anti-CD45RA-Alexa Fluor 700 (clone 2H4LDH11LDB9 (2H4)), anti-CD4-PacificBlue (clone 13B8.2) and anti-HLA-DR-KromeOrange (clone Immu-357) were produced. Liquid antibodies were also used: anti-CD80-APC-Alexa Fluor 750 (BD Biosciences, clone L307.4) and anti-CD127-BV605 (BioLegend, clone A019D5).

Antibodies for Treg cell analysis: using Beckman Coulter’s custom design service and its dry coating technology, custom tubes containing anti-CD25-PE (clone B1.49.9), anti-CD39-PE-Cy5 (clone BA54), anti-PD1-PE-Cy7 (clone PD1.3), anti-CD278-APC (clone ISA-3), anti-CD45RA-Alexa Fluor 700 (clone 2H4LDH11LDB9 (2H4)), anti-CD4-PacificBlue (clone 13B8.2) and anti-CD8-KromeOrange (clone B9.11) were produced. Liquid antibodies were also used: anti-CCR4-PerCP-Cy5.5 (BioLegend, clone L291H4), anti-CD80-APC-Alexa Fluor 750 (BD Biosciences, clone L307.4) and anti-Tim3-BV605 (BioLegend, clone F38-282).

Antibodies for natural killer (NK) cell analysis: using Beckman Coulter’s custom design service and its dry coating technology, custom tubes containing anti-CD159a-PE (clone Z199), anti-PD1-PE-Cy5 (clone PD1.3) anti-CD335-PE-Cy7 (clone BAB281), anti-CD314-APC (clone ON72), anti-CD56-APC-Alexa Fluor 750 (clone N901), anti-CD16-PacBlue (clone 3G8) and anti-CD45-KromeOrange (clone J33) were produced. Liquid antibodies were also used: anti-Tim3-FITC (Miltenyi Biotec, clone REA635), anti-NKG2C-Alexa Fluor 700 (R&D Systems, clone 134591) and anti-CD3-BV605 (BioLegend, clone UCHT1).

Antibodies for myeloid cell analysis: multi-color flow cytometry was also performed using Beckman Coulter’s custom design service and its dry coating technology, and custom tubes containing anti-CD33-FITC (clone D3HL60.251), anti-CD39-PE (clone BA54), anti-CD3-Pe-Cy5 (clone UCTH1), anti-CD19-PE-Cy5 (clone J3-119), anti-CD20-PE-Cy5 (clone B9E9), anti-CD56-PE-Cy5 (clone N901), anti-PD-L1-APC (clone PDL1.3.1), anti-HLA-DR-APC-Alexa Fluor 750 (clone Immu-357), anti-CD15-PacificBlue (clone 80H5), anti-CD14-KromeOrange (clone RMO52) and a mortality marker DRAQ7 were produced. The following liquid antibody was added to the custom tubes: anti-CD11b-BV605 (BioLegend, clone ICRF44).

Staining protocol: 100 μl of total heparinized blood was added to each DURAClone tube containing liquid antibodies, vortexed immediately for 15 s and incubated for 15 min at room temperature in the dark. Two milliliters of red blood lysis solution (VersaLyse solution, A09777, Beckman Coulter) containing 50 μl of the fixative agent IOTest 3 Fixative Solution (A07800, Beckman Coulter) was added, inverted and incubated for 15 min in the dark. After centrifugation and washing with 3 ml of PBS 1×, cells were resuspended in 150 µl of PBS 1× before acquisition on a Canto II cytometer (BD Biosciences).

#### Lymphocyte function analysis

Using Beckman Coulter’s custom design service and its dry coating technology, custom tubes containing anti-IFN-γ-FITC (clone 45.15), anti-CD25-PE (clone B1.49.9), anti-CD4-PE-Cy5.5 (clone 13B8.2), anti-IL-4-PE-Cy7 (clone MP4-25D2), anti-Foxp3-Alexa Fluor 647 (clone 259D), anti-TNF-α-Alexa Fluor 700 (clone IPM2), anti-CD3-APC-Alexa Fluor 750 (clone UCHT1), anti-IL-17A-PacificBlue (clone BL168) and anti-CD8-KromeOrange (clone B9.11) were produced. Liquid antibody was also used: anti-IL-2-BV605 (BioLegend, clone MQ1-17H12).

Staining procedure: 100 μl of total heparinized blood was added to a DURActive 1 tube containing phorbol-myristate-acetate, ionomycin and brefledin A (C11101, Beckman Coulter) for 3 h at 37 °C in the dark. After activation, 25 μl of PerFix-NC R1 buffer (PerFix-NC Kit, B31168, Beckman Coulter) was added on vortex and incubated for 15 min at room temperature. Then, 2 ml of PBS 1× was added, and, after centrifugation, the pellet was resuspended in 25 μl of FBS (Dutscher), and 300 μl of PerFix-NC R2 buffer was added. A 325-μl aliquot was transferred to a DURAClone tube containing the liquid antibody, vortexed immediately for 15 s and incubated for 1 h at room temperature in the dark. PBS 1× (3 ml) was added to the tubes and incubated for 5 min at room temperature in the dark before centrifugation for 5 min at 500*g*. After supernatant removal, the cells were resuspended in 3 ml of 1× PerFix-NC R3 buffer before a further 5-min centrifugation at 500*g*. The pellet was dried and resuspended in 150 μl of 1× R3 buffer. Acquisition was done on a Canto II cytometer (BD Biosciences).

After the acquisition, validation of the compensations was performed for each .fcs file on Kaluza analysis software (Beckman Coulter), and then an unsupervised analysis was performed.

### DNA and RNA extraction

After the evaluation of the tumor cell content in FFPE tumor specimens by a pathologist, samples were macro-dissected to obtain at least 80% tumor cell content for nucleic acid extraction. DNA was isolated from tumor tissue using the Maxwell 16 FFPE Plus LEV DNA Purification Kit (Promega). DNA from whole blood (germline DNA) was isolated using the Maxwell 16 Blood DNA Purification Kit (Promega) following the manufacturer’s instructions. The quantity of extracted genomic DNA was assessed by a fluorometric method with a Qubit device. RNA was extracted using the Maxwell RSC RNA FFPE Kit (Promega) according to the manufacturer’s protocol. DNA and RNA quality and quantity were assessed by spectrophotometry with absorbance at 230 nm, 260 nm and 280 nm. Tumor purity was reported in a table for each exome and RNA-seq data where information was available (Supplementary File Table 7).

### Whole-exome capture and sequencing

Two hundred nanograms of genomic DNA was used for library preparation, using the Agilent SureSelectXT Reagent Kit. The totality of the enriched library was used in the hybridization and captured with SureSelect All Exon v5 or v6 (Agilent) baits. After hybridization, the captured libraries were purified according to the manufacturer’s recommendations and amplified by polymerase chain reaction (12 cycles). Normalized libraries were pooled, and DNA was sequenced on an Illumina NextSeq 500 device using 2× 111-base pair (bp) paired-end reads and multiplexed.

### RNA-seq

RNA depleted of ribosomal RNA was used for the library preparation with a NEBNext Ultra II RNA Directional Library Prep Kit for Illumina according to the manufacturer’s instructions (New England Biolabs). RNA-seq was performed on a NextSeq 500 device (Illumina). The libraries were sequenced with 76-bp paired-end reads.

### scRNA-seq

This analysis was performed on one patient with complete response. Fresh tumor tissue was collected on the day of surgery for this patient. Tumor was mechanically and enzymatically dissociated using a human tumor dissociation kit, according to the manufacturer’s instructions (130-095-929, Miltenyi Biotec). In brief, tumor was cut into small pieces and transferred into gentleMACS C tubes containing the enzyme mix. The dissociation was performed using the gentleMACS Octo Dissociator with heaters and with the human tumor dissociation 37C_h_TDK_1 program. Samples were homogenized before being applied to a MACS SmartStrainer 70 µM (130-110-916, Miltenyi Biotec) and placed in a 50-ml tube. Filters were washed with 20 ml of serum-free RPMI (L0500-500, Dutscher) and then centrifuged at 300*g* for 7 min. After complete aspiration of the supernatant, tumor cell suspensions were resuspended in RPMI and counted with trypan blue to remove dead cells. Cells were frozen in a solution of 50% FBS (Dutscher), 40% RPMI and 10% DMSO (P60-36720100, Dutscher) until further use. We also collected PBMCs for this patient at different timepoints: at cycle 1, at cycle 5 and at the time of surgery; PBMCs were isolated and frozen as described above. On the day of the single-cell experiment, the samples first underwent a specific preparation protocol. In brief, the samples were thawed following the 10x Genomics thawing protocol based on cascade dilutions. Dead cells were then removed with the Dead Cell Removal Kit according to the manufacturer’s instructions (130-090-101, Miltenyi Biotec). CD3 T cells were then isolated for each sample on a magnetic column after labelling with CD3 MicroBeads (130-050-101, Miltenyi Biotec). To better purify CD3 T cells, we labelled the samples with an anti-TCRαβ-PE (clone IP26A, Beckman Coulter) and an anti-TCRγδ (clone IMMU510, Beckman Coulter) to sort the positive cells for one of these two markers with an Aria Fusion Sorter. Finally, we resuspended the cells at 1,000 cells per microliter before proceeding with the cell encapsulation step according to the manufacturer’s instructions using the Chromium device (10x Genomics).

Library preparation was performed using library prep for 5′ mRNA and VDJ (10x Genomics). Sequencing was performed on an Illumina HiSeq 4000 device. Libraries were sequenced with 100-bp paired-end reads.

### Immunohistology procedure

Biopsies were collected before study entry (archival materials), at baseline or during treatment and were fixed after collection in paraformaldehyde and embedded in paraffin by the pathology laboratory. Four-micron slices were cut from FFPE tumor samples. The tissues embedded in paraffin were cut on a Leica rotary microtome (RM2145). For CD8 and PD-L1 staining, slides were deparaffinized and stained using a PT link (Agilent) and an Autostainer 48 (Agilent). In brief, slides were deparaffinized using a pH 9 buffer for 25 min at 95 °C. After cooling, slides were washed in wash buffer (Agilent) twice for 5 min. Peroxydase blocking was performed with peroxydase blocking reagent (SM801, Agilent). Then, anti-human CD8 (1:100, clone C8/144B, M7103, Agilent) or anti-human PD-L1 (1/200, clone QR1, C-P0001-01, Diagomics) was added for 30 min at room temperature. EnVision FLEX HRP polymers (SM802, Agilent) were added for 15 min at room temperature after two washing steps. DAB (SM803, Agilent) was then added to samples for 2 min. After two new washing steps, slides were finally incubated with hematoxylin (SM806, Agilent) for 20 min and permanently mounted using a Leica automated coverslipper. For the double staining procedure, after antigen retrieval as previously described, anti-decorin antibody (1:100, clone E2N2C, 85786, CST) was added for 30 min, and, after amplification steps as previously described, HRP Magenta (GV92511-2, Agilent) was added for 5 min. Antibody elution was next performed with stripping solution^54^. Anti-SATB2 antibody (1:100, clone EP281, BSB-3202, Diagomics) was then applied on tissue sections for 30 min. Amplification steps, counterstaining steps and mounting procedures were then performed as previously described. Once stained and permanently mounted, slides were digitalized with NanoZoomer HT2.0 (Hamamatsu) at ×20 magnification to generate a whole slide imaging (WSI) file in .ndpi format. Using QuPath software (version 2)^55^, CD8 and PD-L1 analysis was performed on three areas of the tumor core and three areas of the invasion front of the slide, and the annotations were validated by a pathologist. For CD8 analysis, the number of positive cells was counted in each area, and an average was calculated. For PD-L1 analysis, a cutoff for each subset was determined on diaminobenzidine intensity (brown staining) and automatically applied on every cell detected in annotated areas (that is, negative, 1+, 2+ and 3+). The PD-L1 H-score was then calculated with the following formula: H-score = [1 × (% cells 1+) + 2 × (% cells 2+) + 3 × (% cells 3+)]^56^. For double staining decorin/SATB2, quantification of decorin intensity was evaluated by an expert pathologist in a three categories (negative, low and high).

### Imaging mass cytometry

#### Antibodies and metal conjugation

Antibodies other than provided ready to use by Standard BioTools were conjugated to purified lanthanide metals and eluted in antibody stabilizer buffer (CANDOR Bioscience) using the Maxpar X8 Antibody Labeling Kit according to the manufacturer’s instructions (PRD002 Rev 14, Fluidigm, Standard BioTools). CD15 was labeled with ^89^Y metal using the procedure described in previous studies^57^. The antibodies used in this panel and the information concerning the clone, the supplier, the tag and the dilution are detailed in Supplementary File Table 8.

#### Antibody staining

After deparaffinization and antigen retrieval using Dako Target Retrieval Solution at pH 9 (S236784-2, Agilent) in a water bath (96 °C for 30 min), 3-µm tissue sections were encircled with a Dako Pen and incubated with SuperBlock (37515, Thermo Fisher Scientific) at room temperature for 45 min and then with FcR Blocking Reagent (130-092-575, Miltenyi) at room temperature for 1 h (1:100 in PBS/1% BSA buffer). After three washes (8 min each) in PBS/0.2% Triton X-100 (PBS-T), the PD-L1 antibody was diluted in PBS/1% BSA buffer and incubated at 4 °C overnight. The next day, the slides were washed three times (8 min each) in PBS-T, and secondary anti-rabbit antibody (3175002G, Standard BioTools) was diluted in at 1:500 in PBS/1% BSA buffer and incubated at room temperature for 2 h. After three washes (8 min each) in PBS-T, metal-tagged antibodies (list in Supplementary File Table 8) were diluted in PBS/1% BSA buffer. After incubation with the primary antibodies at 4 °C overnight, sections were washed in PBS-T three times (8 min each), and nuclei were stained with iridium (1:400 in PBS, Fluidigm, Standard BioTools), a DNA intercalator, for 30 min at room temperature. Sections were washed in PBS for 5 mi and then in distilled water for 5 min and then dried at room temperature for 30 min.

#### Data acquisition

Images were acquired with the Hyperion Imaging System (Fluidigm, Standard BioTools) according to the manufacturer’s instructions. After choosing the region of interest (ROI) in the section, the ROI was ablated with a UV laser at 200 Hz. Data were exported as .mcd files and visualized using Fluidigm MCD viewer 1.0.560.6. The minimum and maximum thresholds were adapted for each marker and for each tissue for optimal visualization. Gamma was set to 1.

#### Imaging mass cytometry data pre-processing and cell segmentation

The raw data (.mcd files) were processed with the Steinbock pipeline (version 0.15.0)^58^. In brief, automated pixel classification was performed using the machine-learning-based Mesmer algorithm (Steinbock toolkit) using the DeepCell library for cell segmentation^59^. In brief, we transformed the .mcd files into .tiff files on which a hot pixel filter of 50 was applied. To generate the segmentation mask, we used DNA as nuclear marker and PanCK, CD163, CD11b, CD45RO, CD31, CD66b, CD11c, CD4, CD68, CD45RA, CD8, CD45, GrB, Ki-67, Zeb-1, CasP3, Tim3 and HLA-DR as membrane/cytoplasmic markers with the default settings (pixel size at 1 µm, whole-cell segmentation, no normalization and mean aggregation). We then generated a second set of individual .tiff files to extract the mean signal intensity per marker for each cell with the computeFeatures function of the R package EBImage^60^ and compiled into .fcs single-cell files with the R package flowCore^61^.

### Cytokine measurement

Forty-five analytes were quantified in the plasma using Human XL Cytokine Magnetic 45-plex Luminex Assay (898855, R&D Systems) according to the manufacturer’s instructions: C-C motif chemokine ligand 2 (CCL2), CCL3, CCL4, CCL5, CCL11, CCL19, CCL20, CD40 ligand, fractalkine, C-X-C motif chemokine ligand 1 (CXCL1), CXCL2, CXCL10, epidermal growth factor (EGF), fibroblast growth factor (FGF), FMS-like tyrosine kinase 3 ligand (FLT3L), granulocyte colony-stimulating factor (G-CSF), granulocyte-macrophage colony-stimulating factor (GM-CSF), granzyme B, IFN-α, IFN-β, IFN-γ, IL-1α, IL-1β, IL-1RA, IL-2, IL-3, IL-4, IL-5, IL-6, IL-7, IL-8, IL-10, IL-12, IL-13, IL-15, IL-17A, IL-17E, IL-33, programmed death-ligand 1 (PDL1), platelet-derived growth factor (PDGF)-AA, PDGF-AB/BB, transforming growth factor (TGF)-α, tumor necrosis factor (TNF)-α, TNF-related apoptosis inducing ligand (TRAIL) and vascular endothelial growth factor (VEGF). The performance assay standard values for each analyte are detailed in Extended Data Table 2.

### ELISpot assay

Circulating tumor-specific T cell responses were assessed by IFN-γ ELISpot after short-term in vitro stimulation of PBMCs with a mixture of eight TERT-derived MHC class II-binding peptides (pool of HLA-DR and HLA-DP-restricted TERT peptides^62,63^) and a mixture of NY-ESO1 peptides at 5 μg ml^−1^ for 6 d as previously described^62,64^. All synthetic peptides (>90% purity) were purchased from JPT. A mixture of peptides referred to as CEF, derived from influenza virus, Epstein–Barr virus and cytomega-iovirus (Cellular Technology), was used to evaluate antiviral recall responses. In brief, the frozen PBMCs were thawed and cultured with tumor-derived peptides (5 µg ml^−1^). The culture was carried out in a 24-well plate (4 × 10^6^ cells per well) in RPMI 5% human serum. IL-7 (5 ng ml^−1^, 200-07, PeproTech) and IL-2 (20 UI ml^−1^, 202-IL-010, Novartis) were added on days 1 and 3, respectively. On day 7 of cell culture, the presence of antigen-specific T cells was measured by IFN-γ ELISpot assay according to the manufacturer’s instructions. In brief, lymphocytes from in vitro stimulation (10^5^ per well) were incubated for 18 h at 37 °C in an ELISpot plate pre-coated with anti-human IFN-γ monoclonal antibody, with or without peptide mixtures in X-VIVO 15 medium (BE04-418, Ozyme). Cells were cultured with medium alone and phorbol 12-myristate 13-acetate (1 ng ml^−1^, P8139, Sigma-Aldrich)/ionomycin (10 mmol L^−1^, I3909, Sigma-Aldrich) as negative and positive controls, respectively. The IFN-γ spots were revealed following the manufacturer’s instructions (Diaclone). The number of specific T cells expressed as ΔIFN-γ spots per 10^5^ cells was calculated after background value substraction (medium). Spot-forming cells were counted using the CTL Immunospot system (Cellular Technology). Responses were considered positive when the IFN-γ spots number was greater than 10 and greater than twice the background^65^.

The same experiment was conducted after synthesis of 14 neopeptides identified from patient exome analysis and expressed predominantly in the somatic exome (fold change (FC) in favor of tumor and median MT 50% inhibitory concentration (IC_50_) < 50). For 10 patients in the study, neopeptides were also synthesized (from one to eight depending on the patient) after being selected in the same manner as previously described. All neopeptides (>90% purity) were purchased from JPT. In brief, the experiment was performed in the same manner as with TERT and NYESO1 peptides, and the patients’ PBMCs were cultured in the presence or absence of the neopeptide pool at 10 µg ml^−1^.

### Whole-exome sequencing data analysis

Reads in the FASTQ format were aligned to the reference human genome GRCh37 using the Burrows–Wheeler aligner (BWA version 0.7.17). Local realignment was performed using the Genome Analysis Toolkit (GATK version 4.13.0). Duplicate reads were removed using Picard version 2.5. In case of matched tumor-normal samples, somatic single-nucleotide variants (SNVs) were identified using a validated pipeline that integrated mutation calls from three different mutation callers. SNVs were called with VarScan (version 2.4.3)^66^ and Mutect (version 1.1.7)^67^, and insertion/deletions (indels) were called with VarScan and Strelka (version 2.9.2)^68^. In case of tumor only, SNVs were called using Mutect2 (ref. ^69^), provided with GATK software.

TMB was calculated using the number of significant SNVs (with untranslated transcribed regions, synonyms, introns and intergenic SNVs filtered out) divided by the number of megabases covered at a defined level. To identify tumor-specific mutant peptides, pVAC-Seq (personalized Variant Antigens by Cancer Sequencing) was used (pVACtools version 1.5.4)^70^; pVAC-Seq is based on HLA typing obtained by HLAminer^71^. TITAN (version 1.23.1)^72^ and SuperFreq (version 1.4.2)^73^ were used, respectively, for matched tumor-normal samples and tumor-only samples to infer the number of copy number alteration (CNA) subclones, the number of large deletions and the loss of heterozygosity (LOH) > 15 Mb from whole-exome sequencing data. It was also used to estimate tumor ploidy. Copy number variant (CNV) signatures were inferred following the methodology of Macintyre et al.^74^. The copy number profile of each patient was reconstructed based on the weighted combination of seven signatures. The MSI score was computed using MSIsensor^75^. The HRD score was obtained through the scarHRD pipeline^76^.

### RNA-seq data analysis

Raw FASTQ data were pseudo-aligned, and gene counts as well as transcripts per kilobase million (TPM) were quantified using Kallisto software^77^. Kallisto transcript index used as reference was built from merged human cDNA and ncDNA files from the GRCh37 assembly Ensembl. Gene-level count and transcripts matrices were then created with the DESeq2 library. Low-count genes were pre-filtered by removing genes with too few reads. Genes differentially expressed were selected using the DESeq2 R package^78^. Gene set enrichment analysis (GSEA)^79^ was performed on resulting differential genes using hallmarks of cancer gene sets from the Broad Institute and the fgsea R package^80^.

Tumor microenvironment (TME)-associated transcriptomic elements were quantified using MCP-counter, ImmuCellAI and tools, following respective guidelines. The MCP-counter^23^ method allows the robust quantification of the absolute abundance of eight immune and two stromal cell populations. ImmuCellAI^24^ estimates the abundance of 24 immune cell types through a gene set signature‐based method. Finally, Kassandra uses a tree machine learning algorithm for the deconvolution of cell proportions in tissue on different hierarchical levels^25^.

The CMScaller^81^ R package was used for consensus molecular subtyping.

### Single-cell data analysis

Cell Ranger (version 3.1.0) was used for raw data pre-processing. Each library was aligned to an indexed hg19 genome using Cell Ranger count. Output from Cell Ranger was loaded into R and further analyzed using the Seurat pipeline (version 3.1.2). Dimensional reduction, clustering and differential expression analysis of scRNA-seq data were performed with the R package Seurat (version 3.2.0)^82^. Cells with expression of fewer than 200 or more than 2,500 genes and cells with more than 10% expression of mitochondrial genes were filtered out of the analysis. Gene expressions were normalized and log-transformed. To compare the four datasets, obtained from tumor at day 0, tumor at day 30, blood at surgery and TILs, they were integrated together using anchors, selected as features that appear most frequently across the datasets. This resulted in a total of 5,764 CD8 T cells (1,724 cells at day 0, 721 cells at day 30, 1,313 cells in blood at surgery and 2,006 cells in TILs). We determined the 20 nearest neighbors of each cell, constructed the shared nearest neighbor (SNN) graph and optimized the modularity function to perform the clustering algorithm. The resulting nine clusters were visualized in a two-dimensional t-distributed stochastic neighbor embedding (tSNE)^83^ representation. Genes differentially expressed between clusters were selected with the Wilcoxon rank-sum test.

To determine differentiation trajectories for cells from all clusters, we used the R package Monocle 2 (ref. ^84^). Monocle uses an algorithm to learn the changes in gene expression sequences that each cell must go through as part of a dynamic biological process (differentiation or regeneration, for example). More precisely, tree-like trajectories are learned using the DDRTree method, sequenced in pseudotime and finally visualized in two-dimensional space.

### TCR sequence analysis

TCR sequencing was used to count clonotypes detected in more than two cells per sample. A cell’s clonotype was defined as the combined alpha and beta chain CDR3 nucleotide sequences for that cell. As it was not possible to deduce beta and alpha chain pairing for partitions with multiple beta chains, these partitions were treated as a single clone. To assess clonal enrichment, the proportion of cells having the same clone was compared between sample types for each clone. To determine whether clonal expansion of CD8 T cells may be driven by common antigen(s), we used the GLIP^27^ algorithm (version 1.0) to assess TCR CDR3 similarity and putative shared specificity across the four samples.

### Statistical analysis

The efficacy population included all participants who met the eligibility criteria and who received at least one complete or two incomplete treatment cycles. All enrolled patients who initiated the study treatment were included in the safety analysis.

PFS was defined as the time from the date of metastasis diagnosis to the first recorded evidence of disease progression by RECIST, clinical evaluation or death. OS was calculated as the time from the date of metastasis diagnosis to the date of death. The median follow-up was calculated using the reverse Kaplan–Meier method, and survival endpoints are described using the Kaplan–Meier method. Data for patients who were alive and event free were censored at the date of last follow-up. Survival probabilities were estimated using the Kaplan–Meier method, and survival curves were compared using the log-rank test. The 95% CIs for fractional survival at any particular time were added to survival curves. Univariate Cox proportional hazard models were performed to estimate the HR and 95% CI to test the association of the different variables with OS and PFS.

Quantitative variables are described as median and range. Qualitative variables are described using number, percentage and the 95% CI (binomial law). Estimated parameters are reported with two-sided 95% CIs. *P* ≤ 0.05 was considered statistically significant. Statistical analyses were performed using R software version 4.0.3 (http://www.R-project.org/), and graphs were drawn using GraphPad Prism version 9.0.2.

Calculations were performed using high-performance computing resources from DNUM CCUB (Centre de Calcul de l’Université de Bourgogne).

### Reporting summary

Further information on research design is available in the Nature Portfolio Reporting Summary linked to this article.

## Online content

Any methods, additional references, Nature Portfolio reporting summaries, source data, extended data, supplementary information, acknowledgements, peer review information; details of author contributions and competing interests; and statements of data and code availability are available at 10.1038/s41591-023-02497-z.

## Supplementary information

Supplementary Information Supplementary Figs. 1–8, Supplementary Tables 1–8, Protocol Clinical Study and CONSORT 2010 Checklist.

Reporting Summary

## Source data

Source Data Fig. 1 Statistical source data.

Source Data Fig. 2 Statistical source data.

Source Data Fig. 3 Statistical source data.

Source Data Fig. 4 Statistical source data.

Source Data Fig. 5 Statistical source data.

Source Data Fig. 6 Statistical source data.

Source Data Extended Data Table 1 Statistical source data.

Source Data Extended Data Table 2 Statistical source data.

Source Data Extended Data Fig. 2 Statistical source data.

Source Data Extended Data Fig. 3 Statistical source data.

Source Data Extended Data Fig. 4 Statistical source data.

Source Data Extended Data Fig. 5 Statistical source data.

Source Data Extended Data Fig. 6 Statistical source data.

Source Data Extended Data Fig. 7 Statistical source data.

Source Data Extended Data Fig. 8 Statistical source data.

## Figures and Tables

**Extended Data Table 1:**
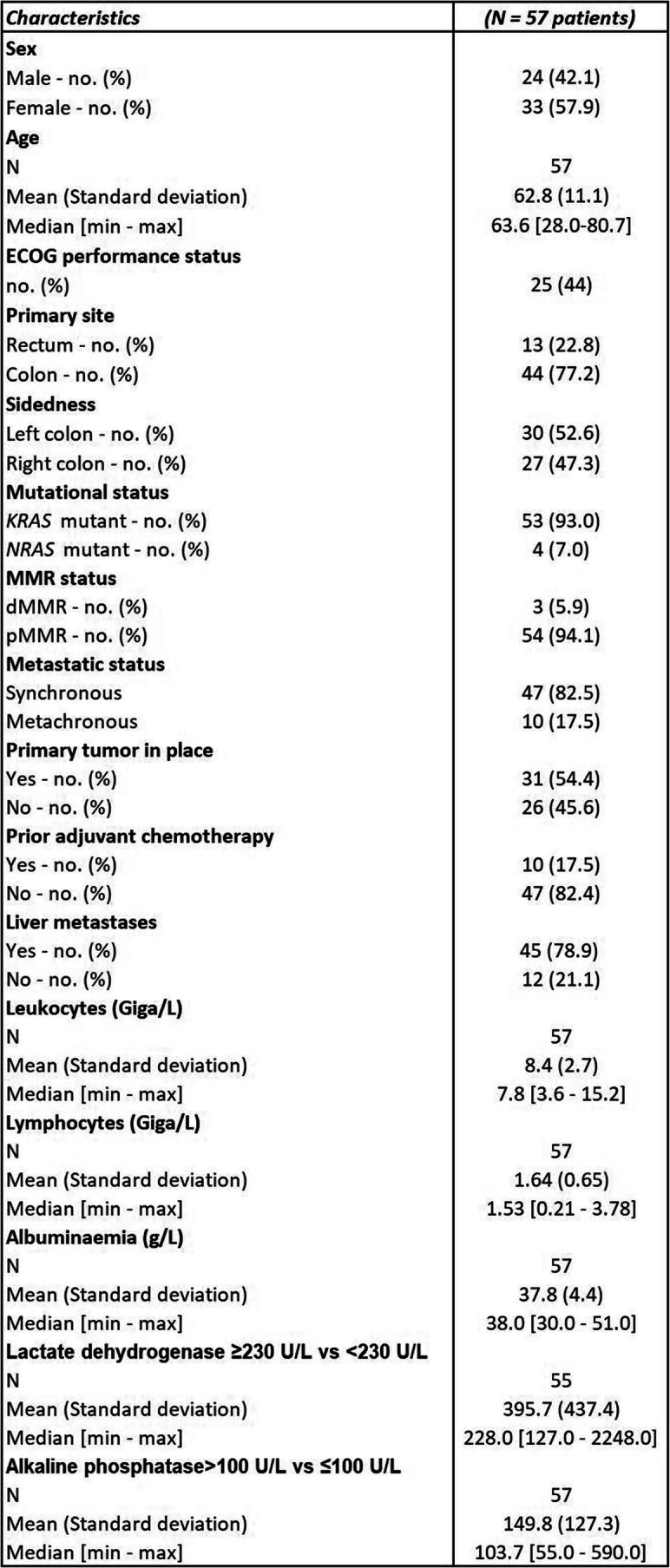
Patient characteristics

**Extended Data Table 2:**
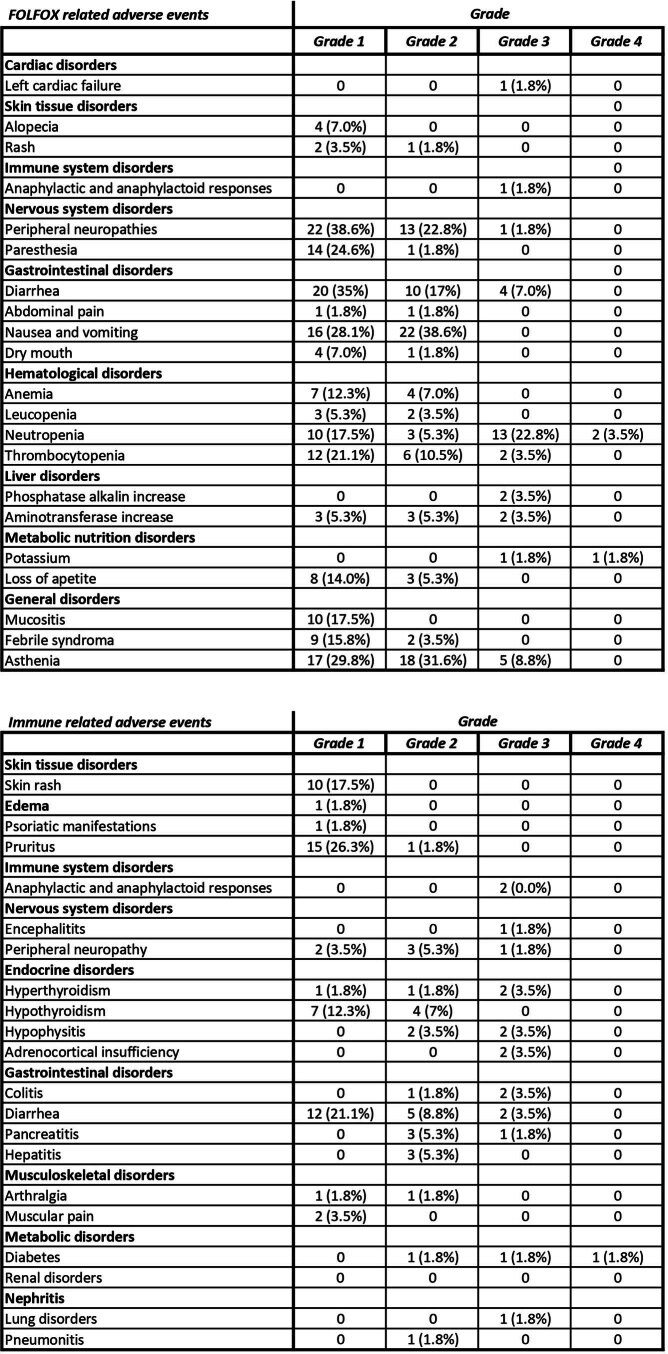
Toxicity attributed to chemotherapy and immunotherapy by investigators

## Data Availability

The RNA-seq and single-cell data generated in this study have been deposited in the Gene Expression Omnibus database under accession number GSE235920. Any request for raw or analyzed data will be reviewed by the study team, and a response can be expected within 14 d. The data generated in this study are subject to patient confidentiality. Any shared data will be de-identified. Requests should be made to the corresponding authors (fghiringhelli@cgfl.fr or mthibaudin@cgfl.fr). Source data are provided with this paper.
