# Supplementary material for: First-line durvalumab and tremelimumab with chemotherapy in RAS-mutated metastatic colorectal cancer: a phase 1b/2 trial
Source: Nat Med. 2023 Aug 10;29(8):2087–98. doi: 10.1038/s41591-023-02497-z (PMC10427431; doi:10.1038/s41591-023-02497-z)
Supplement: Supplementary file 1 — Supplementary Figs. 1–8, Supplementary Tables 1–8, Protocol Clinical Study and CONSORT 2010 Checklist. [file 41591_2023_2497_MOESM1_ESM.pdf]

nature medicine

Article https://doi.org/10.1038/s41591-023-02497-z

First-line durvalumab and tremelimumab with chemotherapy in RAS-mutated metastatic colorectal cancer: a phase 1b/2 trial

In the format provided by the authors and unedited

Supplementary File Figure 1

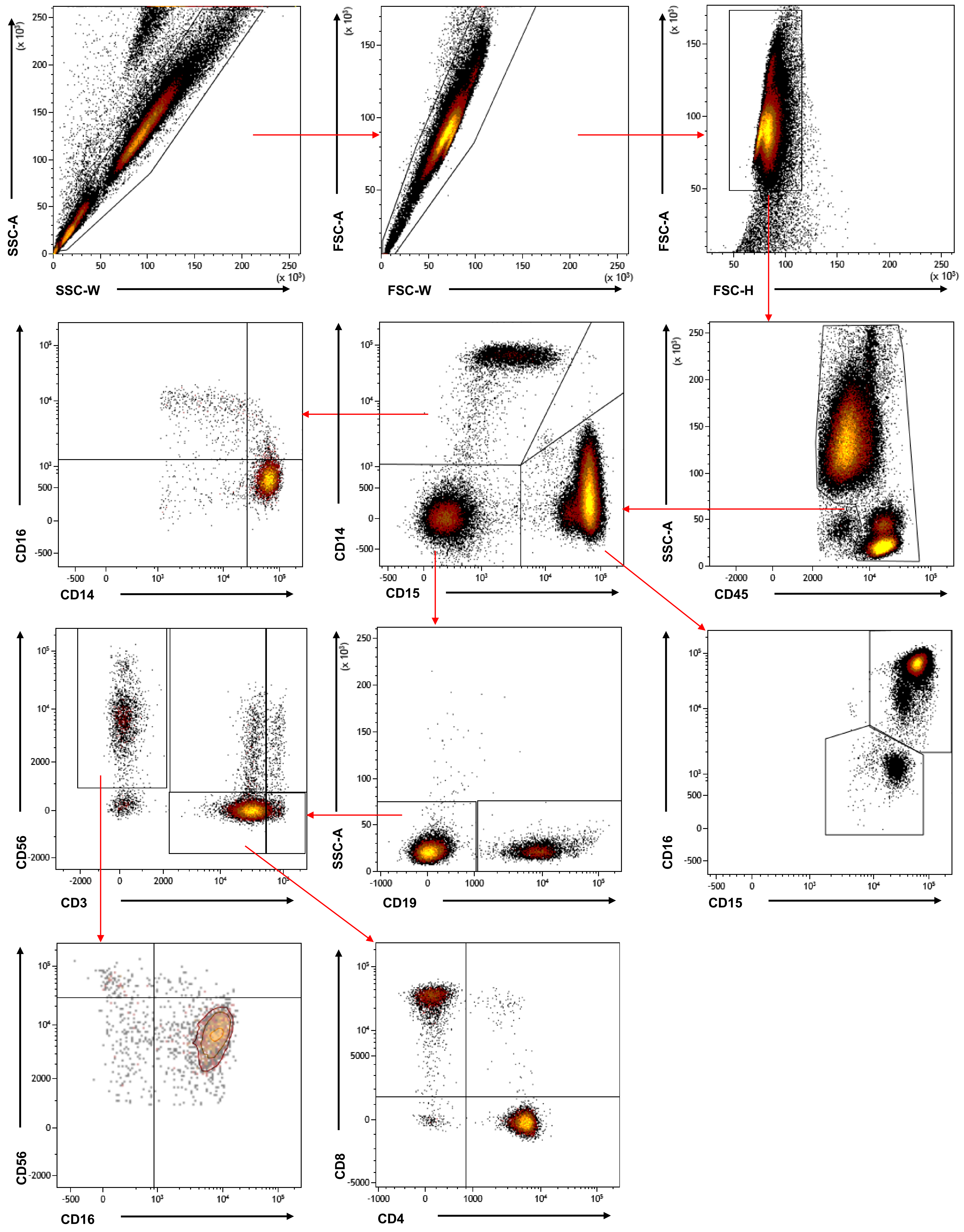

NFS Panel - Whole blood of mCRC patients was stained with anti-CD16, anti-CD56, anti-CD25, anti-HLA-DR, anti-CD14, anti-CD4, anti-CD8, anti-CD3, anti-CD15, anti-CD45, anti-CD127, anti-CCR7 and anti-CD45RA antibodies and analyzed by flow cytometry (Canto II, Becton Dickinson). Patients were monitored for blood levels at baseline and during their treatment at C2, C5 and at progression.

After removal of doublets and debris, CD45+ leukocytes are selected. Neutrophils (CD45+ CD15+ CD14-) and monocytes (CD45+ CD14+ CD15-) are then identified. On each neutrophil and monocyte populations, the expression of CD16 are performed and allowed the identification of subpopulations: Basophils (CD15+ CD16-), Neutrophils (CD15+ CD16+), Classical monocytes (CD14+ CD16-), intermediate monocytes (CD14+ CD16+) and non-classical monocytes (CD14low CD16+). On CD14- CD15- population, B cells are then identified (CD45+ CD14- CD15- CD19+) and on CD19- population the identification of T and NK populations is available: T cells (CD3+ CD56-), NK (CD3- CD56+), NKT (CD3+ CD56+). On CD3+ T cells, CD4+ and CD8+ T cells are then identified and on NK population the status of the cells are studied with the expression of CD16.

Supplementary File Figure 2

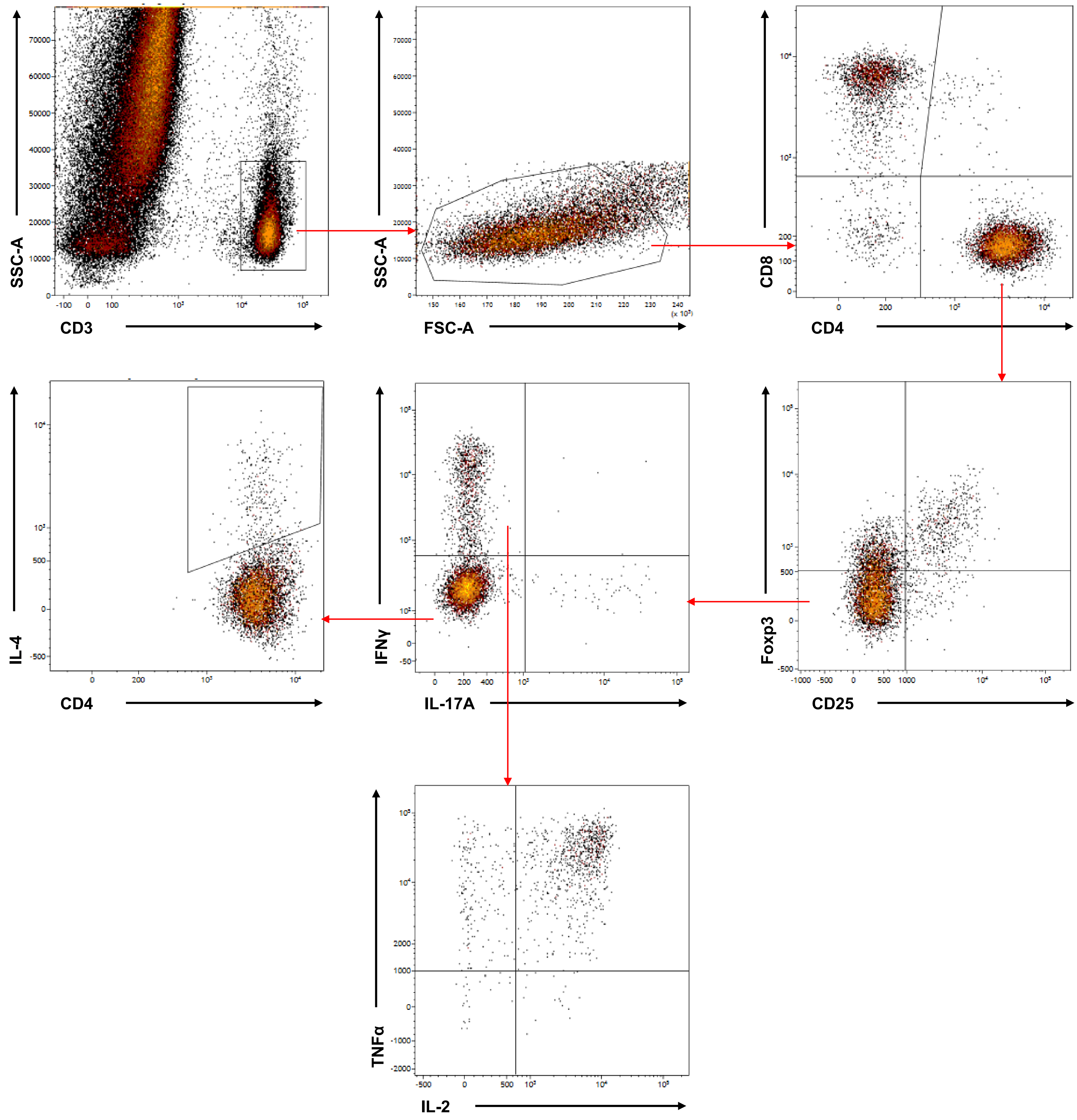

Cytokine Panel - Whole blood of mCRC patients was stimulated with PMa and Ionomycin in the presence of Brefedin-A for 3 hours at 37°C. Blood was then stained with anti-CD3, anti-CD4, anti-CD8, anti-CD25, anti-Foxp3, anti-IFNg, anti-IL-17A, anti-IL-4, anti-IL-2 and anti-TNFa antibodies and analyzed by flow cytometry (Canto II, Becton Dickinson). Patients were monitored for blood levels at baseline and during their treatment at C2, C5 and at progression.

After CD3 identification, the doublets and debris are removed and CD4+ and CD8+ T cells are identified. On these two populations, regulatory populations are selected by the expression of CD25+ and Foxp3+. We then analysed within the CD4conv T cells (CD4+ CD25- Foxp3-) each of the following subpopulations: Th1 (CD4+ Foxp3- IFNγ+ IL-17A-), Th17 (CD4+ Foxp3- IFNγ- IL-17A+), Th1/17 (CD4+ Foxp3- IFNγ+ IL-17A+) and Th2 (CD4+ Foxp3- IFNγ- IL-17A- IL-4+) cells. We performed the same analysis within CD8conv T cells (CD8+ CD25- Foxp3-) : Tc1, Tc17, Tc1/17 and Tc2 cells. For each of these populations, we studied the expression of the cytokines TNFα and IL-2 as shown for Th1 in this gating strategy.

Supplementary File Figure 3

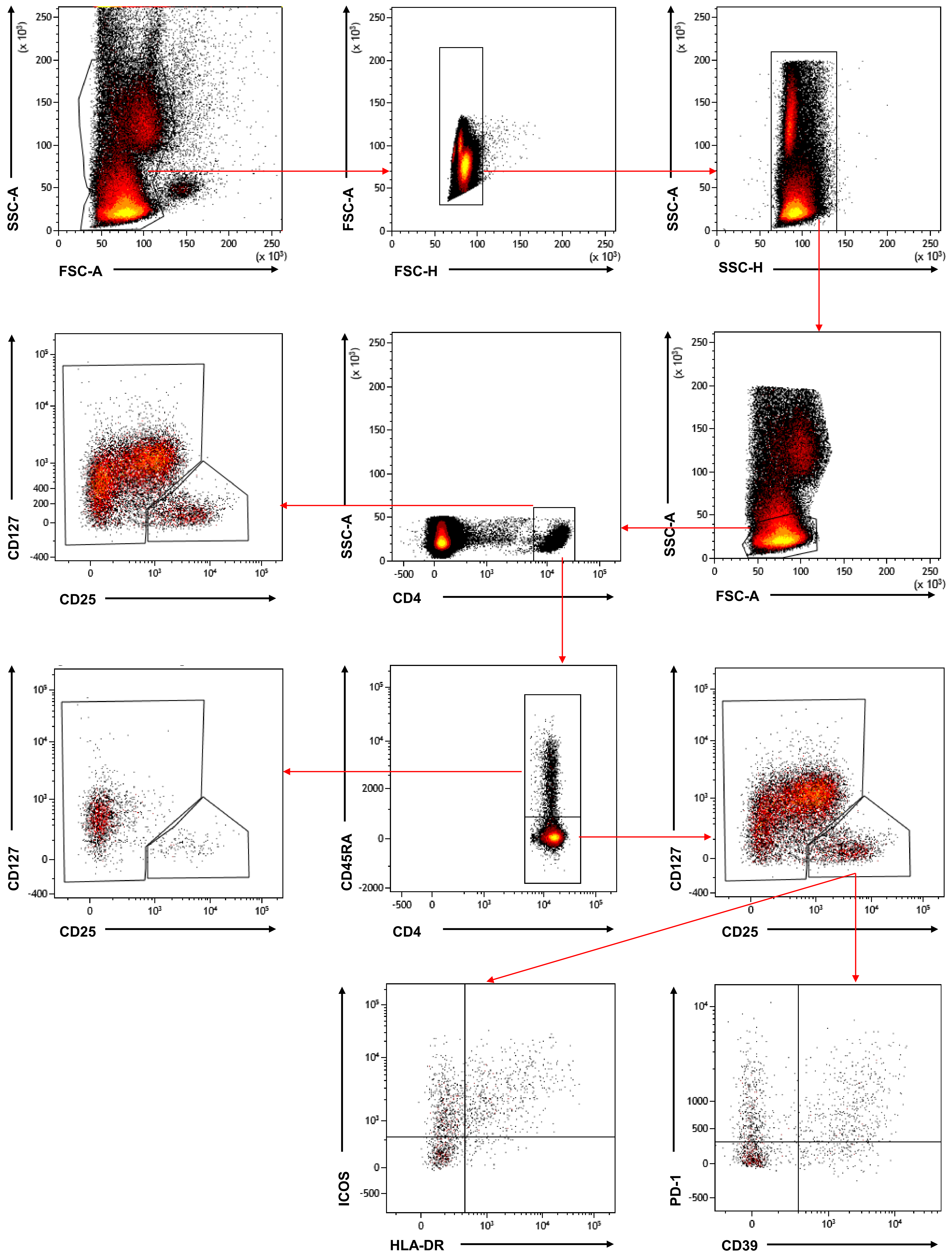

Treg Panel - Whole blood of mCRC patients was stained with anti-CD4, anti-CD25, anti-CD127, anti-CD45RA, anti-HLA-DR, anti-CD39, anti-PD-1, anti-ICOS and anti-CD80 antibodies and analyzed by flow cytometry (Canto II, Becton Dickinson). Patients were monitored for blood levels at baseline and during their treatment at C2, C5 and at progression.

After removal of doublets and debris, CD4+ T cells are selected. Naïve and memory CD4 T cells are identified with the marker CD45RA: naïve CD45RA+ CD4 T cells and memory CD45RA- CD4 T cells. Thanks to the expression of CD25 and CD127, regulatory T cells are identified (CD4+ CD25+ CD127low). Natural and induced regulatory T cells are also identified in the same way from naïve and memory CD4 T cells respectively. On each of its Treg populations, the expression and co-expression of markers was performed: HLA-DR and ICOS or CD39 and PD-1.

Supplementary File Figure 4

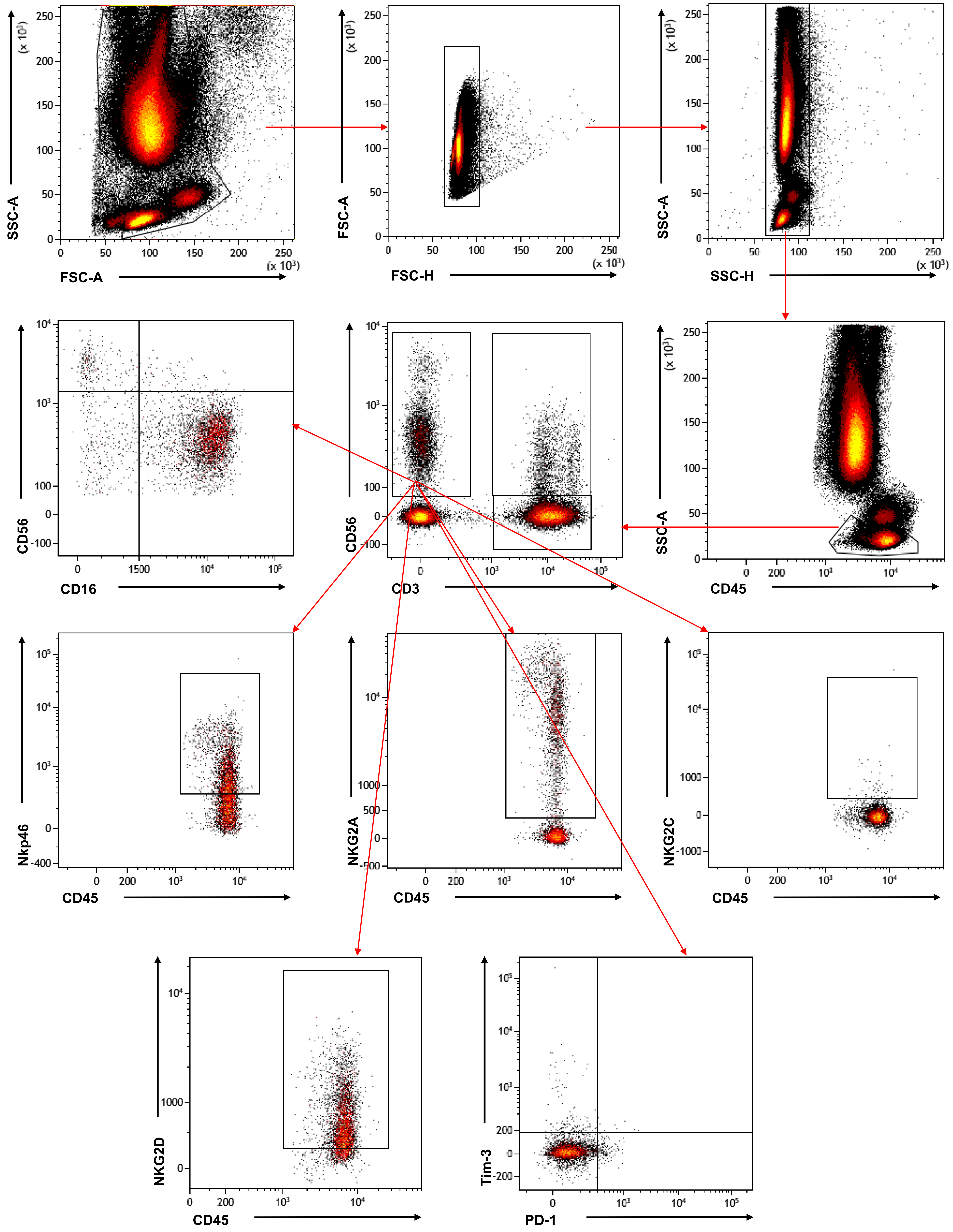

NK Panel - Whole blood of mCRC patients was stained with anti-CD45, anti-CD56, anti-CD3, anti-CD16, anti-Nkp46, anti-NKG2A, anti-NKG2C, anti-NKG2D, anti-PD-1 and anti-Tim3 antibodies and analyzed by flow cytometry (Canto II, Becton Dickinson). Patients were monitored for blood levels at baseline and during their treatment at C2, C5 and at progression.

After removal of doublets and debris, CD45+ lymphocytes are selected by CD45 and SSC-A. After, the identification of T and NK populations is available: T cells (CD3+ CD56-), NK (CD3- CD56+), NKT (CD3+ CD56+). Then, on NK population, the expression of Nkp46, NKG2A, NKG2C and NKG2C and the co-expression of PD-1 and Tim3 was performed.

Supplementary File Figure 5

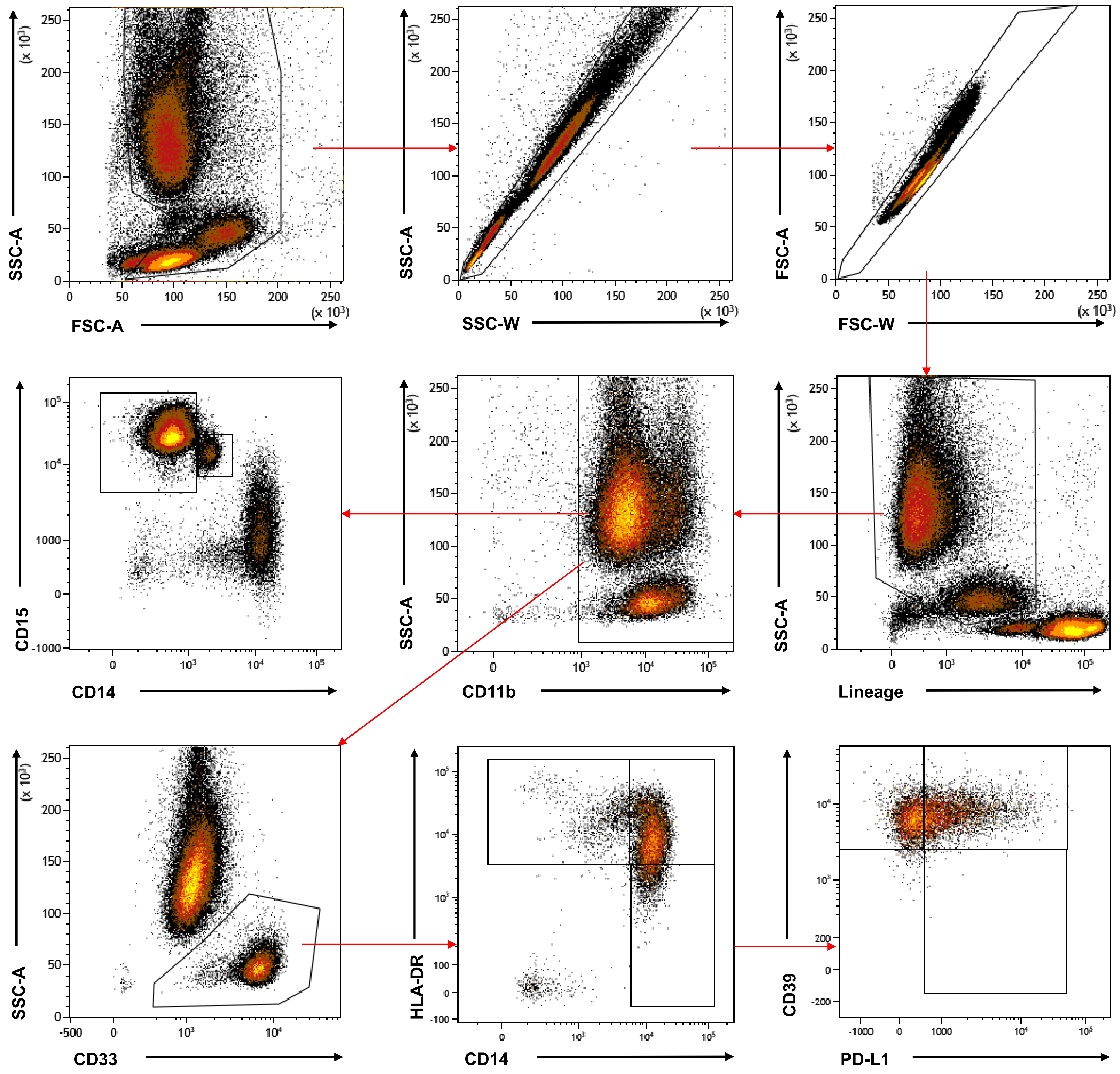

Myeloid Panel - Whole blood of mCRC patients was stained with a viability dye (Draq7) and anti-CD11b, anti-CD15, anti-CD14, anti-CD33, a lineage (composed of an anti-CD3, anti-CD19, anti-CD20, anti-CD56), anti-HLA-DR, anti-CD39 and anti-PD-L1 antibodies and analyzed by flow cytometry (Canto II, Becton Dickinson). Patients were monitored for blood levels at baseline and during their treatment at C2, C5 and at progression.

After removal of doublets and debris, cells are selected with the SSC-A and the absence of expression of lineage. Then, myeloid cells are identified with the marker CD11b and neutrophils (CD45+ CD15+ CD14-) are identified. On CD11b+ population, monocytes are selected with the expression of CD33 and SSC-A. On monocytes, the expression of CD14 and HLA-DR allowed the identification of mMDSC (CD14+ HLA-DRlow), classical monocytes (CD14+ HLA-DR+) and inflammatory monocytes (CD14low HLA-DR+). For each of identified populations, we studied the expression or co-expression of CD39 and PD-L1 as shown for mMDSC in this gating strategy.

Supplementary File Figure 6

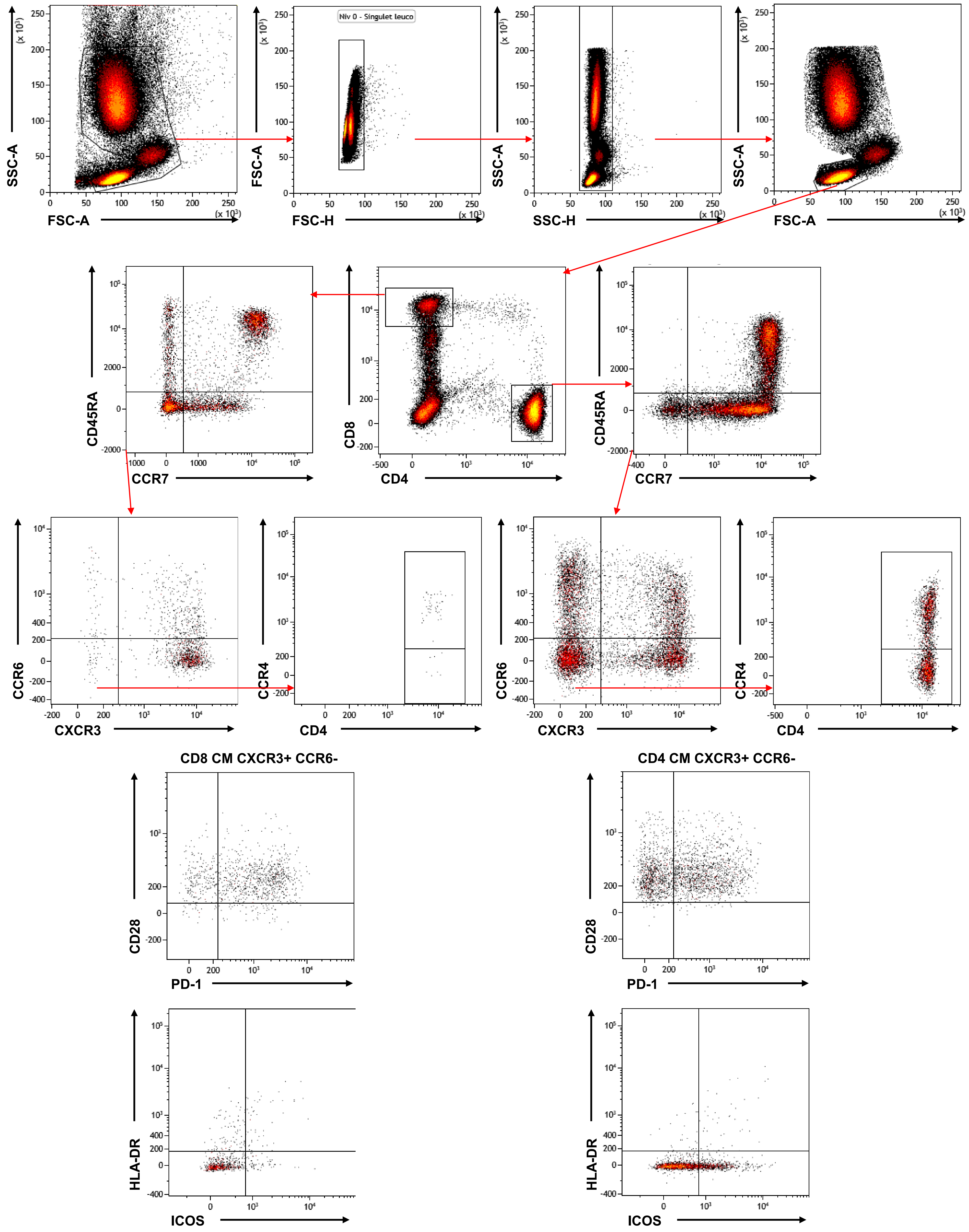

T CD4/CD8 subtypes 1 &2 Panel - Whole blood of mCRC patients was stained for panel 1 with anti-CD4, anti-CD8, anti-CXCR3, anti-CCR6, anti-CCR4, anti-CD45RA, anti-CCR7, anti-HLA-DR, anti-ICOS and anti-Tim3 antibodies and was stained for panel 2 with anti-CD4, anti-CD8, anti-CXCR3, anti-CCR6, anti-CCR4, anti-CD45RA, anti-CCR7, anti-HLA-DR, anti-PD-1 and anti-CD28 antibodies. Then, these two panels were analyzed by flow cytometry (Canto II, Becton Dickinson). Patients were monitored for blood levels at baseline and during their treatment at C2, C5 and at progression.

For the two panels, after removal of doublets and debris, lymphocytes are selected with the size FSC-A and the structure SSC-A. Then, CD4+ and CD8+ T cells are identified and the level of differentiation of each of these two populations is studied using the markers C5RA and CCR7 to identify naïve (CD45RA+ CCR7+), effector memory (CD45RA-, CCR7-), central memory (CD45RA- CCR7+) and EMRA (CD45RA+ CCR7-) cells. On each CD4 and CD8 subpopulation, the expression of CXCR3, CCR6 and CCR4 markers is analyzed and allowed the identification of Th1/Tc1 (CXCR3+ CCR6-), Th17/Tc17 (CXCR3- CCR6+), Th17-Th1/Tc17-Tc1 (CXCR3+ CCR6+) and Th2/Tc2 (CXCR3- CCR6- CCR4+) cells. For each of identified populations, we studied the expression or co-expression of CD28 and PD-1 (panel 1) or of HLA-DR and ICOS (panel 2) as shown for CXCR3+ CCR6- central memory CD8 and CD4 T cells in this gating strategy.

Supplementary File Figure 7

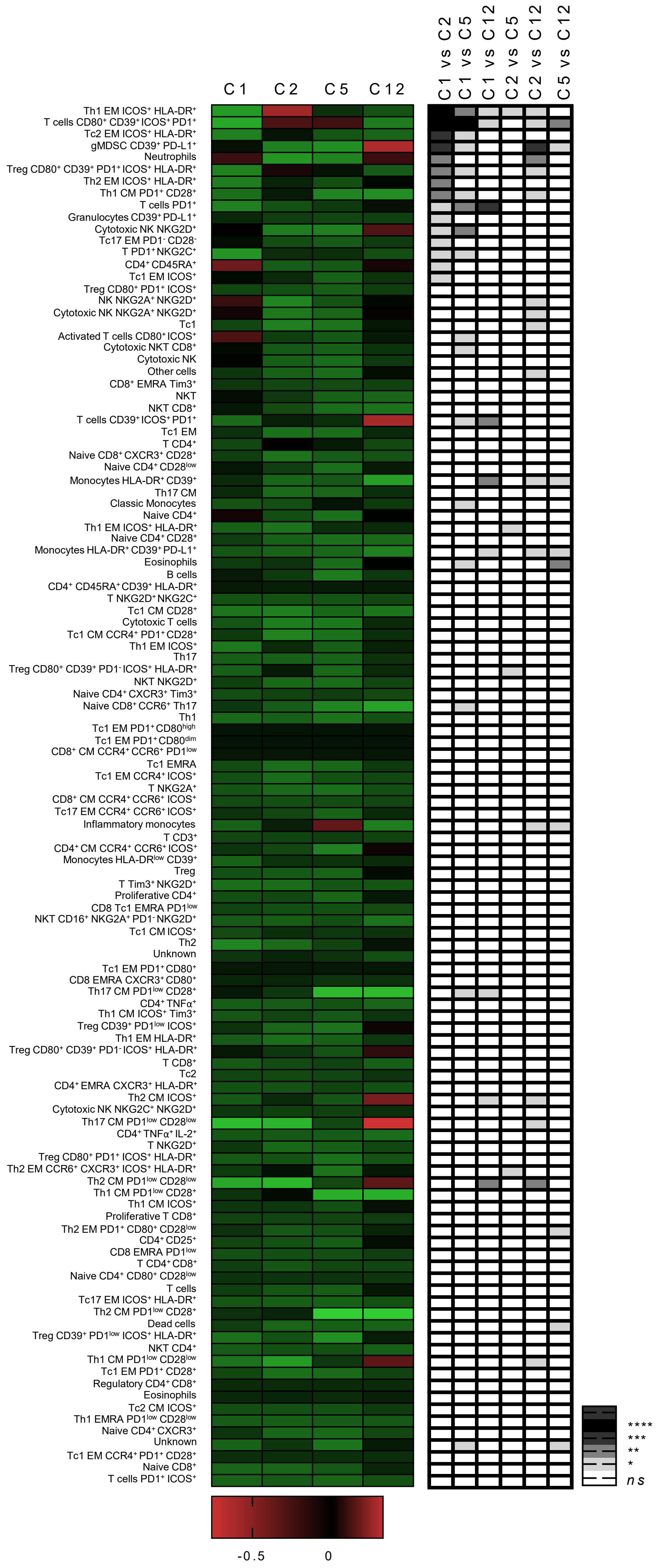

Heat map showing all leukocyte populations identified after analysis of patients' blood at C1 (baseline), C2, C5 and C12 by multiparametric flow cytometry (7 panels) and unsupervised analysis.

Supplementary File Figure 8

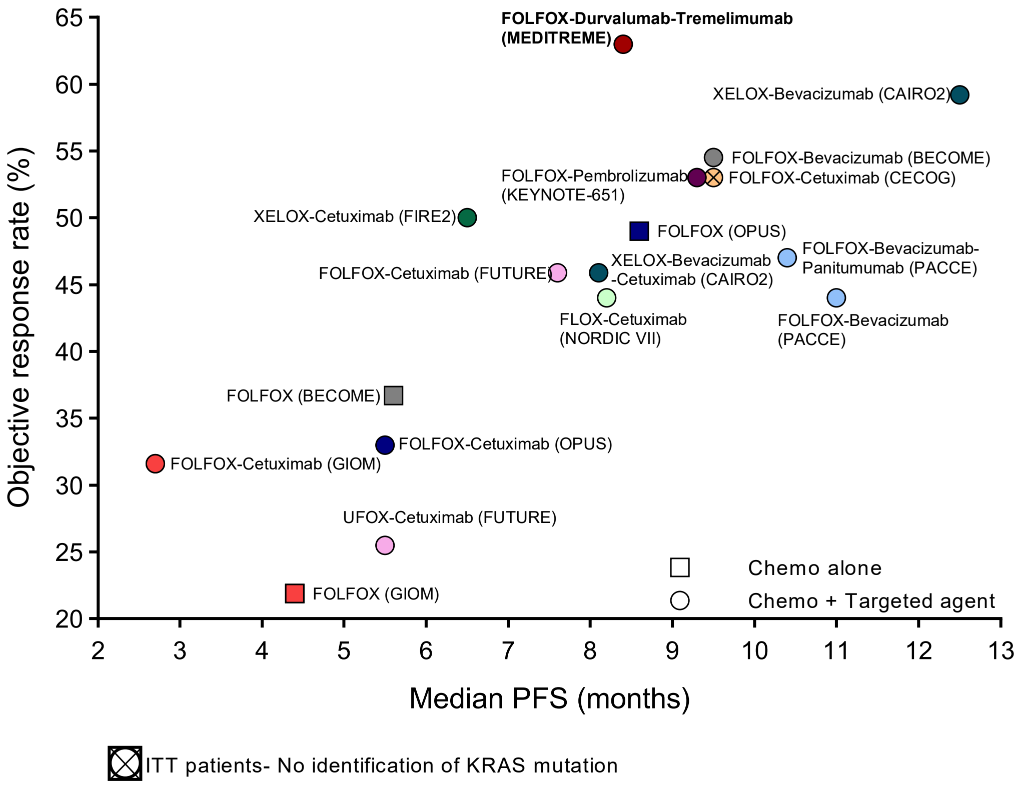

Graph showing the objective response rate versus median PFS of the main studies that compared bi-chemotherapy to bi-chemotherapy plus targeted therapies in colorectal cancer. Data are for patients with mutated RAS tumor, except for the CECOG study (circle/square with a cross).

Investigational Drug	Durvalumab (MEDI4736) Tremelimumab FOLFOX
Study Number	*ESR-15-11282*
Version Number	Version 4.1
Date	23 Jan 2020

Phase Ib/II trial evaluating the safety, tolerability and immunological activity of Durvalumab (MEDI4736) (Anti-PD-L1) Plus Tremelimumab (Anti-CTLA-4) combined with FOLFOX in patients with metastatic colorectal cancer

Sponsor: Centre Georges François Leclerc

EudraCT : 2016-005006-19

Investigator coordinator: Pr François GHIRINGHELLI

DESIGN OF THE STUDY

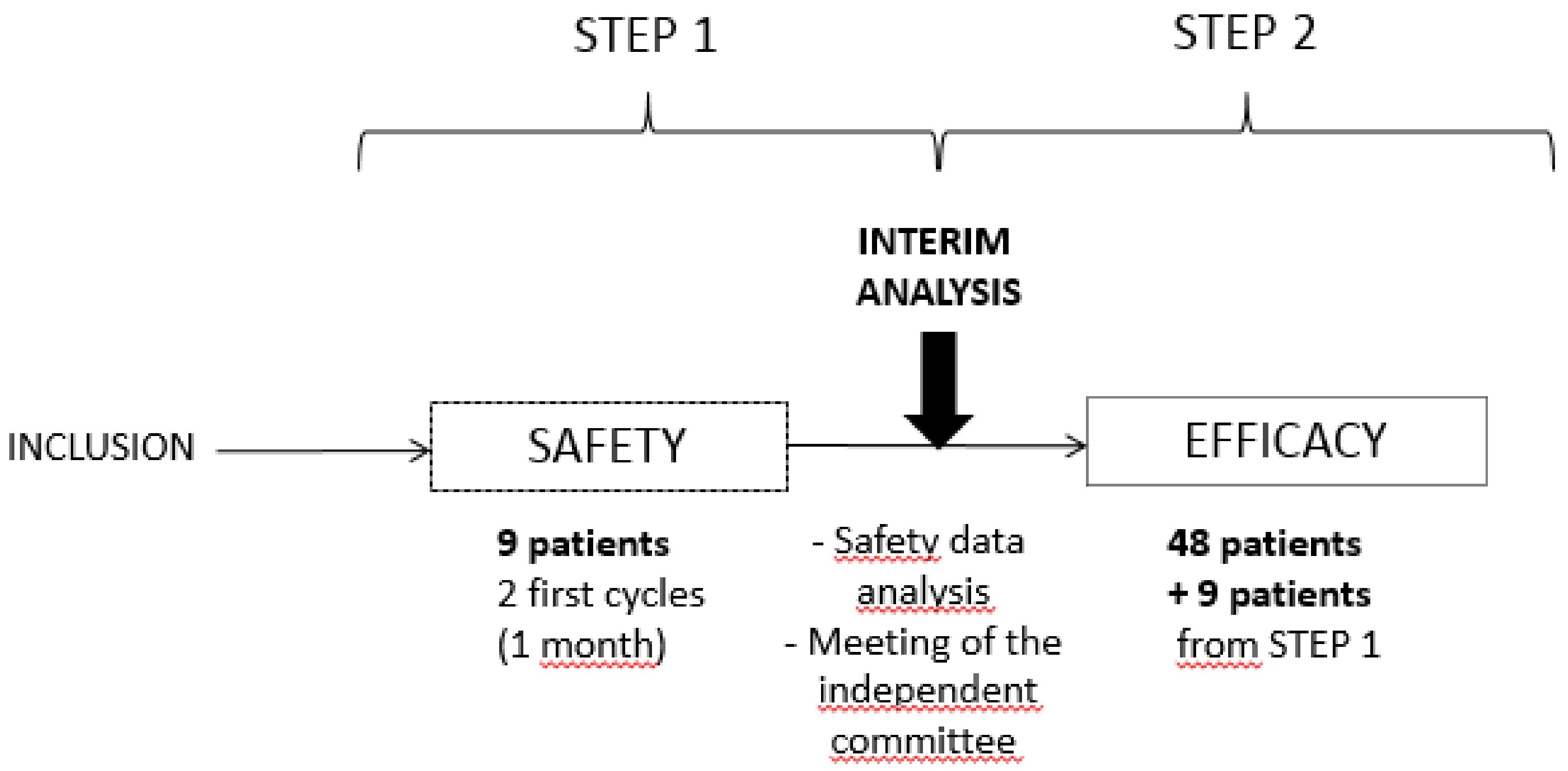

PROTOCOL SYNOPSIS

Clinical Protocol MEDI-TREME-COLON

Study Title: Phase Ib/II trial evaluating the safety, tolerability and immunological activity of Durvalumab (MEDI4736) (Anti-PD-L1) Plus Tremelimumab (Anti-CTLA-4) combined with FOLFOX in patients with metastatic colorectal cancer

Synopsis Number: version 2.0 from 21/06/2018

Clinical Phase: Ib/II

Study Duration: 5 years

Investigational Product(s) and Reference Therapy:

- **Durvalumab (MEDI4736)** will be supplied in glass vials containing 500 mg of liquid solution at a concentration of 50 mg/mL for intravenous (IV) administration.
- **Tremelimumab** will be supplied as a sterile solution for IV infusion, filled in 20 mL or 1,25 mL clear glass vials with a rubber stopper and aluminum seal. Each vial contains 20 mg/mL (with a nominal fill of 20 mL, accounting to 400 mg/vial or fill of 1,25 mL accounting to 25 mg/vial) of tremelimumab, in an isotonic solution at pH 5.5.
- **FOLFOX** will be composed by Oxaliplatin 100 mg; Folinic acid 200 mg and 5-FU 5 mg for intravenous (IV) administration.

Research Hypothesis

Combination of chemotherapy (FOLFOX) with immunotherapy association (anti-PD-L1 + anti-CTLA-4) would act synergistically in patients with colo-rectal cancer.

Phase Ib primary objective (STEP 1):

To determine **the safety** of the combination of Durvalumab (Anti-PD-L1) + Tremelimumab (Anti-CTLA-4) + FOLFOX

Phase II primary objective (STEP 2):

To determine **the efficacy** of the combination of Durvalumab (Anti-PD-L1) + Tremelimumab (Anti-CTLA-4) + FOLFOX in terms of progression free survival (PFS) in patients with colorectal MSS disease.

Phase II secondary Objective:

To determine efficacy of the combination of Durvalumab (Anti-PD-L1) + Tremelimumab (Anti-CTLA-4) + FOLFOX in terms of response to treatment and overall survival in patients with colorectal MSS disease.

- To determine efficacy of the combination of Durvalumab (Anti-PD-L1) + Tremelimumab (Anti-CTLA-4) + FOLFOX in terms of PFS, response to treatement and overall survival in patients with colorectal MSI disease.

ExploratoryStudies:

- To evaluate quality of life at each cycle,
- To determine genetically characterized for MSI status,
- To determine NRAS, KRAS and Braf status,
- To study the immune cells infiltration into the tumor,
- To analyze PD-1,PD-L1, CTLA-4 expression with Ventana assay system
- To determine double labelling of Th1, Th2, Th17, Follicular helper T cells and exhausted T cells,
- To perform identification of tumor-specific mutations,
- To determine candidate of neoantigens and also prediction for proteasomal processing and HLA class I binding will be assess,
- Analyze immune response before and after treatment start,
- To assess local immune response before and after therapy,
- To study evaluation of lymphocyte reactivity to tumor antigens,
- To analyze cytokine production by T cells.

Study Design:
Multicentric, non randomized, prospective, phase Ib/II study.

Step 1: Phase Ib with the aim to confirmed the safety of the association of Durvalumab (q2w 750mg) + Tremelimumab (q4w 75mg) + FOLFOX

Step 2: Phase II with the aim to assess efficacy of the association of Durvalumab (q2w 750mg) + Tremelimumab (q4w 75mg) + FOLFOX

Number of Centers: 9 sites

**Planned participating centers**			
N°	**Investigators**	**Hospital**	**Town**
1	Pr François GHIRINGHELLI	CGFL	Dijon
2	Pr Christophe BORG	CHRU	Besançon
3	Pr Jaafar BENNOUNA	ICO	Nantes
4	Pr Julien TAIEB	Hôpital Européen Georges Pompidou	Paris
5	Dr Romain COHEN	Hôpital Saint Antoine	Paris
6	Dr Antoine DROUILLARD	CHU Dijon	Dijon
7	Dr Marianne FONCK	Institut BERGONIE	Bordeaux
8	Dr Benoist CHIBAUDEL	Institut Hospitalier Franco-Britannique	Levallois-Perret

9	Dr Jérôme MARTIN-BABAU	Hôpital privé des côtes d'Armor	Plérin

Number of Subjects:

Step 1: 9 subjects
Step 2: 48 subjects
Step 1 + Step 2 =**57 subjects**

Study Population:

Patient with metastatic Colo-rectal cancer

Inclusion Criteria:

1. Written informed consent and any locally-required authorization obtained from the subject prior to performing any protocol-related procedures, including screening evaluations,
2. Male or female age > 18 years at time of study entry,
3. Performance status of 0 or 1 according to the Eastern Cooperative Oncology Group (ECOG) and World Health Organisation (WHO),
4. Histologically confirmed diagnoses of colorectal cancer with positive mutated KRas or NRas
5. Patients with metastatic disease
6. First line metastatic disease **Or** First line after localized disease treated by local curative treatment, with or without adjuvant chemotherapy by FOLFOX; Reccurence after the last dose of adjuvant chemotherapy should be ≥ 6 months. Previous perioperative chemotherapy for resecable metastasis is not permitted
7. Life expectancy of > 12 weeks,
8. Adequate normal organ and marrow function as defined below:
   - Haemoglobin > 9.0 g/dL
   - Absolute neutrophil count (ANC) > 1.5 x 10^9/L (>1500 per mm^3)
   - Platelet count > 100 x 10^9/L (≥100,000 per mm^3)
   - Serum bilirubin ≤ 1.5 x institutional upper limit of normal (ULN).
   - AST (SGOT)/ALT (SGPT) ≤ 2.5 x institutional upper limit of normal unless liver metastases are present, in which case it must be ≤ 5x ULN
   - PAL ≤ 5 x institutional upper limit of normal unless liver metastases are present, in which case it must be ≤ 20x ULN
   - Albumin > 30g/L
   - Creatinine < 1.5 X institutional upper limit of normal (ULN)
   - Serum creatinine CL>40 mL/min by the Cockcroft-Gault formula (Cockcroft and Gault 1976) or by 24-hour urine collection for determination of creatinine clearance:

	Males	Females
Creatinine CL (mL/min)	$\frac{\text{Weight (kg) x (140 – Age).}}{\text{72 x serum creatinine (mg/dL)}}$	$\frac{\text{Weight (kg) x (140 – Age)}}{\text{72 x serum creatinine (mg/dL)}}$ x 0.85

9. Tumour evaluation (CT scan) in the previous 4 weeks with presence of at least one measurable lesion according to RECIST 1.1 criteria,
10. At least 4 weeks since the last chemotherapy, immunotherapy or other drug therapy and / or radiotherapy,
11. Recovery to grade ≤ 1 from any adverse event (AE) derived from previous treatment according to the criteria of the National Cancer Institute Common Terminology Criteria for Adverse Events (NCI-CTCAE) version 4.0,
12. For principal study: willingness to provide consent for use of archived tissue with sufficient material available for analysis. For ancillary study: Metastasis should be accessible to performed biopsy
13. Female subjects must either be of non-reproductive potential (ie, post-menopausal by history: ≥60 years old and no menses for ≥1 year without an alternative medical cause; OR history of

hysterectomy, OR history of bilateral tubal ligation, OR history of bilateral oophorectomy) or must have a negative serum pregnancy test upon study entry,

14. Patients must be affiliated to a social security system,

15. Subject is willing and able to comply with the protocol for the duration of the study including undergoing treatment and scheduled visits and examinations including follow up.

Exclusion Criteria:

1. Involvement in the planning and/or conduct of the study (applies to both AstraZeneca staff and/or staff at the study site). Previous enrolment in the present study,

2. Participation in another clinical study with an investigational product during the last 4 weeks,

3. Any previous treatment with a PD-1 or PD-L1 /CTLA-4 inhibitor, including durvalumab or tremelimumab,

4. History of another malignancy within the 5 previous years with low potential risk for recurrence other than :
   - *Adequately treated non-melanoma skin cancer or lentigo maligna without evidence of disease*
   - *Adequately treated carcinoma in situ without evidence of disease eg, cervical cancer in situ*

5. Receipt of the last dose of anti-cancer therapy (chemotherapy, immunotherapy, endocrine therapy, targeted therapy, biologic therapy, tumor embolization, monoclonal antibodies, other investigational agent) 28 days prior to the first dose of study drug (14 days prior to the first dose of study drug for subjects who have received prior TKIs (e.g., erlotinib, gefitinib and crizotinib) and within 6 weeks for nitrosourea or mitomycin C). (*If sufficient wash-out time has not occurred due to the schedule or PK properties of an agent, a longer wash-out period may be required.),*

6. Mean QT interval corrected for heart rate (QTc) ≥470 ms calculated from 3 electrocardiograms (ECGs) using Fridericia's Correction,

7. Current or prior use of immunosuppressive medication within 28 days before the first dose of durvalumab, with the exceptions of intranasal and inhaled corticosteroids or systemic corticosteroids at physiological doses, which are not to exceed 10 mg/day of prednisone, or an equivalent corticosteroid,

8. Any history of hypersensitivity to durvalumab or tremelimumab, FOLFOX or their excipients,

9. Any unresolved toxicity (CTCAE grade >1) from previous anti-cancer therapy. *Subjects with irreversible toxicity that is not reasonably expected to be exacerbated by the investigational product may be included (e.g., hearing loss, peripherally neuropathy)*

10. Any prior Grade ≥3 immune-related adverse event (irAE) while receiving any previous immunotherapy agent, or any unresolved irAE >Grade 1,

11. Active or prior documented autoimmune disease within the past 2 years NOTE: Subjects with vitiligo, Grave's disease, or psoriasis not requiring systemic treatment (within the past 2 years) are not excluded,

12. Active or prior documented inflammatory bowel disease (e.g., Crohn's disease, ulcerative colitis),

13. History of primary immunodeficiency,

14. History of organ transplant that requires use of immunosuppressive,
15. History of allogeneic organ transplant,
16. Uncontrolled intercurrent illness including, but not limited to, ongoing or active infection; Clinically significant cardiovascular disease including: myocardial infarction within 6 months,, symptomatic congestive heart failure, uncontrolled hypertension, unstable angina pectoris, cardiac arrhythmia; history of Mobitz II second degree or third degree heart block without a permanent pacemaker in place, hypotension; rest limb claudication or ischemia within 6 months; active peptic ulcer disease or gastritis, active bleeding diatheses including any subject known to have evidence of acute or chronic hepatitis B, hepatitis C or human immunodeficiency virus (HIV), or psychiatric illness/social situations that would limit compliance with study requirements or compromise the ability of the subject to give written informed consent,
17. Sever concurrent disease, or co-morbidity that in the judgment of the investigator, would make the patient inappropriate for enrolment,
18. Ongoing treatment with CYP3A4 substrates or regularly taking of grapefruit juice,
19. Known history of active tuberculosis,
20. History of leptomeningeal carcinomatosis,
21. Brain metastases or spinal cord compression,
22. Receipt of live attenuated vaccination within 30 days prior to study entry or within 30 days of receiving durvalumab,
23. Female subjects who are pregnant, breast-feeding or male or female patients of reproductive potential who are not employing an effective method of birth control,
24. Any condition that, in the opinion of the investigator, would interfere with evaluation of study treatment or interpretation of patient safety or study results,
25. Symptomatic or uncontrolled brain metastases requiring concurrent treatment, inclusive of but not limited to surgery, radiation and/or corticosteroids,
26. Subjects with uncontrolled seizures,
27. Subjects under guardianship, curatorship or judicial protection
28. Known allergy or hypersensitivity to IP or any excipient
29. Patients with tumors that invade major vessels, as shown unequivocally by imaging studies
30. Patients with central lung metastases (i.e within 2 cm from the hilum) that are cavitary as shown unequivocally by imaging studies
31. Patients with any prior history of bleeding related to the current colorectal cancer
32. Patients with a history of gross hemoptysis (bright red blood of ½ teaspoon or more per episode of coughing) ≤ 3 months prior to enrollment
33. Patients with a recurrence delay smaller than 6 months after the adjuvant chemotherapy.
34. Patients with resecable disease.

**Investigational Product(s), Dose, and Mode of Administration:**<br><br>**- Durvalumab**, 750 mg Q2W (equivalent to 20 mg/kg Q4W) for 12 months in patients > 30 kg<br><br>**- Tremelimumab** 75 mg Q4W (equivalent to 1 mg/kg Q4W) for up to 4 doses/cycles in patients >30 kg<br>Weight-based dosing should be utilized for patients <30 kg durvalumab 20 mg/kg Q4 and tremelimumab 1 mg/kg Q4<br><br>**- FOLFOX:** Oxaliplatin 85 mg/m² (IV, 2h); Folinic acid 400 mg/m² or L-Folinic Acid (200mg/m²) (IV, 2h); 5-FU bolus 400 mg/m² (IV, 10 min) ; 5-FU continu 2400 mg/m² (IV, 46h)
**Study Assessments and Criteria for Evaluation:**<br><br>**STEP 1: Safety Assessments**<br>**The primary endpoint is toxicity** following NCI-CTC v4.0. Toxicity will be assess on the first 9 patients within 2 cycles (30 days) following the first administration of Durvalumab + tremelimumab + FOLFOX and are defined as an adverse event (AE) that may be linked to one of the study drugs.<br><br>Safety data will also be completed during all the STEP 2 of the study<br><br>**STEP 2: Efficacy Assessments**<br><br>1) RECIST 1.1 criteria evaluated in TDM imaging every 3 months,<br>2) irRC (Immune Related Response Criteria) methods,<br>3) Progression free survival (PFS, RECIST 1.1 criteria),<br>4) Overall survival (OS).
**Statistical Methods and Data Analysis:**<br><br>STEP 1: Safety assessment<br>Safety analyses will be performed on the first 9 patients. Toxicities during the first 2 cycles will be considered. If the same grade 3-4 adverse event is reported for more than 3 patients over 9, the trial could prematurely stop after reunion of the independent committee.<br><br>Data on safety will be completed during the second part of the study.<br><br>STEP 2: Efficacy assessment<br>Efficacy analyses will be performed in modified intent-to-treat population (ITT) population ie, all patients following the major inclusion criteria and with a 3 months evaluation. Analyses will be repeated in the intent to treat principle i.e including all enrolled patients whatever eligibility criteria and treatment received patients and in the per protocol population (patients who had received all the planned doses).<br><br>The primary objective will be evaluate at 3 months according to the Simon's design.

Sample Size Determination:

Step 1: Nine patients will be included in the **safety** analyses. If the same grade 3-4 adverse event are reported for more than 1 patient over 3, the trial could prematurely stop after reunion of the independent committee. Patients from the safety population will be included in the efficacy set.

Step 2: The **efficacy** will be determined using a Simon 2 steps phase II design.

The hypotheses are the following:

The primary objective will be evaluated among MSS patients

A PFS of 3 months is not considered of interest. A PFS of 6 months is expected. This is equivalent to assume that a PFS of 50% at 3 months is insufficient and a PFS of 70.7% is expected. With α=10%, β=10% (90% power), 10% of non-evaluable patients, 52 patients with MSS disease are needed including patients of the safety population (9 patients).

The prevalence of MSS disease is around 90-95%, thus 5 additional patients will be included

Overall the study will include **57 patients**.

Schedule of study assessments: Screening and Treatment Period (12 months: maximum of 26 doses, last infusion week 50)

1) **Figure 1a: Schedule of assessment of principal study – Association phase**

   Figure 1b: Schedule of assessment of principal study – Maintenance phase

2) **Figure 2: Schedule of assessment of explorative study**

Figure 1a: Schedule of assessment for principal study – Association phase

Assessments to be performed at the times stipulated in the table and as clinically required in the management of the subject	Scree-ning (J-28 à J-1)	FOLFOX + Durvalumab (MEDI4736) + Tremelimumab							
		V1	V2	V3	V4	V5	V6	V7	V8
	Month	M1		M2		M3		M4	
Written informed consent/assignment of subject dentification number	X								
Demography and history of tobacco and alcohol use	X								
Previous treatments for colo-rectal cancer	X								
Verification of eligibility criteria	X								
Inclusion of the subject	X								
Medical and surgical history	X								
Hepatitis B and C; HIV	X								
Urine hCG or serum βhCGb [a]	X	As clinically indicated							
Weight	X	X	X	X	X	X	X		X
Physical examination[b]	X	X	X	X	X	X	X		X
Vital signs[c] (pre- during and post-infusion vital signs assessments)	X	X	X	X	X	X	X	X	X
Adverse event/serious adverse event assessment[j]		X	X	X	X	X	X	X	X
Concomitant medications	X	X	X	X	X	X	X	X	X
WHO ECOG performance status	X	X	X	X	X	X	X		X
Liver enzyme panel[f]	X	X	X	X	X	X	X	X	X
Serum Chemistry (complete clin chem. panel)	X	X	X	X	X	X	X	X	X
Thyroid function tests (TSH and fT3 and fT4)[f]	X	X			X				X
Hematology[f]	X	X	X	X	X	X	X	X	X
Urinalysis[g]	X				X				X
Coagulation parameters[h]	X	As clinically indicated							
Electrocardiogram[d]	X	As clinically indicated							
Biopsie[l]	X								
CT scan[i]	X						X		
QLQ-C30 and QLQ-CR29	X		X				X		
Durvalumab (750 mg)		X	X	X	X	X	X	X	X
Tremelimumab (75mg)		X		X		X		X	
FOLFOX administration		X	X	X	X	X	X		

Figure 1b: Schedule of assessment for principal study – Maintenance phase

Assessments to be performed at the times stipulated in the table and as clinically required in the management of the subject	Durvalumab (MEDI4736) alone																							
	V9	V10	V11	V12	V13	V14	V15	V16	V17	V18	V19	V20	V21	V22	V23	V24	V25	V26	V27	V28	V29	V30	V31	V32
	M5		M6		M7		M8		M9		M10		M11		M12		M13		M14		M15		M16	
Written informed consent/assignment of subject dentification number																								
Demography and history of tobacco and alcohol use																								
Previous treatments for colo-rectal cancer																								
Verification of eligibility criteria																								
Inclusion of the subject																								
Medical and surgical history																								
Hepatitis B and C; HIV																								
Urine hCG or serum βhCGb [~~b~~ a]	As clinically indicated																							
Weight		X		X		X		X		X		X		X		X		X		X		X		X
Physical examination[~~c~~ b]		X		X		X		X		X		X		X		X		X		X		X		X
Vital signs[~~d~~ c] (pre- during and post-infusion vital signs assessments)	X	X	X	X	X	X	X	X	X	X	X	X	X	X	X	X	X	X	X	X	X	X	X	X
Adverse event/serious adverse event assessment[j]	X	X	X	X	X	X	X	X	X	X	X	X	X	X	X	X	X	X	X	X	X	X	X	X
Concomitant medications	X	X	X	X	X	X	X	X	X	X	X	X	X	X	X	X	X	X	X	X	X	X	X	X
WHO ECOG performance status		X		X		X		X		X		X		X		X		X		X		X		X
Liver enzyme panel[~~f~~]	X	X	X	X	X	X	X	X	X	X	X	X	X	X	X	X	X	X	X	X	X	X	X	X
Serum Chemistry (complete clin chem. panel)				X				X				X				X				X				X
Thyroid function tests (TSH and fT3 and fT4)[~~g~~ f]				X				X				X				X				X				X
Hematology[~~f~~]				X				X				X				X				X				X
Urinalysis[~~h~~ g]				X				X				X				X				X				X
Coagulation parameters[~~i~~ h]	As clinically indicated																							
Electrocardiogram[~~e~~ d]	As clinically indicated and required at EOT																							
Biopsie[l]																								
CT scan[i]				X						X						X						X		
QLQ-C30 and QLQ-CR29				X						X						X						X		
Durvalumab (750 mg)	X	X	X	X	X	X	X	X	X	X	X	X	X	X	X	X	X	X	X	X	X	X	X	X
Tremelimumab (75mg)																								
FOLFOX administration																								

Assessments to be performed at the times stipulated in the table and as clinically required in the management of the subject	Durvalumab (MEDI4736) alone																End of Treatement
	V33	V34	V35	V36	V37	V38	V39	V40	V41	V42	V43	V44	V45	V46	V47	V48	
	M17		M18		M19		M20		M21		M22		M23		M24		
number																	
Demography and history of tobacco and alcohol use																	
Previous treatments for colo-rectal cancer																	
Verification of eligibility criteria																	
Inclusion of the subject																	
Medical and surgical history																	
Hepatitis B and C; HIV																	
Urine hCG or serum βhCGb [~~b~~ a]	As clinically indicated																X
Weight		X		X		X		X		X		X		X		X	X
Physical examination[~~c~~ b]		X		X		X		X		X		X		X		X	X
Vital signs[~~d~~ c]	X	X	X	X	X	X	X	X	X	X	X	X	X	X	X	X	X
Adverse event/serious adverse event assessment[j]	X	X	X	X	X	X	X	X	X	X	X	X	X	X	X	X	X
Concomitant medications	X	X	X	X	X	X	X	X	X	X	X	X	X	X	X	X	X
WHO ECOG performance status		X		X		X		X		X		X		X		X	X
Liver enzyme panel[~~f~~]	X	X	X	X	X	X	X	X	X	X	X	X	X	X	X	X	X
Serum Chemistry (complete clin chem. panel)				X				X				X				X	X
Thyroid function tests (TSH and fT3 and fT4)[~~g~~ f]				X				X				X				X	X
Hematology[~~f~~]				X				X				X				X	X
Urinalysis[~~h~~ g]				X				X				X				X	X
Coagulation parameters[~~i~~ h]	As clinically indicated																
Electrocardiogram[~~e~~ d]	As clinically indicated																X
Biopsie[l]																	
CT scan[i]				X						X						X	
QLQ-C30 and QLQ-CR29				X						X						X	
Durvalumab (750 mg)	X	X	X	X	X	X	X	X	X	X	X	X	X	X	X	X	
Tremelimumab (75mg)																	
FOLFOX administration																	

Figure 2: Schedule of assessment of explorative study

Assessments to be performed at the times stipulated in the table and as clinically required in the management of the subject	Screening J28 à J-1	Durvalumab + Tremelimumab + FOLFOX												Durvalumab (MEDI4736) alone												End of treatment
		V1	V2	V3	V4	V5	V6	V7	V8	V9	V10	V11	V12	V13	V14	V15	V16	V17	V18	V19	V20	VX	VY	V47	V48	
Day		D 1	D 1	D 1	D 1	D 1	D 1	D 1	D 1	D 1	D 1	D 1	D 1	D 1	D 1	D 1	D 1	D 1	D 1	D 1	D 1	D 1	D 1	D 1	D 1	
Month		M1		M2		M3		M4		M5		M6		M7		M8		M9		M10		MX		M16		EOT
Biopsie of tumor tissue sample (PCR, immunohistochmistry, wall exome sequencing, neo antigens determinsation)[m]						X							X													
Blood samples for genetic profile of the tumor and circulating DNA[n]		X																								
Blood samples for:<br>- immune response analyze (flow cytometry; MDSC; circulation PD-L1)[o]<br>- evaluation of lymphocyte reactivity to tumor antigens[o]<br>- for circulating soluble factors (to assess cytokines, chemokines, growth factors and antibodies against tumour and self antigens in circulation)[o]		X	X			X							X[k]													x[k]

* Biological samples have to be collected at Visite 12 if no progressive disease (PD) is diagnosed before Visite 12. If progressive disease (PD) is diagnosed before Visite 12, End of study visit should be performed and samples should be collected. Samples should be picked only once at Visite 12 or at the EOT visit.

[a] Pre-menopausal female subjects of childbearing potential only

[b] Full physical examination at baseline; targeted physical examination at other time points

[c] Subjects will have their blood pressure and pulse measured before, during and after the infusion at the following times (based on a 60-minute infusion):

- At the beginning of the infusion (at 0 minutes)
- At 30 minutes during the infusion (±5 minutes)
- At the end of the infusion (at 60 minutes ±5 minutes)
- In the 1 hour observation period post-infusion: 30 and 60 minutes after the infusion (ie, 90 and 120 minutes from the start of the infusion) (±5 minutes) – for the first infusion only and then for subsequent infusions as clinically indicated

  If the infusion takes longer than 60 minutes then blood pressure and pulse measurements should be collected every 30 minutes (±5 minutes) and as described above or more frequently if clinically indicated.

d ECG during screening. Thereafter as clinically indicated. Baseline and abnormal ECG at any time in triplicate others single

e If screening laboratory assessments are performed within 3 days prior to Day 1 they do not need to be repeated at Day 1. During the treatment, laboratory assessments must be performed within 3 days prior to the infusion. Results for safety bloods must be available and reviewed before commencing an infusion.

f Free T3 and free T4 will only be measured if TSH is abnormal. They should also be measured if there is clinical suspicion of an adverse event related to the endocrine system.

g Urinalysis performed at Screening, every 4 weeks and as clinically indicated.

h Coagulation tests: prothrombin time, APTT and INR – only performed at Screening and as clinically indicated.

i CT (preferred) or MRI scans, preferably with IV contrast, are collected during screening (for baseline) and as close to and prior to initiation of study treatment. Response according to RECIST 1.1 criteria (CR, PR) requires a confirmatory scan preferably at the next regularly scheduled imaging visit and no earlier than 4 weeks after the prior assessment of CR, PR, or SD. CT scan evaluation should be perform every 3 months at fixed time.

j For AEs/SAEs reported during prescreening additional information such as medical history and concomitant medications may be needed.

k Blood sampling must be done at visit 12. If patient stop the study before visit 12, blood samples must be drawn at end of study visit (EOT). If blood sampling is performed at visit 12, no need to perform blood sampling at EOT.

l A new biopsy is required only if no sufficient archiving material is available

m Biopsy use for principal study will be used for ancillary study (archiving material or fresh biopsy). If the cure is postponed, the biopsy must be postponed as well. Biopsies should always match C5, C12.

n Blood sample performed only if patient signed the genetic informed consent form

o If the cure is postponed, the blood samples for ancillary study must be postponed as well. Biological samples have to be collected at cycle 1-2-5 and 12 (or EOT).

TABLE OF CONTENTS PAGE

DESIGN OF THE STUDY 2
PROTOCOL SYNOPSIS 3
TABLE OF CONTENTS 17
ABBREVIATIONS AND DEFINITION OF TERMS 22
1. INTRODUCTION 26
1.1 Disease Background 27
1.1.1 Immunotherapies 28
1.1.2 Durvalumab 28
1.1.3 Tremelimumab 29
1.1.4 Durvalumab in combination with tremelimumab 30
1.2 Research hypothesis 31
1.3 Rationale for conducting this study 31
1.3.1 Durvalumab + tremelimumab combination therapy dose rationale 32
1.3.2 Rationale for 4 cycles of combination therapy followed by durvalumab monotherapy 33
1.4 Benefit/risk and ethical assessment 34
1.4.1 Potential benefits 34
1.4.1.1 Durvalumab 34
1.4.1.2 Tremelimumab 34
1.4.1.3 Durvalumab tremelimumab 35
1.4.2 Potential risks 35
1.4.2.1 Durvalumab 35
1.4.2.2 Tremelimumab 37
1.4.2.3 Durvalumab + tremelimumab 37
1.4.2.4 Fixed Dosing for durvalumab and tremelimumab 38
2. STUDY OBJECTIVE 39
2.1 Primary objective(s) 39
2.2 Secondary objective 40
2.3 Exploratory objective(s) 40
3. STUDY DESIGN 41
3.1 Overview of study design 41
3.2 Study schema 41
3.3 Study Oversight for Safety Evaluation 42

4. PATIENT SELECTION, ENROLLMENT, RESTRICTIONS, DISCONTINUATION AND WITHDRAWAL 42
4.1 Inclusion criteria....42
4.2 Exclusion criteria 44
4.3 Withdrawal of Subjects from Study Treatment and/or Study 46
4.4 Replacement of subjects 47
5. INVESTIGATIONAL PRODUCT(S)....47
5.1 Durvalumab and tremelimumab....47
5.1.1 Formulation/packaging/storage....47
5.2 Dose and treatment regimens....47
5.2.1 Treatment regimens....47
5.2.2 Duration of treatment and criteria for retreatment....48
5.2.3 Study drug preparation of durvalumab and tremelimumab 49
5.2.4 Monitoring of dose administration....51
5.2.5 Accountability and dispensation....52
5.2.6 Disposition of unused investigational study drug 52
5.3 FOLFOX 52
5.3.1 Formulation/packaging/storage....52
5.3.2 Product preparation, Doses and treatment regimens administration....52
5.3.3 Monitoring of dose administration....53
5.3.4 Accountability and dispensation....53
5.3.5 Disposition of unused investigational study drug 53
6. TREATMENT PLAN....53
6.1 Subject enrollment 53
6.1.1 Procedures for enrolment 53
6.1.2 Procedures for handling subjects incorrectly enrolled....53
6.1.3 Procedures for handling subjects lost of follow up or untreated enrolled 54
6.2 Dose Modification and Toxicity Management 54
6.2.1 Durvalumab and tremelimumab....54
6.2.2 FOLFOX (5-Fluorouracil + oxaliplatin)....55
6.2.3 Toxicity management guidelines for combination treatment regimen 59
7. RESTRICTIONS DURING THE STUDY AND CONCOMITANT TREATMENT(S)....60
7.1 Restrictions during the study 60

7.2 Concomitant treatment(s)....................60
7.2.1 Permitted concomitant medications....................61
7.2.2 Excluded Concomitant Medications....................61
8. STUDY PROCEDURES....................63
8.1 Schedule of study procedures....................63
8.1.1 Screening Phase....................63
8.1.2 Treatment Phase....................64
8.1.3 End of Treatment....................64
8.1.4 Explorative study....................65
8.2 Description of study procedures....................65
8.2.1 Medical history and physical examination, electrocardiogram, weight and vital signs....................65
8.2.2 Physical examination....................65
8.2.3 Electrocardiograms....................65
8.2.4 Vital signs....................66
8.2.5 Clinical laboratory tests....................67
8.2.6 Patient reported outcomes (PRO) - QLQ-C30 and QLQ-CR29....................68
8.3 Biological sampling procedures....................68
8.3.1 Biopsy....................68
8.3.1.1 Principal study (Exome analysis and immunohistochemistry)....................68
8.3.1.2 Ancillary study (Immunohistochemistry)....................72
8.3.1.3 Tissues Samples shipment....................72
8.3.1.4 Summary of tissue sample management....................73
8.3.2. Immunomonitoring study (Ancillary study):....................74
8.3.3. Withdrawal of informed consent for donated biological samples....................76
9. DISEASE EVALUATION AND METHODS....................77
9.1. RECIST 1.1 criteria....................78
9.1.1. Measurability of tumor lesions at baseline....................78
9.1.2. Response Criteria....................78
9.2. Modified irRC methods....................80
9.3. Progression free survival (PFS)....................80
9.4. Overall survival (OS)....................80
10. ASSESSMENT OF SAFETY....................81
10.1. Safety Parameters....................81
10.1.1. Definition of adverse events....................81
10.1.2. Definition of serious adverse events....................81
10.1.3. Durvalumab + tremelimumab adverse events of special interest....................82
10.1.4. Immune-related adverse events....................84
10.1.5. Assessment of severity....................85

10.1.6. Assessment of relationship 86
10.2. Recording of adverse events and serious adverse events 86
10.2.1. Study recording period and follow-up for adverse events and serious adverse events 87
10.2.2. Reporting of serious adverse events 88
10.2.2.1. Reporting of deaths 89
10.2.3. Other events requiring reporting 89
10.2.3.1. Overdose 89
10.2.3.2. Hepatic function abnormality 89
10.2.3.3. Pregnancy 90
10.2.3.4. Maternal exposure 90
10.2.4. Paternal exposure 90
11. STATISTICAL METHODS AND SAMPLE SIZE DETERMINATION 91
11.1. Description of analysis sets for the step 1 91
11.2. Description of analysis sets for the step 2 (Simon's phase II trial) 91
11.2.1. Safety analysis set 91
11.2.2. Efficacy analysis set 91
11.3. Methods of statistical analyses 91
11.3.1. Safety Analyses 92
11.3.2. Efficacy Analyses set 92
11.3.3. Exploratory Analyses 92
11.3.4. Interim analyses 92
11.4. Determination of sample size 93
12. ETHICAL AND REGULATORY REQUIREMENTS 93
12.1. Ethical conduct of the study 93
12.2. Ethics and regulatory review 94
12.3. Informed consent 94
12.4. Changes to the protocol and informed consent form 94
12.5. Audits and inspections 95
13. STUDY MANAGEMENT 95
13.1. Training of study site personnel 95
13.2. Monitoring of the study 95
13.3. Study timetable and end of study 96
13.4. Study timetable and end of study 96
14. DATA MANAGEMENT 96

14.1 Documentation of Essential Documents/Supplements at Study Center during the study... 96

14.2 Data protection 96

15. INVESTIGATIONAL PRODUCT AND OTHER TREATMENTS..................99

15.1. Identity of investigational product(s) 99

16. LIST OF REFERENCES 99

LIST OF TABLES

Table 1. Effective methods of contraception (2 methods must be used).................................. 60

Table 2. Prohibited and Rescue Medications 62

Table 3. Hematology Laboratory Tests 67

Table 4. Clinical chemistry (Serum or Plasma) Laboratory Tests 67

Table 5. Urinalysis Tests 68

Table 6. Summary of Biopsy tissues samples 74

LIST OF FIGURES

Figure 1a: Schedule of assessment for principal study - Association phase .. Erreur ! Signet non défini.**3**

Figure 1b: Schedule of assessment for principal study - Maintenance phase. Erreur ! Signet non défini.**4**

Figure 2: Schedule of assessment of explorative study...155

LIST OF APPENDICES

Appendix 1. Dose modification and toxicity management guidelines for immune-mediated, infusion related, and non immuned-mediated reactions for the combination of durvalumab and tremelimumab 107
Appendix 2. Durvalumab DOSE CALCULATIONS 137
Appendix 3. Durvalumab DOSE VOLUME CALCULATIONS.. 138
Appendix 4. Tremelimumab DOSE CALCULATIONS.. 139
Appendix 5. Tremelimumab DOSE VOLUME CALCULATIONS 140

ABBREVIATIONS AND DEFINITION OF TERMS

The following abbreviations and special terms are used in this study Clinical Study Protocol.

Abbreviation or special term	Explanation
AChE	Acetylcholine esterase
ADA	Anti-drug antibody
AE	Adverse event
AESI	Adverse event of special interest
ALK	Anaplastic lymphoma kinase
ALT	Alanine aminotransferase
APF12	Proportion of patients alive and progression free at 12 months from randomization
AST	Aspartate aminotransferase
AUC	Area under the curve
$AUC_{0\text{-}28day}$	Area under the plasma drug concentration-time curve from time zero to Day 28 post-dose
AUC_{ss}	Area under the plasma drug concentration-time curve at steady state
BICR	Blinded Independent Central Review
BoR	Best objective response
BP	Blood pressure
C	Cycle
CD	Cluster of differentiation
CI	Confidence interval
CL	Clearance
C_{max}	Maximum plasma concentration
$C_{max,ss}$	Maximum plasma concentration at steady state
CR	Complete response
CSA	Clinical study agreement
CSR	Clinical study report
CT	Computed tomography

Abbreviation or special term	Explanation
CTCAE	Common Terminology Criteria for Adverse Event
CTLA-4	Cytotoxic T–lymphocyte-associated antigen 4
$C_{trough,ss}$	Trough concentration at steady state
CXCL	Chemokine (C-X-C motif) ligand
DoR	Duration of response
EC	Ethics Committee, synonymous to Institutional Review Board and Independent Ethics Committee
ECG	Electrocardiogram
ECOG	Eastern Cooperative Oncology Group
eCRF	Electronic case report form
EDoR	Expected duration of response
EGFR	Epidermal growth factor receptor
EU	European Union
FAS	Full analysis set
FDA	Food and Drug Administration
GCP	Good Clinical Practice
GI	Gastrointestinal
GMP	Good Manufacturing Practice
hCG	Human chorionic gonadotropin
HIV	Human immunodeficiency virus
HR	Hazard ratio
IB	Investigator's Brochure
ICF	Informed consent form
ICH	International Conference on Harmonization
IDMC	Independent Data Monitoring Committee
IFN	Interferon
IgE	Immunoglobulin E
IgG	Immunoglobulin G
IHC	Immunohistochemistry
IL	Interleukin

Abbreviation or special term	Explanation
ILS	Interstitial lung disease
IM	Intramuscular
IMT	Immunomodulatory therapy
IP	Investigational product
irAE	Immune-related adverse event
IRB	Institutional Review Board
irRECIST	Immune-related Response Evaluation Criteria in Solid Tumors
ITT	Intent-to-Treat
IV	Intravenous
IVRS	Interactive Voice Response System
IWRS	Interactive Web Response System
mAb	Monoclonal antibody
MDSC	Myeloid-derived suppressor cell
MedDRA	Medical Dictionary for Regulatory Activities
MHLW	Minister of Health, Labor, and Welfare
miRNA	Micro-ribonucleic acid
MRI	Magnetic resonance imaging
NCI	National Cancer Institute
NE	Not evaluable
NSCLC	Non–small-cell lung cancer
OAE	Other significant adverse event
ORR	Objective response rate
OS	Overall survival
PBMC	Peripheral blood mononuclear cell
PD	Progressive disease
PD-1	Programmed cell death 1
PD-L1	Programmed cell death ligand 1
PD-L2	Programmed cell death ligand 2
PDx	Pharmacodynamic(s)
PFS	Progression-free survival

Abbreviation or special term	Explanation
PFS2	Time to second progression
PGx	Pharmacogenetic research
PK	Pharmacokinetic(s)
PR	Partial response
q2w	Every 2 weeks
q3w	Every 3 weeks
q4w	Every 4 weeks
q6w	Every 6 weeks
q8w	Every 8 weeks
QTcF	QT interval corrected for heart rate using Fridericia's formula
RECIST 1.1	Response Evaluation Criteria in Solid Tumors, version 1.1
RNA	Ribonucleic acid
RR	Response rate
RT-QPCR	Reverse transcription quantitative polymerase chain reaction
SAE	Serious adverse event
SAP	Statistical analysis plan
SAS	Safety analysis set
SD	Stable disease
SNP	Single nucleotide polymorphism
SoC	Standard of Care
sPD-L1	Soluble programmed cell death ligand 1
T_3	Triiodothyronine
T_4	Thyroxine
TSH	Thyroid-stimulating hormone
ULN	Upper limit of normal
US	United States
WBDC	Web-Based Data Capture
WHO	World Health Organization

1. INTRODUCTION

Colo-rectal cancer (CRC) is still one of the leading causes of cancer death worldwide. In France, approximately 40 500 new cases are diagnosed each year. With more than 17 500 deaths in France in 2011, colo-rectal cancer is responsible for more than 12 percent of all cancer deaths, the overwhelming of deaths occurring in patients with metastatic disease. Metastatic disease treatment relies mainly on chemotherapy with more often palliative objective when metastases could not be removed. Approximately half of the patients with CRC will develop metastases, with a liver localization in 50-70 % of cases; only 10-20 % will be accessible to curative resection. For the 80-90 % of the remaining cases, the prognosis is bad (DM Parkin, 2005) however the median overall survival of CRC patients increases with improvement of chemotherapeutic protocol from 12 months with 5-fluororuracil monotherapy to around 30 months in recent clinical trials (M Van den Eynde, 2009; JY Douillard 2000; A Johnson 2013; BJ Giantonio 2007; LB Saltz 2008; J Bennouna 2013; D Cunnigham 2004)

The conventional treatment of non resecable metastatic colorectal cancer is based on systematic chemotherapy. The aim of this treatment is to maintain the quality of life and to extend patient survival. Drugs having demonstrated an efficacy are fluoropyrimidines, irinotecan in monotherapy or in association with fluoropyrimidine, and oxaliplatin in association with fluoropyrimidines. Recently, targeted therapies strengthened the armamentarium. Indeed, bevacizumab presents an interest in association with fluoropyrimidines, oxaliplatin and irinotecan. Anti-EGFR antibodies (panitumumab and cetuximab) present an efficacy in patients bearing metastic tumors without mutation of the KRAS and NRAS genes (National Thesaurus of Digestive Cancerology) (Y Douillard 2013; E Van Custem 2009; M Peeters 2015). In second line, aflibercept, a recombinant molecule targeting anti-angiogenic factor improves PFS and OS in association with fluoropyrimidine and irinotecan (Van Custem 2012). In third line, the new multitarget tyrosine kinase inhibitor regorafenib was shown to improve survival (Grothey A 2013).

In addition to chemotherapy a promising approach of clinical development in colo-rectal cancer is immunotherapy. Many studies highlight the fact that colo-rectal cancer can be recognized by the immune system and it is well admit that CD4/CD8 T cells infiltrates are associated with cancer prognosis. Recent data have provided a clearer understanding of the factors limiting the antitumor immune response in colo-rectal cancer. Firstly, inhibition of CTLA-4 signaling is a validated approach to cancer therapy. Then, the most critical checkpoint pathways responsible for mediating tumor-induced immune suppression is the programmed death-1 (PD-1) and PD ligand 1 (PD-L1) pathway. This discovery was linked to the development of new immunotherapies in cancer treatment. Anti-PD-1/PD-L1 mAbs give antitumoral response in many different types of human cancer. PD-1 is expressed on activated immune cells (T cells, natural killer) and can link to PD-L1 (PD ligand 1) express on APC (Antigen-Presenting-Cell) or on tumor cells. Usually, the interaction between PD1 and PDL1 induce inhibition of T cells proliferation survival, and effectors functions (cytotoxicity and cytokine release); induces apoptosis of tumor-specific T cells thus promoting T-cells tolerance and preventing tissue damage in settings of chronic inflammation. In pathological context, such as cancer, the PD-1/PD-L1 pathway contributes to immune suppression and evasion. In the field of colorectal cancer a resent phase I study, underline some impressive response rate in patients with colorectal tumor with mismatch repair deficiency (Le DT, 2015). In contrast response rate to monotherapy against PD1 seems very modest.

Recent preclinical data suggest that combination of PD1/PDL1 inhibitor with immunogenic cell death inducer like radiotherapy or oxaliplatin could enhance the efficacy of such treatment (Dovedi 2014; Twyman Saint Victor 2015; Stewart 2015). Furthermore, the Pr Ghiringhelli team (Dijon) focuses on immunotherapy in colo-rectal cancer. Preliminary preclinical work in two in vivo tumor models of colon cancer in mice shows a strong interest in using an anti-PD-L1 in combination with standard treatment of colo-rectal cancer (FOLFOX). In these models, the survival of mice that are treated with the combination therapy reached 40% when no mice were alive with FOLFOX treatment alone. Our work raises the hypothesis that 5FU and oxaliplatin not only kill cancer cell but also enhance the immune response against cancer and by this way favor the efficacy of anti-PD-L1. These results suggest that the combination of chemotherapy with immunotherapy would act synergistically in patients with colo-rectal cancer.

1.1 Disease Background

Immune responses directed against tumors are one of the body's natural defence against the growth and proliferation of cancer cells. However, over time and under pressure from immune attack, cancers develop strategies to evade immune-mediated killing allowing them to develop unchecked. One such mechanism involves upregulation of surface proteins that deliver inhibitory signals to cytotoxic T cells. Programmed cell death ligand 1 (PD-L1) is one such protein, and is upregulated in a broad range of cancers with a high frequency, with up to 88% expression in some tumor types. In a number of these cancers, including lung (Mu et al, 2011), renal (Thompson et al, 2005; Thompson et al, 2006; Krambeck et al, 2007), pancreatic (Nomi et al, 2007; Loos et al, 2008; Wang et al, 2010), ovarian cancer (Hamanishi et al, 2007), and hematologic malignancies (Andorsky et al, 2011; Brusa et al, 2013) tumor cell expression of PD-L1 is associated with reduced survival and an unfavorable prognosis.

Programmed cell death ligand 1 is part of a complex system of receptors and ligands that are involved in controlling T-cell activation. PD-L1 acts at multiple sites in the body to help regulate normal immune responses and is utilized by tumors to help evade detection and elimination by the host immune system tumor response. In the lymph nodes, PD-L1 on antigen-presenting cells binds to PD-1 or CD80 on activated T cells and delivers an inhibitory signal to the T cell (Keir et al, 2008; Park et al, 2010). This results in reduced T-cell activation and fewer activated T cells in circulation. In the tumor microenvironment, PD-L1 expressed on tumor cells binds to PD-1 and CD80 on activated T cells reaching the tumor. This delivers an inhibitory signal to those T cells, preventing them from killing target cancer cells and protecting the tumor from immune elimination (Zou and Chen, 2008).

Immune responses directed against tumors are one of the body's natural defenses against the growth and proliferation of cancer cells. T cells play a critical role in antitumor immunity and their infiltration and activity have been linked to improved prognosis in a number of cancers (Pagès et al, 2010; Nakano et al, 2001; Suzuki et al, 2011; Burt et al, 2011). Immune evasion, primarily through suppression of T-cell activity, is now recognized as one of the hallmarks of cancer. Such evasion can occur via a range of mechanisms including production of suppressive cytokines such as IL-10, secretion of chemokines and growth factors that recruit and sustain suppressive regulatory T cells (Tregs) and inflammatory macrophages, and expression of inhibitory surface molecules such as B7-H1. Tumor types characterized as being responsive to immunotherapy-based approaches include melanoma (Weber et al, 2012), renal cell carcinoma (RCC; McDermott, 2009), bladder cancer (Kresowik and Griffith, 2009), and malignant mesothelioma (Bograd et al, 2011). Inhibition of CTLA-4

signaling is a validated approach to cancer therapy, as shown by the approval in 2011 of ipilimumab for the treatment of metastatic melanoma based on statistically significant and clinically meaningful improvement in OS (Hodi et al, 2010; Robert et al, 2011).

In general, tumor response rates to anti-CTLA-4 therapy are low (~10%). However, in patients who respond, the responses are generally durable, lasting several months even in patients with aggressive tumors such as refractory metastatic melanoma. Because these agents work through activation of the immune system and not by directly targeting the tumor, responses can occur late and some patients may have perceived progression of their disease in advance of developing disease stabilization or a tumor response. In some cases, early growth of pre-existing lesions or the appearance of new lesions may have been due to immune-cell infiltration into the tumor and not due to proliferation and extension of neoplastic cells, per se (Wolchok et al, 2009). Overall, although the impact on conventionally defined PFS can be small, durable response or stable disease seen in a proportion of patients can lead to significant prolongation of OS. The melanoma data with ipilimumab clearly demonstrate that a small proportion of patients with an objective response had significant prolongation of OS, supporting the development of this class of agents in other tumors. Although Phase 2 and Phase 3 studies of tremelimumab in metastatic melanoma did not meet the primary endpoints of response rate and OS, respectively, the data suggest activity of tremelimumab in melanoma (Kirkwood et al, 2010; Ribas et al, 2013). In a large Phase 3 randomized study comparing tremelimumab with dacarbazine (DTIC)/temozolomide in patients with advanced melanoma, the reported median OS in the final analysis was 12.58 months for tremelimumab versus 10.71 months for DTIC/temozolomide (HR = 1.1416, p = 0.1272; Ribas et al, 2013).

1.1.1 Immunotherapies

It is increasingly understood that cancers are recognized by the immune system, and, under some circumstances, the immune system may control or even eliminate tumors (Dunn et al 2004). Studies in mouse models of transplantable tumors have demonstrated that manipulation of co-stimulatory or co-inhibitory signals can amplify T-cell responses against tumors (PEGGS et al 2009). This amplification may be accomplished by blocking co-inhibitory molecules, such as cytotoxic T-lymphocyte-associated antigen 4 (CTLA-4) or programmed cell death 1 (PD-1), from binding with their ligands, B7 or B7-H1 (programmed cell death ligand 1 [PD-L1]).

1.1.2 Durvalumab

The non-clinical and clinical experience is fully described in the current version of the durvalumab Investigator's Brochure (IB Version 9.0).

Durvalumab is a human monoclonal antibody (mAb) of the immunoglobulin G (IgG) 1 kappa subclass that inhibits binding of PD-L1 and is being developed by AstraZeneca/MedImmune for use in the treatment of cancer. (MedImmune is a wholly owned subsidiary of AstraZeneca; AstraZeneca/MedImmune will be referred to as AstraZeneca throughout this document.) As durvalumab is an engineered mAb, it does not induce antibody-dependent cellular cytotoxicity or complement-dependent cytotoxicity. The proposed mechanism of action for durvalumab is interference of the interaction of PD-L1.

PD-L1 is expressed in a broad range of cancers with a high frequency, up to 88% in some types of cancers. In a number of these cancers, including lung, the expression of PD-L1 is associated with reduced survival and an unfavorable prognosis. In lung cancer, only 12% of patients with tumors expressing PD-L1 survived for more than 3 years, compared with 20% of patients with tumors lacking PD-L1 (Mu et al 2011). Based on these findings, an anti-PD-L1 antibody could be used therapeutically to enhance anti-tumor immune responses in patients with cancer. Results of several non-clinical studies using mouse tumor models support this hypothesis, where antibodies directed against PD-L1 or its receptor PD-1 showed anti-tumor activity (Hirano et al 2005, Iwai et al 2002, Okudaira et al 2009, Zhang et al 2008).

Durvalumab has been given to humans as part of ongoing studies as a single drug or in combination with other drugs As of the DCO dates (15Apr2015 to 12Jul2015, durvalumab IB version 8.0), a total of 1,883 subjects have been enrolled and treated in 30 ongoing durvalumab clinical studies, including 20 sponsored and 10 collaborative studies. Of the 1,883 subjects, 1,279 received durvalumab monotherapy, 440 received durvalumab in combination with tremelimumab or other anticancer agents, 14 received other agents (1 gefitinib, 13 MEDI6383), and 150 have been treated with blinded investigational product. No studies have been completed or terminated prematurely due to toxicity.

As of 09Feb2015, PK data were available for 378 subjects in the dose-escalation and dose-expansion phases of Study CD-ON-durvalumab-1108 following treatment with durvalumab 0.1 to 10 mg/kg every 2 weeks (Q2W) or 15 mg/kg every 3 weeks (Q3W). The maximum observed concentration (C_{max}) increased in an approximately dose-proportional manner over the dose range of 0.1 to 15 mg/kg. The area under the concentration-time curve from 0 to 14 days (AUC_{0-14}) increased in a greater than dose-proportional manner over the dose range of 0.1 to 3 mg/kg and increased dose-proportionally at ≥ 3 mg/kg. These results suggest durvalumab exhibits nonlinear PK likely due to saturable target-mediated CL at doses < 3 mg/kg and approaches linearity at doses ≥ 3 mg/kg. Near complete target saturation (soluble programmed cell death ligand 1 [sPD-L1] and membrane bound) is expected with durvalumab ≥ 3 mg/kg Q2W. Exposures after multiple doses showed accumulation consistent with PK parameters estimated from the first dose. In addition, PK simulations indicate that following durvalumab 10 mg/kg Q2W dosing, > 90% of subjects are expected to maintain PK exposure ≥ 40 µg/mL throughout the dosing interval.

As of 09Feb2015, a total of 388 subjects provided samples for ADA analysis. Only 8 of 388 subjects (1 subject each in 0.1, 1, 3, and 15 mg/kg cohorts, and 4 subjects in 10 mg/kg cohort) were ADA positive with an impact on PK/pharmacodynamics in 1 subject in the 3 mg/kg cohort.

1.1.3 Tremelimumab

The non-clinical and clinical experience is fully described in the current version of the tremelimumab Investigator's Brochure (IB Version 5.0).

Tremelimumab is an IgG 2 kappa isotype mAb directed against the cytotoxic T-lymphocyte-associated protein 4 (CTLA-4) also known as CD152 (cluster of differentiation 152) This is an immunomodulatory therapy (IMT) that is being developed by AstraZeneca for use in the treatment of cancer.

Binding of CTLA-4 to its target ligands (B7-1 and B7-2) provides a negative regulatory signal, which limits T-cell activation. Anti-CTLA-4 inhibitors antagonize the binding of CTLA-4 to B7 ligands and

enhance human T-cell activation as demonstrated by increased cytokine (interleukin [IL]-2 and interferon [IFN] gamma) production in vitro in whole blood or peripheral blood mononuclear cell (PBMC) cultures (Tarhini and Kirkwood 2008). In addition, blockade of CTLA-4 binding to B7 by anti-CTLA-4 antibodies results in markedly enhanced T-cell activation and anti-tumor activity in animal models, including killing of established murine solid tumors and induction of protective anti-tumor immunity. (Refer to the tremelimumab IB, Edition 5.0, for more information.) Therefore, it is expected that treatment with an anti-CTLA-4 antibody, such as tremelimumab, will lead to increased activation of the human immune system, increasing anti-tumor activity in patients with solid tumors.

An extensive program of non-clinical and clinical studies has been conducted for tremelimumab both as monotherapy and combination therapy with conventional anticancer agents to support various cancer indications using different dose schedules. As of the data cut-off date of 12 November 2014 (for all studies except D4190C00006, which has a cut-off date of 4 December 2014), 973 patients have received tremelimumab monotherapy (not including 497 patients who have been treated in the blinded Phase 2b study, D4880C00003) and 208 patients have received tremelimumab in combination with other agents. Details on the safety profile of tremelimumab monotherapy are summarized in Section 1.4.2.2. Refer to the current tremelimumab IB for a complete summary of non-clinical and clinical information; see Section 6.6 for guidance on management of tremelimumab-related toxicities.

Tremelimumab exhibited a biphasic PK profile with a long terminal phase half-life of 22 days. Overall, a low incidence of ADAs (<6%) was observed for treatment with tremelimumab.

1.1.4 Durvalumab in combination with tremelimumab

Targeting both PD-1 and CTLA-4 pathways may have additive or synergistic activity (Pardoll 2012) because the mechanisms of action of CTLA-4 and PD-1 are non-redundant; therefore, AstraZeneca is also investigating the use of durvalumab + tremelimumab combination therapy for the treatment of cancer.

Study D4190C00006 is a Phase Ib dose-escalation study to establish safety, PK/PDx, and preliminary anti-tumor activity of durvalumab + tremelimumab combination therapy in patients with advanced NSCLC. The dosing schedule utilized is durvalumab every 2 weeks (q2w) or every 4 weeks (q4w) up to Week 50 and 48 (12 months), combined with tremelimumab q4w up to Week 24 for 7 doses then every 12 weeks for 2 additional doses for up to 12 months. The study is ongoing and continues to accrue.

Study D4190C00006: As of 20Feb2015, durvalumab PK (n = 55) and tremelimumab PK (n = 26) data were available from 10 cohorts (1a, 2a, 3a, 3b, 4, 4a, 5, 5a, 8, and 9) following durvalumab every 4 weeks (Q4W) or Q2W dosing in combination with tremelimumab Q4W regimens. An approximately dose-proportional increase in PK exposure (C_{max} and area under the concentration-time curve from 0 to 28 days [AUC_{0-28}]) of both durvalumab and tremelimumab was observed over the dose range of 3 to 15 mg/kg durvalumab Q4W and 1 to 10 mg/kg tremelimumab Q4W. Exposures following multiple doses demonstrated accumulation consistent with PK parameters estimated from the first dose. It is to be noted that steady state PK parameters are based on limited numbers of subjects. The observed PK exposures of durvalumab and tremelimumab following combination were consistent with respective monotherapy data, indicating no PK interaction between these 2 agents.

As of 20Feb2015, ADA data were available from 60 subjects for durvalumab and 53 subjects for tremelimumab in Study D4190C00006. Four of 60 subjects were ADA positive for anti-durvalumab antibodies post treatment. One of 53 subjects was ADA positive for anti-tremelimumab antibodies post treatment. There was no clear relationship between ADA and the dose of either durvalumab or tremelimumab, and no obvious association between ADA and safety or efficacy.

Durvalumab has also been combined with other anticancer agents, including gefitinib, dabrafenib, and trametinib. To date, no PK interaction has been observed between durvalumab and these agents.

1.2 Research hypothesis

Many clinical and preclinical study underline that inhibitors of PD-1/PD-L1 pathway improves the immune response by restoring T-cell effectors functions, including cytolytic activity against tumor cells. However despite their efficacy in many cancer types this drug is not effective in all patients. Such data raise the hypothesis that combination of PD1/PDL1 blockade in association with chemotherapy could be an effective pathway to enhance the number of responder patients.

The rationale for evaluating the combination of MEDI4736 and tremelimumab in human studies is supported by nonclinical data in mouse models of transplantable solid tumors that show superior antitumor activity of combination therapy over monotherapy (Curran et al, 2010; Mangsbo et al, 2010). Secondly, the mechanisms of action for CTLA-4 and PD-1 are non-redundant; suggesting that targeting both pathways may have additive or synergistic activity (Pardoll, 2012). These observations suggest that combination therapy may generate superior antitumor activity (compared to monotherapy), which may translate into high rates of response in NSCLC. However, the combination of these 2 agents may increase the frequency or severity of toxicities and thus, a Phase 1 dose-escalation study in the appropriate setting to explore this combination is indicated.

In addition it is currently well known that chemotherapy in addition to its cytotoxic effect could also have positive effect on the immune system. This is notably the case for 5FU, which could reduce tumor, induced immune tolerance via its capacity to eliminate myeloid derived suppressor cells (Vincent et al Cancer Res 2010). In addition, oxaliplatin by its capacity to induce immunogenic cell death could boost antitumor immune response. Preliminary preclinical work in two in vivo tumor models of colon cancer in mice shows a strong interest in using an anti-PD-L1 in combination with standard treatment of colo-rectal cancer (FOLFOX). In these models, the survival of mice that are treated with the combination therapy reached 40% when no mice were alive with FOLFOX treatment alone. These results suggest that the combination of chemotherapy with immunotherapy would act synergistically in patients with colo-rectal cancer.

1.3 Rationale for conducting this study

As an antibody that blocks the interaction between PD-L1 and its receptors, durvalumab may relieve PD-L1-dependent immunosuppressive effects and, therefore, enhance the cytotoxic activity of anti-tumor T-cells. This hypothesis is supported by emerging clinical data from other mAbs targeting the PD-L1/PD-1 pathway, which provide early evidence of clinical activity and a manageable safety profile (Brahmer et al 2012, Topalian et al 2012). Responses have been observed in patients with PD-L1-positive tumors and patients with PD-L1-negative tumors. In addition, durvalumab monotherapy has shown durable responses in NSCLC in Study 1108 (see Section 1.4.1.1).

The rationale for combining durvalumab and tremelimumab is that the mechanisms of CTLA 4 and PD-1 are non-redundant, suggesting that targeting both pathways may have additive or synergistic activity (Pardoll 2012). In fact, combining immunotherapy agents has been shown to result in improved response rates (RRs) relative to monotherapy. For example, the concurrent administration of nivolumab and ipilimumab to patients with advanced melanoma induced higher objective response rates (ORRs) than those obtained with single-agent therapy. Importantly, responses appeared to be deep and durable (Wolchok et al 2013). Similar results have been observed in an ongoing study of durvalumab + tremelimumab in NSCLC (Antonia et al 2014), with further updated details presented in this clinical study protocol.

1.3.1 Durvalumab + tremelimumab combination therapy dose rationale

The durvalumab + tremelimumab doses and regimen selected for this study are based on the goal of selecting an optimal combination dose of durvalumab and tremelimumab that would yield sustained target suppression (sPD-L1), demonstrate promising efficacy, and have an acceptable safety profile.

In order to reduce the dosing frequency of durvalumab to align with the q4w dosing of tremelimumab, while ensuring an acceptable PK/PDx, safety, and efficacy profile, cohorts were narrowed to 15 and 20 mg/kg durvalumab q4w. PK simulations from the durvalumab monotherapy data indicated that a similar area under the plasma drug concentration-time curve at steady state (AUC_{ss}; 4 weeks) was expected following both 10 mg/kg q2w and 20 mg/kg q4w durvalumab. The observed durvalumab PK data from the D4190C00006 study were well in line with the predicted monotherapy PK data developed preclinical. This demonstrates similar exposure of durvalumab 20 mg/kg q4w and 10 mg/kg q2w, with no alterations in PK when durvalumab and tremelimumab (doses ranging from 1 to 3 mg/kg) are dosed together. While the median C_{max} at steady state ($C_{max,ss}$) is expected to be higher with 20 mg/kg q4w (approximately 1.5 fold) and median trough concentration at steady state ($C_{trough,ss}$) is expected to be higher with 10 mg/kg q2w (approximately 1.25 fold), this is not expected to impact the overall safety and efficacy profile, based on existing preclinical and clinical data.

Monotonic increases in PDx activity were observed with increasing doses of tremelimumab relative to the activity observed in patients treated with durvalumab monotherapy. There was evidence of augmented PDx activity relative to durvalumab monotherapy with combination doses containing 1 mg/kg tremelimumab, inclusive of both the 15 and 20 mg/kg durvalumab plus 1 mg/kg tremelimumab combinations.

Patients treated with doses of tremelimumab above 1 mg/kg had a higher rate of adverse events (AEs), including discontinuations due to AEs, serious AEs (SAEs), and severe AEs. Between the 10 mg/kg durvalumab + 1 mg/kg tremelimumab and 10 mg/kg durvallumab + 3 mg/kg tremelimumab cohorts treated at the q2w schedule, the number of patients reporting any AE, Grade 3 AEs, SAEs, and treatment-related AEs was higher in the 10 mg/kg durvalumab + 3 mg/kg tremelimumab cohort than the 10 mg/kg durvalumab + 1 mg/kg tremelimumab cohort. A similar pattern was noted in the q4w regimens, suggesting that, as the dose of tremelimumab increased above 1 mg/kg, a higher rate of treatment-related events may be anticipated. Further, the SAEs frequently attributed to immunotherapy, pneumonitis and colitis, were more commonly seen in cohorts using either 3 or 10 mg/kg of tremelimumab compared to the 1-mg/kg dose cohorts. Together, these data suggest that a combination using a tremelimumab dose of 1 mg/kg appeared to minimize the rate of toxicity when

combined with durvalumab. As a result, all combination doses utilizing either the 3 or 10 mg/kg doses of tremelimumab were eliminated in the final dose selection.

In contrast, cohorts assessing higher doses of durvalumab with a constant dose of tremelimumab did not show an increase in the rate of AEs. The data suggested that increasing doses of durvalumab might not impact the safety of the combination as much as the tremelimumab dose. Further, safety data between the 10-mg/kg and 20-mg/kg cohorts were similar, with no change in safety events with increasing dose of durvalumab.

In Study D4190C00006, of all treatment cohorts, the cohort of 11 patients treated in the 20 mg/kg durvalumab + 1 mg/kg tremelimumab group had the fewest AEs, Grade ≥3 AEs, SAEs, and treatment discontinuations due to AEs, but still showed strong evidence of clinical activity. This cohort had a lower number of treatment-related Grade ≥3 AEs or treatment-related SAEs. No dose-limiting toxicities were reported.

Preliminary clinical activity of the durvalumab and tremelimumab combination did not appear to change with increasing doses of tremelimumab. The 15- and 20-mg/kg durvalumab q4w cohorts demonstrated objective responses at all doses of tremelimumab, and increasing doses of tremelimumab did not provide deeper or more rapid responses.

Efficacy data suggested that the 20 mg/kg durvalumab + 1 mg/kg tremelimumab dose cohort may demonstrate equivalent clinical activity to other dose combinations. A total of 5 of 11 patients in the 20 mg/kg durvalumab + 1 mg/kg tremelimumab cohort were evaluable for efficacy with at least 8 weeks of follow-up. Of these, there were 2 patients (40%) with partial response (PR), 1 patient (20%) with stable disease (SD), and 1 patient (20%) with progressive disease (PD). (The fifth patient had only a single scan, which was conducted outside the window for these evaluations.)

Additionally, of all cohorts, the 20 mg/kg durvalumab + 1 mg/kg tremelimumab dose cohort had the fewest AEs, Grade ≥3 AEs, SAEs, and treatment discontinuations due to AEs, but still showed some evidence of clinical activity. All together, the data suggested that a 20 mg/kg durvalumab + 1 mg/kg tremelimumab dose combination should be selected for further development.

1.3.2 Rationale for 4 cycles of combination therapy followed by durvalumab monotherapy

Long-term follow up on melanoma patients treated with ipilimumab, an anti-CTLA-4 targeting antibody (dosed every 3 weeks (Q3W) for 4 doses and then discontinued), shows that patients responding to ipilimumab derive long-term benefit, with a 3-year OS rate of approximately 22%. Furthermore, the survival curve in this population reached a plateau at 3 years and was maintained through 10 years of follow up (Schadendorf et al 2013).

Similar data have been presented for other anti-PD-1/PD-L1 targeting antibodies:

Nivolumab (anti-PD-1) was dosed q2w for up to 96 weeks in a large Phase I dose-escalation and expansion study, and showed responses were maintained for a median of 22.94 months for melanoma (doses 0.1 mg/kg to 10 mg/kg), 17 months for NSCLC (doses 1, 3, and 10 mg/kg), and 12.9 months for renal cell carcinoma patients (doses 1 and 10 mg/kg) at the time of data analysis (Hodi et al 2014,

Brahmer et al 2014, Drake et al 2013). Furthermore, responses were maintained beyond treatment discontinuation in the majority of patients who stopped nivolumab treatment (either due to protocol specified end of treatment, complete response (CR), or toxicity) for up to 56 weeks at the time of data analysis (Topalian et al 2014).

MPDL3280a (anti-PD-L1) and the combination of nivolumab with ipilimumab, in which patients were dosed for a finite time period and responses maintained beyond treatment discontinuation have been reported (Herbst et al 2013, Wolchok et al 2013).

Similar long term results may be expected with use of other immune-mediated cancer therapeutics including anti-CTLA-4 antibodies such as tremelimumab, anti PD-L1 antibodies such as durvalumab, or the combination of the two.

1.4 Benefit/risk and ethical assessment

1.4.1 Potential benefits

1.4.1.1 Durvalumab

The majority of the safety and efficacy data currently available for durvalumab are based on the first time in-human, single-agent study (Study 1108) in patients with advanced solid tumors. Data from Study 1108 were presented at the European Society for Medical Oncology 2014 Congress. Overall, 456 of 694 subjects treated with durvalumab 10 mg/kg Q2W were evaluable for response (defined as having ≥ 24 weeks follow-up, measurable disease at baseline, and ≥ 1 follow-up scan, or discontinued due to disease progression or death without any follow-up scan). In PD-L1 unselected patients, the objective response rate (ORR), based on investigator assessment per Response Evaluation Criteria in Solid Tumors (RECIST) v1.1, ranged from 0% in uveal melanoma (n = 23) to 20.0% in bladder cancer (n = 15), and disease control rate at 24 weeks (DCR-24w) ranged from 4.2% in triple-negative breast cancer (TNBC; n = 24) to 39.1% in advanced cutaneous melanoma (n = 23). PD-L1 status was known for 383 of the 456 response evaluable subjects. Across the PD-L1-positive tumors, ORR was highest for bladder cancer, advanced cutaneous melanoma, hepatocellular carcinoma (HCC; n = 3 each, 33.3% each), NSCLC (n = 86, 26.7%), and squamous cell carcinoma of the head and neck (SCCHN; n = 22, 18.2%). In the PD-L1-positive subset, DCR-24w was highest in advanced cutaneous melanoma (n = 3, 66.7%), NSCLC (n = 86, 36.0%), HCC and bladder cancer (n = 3 each, 33.3% each), and SCCHN (n = 22, 18.2%). (Antonia et al 2014b).

1.4.1.2 Tremelimumab

In a single-arm, Phase II study (Study A3671008) of tremelimumab administered at 15 mg/kg every 90 days to patients with refractory melanoma, an RR of 7% and a median OS of 10 months in the second-line setting (as compared to approximately 6 months with best supportive care reported from a retrospective analysis; Korn et al 2008) were observed (Kirkwood et al 2010). In a randomized, open-label, first-line Phase III study of tremelimumab (administered at 15 mg/kg every 90 days) versus chemotherapy (dacarbazine or temozolomide) in advanced melanoma (Study A3671009), results of the final analysis showed an RR of 11% and a median OS of 12.58 months in this first-line setting as compared to 10.71 months with standard chemotherapy; however, these results were not statistically significant (Ribas et al 2013). Additionally, a Phase II maintenance study (Study A3671015) in patients

with Stage IIIB or IV NSCLC who had responded or remained stable failed to achieve statistical significance. The primary endpoint of PFS at 3 months was 22.7% in the tremelimumab arm (15 mg/kg) compared with 11.9% in the best supportive care arm (Study A3671015).

1.4.1.3 Durvalumab tremelimumab

The preclinical and clinical justification for this combination as noted in Section 1.1.4 also supports the synergy of this combination. Available data, such as those presented by Wolchok et al, suggest that the combination of agents targeting PD-1/PD-L1 and CTLA-4 may have profound and durable benefits in patients with melanoma (Wolchok et al 2013). Of the 102 subjects with advanced NSCLC treated with durvalumab in combination with tremelimumab in Study D4190C00006, 63 subjects with at least 16 weeks of follow-up were evaluable for response (defined as measurable disease at baseline and at least 1 follow-up scan; this included discontinuations due to disease progression or death without follow-up scan). Of the 63 evaluable subjects, 17 (27%) had a best overall response of PR, 14 (22%) had SD, 22 (35%) had PD, and 10 (16%) were not evaluable. The ORR (confirmed and unconfirmed CR or PR) was 27% and the DCR (CR, PR, or SD) was 49% as assessed by RECIST v1.1.

Current experience with single-agent IMT studies suggests that clinical responses may be restricted to a subset of any given patient population and that it might be beneficial to enrich the patient population by selecting patients likely to respond to therapy. To date, no assay has been established or validated, and no single approach has proven accurate, for patient enrichment for IMTs. However, independent data from multiple sources using different assays and scoring methods suggests that PD-L1 expression on tumor cells and/or tumor infiltrating cells may be associated with greater clinical benefit.

Data from ongoing studies with durvalumab and other agents targeting the PD-1/PD-L1 pathway suggest, as shown in a number of tumor types (eg, NSCLC, renal cell carcinoma, and melanoma), that monotherapy may be more efficacious (in terms of ORR) in patients who are PD-L1-positive.

Given these findings, a number of ongoing studies are assessing the activity of agents in patients with PD-L1–positive tumors. There is also an unmet medical need in patients with PD-L1–negative tumors that needs to be addressed. Data, as of 27 January 2015 from Study 006 show that with the addition of tremelimumab to durvalumab, the ORR can be increased to 25% in patients with PD-L1 negative NSCLC. As patients with PD-L1 positive disease can also have an increase in ORR, from 25% with durvalumab monotherapy, to 36% with the combination of durvalumab and tremelimumab, the study will enroll all patients with NSCLC, with an emphasis on those determined to be PD-L1 negative.

1.4.2 Potential risks

1.4.2.1 Durvalumab

Potential risks, based on the mechanism of action of durvalumab and related molecules, include immune-mediated reactions, such as enterocolitis, dermatitis, hepatitis/hepatotoxicity, endocrinopathy, pneumonitis, and neuropathy or neurologic events. Additional important potential risks include infusion-related reactions, hypersensitivity, anaphylaxis or serious allergic reactions, serious infections, and immune complex disease.

Study CD-ON-durvalumab-1108: The safety profile of durvalumab monotherapy in the 694 subjects with advanced solid tumors treated at 10 mg/kg Q2W in Study CD-ON-durvalumab-1108 has been broadly consistent with that of the overall 1,279 subjects who have received durvalumab monotherapy (not including subjects treated with blinded investigational product) across the clinical development program. The majority of treatment-related AEs were manageable with dose delays, symptomatic treatment, and in the case of events suspected to have an immune basis, the use of established treatment guidelines for immune-mediated toxicity. As of 07May2015, among the 694 subjects treated with durvalumab 10 mg/kg Q2W in Study CD-ON-durvalumab-1108, a total of 378 subjects (54.5%) experienced a treatment-related AE, with the most frequent (occurring in ≥ 5% of subjects) being fatigue (17.7%), nausea (8.6%), diarrhea (7.3%), decreased appetite (6.8%), pruritus (6.3%), rash (6.1%), and vomiting (5.0%). A majority of the treatment-related AEs were Grade 1 or Grade 2 in severity with ≥ Grade 3 events occurring in 65 subjects (9.4%). Treatment-related ≥ Grade 3 events reported in 3 or more subjects (≥ 0.4%) were fatigue (12 subjects, 1.7%); increased aspartate aminotransferase (AST; 7 subjects, 1.0%); increased gamma-glutamyltransferase (GGT; 6 subjects, 0.9%); increased alanine aminotransferase (ALT; 5 subjects, 0.7%); and colitis, vomiting, decreased appetite, and hyponatremia (3 subjects, 0.4% each). Six subjects had treatment-related Grade 4 AEs (upper gastrointestinal hemorrhage, increased AST, dyspnea, neutropenia, colitis, diarrhea, and pneumonitis) and 1 subject had a treatment-related Grade 5 event (pneumonia). Treatment-related serious adverse events (SAEs) that occurred in ≥ 2 subjects were colitis and pneumonitis (3 subjects each). A majority of the treatment-related SAEs were ≥ Grade 3 in severity and resolved with or without sequelae. AEs that resulted in permanent discontinuation of durvalumab were considered as treatment related in 18 subjects (2.6%), with colitis being the most frequent treatment-related AE resulting in discontinuation (3 subjects). A majority of the treatment-related AEs resulting in discontinuation of durvalumab were ≥ Grade 3 in severity and resolved with or without sequelae.

Study D4191C00003/ATLANTIC: The safety profile of durvalumab monotherapy in Study CD-ON-durvalumab-1108 is generally consistent with that of Study D4191C00003/ATLANTIC in subjects with locally advanced or metastatic non-small-cell lung cancer (NSCLC) treated with durvalumab 10 mg/kg Q2W. As of 05May2015, 264 of 303 subjects (87.1%) reported any AE in Study D4191C00003/ATLANTIC. Overall, events reported in ≥ 10% of subjects were dyspnea (18.8%), fatigue (17.8%), decreased appetite (17.5%), cough (14.2%), pyrexia (12.2%), asthenia (11.9%), and nausea (11.2%). Nearly two-thirds of the subjects experienced AEs that were Grade 1 or 2 in severity and manageable by general treatment guidelines as described in the current durvalumab study protocols. Grade 3 or higher AEs were reported in 107 of 303 subjects (35.3%). A total of 128 subjects (42.2%) reported AEs that were considered by the investigator as related to investigational product. Treatment-related AEs (all grades) reported in ≥ 2% of subjects were decreased appetite (6.6%); fatigue (5.9%); asthenia (5.0%); nausea (4.6%); pruritus (4.3%); diarrhea, hyperthyroidism, hypothyroidism, and pyrexia (3.3% each); rash (2.6%); weight decreased (2.3%); and vomiting (2.0%). Treatment-related Grade 3 AEs reported in ≥ 2 subjects were pneumonitis (3 subjects) and increased GGT (2 subjects). There was no treatment-related Grade 4 or 5 AEs. Ninety-four of 303 subjects (31.0%) reported any SAE. SAEs that occurred in ≥ 1.0% of subjects were dyspnea (6.6%); pleural effusion, general physical health deterioration (2.3% each); pneumonia (2.0%); hemoptysis, pulmonary embolism (1.3% each); and pneumonitis, respiratory failure, disease progression (1.0% each). Nine subjects had an SAE considered by the investigator as related to durvalumab. Each

treatment-related SAE occurred in 1 subject each with the exception of pneumonitis, which occurred in 3 subjects. Fifteen of 303 subjects (5.0%) have died due to an AE (pneumonia [3 subjects]; general physical health deterioration, disease progression, hemoptysis, dyspnea [2 subjects each]; pulmonary sepsis, respiratory distress, cardiopulmonary arrest [verbatim term (VT)], hepatic failure, and sepsis [1 subject each]). None of these events was considered related to durvalumab. Twenty-three of 303 subjects (7.6%) permanently discontinued durvalumab treatment due to AEs. Events that led to discontinuation of durvalumab in ≥ 2 subjects were dyspnea, general physical health deterioration, and pneumonia. Treatment-related AEs that led to discontinuation were increased ALT and increased hepatic enzyme, which occurred in 1 subject each.

1.4.2.2 Tremelimumab

Potential risks, based on the mechanism of action of tremelimumab and related molecules (ipilimumab) include potentially immune-mediated gastrointestinal (GI) events including enterocolitis, intestinal perforation, abdominal pain, dehydration, nausea and vomiting, and decreased appetite (anorexia); dermatitis including urticaria, skin exfoliation, and dry skin; endocrinopathies including hypophysitis, adrenal insufficiency, and hyperthyroidism and hypothyroidism; hepatitis including autoimmune hepatitis and increased serum ALT and AST; pancreatitis including autoimmune pancreatitis and lipase and amylase elevation; respiratory tract events including pneumonitis and interstitial lung disease (ILD); nervous system events including encephalitis, peripheral motor and sensory neuropathies, and Guillain-Barré syndrome; cytopenias including thrombocytopenia, anemia, and neutropenia; infusion-related reactions; anaphylaxis; and serious allergic reactions. The profile of AEs and the spectrum of event severity have remained stable across the tremelimumab clinical program and are consistent with the pharmacology of the target. To date, no tumor type or stage appears to be associated with unique AEs (except for vitiligo that appears to be confined to patients with melanoma). Overall, 944 of the 973 patients (97.0%) treated with tremelimumab monotherapy as of the data cutoff date of 12 November 2014 (for all studies except D4190C00006 that has a cutoff date of 04 December 2014 and not including 497 patients who have been treated in the ongoing blinded Phase IIb Study D4880C00003) experienced at least 1 AE. The events resulted in discontinuation of tremelimumab in 10.0% of patients, were serious in 36.5%, were Grade ≥3 in severity in 49.8%, were fatal in 67.7%, and were considered to be treatment related in 79.1% of patients. The frequency of any AEs and Grade ≥3 AEs was generally similar across the tremelimumab dose groups. However, a higher percentage of patients in the 10 mg/kg every 28 days and 15 mg/kg every 90 days groups compared with the All Doses <10 mg/kg group experienced treatment-related AEs, SAEs, AEs resulting in discontinuation of investigational product (IP), and deaths.

1.4.2.3 Durvalumab + tremelimumab

No safety studies in animals have been performed combining tremelimumab with durvalumab. As both CTLA-4 and PD-L1 have mechanisms of actions that enhance activation of immune cells, their potential to induce cytokine release was tested in a whole-blood assay system. Durvalumab and tremelimumab, either alone or in combination, did not induce cytokine release in blood from any donor.

Study D4190C00006: The safety profile of durvalumab and tremelimumab combination therapy in the 102 subjects with advanced NSCLC in Study D4190C00006 is generally consistent with that observed across 177 subjects treated with durvalumab and tremelimumab combination therapy (not including subjects treated with blinded investigational product). As of 15Apr2015, 95 of 102 subjects (93.1%)

reported at least 1 AE. All subjects in the tremelimumab 3 and 10 mg/kg dose cohorts experienced AEs; subjects in the durvalumab 20 mg/kg and tremelimumab 1 mg/kg Q4W cohort experienced the lowest AE rate (77.8%). Treatment-related AEs were reported in 74 of 102 subjects (72.6%), with events occurring in > 10% of subjects being diarrhea (27.5%), fatigue (22.5%), increased amylase and pruritus (14.7% each), rash (12.7%), colitis (11.8%), and increased lipase (10.8%). Treatment-related ≥ Grade 3 AEs reported in ≥ 5% of subjects were colitis (8.8%), diarrhea (7.8%), and increased lipase (5.9%). Five subjects reported treatment-related Grade 4 events (sepsis, increased ALT, and increased AST in 1 subject; increased amylase in 2 subjects; myasthenia gravis in 1 subject; and pericardial effusion in 1 subject) and 2 subjects had treatment-related Grade 5 events (polymyositis and an uncoded event of neuromuscular disorder [VT]); the Grade 4 event of myasthenia gravis and Grade 5 polymyositis occurred in 1 subject. There were 2 subjects (both in the MEDI4736 20 mg/kg + tremelimumab 3 mg/kg Q4W cohort) with dose-limiting toxicities (DLTs): 1 subject with Grade 3 increased AST, and 1 subject with Grade 3 increased amylase and Grade 4 increased lipase. Fifty-six subjects (54.9%) reported SAEs, with events occurring in > 5% of subjects being colitis (9.8%) and diarrhea (7.8%). Thirty-six subjects (35.3%) experienced treatment-related SAEs. Twenty-seven subjects (26.5%) permanently discontinued treatment due to AEs. Treatment-related AEs resulting in discontinuation in ≥ 2 subjects were colitis (7 subjects), pneumonitis (5 subjects), diarrhea (3 subjects), and increased AST (2 subjects).Additional safety results from this study are presented in Section 1.3.1 and the durvalumab IB.

In the literature (Wolchok et al 2013), using the combination of the same class of drugs (eg, anti-PD-1 and anti-CTLA4 antibodies), specifically nivolumab + ipilimumab in a study involving patients with malignant melanoma, the safety profile of this combination had shown occurrences of AEs assessed by the Investigator as treatment-related in 93% of treated patients, with the most frequent events being rash (55% of patients), pruritus (47% of patients), fatigue (38% of patients), and diarrhea (34% of patients). Grade 3 or 4 AEs, regardless of causality, were noted in 72% of patients, with Grade 3 or 4 events assessed by the Investigator as treatment-related in 53%. The most frequent of these Grade 3 or 4 events assessed by the Investigator as treatment-related include increased lipase (in 13% of patients), AST (in 13%), and ALT levels (in 11%). Frequent Grade 3 or 4 selected AEs assessed by the Investigator as treatment-related in the combination therapy included hepatic events (in 15% of patients), GI events (in 9%), and renal events (in 6%). Isolated cases of pneumonitis and uveitis were also observed.

1.4.2.4 Fixed Dosing for durvalumab and tremelimumab

A population PK model was developed for durvalumab using monotherapy data from a Phase 1 study (*study 1108; N=292; doses= 0.1 to 10 mg/kg Q2W or 15 mg/kg Q3W; solid tumors*). Population PK analysis indicated only minor impact of body weight (WT) on PK of durvalumab (coefficient of ≤ 0.5). The impact of body WT-based (10 mg/kg Q2W) and fixed dosing (750 mg Q2W) of durvalumab was evaluated by comparing predicted steady state PK concentrations (5th, median and 95th percentiles) using the population PK model. A fixed dose of 750 mg was selected to approximate 10 mg/kg (based on median body WT of ~75 kg). A total of 1000 patients were simulated using body WT distribution of 40–120 kg. Simulation results demonstrate that body WT-based and fixed dosing regimens yield similar median steady state PK concentrations with slightly less overall between-subject variability with fixed dosing regimen.

Similarly, a population PK model was developed for tremelimumab using data from Phase 1 through Phase 3 (*N=654; doses= 0.01 to 15 mg/kg Q4W or Q90D; metastatic melanoma*) [Wang et al. 2014]. Population PK model indicated minor impact of body WT on PK of tremelimumab (coefficient of ≤ 0.5). The WT-based (1 mg/kg Q4W) and fixed dosing (75 mg/kg Q4W; based on median body WT of ~75 kg) regimens were compared using predicted PK concentrations (5th, median and 95th percentiles) using population PK model in a simulated population of 1000 patients with body weight distribution of 40 to 120 kg. Similar to durvalumab, simulations indicated that both body WT-based and fixed dosing regimens of tremelimumab yield similar median steady state PK concentrations with slightly less between-subject variability with fixed dosing regimen.

Similar findings have been reported by others [Ng et al 2006, Wang et al. 2009, Zhang et al, 2012,Narwal et al 2013]. Wang and colleagues investigated 12 monoclonal antibodies and found that fixed and body size-based dosing perform similarly, with fixed dosing being better for 7 of 12 antibodies . In addition, they investigated 18 therapeutic proteins and peptides and showed that fixed dosing performed better for 12 of 18 in terms of reducing the between-subject variability in pharmacokinetic/pharmacodynamics parameters [Zhang et al 2012].

A fixed dosing approach is preferred by the prescribing community due to ease of use and reduced dosing errors. Given expectation of similar pharmacokinetic exposure and variability, we considered it feasible to switch to fixed dosing regimens. Based on average body WT of 75 kg, a fixed dose of 750 mg Q2W MEDI4736 (equivalent to 10 mg/kg Q2W), 1500 mg Q4W durvalumab (equivalent to 20 mg/kg Q4W) and 75 mg Q4W tremelimumab (equivalent to 1 mg/kg Q4W) is included in the current study.

Fixed dosing of durvalumab and tremelimumab is recommended only for subjects with > 30kg body weight due to endotoxin exposure. Patients with a body weight less than or equal to 30 kg should be dosed using a weight-based dosing schedule.

2. STUDY OBJECTIVE

2.1 Primary objective(s)

Phase Ib Objective:
To determine the **safety** of the combination of Durvalumab (Anti-PDL-1) + Tremelimumab (Anti-CTLA-4) + FOLFOX

Phase II Objectives:
To determine efficacy of the combination of Durvalumab (Anti-PD-L1) + Tremelimumab (Anti-CTLA-4) + FOLFOX in terms of PFS in patients with colorectal MSS disease.

2.2 Secondary objective

Phase II Secondary Objective:

To determine efficacy of the combination of Durvalumab (Anti-PDL1) + Tremelimumab (Anti-CTLA-4) + FOLFOX in terms of response to treatment and overall survival in patients with colorectal MSS disease.

- To determine efficacy of the combination of Durvalumab (Anti-PD-L1) + Tremelimumab (Anti-CTLA-4) + FOLFOX in terms of PFS, response to treatment and overall survival in patients with colorectal MSI disease.

2.3 Exploratory objective(s)

Exploratory Studies :

- To evaluate quality of life at each cycle,
- To determine genetically characterized for MSI status,
- To determine NRAS, KRAS and Braf status,
- To study the immune cells infiltration into the tumor,
- To analyze PD-1, PD-L1, CTLA-4 expression with Ventana assay system
- To determine double labelling of Th1, Th2, Th17, Follicular helper T cells and exhausted T cells,
- To perform identification of tumor-specific mutations,
- To determine candidate of neoantigens and also prediction for proteasomal processing and HLA class I binding will be assess,
- Analyze immune response before and after treatment start,
- To assess local immune response before and after therapy,
- To study evaluation of lymphocyte reactivity to tumor antigens,
- To analyze cytokine production by T cells.

3. STUDY DESIGN

3.1 Overview of study design

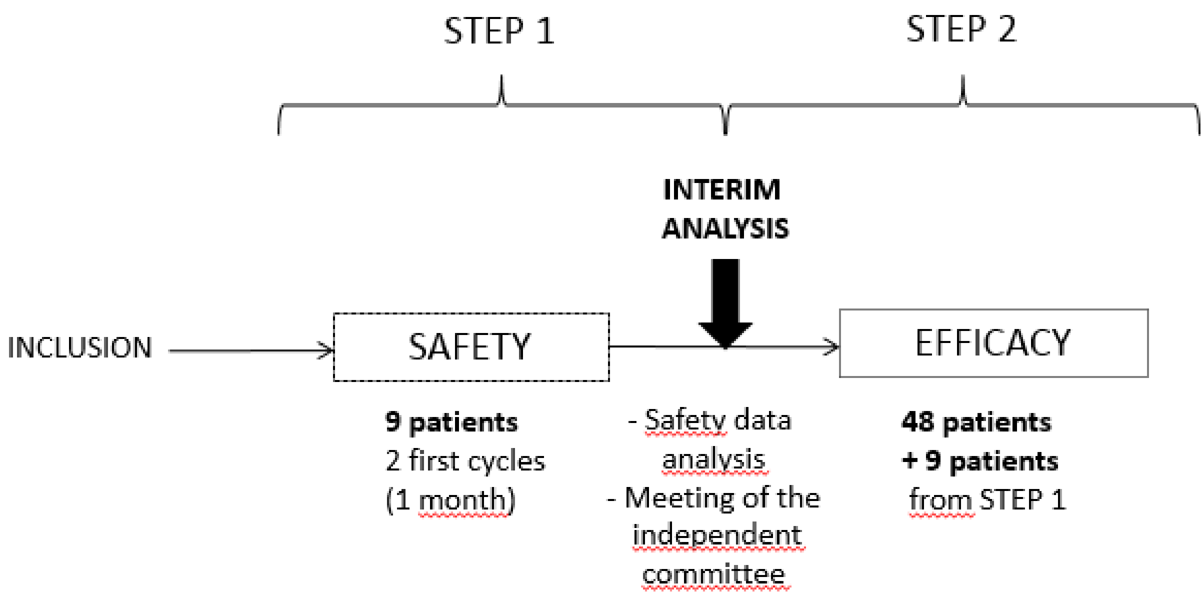

Figure 1: overview of the study design

Study will be performed in 2 step:

- ⇨ **STEP 1** will assess the safety of the combination of Durvalumab 750mg q2w + tremelimumab 75mg q4w + FOLFOX during the 2 first cycles of treatment.
- ⇨ **STEP 2** will assess the efficacy of the combination of Durvalumab 750mg q2w + tremelimumab 75mg q4w + FOLFOX

3.2 Study schema

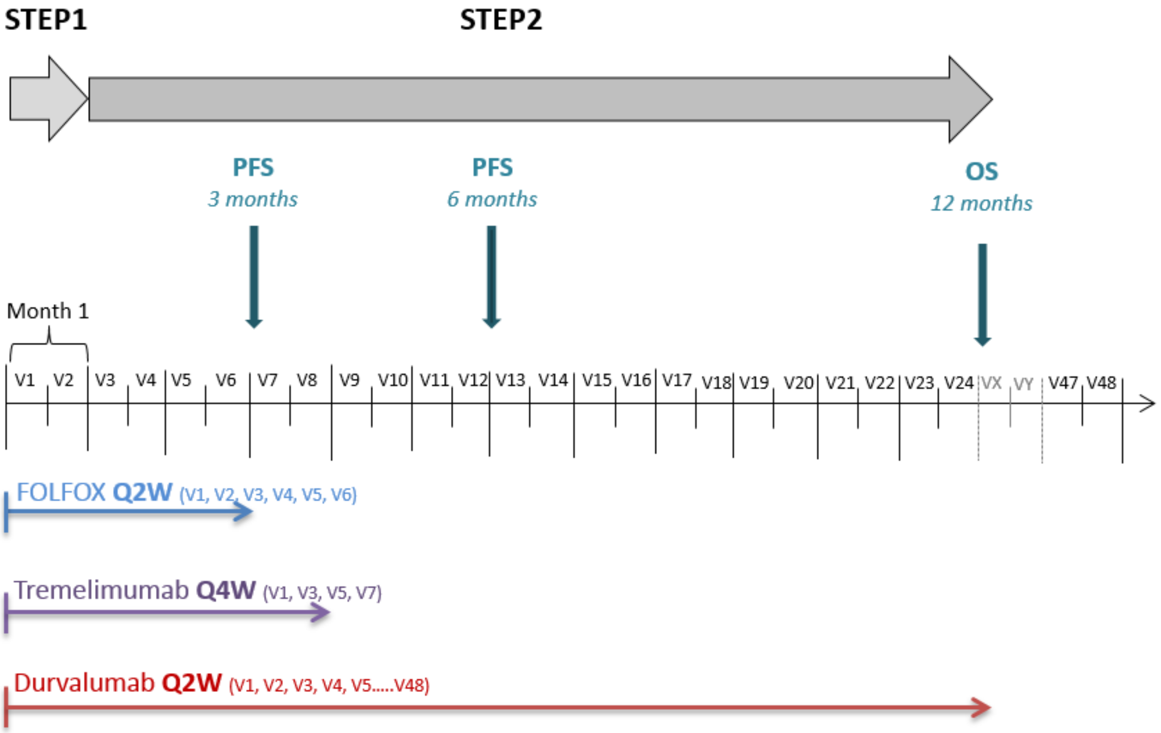

Figure 2: schema of the study

3.3 Study Oversight for Safety Evaluation

The Study could be suspended or ended by the sponsor in concertation with AstraZeneca and if requested by the competent authorities (Ethic committee and the national safety drug agency) for the following reason:

- Frequency and/or severity of unexpected toxicity
- Inadequate patient recruitment
- Insufficient quality of data collection

4. PATIENT SELECTION, ENROLLMENT, RESTRICTIONS, DISCONTINUATION AND WITHDRAWAL

Each patient must meet all of the inclusion criteria (Section 4.1) and none of the exclusion criteria (Section 4.2) for this study. Under no circumstances, will there be exceptions to this rule.

4.1 Inclusion criteria

For inclusion in the study, patients should fulfill the following criteria:

1. Written informed consent and any locally-required authorization obtained from the subject prior to performing any protocol-related procedures, including screening evaluations,

2. Male or female age ≥ 18 years at time of study entry,
3. Performance status of 0 or 1 according to the Eastern Cooperative Oncology Group (ECOG) and World Health Organisation (WHO),
4. Histologically confirmed diagnoses of colorectal cancer with positive mutated KRas or NRas.
5. Patients with metastatic disease
6. First line metastatic disease
   Or
   First line after localized disease treated by local curative treatment, with or without adjuvant chemotherapy by FOLFOX; Reccurence after the last dose of adjuvant chemotherapy should be ≥ 6 months. Previous perioperative chemotherapy for resecable metastasis is not permitted
7. Life expectancy of > 12 weeks,
8. Adequate normal organ and marrow function as defined below:
   - Haemoglobin > 9.0 g/dL
   - Absolute neutrophil count (ANC) > 1.5 x 10^9/L (>1500 per mm^3)
   - Platelet count > 100 x 10^9/L (≥100,000 per mm^3)
   - Serum bilirubin ≤ 1.5 x institutional upper limit of normal (ULN).
   - AST (SGOT)/ALT (SGPT) ≤ 2.5 x institutional upper limit of normal unless liver metastases are present, in which case it must be ≤ 5x ULN
   - PAL ≤ 5 x institutional upper limit of normal unless liver metastases are present, in which case it must be ≤ 20x ULN
   - Albumin > 30g/L
   - Creatinine < 1.5 X institutional upper limit of normal (ULN)
   - Serum creatinine CL>40 mL/min by the Cockcroft-Gault formula (Cockcroft and Gault 1976) or by 24-hour urine collection for determination of creatinine clearance:

	Males	Females
Creatinine CL (mL/min)	Weight (kg) x (140 – Age). / 72 x serum creatinine (mg/dL)	Weight (kg) x (140 – Age) x 0.85 / 72 x serum creatinine (mg/dL)

9 Tumour evaluation (CT scan) in the previous 4 weeks with presence of at least one measurable lesion according to RECIST 1.1 criteria,

10 At least 4 weeks since the last chemotherapy, immunotherapy or other drug therapy and / or radiotherapy,

11 Recovery to grade ≤ 1 from any adverse event (AE) derived from previous treatment according to the criteria of the National Cancer Institute Common Terminology Criteria for Adverse Events (NCI-CTCAE) version 4.0,

12 For principal study: willingness to provide consent for use of archived tissue with sufficient material available for analysis.

For ancillary study: Metastasis should be accessible to performed biopsy

13 Female subjects must either be of non-reproductive potential (ie, post-menopausal by history: ≥60 years old and no menses for ≥1 year without an alternative medical cause; OR history of hysterectomy, OR history of bilateral tubal ligation, OR history of bilateral oophorectomy) or must have a negative serum pregnancy test upon study entry,

14 Patients must be affiliated to a social security system,

15 Subject is willing and able to comply with the protocol for the duration of the study including undergoing treatment and scheduled visits and examinations including follow up.

4.2 Exclusion criteria

Subjects should not enter the study if any of the following exclusion criteria are fulfilled:

1 Involvement in the planning and/or conduct of the study (applies to both AstraZeneca staff and/or staff at the study site). Previous enrolment in the present study,

2 Participation in another clinical study with an investigational product during the last 4 weeks,

3 Any previous treatment with a PD1 or PD-L1 inhibitor, including durvalumab or an anti-CTLA4, including tremelimumab (Unless prior PD1/PD-L1 or CTLA-4 inhibition is a specific entry criterion)

4 History of another malignancy within the 5 previous years with low potential risk for recurrence other than :

- *Adequately treated non-melanoma skin cancer or lentigo maligna without evidence of disease*
- *Adequately treated carcinoma in situ without evidence of disease eg, cervical cancer in situ*

5 Receipt of the last dose of anti-cancer therapy (chemotherapy, immunotherapy, endocrine therapy, targeted therapy, biologic therapy, tumor embolization, monoclonal antibodies, other investigational agent) 28 days prior to the first dose of study drug (14 days prior to the first dose of study drug for subjects who have received prior TKIs (e.g., erlotinib, gefitinib and crizotinib) and within 6 weeks for nitrosourea or mitomycin C).

6 Mean QT interval corrected for heart rate (QTc) ≥470 ms calculated from 3 electrocardiograms (ECGs) using Fridericia's Correction,

7 Current or prior use of immunosuppressive medication within 28 days before the first dose of durvalumab or tremelimumab, with the exceptions of intranasal and inhaled corticosteroids or systemic corticosteroids at physiological doses, which are not to exceed 10 mg/day of prednisone, or an equivalent corticosteroid

8 Any history of hypersensitivity to durvalumab, tremelimumab, FOLFOX or their excipients,

9 Any unresolved toxicity (CTCAE grade >1) from previous anti-cancer therapy. Subjects with irreversible toxicity that is not reasonably expected to be exacerbated by the investigational product may be included (e.g., hearing loss, peripherally neuropathy)

10 Any prior Grade ≥3 immune-related adverse event (irAE) while receiving any previous immunotherapy agent, or any unresolved irAE >Grade 1 ,

11 Active or prior documented autoimmune disease within the past 2 years NOTE: Subjects with vitiligo, Grave's disease, or psoriasis not requiring systemic treatment (within the past 2 years) are not excluded,

12 Active or prior documented inflammatory bowel disease (e.g., Crohn's disease, ulcerative colitis),

13 History of primary immunodeficiency,

14 History of organ transplant that requires use of immunosuppressive,

15 History of allogeneic organ transplant,

16 Uncontrolled intercurrent illness including, but not limited to, ongoing or active infection; Clinically significant cardiovascular disease including: myocardial infarction within 6 months,, symptomatic congestive heart failure, uncontrolled hypertension, unstable angina pectoris, cardiac arrhythmia; history of Mobitz II second degree or third degree heart block without a permanent pacemaker in place, hypotension,; rest limb claudication or ischemia within 6 months; active peptic ulcer disease or gastritis, active bleeding diatheses including any subject known to have evidence of acute or chronic hepatitis B, hepatitis C or human immunodeficiency virus (HIV), or psychiatric illness/social situations that would limit compliance with study requirements or compromise the ability of the subject to give written informed consent,

17 Sever concurrent disease, or co-morbidity that in the judgment of the investigator, would make the patient inappropriate for enrolment,

18 Ongoing treatment with CYP3A4 substrates or regularly taking of grapefruit juice,

19 Known history of active tuberculosis,

20 History of leptomeningeal carcinomatosis,

21 Brain metastases or spinal cord compression,

22 Receipt of live attenuated vaccination within 30 days prior to study entry or within 30 days of receiving durvalumab or tremelimumab

23 Female subjects who are pregnant, breast-feeding or male or female patients of reproductive potential who are not employing an effective method of birth control,

24 Any condition that, in the opinion of the investigator, would interfere with evaluation of study treatment or interpretation of patient safety or study results,

25 Symptomatic or uncontrolled brain metastases requiring concurrent treatment, inclusive of but not limited to surgery, radiation and/or corticosteroids,

26 Subjects with uncontrolled seizures,

27 Female patients who are pregnant or breastfeeding or male or female patients of reproductive potential who are not willing to employ effective birth control from screening to 180 days after the last dose of durvalumab + tremelimumab combination therapy or 90 days after the last dose of durvalumab monotherapy, whichever is the longer time period

28 Known allergy or hypersensitivity to IP or any excipient

29 Patients with tumors that invade major vessels, as shown unequivocally by imaging studies

30 Patients with central lung metastases (i.e within 2 cm from the hilum) that are cavitary as shown unequivocally by imaging studies

31 Patients with any prior history of bleeding related to the current colorectal cancer

32 Patients with a history of gross hemoptysis (bright red blood of ½ teaspoon or more per episode of coughing) ≤ 3 months prior to enrollment

33. Patients with a recurrence delay smaller than 6 months after the adjuvant chemotherapy.

34. Patients with resecable disease

4.3 Withdrawal of Subjects from Study Treatment and/or Study

An individual subject will not receive any further Durvalumab or Tremelimumab products if any of the following occur in the subject in question:

1. Withdrawal of consent or lost to follow-up
2. Adverse event that, in the opinion of the investigator or the sponsor, contraindicates further dosing
3. Subject is determined to have met one or more of the exclusion criteria for study participation at study entry and continuing investigational therapy might constitute a safety risk
4. Pregnancy or intent to become pregnant
5. Any AE that meets criteria for discontinuation
6. Dose-limiting toxicity
7. Grade ≥ 3 infusion reaction
8. Subject noncompliance that, in the opinion of the investigator or sponsor, warrants withdrawal; eg, refusal to adhere to scheduled visits
9. Initiation of alternative anticancer therapy including another investigational agent
10. Confirmation of PD and investigator determination that the subject is no longer benefiting from treatment with durvalumab

Subjects who are permanently discontinued from further receipt of investigational product, regardless of the reason (withdrawal of consent, due to an AE, other), will be identified as having permanently discontinued treatment.
Subjects who are permanently discontinued from receiving investigational product will be followed for safety per Section 10.0 unless consent is withdrawn or the subject is lost to follow-up or enrolled in another clinical study, in these cases, all study procedure will be stopped. All subjects will be followed for survival. Subjects who decline to return to the site for evaluations will be offered follow-up by phone every 4 months as an alternative.

Withdrawal of consent
If consent is withdrawn, the subject will not receive any further investigational product or further study observation.

4.4 Replacement of subjects

Efficacy analyses will be performed in modified ITT, ie including all patients following the major inclusion criteria. Other patients will be replaced (anticipated dropout rate of 10%).

5. INVESTIGATIONAL PRODUCT(S)

5.1 Durvalumab and tremelimumab

The Investigational Products Supply section of AstraZeneca/MedImmune will supply durvalumab and tremelimumab to the investigator as a solution for infusion after dilution.

5.1.1 Formulation/packaging/storage

Durvalumab

Durvalumab will be supplied by AstraZeneca as a 500-mg vial solution for infusion after dilution. The solution contains 50 mg/mL durvalumab, 26 mM histidine/histidine-hydrochloride, 275 mM trehalose dihydrate, and 0.02% (weight/volume) polysorbate 80; it has a pH of 6.0. The nominal fill volume is 10 mL. Investigational product vials are stored at 2°C to 8°C (36°F to 46°F) and must not be frozen. Durvalumab must be used within the individually assigned expiry date on the label.

Tremelimumab

Tremelimumab will be supplied by AstraZeneca as a 400-mg or a 25mg vial solution for infusion after dilution. The solution contains 20 mg/mL of tremelimumab, 20 mM histidine/histidine hydrochloride, 222 mM trehalose dihydrate, 0.02% (w/v) polysorbate 80, and 0.27 mM disodium edetate dihydrate (EDTA); it has a pH of 5.5 and density of 1,034 g/mL. The nominal fill volume is 20 mL.for the 400mg and 1,25mL for the 25mg vial.Investigational product vials are stored at 2°C to 8°C (36°F to 46°F) and must not be frozen. Tremelimumab must be used within the individually assigned expiry date on the label.

5.2 Dose and treatment regimens

5.2.1 Treatment regimens

Durvalumab + tremelimumab

Patient will receive 750mg durvalumab via IV infusion q2w for up to 8 doses/cycles and 75 mg tremelimumab via IV infusion q4w for up to 4 doses/cycles, and then continue 750mg durvalumab q2w starting on Week 16 for up to 20 months (40 doses). Dosing outside the window should be discussed with the Study Physician. Tremelimumab will be administered first. Durvalumab infusion will start approximately 1 hour after the end of tremelimumab infusion. The duration will be approximately 1 hour for each infusion. A 1-hour observation period is required after the first infusion of durvalumab and tremelimumab. If no clinically significant infusion reactions are observed during or after the first cycle, subsequent infusion observation periods can be at the Investigator's discretion

(suggested 30 minutes after each durvalumab and tremelimumab infusion). FOLFOX will be administered last and will be delivered as standard practice q2w.

5.2.2 Duration of treatment and criteria for retreatment

Treatment will be delivered with a maximum of 24 months:

- 4 months with the association Durvalumab + Tremelimumab + FOLFOX (M1 to M4)
- 20 months of maintenance with Durvalumab alone (M5 to M24)

Retreatment is allowed (**once only**) for patients meeting the retreatment criteria below. The same treatment guidelines followed during the initial 24-month (4 months with the association + 20 months maintenance) treatment period will be followed during the retreatment period, including the same dose and frequency of treatments and the same schedule of assessments.
Patients may undergo retreatment in 1 clinical scenarios, described below:

1. Patients who complete the 4 dosing cycles of the combination of durvalumab and tremelimumab portion of the regimen (with clinical benefit per Investigator judgment), but subsequently have evidence of PD during the durvalumab monotherapy portion of the combination regimen, with or without confirmation according to RECIST 1.1, may restart treatment with the combination (FOLFOX + Durvalumab + Tremelimumab).

Before restarting their assigned treatment, the Investigator should ensure that the patient :

- does not have any significant, unacceptable, or irreversible toxicities that indicate continuing treatment will not further benefit the patient.
- Still fulfils the eligibility criteria for this study, to restart folfox, durvalumab and tremelimumab.
- Has not received an intervening systemic anticancer therapy after their assigned treatment discontinuation.
- Has to perform a baseline tumor assessment within 28 days of restarting their assigned treatment; all further scans should occur with the same frequency as during the initial 12 months of treatment (relative to the date of randomization) until study treatment is stopped (maximum of 12 months of further treatment).

During the retreatment period, patients receiving durvalumab + tremelimumab may resume durvalumab dosing at 750mg q2w with 75 mg of tremelimumab q4w for 4 doses each and combined to FOLFOX for 3 months. Patients will then continue with durvalumab monotherapy at 750 mg q2w, until month 24 if no PD is observed.

Treatment through 24 months is at the Investigator's discretion, and the Investigator should ensure that patients do not have any significant, unacceptable, or irreversible toxicity that indicate that continuing treatment will not further benefit the patient. A patient with a confirmed progression receiving durvalumab + tremelimumab cannot continue therapy or obtain retreatment if dosing is ongoing in the combination portion of therapy (**q4w dosing**) and progression occurs in a target lesion that has previously shown a confirmed response.

Patients who AstraZeneca and/or the Investigator determine may not continue treatment will enter follow-up.

5.2.3 Study drug preparation of durvalumab and tremelimumab

Based on average body WT of 75 kg, a fixed dose of 750 mg Q2W durvalumab and 75 mg Q4W tremelimumab (equivalent to 1 mg/kg Q4W) will be administered.

Preparation of durvalumab doses for administration with an IV bag

The dose of durvalumab for administration must be prepared by the Investigator's or site's designated IP manager using aseptic technique. Total time from needle puncture of the durvalumab vial to the start of administration should not exceed:

- 24 hours at 2°C to 8°C (36°F to 46°F)
- 4 hours at room temperature

If in-use storage time exceeds these limits, a new dose must be prepared from new vials. Infusion solutions must be allowed to equilibrate to room temperature prior to commencement of administration.

No incompatibilities between durvalumab and polyvinylchloride or polyolefin IV bags have been observed. Dose of 750 mg durvalumab for patients >30 kg will be administered using an IV bag containing 0.9% (w/v) saline, with a final durvalumab concentration ranging from 1 to 20 mg/mL, and delivered through an IV administration set with a 0.2- or 0.22-µm in-line filter. Remove 30.0 mL of IV solution from the IV bag prior to addition of durvalumab. Next, 30.0 mL of durvalumab (ie, 1500 mg of durvalumab) is added to the IV bag such that final concentration is within 1 to 20 mg/mL (IV bag volumes 100 to 1000 mL). Mix the bag by gently inverting to ensure homogeneity of the dose in the bag.

Patient weight at baseline should be used for dosing calculations unless there is a ≥10% change in weight. Dosing day weight can be used for dosing calculations instead of baseline weight per institutional standard.

For patients <30kg, Calculate the dose volume of durvalumab and tremelimumab and number of vials needed for the subject to achieve the accurate dose.

Durvalumab will be administered at room temperature (approximately 25°C) by controlled infusion via an infusion pump into a peripheral or central vein. Following preparation of durvalumab, the entire contents of the IV bag should be administered as an IV infusion over approximately 60 minutes (±5 minutes), using a 0.2, or 0.22-µm in-line filter. Less than 55 minutes is considered a deviation.

The IV line will be flushed with a volume of IV solution (0.9% [w/v] saline equal to the priming volume of the infusion set used after the contents of the IV bag are fully administered, or complete the infusion according to institutional policy to ensure the full dose is administered and document if the line was not flushed.

Standard infusion time is 1 hour. However, if there are interruptions during infusion, the total allowed time should not exceed 8 hours at room temperature. The table below summarizes time allowances and temperatures.

Durvalumab hold and infusion times

Maximum time from needle puncture to start of administration	4 hours at room temperature, 24 hours at 2°C to 8°C
Maximum time for IV bag infusion, including interruptions	8 hours at room temperature

In the event that either preparation time or infusion time exceeds the time limits outlined in the table, a new dose must be prepared from new vials. Durvalumab does not contain preservatives, and any unused portion must be discarded.

Preparation of tremelimumab doses for administration with an IV bag

The dose of tremelimumab for administration must be prepared by the Investigator's or site's designated IP manager using aseptic technique. Total time from needle puncture of the tremelimumab vial to the start of administration should not exceed:

- 24 hours at 2°C to 8°C (36°F to 46°F)
- 4 hours at room temperature

It is recommended that the prepared final IV bag be stored in the dark at 2ºC-8°C (36°F-46°F) until needed. If storage time exceeds these limits, a new dose must be prepared from new vials. The refrigerated infusion solutions in the prepared final IV bag should be equilibrated at room temperature for about 2 hours prior to administration. Tremelimumab does not contain preservatives and any unused portion must be discarded.

No incompatibilities between tremelimumab and polyvinylchloride or polyolefin IV bags have been observed. Doses of 75 mg tremelimumab for patients >30 kg will be administered using an IV bag containing 0.9% (w/v) saline, with a final tremelimumab concentration ranging from 0.1 mg/mL to 10 mg/mL, and delivered through an IV administration set with a 0.2 μm or 0.22 μm in-line filter. Remove 3.8 mL of IV solution from the IV bag prior to addition of tremelimumab. Next, 3.8 mL of tremelimumab (ie, 75 mg of tremelimumab) is added to the IV bag such that final concentration is within 0.1 mg/mL to 10 mg/mL (IV bag volumes 50 to 500 mL). Mix the bag by gently inverting to ensure homogeneity of the dose in the bag.

Patient weight at baseline should be used for dosing calculations unless there is a ≥10% change in weight. Dosing day weight can be used for dosing calculations instead of baseline weight per institutional standard.

For patients <30 kg, Calculate the dose volume for tremelimumab and number of vials needed for subject to achive the accurate dose.

Tremelimumab will be administered at room temperature (approximately 25°C) by controlled infusion via an infusion pump into a peripheral or central vein. Following preparation of tremelimumab, the entire contents of the IV bag should be administered as an IV infusion over approximately 60 minutes (±5 minutes), using a 0.2, or 0.22-μm in-line filter. Less than 55 minutes is considered a deviation.

The IV line will be flushed with a volume of 0.9% (w/v) saline equal to the priming volume of the infusion set used after the contents of the IV bag are fully administered, or complete the infusion according to institutional policy to ensure the full dose is administered and document if the line was not flushed.

Standard infusion time is 1 hour. However, if there are interruptions during infusion, the total allowed time should not exceed 8 hours at room temperature. The table below summarizes time allowances and temperatures.

Tremelimuab hold and infusion times

Maximum time from needle puncture to start of administration	4 hours at room temperature, 24 hours at 2°C to 8°C
Maximum time for IV bag infusion, including interruptions	8 hours at room temperature

In the event that either preparation time or infusion time exceeds the time limits outlined in the table, a new dose must be prepared from new vials. Tremelimumab does not contain preservatives, and any unused portion must be discarded.

5.2.4 Monitoring of dose administration

Patients will be monitored during and after the infusion with assessment of vital signs at the times specified in the Study Protocol.

In the event of a ≤Grade 2 infusion-related reaction, the infusion rate of study drug may be decreased by 50% or interrupted until resolution of the event and re-initiated at 50% of the initial rate until completion of the infusion. For patients with a ≤Grade 2 infusion-related reaction, subsequent infusions may be administered at 50% of the initial rate. Acetaminophen and/or an antihistamine (eg, diphenhydramine) or equivalent medications per institutional standard may be administered at the discretion of the investigator. If the infusion-related reaction is ≥Grade 3 or higher in severity, study drug will be discontinued.

As with any antibody, allergic reactions to dose administration are possible. Appropriate drugs and medical equipment to treat acute anaphylactic reactions must be immediately available, and study personnel must be trained to recognize and treat anaphylaxis. The study site must have immediate access to emergency resuscitation teams and equipment in addition to the ability to admit patients to an intensive care unit if necessary

5.2.5 Accountability and dispensation

General Accountabilities and each dispensation of durvalumab and tremelimumab should be recorded on the pharmacy drug accountability logs and patient accountability log of the study and should be kept in the pharmacy files. General accountabilities and dispensation of durvalumab to each subject (dose, lot number, dates etc..) will be verified by the clinical research associate during monitoring visit at the pharmacy.

5.2.6 Disposition of unused investigational study drug

The site will account for all investigational study drug dispensed and also for appropriate destruction. Certificates of delivery and destruction must be signed and a destruction certificates should be provided and signed for each durvalumab destruction.

5.3 FOLFOX

Tremelimumab will be administered first, then Durvalumab and then FOLFOX will be administered last and will be delivered as standard practice q2w.

5.3.1 Formulation/packaging/storage

FOLFOX will be supplied by each site as this treatment is already on the market. Each investigational site will be provided by 5mg/mL Oxaliplatin vial (100mg/20mL); 50mg/mL 5-Fluorouracil vial (5000mg/100mL) and Folinic acid 200mg/mL for infusion after dilution. Oxaliplatin, Folinic acid and 5-Fluorouracil are stored between 15°C to 25°C and must not be frozen. Oxaliplatin and 5-Fluorouracil must be used within the individually assigned expiry date on the label.

5.3.2 Product preparation, Doses and treatment regimens administration

Subjects will be administered with FOLFOX in line with normal clinical practice, with a dose and schedule of Oxaliplatin, 85 mg/m2 administered as intravenous infusion over 2 hours in 250 ml dextrose 5% or sterile water for injection concurrently (via a Y-connector) with LV, 400 mg/m2 (400 mg/m2 for form dl or 200 mg/m2 for form l) administered as intravenous infusion over 2 hours, in 250 ml dextrose 5%, or sterile water for injection followed by 5-FU, 400 mg/m2 administered as a bolus injection (intravenous push administered manually in approximately 2 minutes), followed by 5-FU, 2400 mg/m2 administered as a intravenous infusion over 46 hours. Oxaliplatin should always be administered before fluoropyrimidines.

Dose of oxaliplatin, 5-FU and LV will be administered on the basis of milligrams of each drug per square meter of body surface area (BSA) as measured at baseline (mg/m2). The dose of oxaliplatin administered should be as close as possible to the calculated dose and will be limited to a maximum BSA of 2.0 m2. Though the weight of the patient may change throughout the study, BSA will be assumed to stay close to that measured at baseline, i.e. no dose adjustments for changes in body weight will be done unless weight loss alone is considered to be an adverse event of grade 2 or more. Cycle length is 2 weeks comprising approximately 48 hours of infusion and 12 days of rest. It is expected that subjects will receive between 1 and 12 cycles of FOLFOX (6 months).

5.3.3 Monitoring of dose administration

Dose reductions during treatment should be based on adverse events. Oxaliplatin administration does not require hyperhydration. In the event of extravasation, administration must be discontinued immediately. For nausea and vomiting, 5-HT3 antagonists with or without dexamethasone are strongly recommended for oxaliplatin-based chemotherapy. Oxaliplatin must be infused either by peripheral vein or central venous line over 2 hours. The infusion line must be adequately flushed with 5% dextrose solution (D5W) between oxaliplatin infusion and the administration of any other drug. If desired, oxaliplatin can be administered concurrently (via a Y-connector from separate bags and lines) with leucovorin, given as a 2-hour IV infusion in 250 ml dextrose 5%, but should not be administered concurrently with 5-FU.

5.3.4 Accountability and dispensation

General Accountabilities and each dispensation of FOLFOX should be recorded on the pharmacy drug accountability logs and patient accountability log of the study and should be kept in the pharmacy files. General accountabilities and dispensation of FOLFOX to each subject (dose, lot number, dates etc..) will be verified by the clinical research associate during monitoring visit at the pharmacy.

5.3.5 Disposition of unused investigational study drug

The site will account for all FOLFOX study drugs dispensed and also for appropriate destruction. Certificates of delivery and destruction must be signed and a destruction certificates should be provided and signed for each FOLFOX drug destruction.

6. TREATMENT PLAN

6.1 Subject enrollment

6.1.1 Procedures for enrolment

After the patient signed the inform consent form and the investigator has validated all the inclusion exclusion criteria, eligible patient will be included. The principal investigator will perform the enrollment via Tenalea system. In return, the corresponding Centre will receive via email, confirmation of the inclusion of the subject with inclusion number allocated to the patient. In case of problem with the e-randomization, the Data management department of the Centre Georges François Leclerc in charge of the inclusion could be contacted. Contact details are summarized below:

Unité de Méthodologie, Biostatistiques et Data Management
From Monday to Friday from 9A.M to 6 P.M
Phone: +33(0)3.80.73. 77. 84 - Fax : +33 (0)3.80. 80 73 77 34

6.1.2 Procedures for handling subjects incorrectly enrolled

Subjects who are incorrectly enrolled and have not completed 2 Cycles of treatment will be replaced.

Subjects who have been enrolled, in spite of not meeting all the inclusion/exclusion criteria will not be replaced, data analyses will take them into account (both intention to treat and per protocol analysis).

To limit this incorrectly enrolled patients, inclusion criteria will be checked using an inclusion base before inclusion in the escalation dose phase and before inclusion in the expansion cohort.

6.1.3 Procedures for handling subjects lost of follow up or untreated enrolled

If a patient is lost of follow up, died, and can not received at least 2 cycles of treatment combination he will be replaced.
If a patient receives less than 2 cycles of treatment combination, he will be replaced.
All patients with at least 2 cycles of treatment will be included in the study and analysis. Subjects who discontinue from the study and received two cycles of treatment combination will not be replaced. Toxicity analyses will be performed using the dose level in which the patient has been enrolled. For the escalation dose phase, if a patient can analyses for preliminary efficacy in the expansion cohort will be performed both in intention to treat and per protocol. Noticed that the sample size calculation anticipate a drop out rate of 15%.

6.2 Dose Modification and Toxicity Management

6.2.1 Durvalumab and tremelimumab

For adverse events (AEs) that are considered at least partly due to administration of durvalumab the following dose adjustment guidance may be applied:

- Treat each of the toxicities with maximum supportive care (including holding the agent suspected of causing the toxicity where required).
- If the symptoms promptly resolve with supportive care, consideration should be given to continuing the same dose of durvalumab or tremelimumab along with appropriate continuing supportive care. If medically appropriate, dose modifications are permitted for durvalumab and tremelimumab (see Appendix 1).
- All dose modifications should be documented with clear reasoning and documentation of the approach taken.

Investigators should exercise clinical judgment in managing actual patients alongside the guidelines presented in the protocol(s). An event that exhibits rapid progression and/or the likelihood for high morbidity/mortality requires that clinical judgment be exercised above and beyond toxicity management guidelines to ensure that treatment is optimally tailored to any given patient's specific case. For example, the general principles outlined in the toxicity management guidelines describe prompt initiation of corticosteroids for both Grade 2 events (that have persisted for 4-5 days) and Grade 3-4 events; clinical judgment applied to this baseline guidance for an event that exhibits rapid progression and/or the likelihood for high morbidity/mortality – such as myocarditis – would warrant prompt initiation of high-dose corticosteroids without delay even for grade 2 cases. Similarly, clinical judgment for patients with suspected myocarditis should lead investigators to obtain a cardiology consult and institute a thorough diagnostic work-up (that includes exclusion of other alternate causes such as infection), and the appropriate management that includes discontinuing drug (permanently if biopsy-proven immune-mediated myocarditis) and, as already noted, the prompt use of steroids or other immunosuppressives

In addition, there are certain circumstances in which durvalumab or tremelimumab should be permanently discontinued.

Following the first dose of durvalumab or tremelimumab, subsequent administration of durvalumab or tremelimumab can be modified based on toxicities observed (see Appendix 1). Dose reductions are not permitted.

Based on the mechanism of action of durvalumab or tremelimumab leading to T-cell activation and proliferation, there is the possibility of observing immune related Adverse Events (irAEs) during the conduct of this study. Potential irAEs include immune-mediated enterocolitis, dermatitis, hepatitis, and endocrinopathies. Subjects should be monitored for signs and symptoms of irAEs. In the absence of an alternate etiology (e.g., infection or PD) signs or symptoms of enterocolitis, dermatitis, hepatitis, and endocrinopathy should be considered to be immune-related.

Dose modification recommendations and toxicity management guidelines for immune-mediated reactions, for infusion-related reactions, and for non-immune-mediated reactions are detailed in Appendix 1.

In addition, management guidelines for adverse events of special interest (AESIs) are detailed in Section 10.1.3. All toxicities will be graded according to NCI CTCAE v4.03

6.2.2 FOLFOX (5-Fluorouracil + oxaliplatin)

All toxicities will be recorded on the flow sheet and appropriately graded according to CTCAE Version 4.03 criteria, as regard to severity and whenever possible in relationship to drug or disease. Guidelines to be followed in the case of a deviation from treatment schedule (i.e. treatment interruption or dose modification due to an adverse event) are specific for each treatment regimen, and are described in the following subsections.
- For toxicities which are considered by the investigator unlikely to develop into serious or life threatening events (e.g. alopecia, altered taste etc.), treatment will be continued at the same dose without reduction or interruption. In addition, no dose reductions or interruptions will be required for anaemia (non-haemolytic) as it can be satisfactorily managed by transfusions

- If, in the opinion of the investigator, a toxicity is considered to be due solely to one drug (e.g. neurotoxicity due to oxaliplatin), the dose of the other drugs does not require modification.

- Dose modifications for isolated abnormal hematologic lab values will be based on haematological parameters at start of a treatment cycle. There is no scheduled sampling during a treatment cycle and thus, no scheduled collection of nadir values.

Oxaliplatin-induced neurotoxicity:

Peripheral neuropathy:
Oxaliplatin is consistently associated with two types of peripheral neuropathy: paresthesia and dysesthesia of the hands and feet (chronic), and peri-oral region (early onset). Patients treated with oxaliplatin in this study will be counselled to avoid cold drinks and exposure to cold water or air, especially for 3 to 5 days following oxaliplatin administration. For peripheral neuropathy, the scale reported in the following table will be used to determine dose adjustments.

Laryngopharyngeal dysesthesia:

An unusual laryngopharyngeal dysesthesia, a sensation of loss of breathing (acute respiratory distress) without any objective evidence of respiratory distress (hypoxia, laryngospasm, or bronchospasm), also has been observed. This neurotoxicity may be induced or exacerbated upon exposure to cold. If a patient develops laryngopharyngeal dysesthesia, the patient's oxygen saturation should be evaluated via a pulse oximeter and, if normal, reassurance, a benzodiazepine or other anxiolytic agent should be considered and the patient should be observed in the clinic until the episode has resolved. The oxaliplatin infusion may then be continued at 1/3 of the previous rate. Because this syndrome may be associated with the rapidity of oxaliplatin infusion, subsequent doses of oxaliplatin should be administered as 6-hour infusions (instead of the normal 2-hour infusion). Patients on oxaliplatin should not receive cold drinks or ice chips on Day 1 of each cycle as this may exacerbate oral or throat dysesthesia, as well as laryngopharyngeal dysesthesia. Administration of prophylactic medication such as Mg++, Ca++ infusions or others is at the discretion of the investigator (1g Ca-gluconate and 1g Mg-sulfate in 100 ml D5W over 15 to 30 min immediately before and after oxaliplatin). Note: administration of 5-FU must be postponed until the end of oxaliplatin infusion. If oxaliplatin has to be stopped because of neurotoxicity, 5-FU and LV or capecitabine should be administered as per protocol and for the duration required by randomization, i.e. 3 or 6 months.

Allergic reactions:

For Grade 1 or 2 acute hypersensitivity reactions, no dose modification of oxaliplatin is required if, in the investigator’s opinion, it is in the patient’s best interest to continue. Pre-medication with dexamethasone 20 mg IV, diphenhydramine 50 mg IV, and one of the following: cimetidine 300 mg IV, ranitidine 50 mg IV, or famotidine 20 mg IV 30 minutes prior to study drug administration is suggested. If an allergic reaction persists into the next cycle, administer 50 mg dexamethasone PO 12 hours and 6 hours prior to administration of oxaliplatin. For Grade 3 or 4 acute hypersensitivity reactions, treatment with oxaliplatin should be discontinued.

Pulmonary fibrosis:

In the case of respiratory symptoms indicative of pulmonary fibrosis such as nonproductive cough, dyspnea, crackles, rales, hypoxia, tachypnea or radiological pulmonary infiltrates, oxaliplatin should be interrupted pending further investigation. If interstitial pulmonary fibrosis is confirmed, permanently discontinue oxaliplatin.

Neurologic Toxicity Scale – Oxaliplatin Dose

TOXICITY	Grade	Duration of Toxicity		
		**1-7 Days**	**> 7 Days**	**Persistent between cycles**
Paresthesia/dysesthesia that do not interfere with function	1	No dose reduction	No dose reduction	75 mg/m^2
Paresthesia/dysesthesia, interfering with function, but not activities of daily living (ADL)	2	No dose reduction	75 mg/m^2	Stop treatment until recovery (grade 0 or 1)
Paresthesia/dysesthesia with pain or with functional impairment that also interfere with ADL	3	75 mg/m^2	Stop treatment until recovery (grade 0 or 1)	Stop treatment until recovery (grade 0 or 1)
Paresthesia/dysesthesia that are disabling or life-threatening	4	Top treatment permanently	Top treatment permanently	Top treatment permanently
ACUTE: (during or after the 2-infusion) laryngopharyngeal dysesthesia		Increase duration of next infusion to 6 hours ± benzodiazepines	Not applicable	Not applicable

FOLFOX induced Toxicity (other than neurotoxicity)

Haematological:

Based on the most severe toxicity experienced since the last treatment, the following dose modifications should be used for haematological toxicities.
FOLFOX is known to increase in the risk of febrile neutropenia (10-20 %), G-CSF may be used depending on the patient risk factors (cf EORTC recommendation https://www.sciencedirect.com/science/article/pii/S0959804906003911) .

Haematological toxicity scale – Oxaliplatine + 5-FU Dose adjustement

TOXICITY	Grade	5-FU bolus	5-FU infusion	Oxaliplatin
Hemoglobin	All	No dose reduce	No dose reduce	No dose reduce
Neutropenia[b]	3	200 mg/m²	200 mg/m²	75 mg/m²
Neutropenia	4	0	200 mg/m²	75 mg/m²
Febrile Neutropenia[a]	≥ 3	0	200 mg/m²	75 mg/m²
Thrombocytopenia[b]	≥ 3	200 mg/m²	200 mg/m²	75 mg/m²

[a]Febrile Neutropenia Grade 3=ANC<1.0X10⁹/L with fever 38.5°C
[b]Dose reduction to applied at the second occurrence of persisting Grade 2 haematological toxicity leading to an extended rest period of ≥ 2 weeks
Supress 5FU bolus if neutropenia or thrombocytopenia grade 3-4 occurrence after first dose reduction to 200mg/m²
The dose of leucovorin should remain identical

Non-haematological
Based on the most severe toxicity experienced since the last treatment, the following dose modifications should be used for non-haematological toxicities. The rest period should be extended until all non-haematological toxicities have subsided to Grade 1 or less, except increased bilirubin and ALAT which must recover to Grade 1 or baseline Grade, whichever is higher.

Nausea and vomiting:
For Grade 3 nausea and/or vomiting that occurs with suboptimal antiemetic therapy, treatment should be continued for the next course with an effective anti-emetic treatment and without dose modification. The administration of 5-HT3 antagonists (granisetron, ondansetron or variants) with corticosteroids (e.g. dexamethasone) is recommended for prevention and treatment of oxaliplatin-induced emesis.

Diarrhea:
If Grade 3 or 4 diarrhea occurs at any time, the doses should be reduced according to Table below. After Grade 3 or 4 diarrhea, the patient must have recovered to Grade 1 or less before treatment can be re-initiated.

Stomatitis:
After Grade 3 or 4 stomatitis, the doses should be reduced according to table below. The patient must have recovered to Grade 1 or less before treatment can be re-initiated.

Cardiac toxicity:

For Grade ≥ 2 cardiac toxicity which is attributable to 5-FU, patients will be permanently discontinued from 5-FU/LV therapy.

Gastro-intestinal ulceration and bleeding:

For Grade ≥ 2 gastro-intestinal toxicity which is attributable to 5-FU, treatment will be held until recovery to Grade ≤ 1.

Skin toxicity:

Treatment will be held for Grade 3 or 4 toxicity until recovery to Grade ≤ 1. Treatment may be withheld to allow for recovery. The extended rest period should not exceed 3 weeks from the scheduled administration. If the patient does not recover to Grade ≤ 1 in this timeframe, he/she will be taken off treatment.

Non haematological toxicity scale – Oxaliplatine + 5-FU dose adjustment

TOXICITY	Grade	5-FU bolus	5-FU infusion	Oxaliplatin
Allergic reactions	≥ 3	No dose reduce	No dose reduce	Stop treatment permanently
Respiratory symptoms indicative of pulmonary fibrosis	any	No dose reduce	No dose reduce	Interrupt treatment and investigate cause of symptoms
Interstitial pulmonary fibrosis not present at baseline	any	No dose reduce	No dose reduce	Stop treatment permanently
Nausea and/or Vomiting	≥ 3	200 mg/m^2	2000 mg/m^2	75 mg/m^2
Diarrhea	≥ 3	200 mg/m^2	2000 mg/m^2	75 mg/m^2
Stomatitis	3	200 mg/m^2	2000 mg/m^2	No dose reduce
Stomatitis	4	0	2000 mg/m^2	75 mg/m^2
Cardiac toxicity (HF syndrome)	≥ 3	Stop treatment permanently	Stop treatment permanently	Stop treatment permanently
Skin toxicity (HF syndrome)	≥ 3	200 mg/m^2	2000 mg/m^2	No dose reduce
The dose of leucovorin should remain identical				

6.2.3 Toxicity management guidelines for combination treatment regimen

Combination of Durvalumab, tremelimumab and FOLFOX has never been evaluated. Any potential overlapping toxicity may occur following treatment with this combination and will be difficult to assess. Toxicity will not be manage individually but will be management according to the guidelines described in section 6.2.1 and 6.2.2 and both treatments will be adjusted simultaneously.

7. RESTRICTIONS DURING THE STUDY AND CONCOMITANT TREATMENT(S)

7.1 Restrictions during the study

Females of childbearing potential who are sexually active with a non-sterilized male partner must use 2 methods of effective contraception (Table 1) from the time of screening and must agree to continue using such precautions for 180 days after the last dose of durvalumab + tremelimumab combination therapy or 90 days after the last dose of durvalumab monotherapy, whichever is the longer time period; cessation of birth control after this point should be discussed with a responsible physician. Periodic abstinence, the rhythm method, and the withdrawal method are not acceptable methods of birth control.

- Females of childbearing potential are defined as those who are not surgically sterile (ie, bilateral tubal ligation, bilateral oophorectomy, or complete hysterectomy) or post-menopausal (defined 12 months with no menses without an alternative medical cause).

- Non-sterilized males who are sexually active with a female partner of childbearing potential must use 2 acceptable methods of effective contraception (see Table 1) from screening through 180 days after receipt of the final dose of durvalumab + tremelimumab combination therapy or 90 days after receipt of the final dose of durvalumab monotherapy, whichever is the longer time period.

Restrictions relating to concomitant medications are described in Section 7.2.

Table 1. Effective methods of contraception (2 methods must be used)

Barrier methods	Intrauterine device methods	Hormonal methods
Male condom plus spermicide	Copper T	Implants
Cap plus spermicide	Progesterone T[a]	Hormonal shot or injection
Diaphragm plus spermicide	Levonorgestrel-releasing intrauterine system (eg, Mirena®)[a]	Combined pill
		Minipill
		Patch

[a] This is also considered to be a hormonal method.

Blood donation

Subjects should not donate blood while participating in this study or for at least 90 days following the last infusion of durvalumab or tremelimumab or until the time specified in the prescribing information of FOLFOX.

7.2 Concomitant treatment(s)

The Principal Investigator must be informed as soon as possible about any medication taken from the time of screening until the end of the clinical phase of the study (final study visit). Any concomitant medication(s), including herbal preparations, taken during the study will be recorded in the CRF.

Restricted, prohibited, and permitted concomitant medications are described in the following tables. Refer to Section 6.6 for guidance on management of IP-related toxicities.

7.2.1 Permitted concomitant medications

Investigators may prescribe concomitant medications or treatments (e.g., acetaminophen, diphenhydramine) deemed necessary to provide adequate prophylactic or supportive care except for those medications identified as “excluded” as listed in Section 7.2.2.

7.2.2 Excluded Concomitant Medications

The following medications are considered exclusionary during the study.

1. Any investigational anticancer therapy other than the protocol specified therapies (Durvalumab, tremelimumab and FOLFOX are allowed)

2. Any concurrent chemotherapy, radiotherapy (except palliative radiotherapy), immunotherapy, biologic or hormonal therapy for cancer treatment, Concurrent use of hormones for noncancer-related conditions (e.g., insulin for diabetes and hormone replacement therapy) is acceptable. Local treatment of isolated lesions for palliative intent is acceptable (e.g., by local surgery or radiotherapy)

3. Immunosuppressive medications including, but not limited to systemic corticosteroids at doses not exceeding 10 mg/day of prednisone or equivalent, methotrexate, azathioprine, and TNF-α blockers. Use of immunosuppressive medications for the management of investigational product-related AEs or in subjects with contrast allergies is acceptable. In addition, use of inhaled and intranasal corticosteroids is permitted. <<A temporary period of steroids will be allowed for different indications, at the discretion of the principal investigator (e.g., chronic obstructive pulmonary disease, radiation, nausea, etc).>>

4. Live attenuated vaccines within 30 days of durvalumab and tremelimumab dosing (ie, 30 days prior to the first dose, during treatment with durvalumab and tremelimumab for 30 days post discontinuation of durvalumab and tremelimumab. Inactivated vaccines, such as the injectable influenza vaccine, are permitted.

5. Patient with large blood vessel invasion are at higher risk for bleeding and any agents affecting platelet function/coagulation should be avoided (Aspirin, NSAIDS…)

Table 2. Prohibited and Rescue Medications

Prohibited medication/class of drug:	Usage:
Additional investigational anticancer therapy concurrent with those under investigation in this study	Should not be given whilst the patient is on IP treatment
mAbs against CTLA-4, PD-1, or PD-L1	Should not be given whilst the patient is on IP treatment through 90 days after the last dose of IP.
Any concurrent chemotherapy, local therapy (except palliative radiotherapy for non-target lesions, eg, radiotherapy, surgery, radiofrequency ablation), biologic therapy, or hormonal therapy for cancer treatment	Should not be given whilst the patient is on IP treatment (including SoC). (Concurrent use of hormones for non-cancer-related conditions [eg, insulin for diabetes and hormone replacement therapy] is acceptable.)
Immunosuppressive medications, including, but not limited to, systemic corticosteroids at doses exceeding 10 mg/day of prednisone or its equivalent, methotrexate, azathioprine, and tumor necrosis factor α blockers	Should not be given whilst the patient is on IP treatment (including SoC). (Use of immunosuppressive medications for the management of IP-related AEs or in patients with contrast allergies is acceptable. In addition, use of inhaled, topical, and intranasal corticosteroids is permitted.
Live attenuated vaccines	Should not be given through 30 days after the last dose of IP (including SoC) during the study

Rescue/supportive medication/class of drug:	Usage:
Concomitant medications or treatments (eg, acetaminophen or diphenhydramine) deemed necessary by the Investigator to provide adequate prophylactic or supportive care, except for those medications identified as “prohibited” as listed above	To be administered as prescribed by the Investigator
Best supportive care (including antibiotics, nutritional support, growth factor support, correction of metabolic disorders, optimal symptom control, and pain management [including palliative radiotherapy, etc])	Should be used when necessary for all patients
Best supportive care (including antibiotics, nutritional support, growth factor support, correction of metabolic disorders, optimal symptom control, and pain management [including palliative radiotherapy, etc])	Should be used when necessary for all patients

8. STUDY PROCEDURES

8.1 Schedule of study procedures

Before study entry, throughout the study, and following study drug discontinuation, various clinical and diagnostic laboratory evaluations are outlined. The purpose of obtaining these detailed measurements is to ensure adequate safety and tolerability assessments. Clinical evaluations and laboratory studies may be repeated more frequently if clinically indicated. The Schedules of Assessments during the screening and treatment period is provided following the Protocol Synopsis

8.1.1 Screening Phase

Screening procedures will be performed up to 28 days before Day 1, unless otherwise specified. All subjects must first read, understand, and sign the IRB/REB/IEC-approved ICF before any study-specific screening procedures are performed. After signing the ICF, completing all screening procedures, and being deemed eligible for entry, subjects will be enrolled in the study. Procedures that are performed prior to the signing of the ICF and are considered standard of care may be used as screening assessments if they fall within the 28-day screening window.

The following procedures will be performed during the Screening Visit:

- Informed Consent
- Review of eligibility criteria
- Medical history and demographics
- Complete physical exam
- ECOG Performance Status
- Vitals signs, weight and height
- 12-lead ECG (in triplicate [2-5 minutes apart])
- Review of prior/concomitant medications
- Imaging by CT scan
- Tumor Biopsy with known mutational status (Kras/Nras/Braf – MSS/MSI). If initial biopsy (initial tumor or metastasis) exist with sufficient material for analysis and if patient agree to provide consent to use archived tissue, no new biopsy should be performed.
  If no material exist, a new biopsy should be performed.
- Clinical laboratory tests for:
  - Hematology (see Table 3)
  - Clinical chemistry (see Table 4)
  - TSH
  - Coagulation (PT, PTT, INR)
  - Creatinine Clearance
  - Serum pregnancy test (for women of childbearing potential only)
  - Hepatitis serologies
  - Urinalysis (see Table 5)

8.1.2 Treatment Phase

Procedures to be conducted during the treatment phase of the study are presented in the Schedule of Assessments table 1. Screening procedures performed within J28 to J-1 of Cycle 1 Day 1.

- Complete physical exam
- ECOG Performance Status
- Weight
- Vitals signs to be performed before infusion and should also be repeated 30mn after tremelimumab and durvalumab infusion
- 12-lead ECG if clinically indicated
- Review of prior/concomitant medications
- Imaging by CT scan at Cycle 6, 12 and 18
- Clinical laboratory tests scheduled as described in the schedule of assessment:
    - Hematology (see Table 3)
    - Clinical chemistry (see Table 4)
    - TSH
    - Coagulation (PT, PTT, INR)
    - Creatinine Clearance
    - Serum pregnancy test (for women of childbearing potential only) and if clinically indicated
    - Hepatitis serologies
    - Urinalysis (see Table 5)

8.1.3 End of Treatment

End of treatment is defined as the last planned dosing visit within the 24-month dosing period corresponding to Cycle 48. For subjects who discontinue durvalumab or tremelimumab prior to 12 months, end of treatment is considered the last visit where the decision is made to discontinue treatment. All required procedures may be completed within ± 7 days of the end of treatment visit. Repeat disease assessment is not required if performed within 28 days prior to the end of treatment visit.

Procedures to be conducted at the end of treatment visit are listed bellow:

- Complete physical exam
- ECOG Performance Status
- Vitals signs, weight
- 12-lead ECG
- Review of prior/concomitant medications
- Imaging by CT scan
- Clinical laboratory tests scheduled as described in the schedule of assessment:
    - Hematology (see Table 3)
    - Clinical chemistry (see Table 4)
    - TSH

- Coagulation (PT, PTT, INR)
- Creatinine Clearance
- Serum pregnancy test (for women of childbearing potential only) if clinically indicated
- Hepatitis serologies
- Urinalysis (see Table 5)

All subjects will be followed for survival until the end of the study regardless of further treatments, or until the sponsor ends the study.

8.1.4 Explorative study

Procedures to be conducted for the exploratory study are presented in the Schedule of Assessments on table II. Before any procedure, patients should consent to participate to the exploratory study by signing the informed consent form related to the explorative studies.

8.2 Description of study procedures

8.2.1 Medical history and physical examination, electrocardiogram, weight and vital signs

Findings from medical history (obtained at screening) and physical examination shall be given a baseline grade according to the procedure for AEs. Increases in severity of pre-existing conditions during the study will be considered AEs, with resolution occurring when the grade returns to the pre-study grade or below.

Physical examinations will be performed on study days noted in the Schedule of Assessments.

8.2.2 Physical examination

Physical examinations will be performed according to the assessment schedule. Full physical examinations will include assessments of the head, eyes, ears, nose, and throat and the respiratory, cardiovascular, GI, urogenital, musculoskeletal, neurological, dermatological, hematologic/lymphatic, and endocrine systems. Height will be measured at Screening only. Targeted physical examinations are to be utilized by the Investigator on the basis of clinical observations and symptomatology. Situations in which physical examination results should be reported as AEs are described in Section 10

8.2.3 Electrocardiograms

Resting 12-lead ECGs will be recorded at screening and as clinically indicated throughout the study. ECGs should be obtained after the patient has been in a supine position for 5 minutes and recorded while the patient remains in that position.

At Screening, a single ECG will be obtained on which QTcF must be <470 ms.

In case of clinically significant ECG abnormalities, including a QTcF value >470 ms, 2 additional 12-lead ECGs should be obtained over a brief period (eg, 30 minutes) to confirm the finding.

Situations in which ECG results should be reported as AEs are described in Section 10.0.

8.2.4 Vital signs

Vital signs (blood pressure [BP], pulse, temperature, and respiration rate) will be evaluated according to the assessment schedules.

On infusion days, patients receiving durvalumab + tremelimumab treatment will be monitored during and after infusion of IP as presented in the bulleted list below.

Supine BP will be measured using a semi-automatic BP recording device with an appropriate cuff size, after the patient has rested for at least 5 minutes. BP and pulse will be collected from patients receiving durvalumab + tremelimumab treatment before, during, and after each infusion at the following times (based on a 60-minute infusion):

Prior to the beginning of the infusion (measured once from approximately 30 minutes before up to 0 minutes [ie, the beginning of the infusion]).

Approximately 30 minutes during the infusion (**halfway** through infusion).

At the end of the infusion (approximately 60 minutes ±5 minutes).

A 1-hour observation period is required after the first infusion of durvalumab and tremelimumab.

If no clinically significant infusion reactions are observed during or after the first cycle, subsequent infusion observation periods can

be at the Investigator's discretion (suggested 30 minutes after each durvalumab and tremelimumab infusion).

If the infusion takes longer than 60 minutes, then BP and pulse measurements should follow the principles as described above or be taken more frequently if clinically indicated. The date and time of collection and measurement will be recorded on the appropriate eCRF. Additional monitoring with assessment of vital signs is at the discretion of the Investigator per standard clinical practice or as clinically indicated.

Body weight is also recorded along with vital signs.

Situations in which vital signs results should be reported as AEs are described in Section 10.3. A complete physical examination will be performed and will include an assessment of the following (as clinically indicated): general appearance, respiratory, cardiovascular, abdomen, skin, head and neck (including ears, eyes, nose and throat), lymph nodes, thyroid, musculo-skeletal (including spine and extremities), genital/rectal, and neurological systems and at screening only, height.

8.2.5 Clinical laboratory tests

The following clinical laboratory tests will be performed (see the Schedule of Assessments)

- Coagulation parameters: Activated partial thromboplastin time and International normalised ratio to be assessed at baseline and as clinically indicated
- Pregnancy test (female subjects of childbearing potential only)
    - Urine human chorionic gonadotropin
    - Serum beta-human chorionic gonadotropin (at screening only)
- Thyroid Stimulating Hormone
    - free T3 and free T4 only if TSH is abnormal
- Other laboratory tests
    - Hepatitis A antibody, hepatitis B surface antigen, hepatitis C antibody
    - HIV antibody

Table 3. Hematology Laboratory Tests

Basophils	Mean corpuscular volume
Eosinophils	Monocytes
Hematocrit	Neutrophils
Hemoglobin	Platelet count
Lymphocytes	Red blood cell count
Mean corpuscular hemoglobin	Total white cell count
Mean corpuscular hemoglobin concentration	

Table 4. Clinical chemistry (Serum or Plasma) Laboratory Tests

Albumin	Glucose
Alkaline phosphatase	Lactate dehydrogenase
Alanine aminotransferase	Lipase
Amylase	Magnesium
Aspartate aminotransferase	Potassium
Bicarbonate	Sodium
Calcium	Total bilirubin[a]

Table 4. Clinical chemistry (Serum or Plasma) Laboratory Tests

Albumin	Glucose
Chloride	Total protein
Creatinine	Urea or blood urea nitrogen, depending on local practice
Gamma glutamyltransferase[b]	Uric acid

[a] If Total bilirubin is ≥2xULN (and no evidence of Gilbert's syndrome) then fractionate into direct and indirect bilirubin
[b] At baseline and as clinically indicated

Table 5. Urinalysis Tests[a]

Bilirubin	pH
Blood	Protein
Glucose	Specific gravity
Ketones	Colour and appearance

[a] Microscopy should be used as appropriate to investigate white blood cells and use the high power field for red blood cells

8.2.6 Patient reported outcomes (PRO) - QLQ-C30 and QLQ-CR29

The PRO questionnaires should be administered and completed at the clinic in accordance with the study plan in the schedule of events of ancillary studies. Patients must complete the QLQ-C30 and QLQ-CR29 at the same time in any order.

The instruction for completion is as follow:

- It must be completed by the patient
- It must be completed by the patient before any investigations or procedures are performed at the clinic
- Only one answer for each question
- The patients should not receive help from family, friend or clinical staff to answer the questionnaires.

8.3 Biological sampling procedures

8.3.1 Biopsy

8.3.1.1 Principal study (Exome analysis and immunohistochemistry)

At screening, tissue samples from initial tumor or metastasis will be use for Exome analysis and immunohistochemistry. Archived tissue could be used if patient consent and if sufficient material for analysis is available.

- For exome analysis: 10 X 5µm thick unstained section from FFPE bloc + 1 HES blade encircling the tumor zone and estimate of the tumor cell content of the fringed area.

- For immunohistochemistry: 5 to 11 X 4µm thick unstained section from FFPE bloc

If no sufficient material is available to perform exome and immunohistochemistry, meaning a minimum of 16 to 21 unstained section in the existing block, patients will be asked to undergo a new biopsy in the context of the principal study if considered clinically appropriate by their treating physician.

⇨ **Existing archived tissue samples**

If initial biopsies samples (initial tumor or metastasis) already exist, they should be retrieving from the Bio-Bank storage location. These blocks should undergo quality review, prior to evaluation or shipment. When submitting sample for PD-L1 testing and for whole exome and RNAseq analysis, the recommendation is to ship the block for sectioning to occur at the central laboratory at Dijon. Blocks should be shipped containing enough material to be provided to allow a minimum of 11 sections of 5 micron thick for RNAseq analysis and 5-11 sections of 4µm thick each for immunohistochemistry. A total of 16-21 sections should be performed and send.

Where it is not possible or indicated to ship the block to the central laboratory in Dijon, unstained slides should be prepared from the paraffin-embedded tumor sample block (described below) prior to evaluation or shipment.

1) **10 x 5 micron (µm) thick, unstained** from initial tumor tissues or metastasis should be provided for whole exome analysis and RNAseq + **1 HES blade** encircling the tumor zone and an estimate of the tumor cell content of the fringed area. This sample will allow to:

- To determine genetically characterized for MSI status using classically determination of microsatellite instability using multiplex PCR.

- To determine NRAS, KRAS and Braf status using allelic discrimination and CIMP phenotype will be determined using bisulfitation and analysis of CpG Methylator specific marker using PCR.

- To perform identification of tumor-specific mutations, whole-exome sequencing of tumor cells and autologous healthy tissue (frozen sample) will be realized. After DNA extraction, exon capture will be performed with the use of the SureSelect Human All Exon 50-Mb kit. Enriched exome libraries will be sequenced on the local NextSeq (Illumina) platform to provide mean exome coverage of more than 100x. Only non-synonymous and indel mutations will be retained for final genomic analyses.

- We will determine candidate of neoantigens: For each case, to predict potential neoantigens from the set of non-synonymous and indel mutations. Subsequently, predictions for proteasomal processing and HLA class I binding will be performed on stretches of amino acid sequences that contained non synonymous mutations, using the NetChop Cterm3.0 and NetMHC3.2 algorithms.The number of non-synonymous mutations and candidate neoantigens will be correlated with quantitative evaluation of each immune population, response to therapy (radiological) and patients survival (DFS & OS).

- To have a more view of the local immune response RNAseq will be performed on biopsy before and after therapy and the immune contexture will be assessed in this RNA-Seq using a strategy of determination of the expression of immune metagene with normalization to normal liver tissue and a comparison of this immune contexture observer before and after therapy. Metagene analyse will involve CTL response, immune checkpoints, immune checkpoints ligand, Helper T cells response (Th1, Th2 Th9, Th17, Treg, TFH), MDSC, macrophage, dendritic cells, inflammation.

2) A minimum of **5-11 x 4 micron (µm) thick, unstained sections** should be provided and will allow to:

- study the immune cells infiltration into the tumor by immunohistochemistry staining. Immune infiltrate will be assessed by immunohistochemistry (CD3, CD8, PDL1, PD1, CTLA4, Nkp46, Foxp3, DcLamp, CD20, CD14, CD4). Such marker will inform us on the effect of the therapy on variation of T cells infiltrates, NK cells, Treg cells, Dendritic cells, MDSC). The samples will be stored at the site of patient’s care and centralized at the “plateforme de Transfer en Biologie du Cancer” in the Centre Georges-François Leclerc in Dijon under the direction of Pr. François GHIRINGHELLI.
- analyze PD-1 and PD-L1 (using SP263 antibody) or CTLA-4 tumor expression by IHC and PCR. The samples will be stored at the site of patient’s care and centralized in the Platform of Molecular Genetics (CGFL, Dijon), under the direction of Romain Boidot.
  - ➢ <u>Quality control of samples to be used for PD-L1 testing and also for Whole exome and RNAseq analysis:</u>
- The site pathologist prior to PD-L1 testing should assess tissue.
- Each sample should be reviewed for:
  - Adequate fixation
  - Good preservation of morphology
  - Presence of tumor tissue
  - Histopathology consistent with indication
  - Greater than 100 tumor cells are required to determine PD-L1 status – tumor cell content must be reviewed prior to testing in order for PD-L1 obtain a valid result.

➢ Sectioning instructions

Where it is not possible or indicated to ship the block to laboratory for PD-L1 testing unstained slides should be prepared from the paraffin-embedded tumor sample block as described below:

- A minimum of 5-11 x 4 micron (µm) thick, unstained sections should be provided for PD-L1 testing + a minimum of 10 x 5 micron (µm) thick, unstained should be provided for whole exome analysis and RNAseq
- A new disposable microtome blade must be used for each block to prevent contamination between Slides are stable under these conditions for 6 months.
- Patient samples
- Apply one section per slide to positively-charged Superfrost glass slides
- For PD-L1 The sections should be dried overnight between room temperature and 37°C. Do not dry sections at temperatures above 37°C.

Sections should be stored at ambient temperature and protected from light until use or shipment to testing lab by courier at ambient temperature. It is recommended that slides are cut freshly prior to PD-L1 testing and they are used within 90 days of being cut to obtain PD-L1 status

⇨ **Fresh biopsy tissue samples**

Fresh metastasis Biopsies (Liver metastasis will be preferable) will be performed according to the following protocol. Samples will be collected via a core needle of 18 gauge or larger or be collected as an excisional tumour biopsy sample. Where institutional practice in this setting uses a smaller gauge needle, samples should be submitted to ensure that availability of result can be achieved. Two fine needle biopsy of metastases will be performed under ultrasound scanning appliance or by their anatomical position in the tissue via a core needle of 18 gauge or larger. This type of biopsy has shown its safety in conditions similar to patients treated with antiangiogenic (Cancer 2013 Apr 1; 119 (7): 1357-1364).

The two fine needles will be stored in formalin and then Formalin fixed and paraffin embedded (FFPE) as decribed below:

• Fixation of biopsy samples for immune response analyze testing
- Previously frozen tissue is not acceptable for processing to FFPE for PD-L1 testing. To fix newly acquired tissue, place immediately (within 30 min of excision) into an adequate volume of 10% v/v neutral buffered formalin (NBF). Samples should remain in fixative for 24 – 48 hours at room temperature.
- It is vital that there is an adequate volume of fixative relevant to the tissue (at least a 10 volume excess) and that large specimens (if any) are incised prior to fixation to promote efficient tissue preservation.

• Embedding in paraffin for immune response analyze testing
- An overnight processing schedule into paraffin is recommended
- Below is the suggested routine overnight processing schedule:

• Storage of tumor blocks for immune response analyze testing

- FFPE blocks should be stored at ambient temperature and protected from light until shipment by courier at ambient temperature. FFPE blocks are stable under these conditions for an indefinite period. Please refer to section 8.3.1 a) for sectioning instructions

Same protocol as described above in section should be observed for sampling preparation of:
- 10 x 5 micron (µm) thick, unstained for whole exome analysis and RNAseq + 1 HES blade encircling the tumor zone and an estimate of the tumor cell content of the fringed area.
- 5-11 x 4 micron (µm) thick, unstained sections

8.3.1.2 Ancillary study (Immunohistochemistry)

If patient agree to participate to the ancillary studies, a double labelling of Th1, Th2, Th17, Follicular helper T cells will be performed at 3 different time point:

- Screening
- Visit 5 (3 Months)
- Visit 12 (6 Months)

- If archival tissue samples from metastasis or initial tumor is available and sufficient to performed 6 X 4µm thick, unstained sections, no new biopsy is required at screening.

- If no archival tissue samples is available a new biopsy will be required at screening with the same procedure described above in the "**Fresh biopsy tissue samples" part.**

For visit 5 and 12 a new biopsy will be required in order to have enough tissue to performed a minimum of 6 X 4µm unstained section from FFPE block.

Double IHC procedures will be performed once validated by a pathologist. If double staining cannot be appropriately validated, single stains as those for the main study will be performed.

8.3.1.3 Tissues Samples shipment

In case of tissue limiting, we will prioritize Exome analysis and RNA-seq first, then we will address PD1, PDL1 and CD8 expression by IHC and finally we will use material to performed double staining IHC, which we estimate less important. The tumour specimen submitted to establish eligibility should be of sufficient quantity (i.e. >100 tumour cells) to allow for PD-L1 immunohistochemistry analyses.

The following fields of data should be collected from the site/institution collecting and if, indicated shipping of the samples:

- Patient identifier (ecode or unique identifier)
- Specimen identifier (written on the specimen)
- Site identifier
- Specimen collection date
- Type of specimen submitted
- Quantity of specimen
- Date of sectioning

- Archival or fresh tumor and localization (intial tumor or metastasis)
- Tumor type
- Primary tumor location
- Metastatic tumor location (if applicable)
- Fixative

The following fields of data should be collected from PD-L1 testing laboratory:

- Are the negative and positive controls stained correctly
- Is the H&E material acceptable
- Is morphology acceptable
- Total percent positivity of PD-L1 in tumor cells
- PD-L1 status (positive, negative or NA) in tumor cells

Total percent positivity of PD-L1 in infiltrating immune.

Samples will be send at the end of the study by a shipment organized by the sponsor.

8.3.1.4 Summary of tissue sample management

**Whole exome analysis and RNAseq**	
Screening	Archival tissue samples (initial tumor or metastasis)
	- 10 X 5 µm thick, unstained sections from FFPE block<br>- 1 HES blade encircling the tumor zone and an estimate of the tumor cell content of the fringed area
	Fresh biopsie (metastasis)
	2 core fresh biopsy fixed in formalin and paraffin<br>- 10 X 5 µm thick, unstained sections from FFPE block<br>- 1 HES blade encircling the tumor zone and an estimate of the tumor cell content of the fringed area
**Condition of sending**	Send at room temperature throughout the study or one single sending at 4°C at the end of the study
**Shipment adress**	Centre Georges-François Leclerc<br>Plateforme de transfert en biologie cancérologie<br>Etude Medi-Treme-Colon - Romain Boidot<br>1 rue du Pr Marion<br>21079 Dijon

Immunohistochemistry		
**Principal study**	Screening	**Archival tissue sample (initial tumor or metastasis)**
		5 to 11 X 4 µm thick, unstained sections from FFPE block
		**Fresch tissue sample (initial tumor or metastasis)**
		5 to 11 X 4 µm thick, unstained sections from FFPE block
**Ancillary study**	Screening	**Archival tissue sample**
		6 X 4µm thick, unstained sections from FFPE block
		**Fresch tissue sample (metastasis)**
		1 core fresh biopsy fixed in formalin and paraffin<br>6 X 4µm thick, unstained sections from FFPE block
	Visit 5 Day 1 (3 Month)	1 core fresh biopsy fixed in formalin and paraffin<br>6 X 4µm thick, unstained sections from FFPE block
	Visit 12 Day 1 (6 Month)	1 core fresh biopsy fixed in formalin and paraffin<br>6 X 4µm thick, unstained sections from FFPE block
**Condition of sending**		Send at room temperature throughout the study or one single sending at 4°C at the end of the study
**Shipment adress**		Centre Georges-François Leclerc<br>Plateforme de transfert en biologie cancérologie<br>Etude Medi-Treme-Colon - Romain Boidot<br>1 rue du Pr Marion<br>21079 Dijon

	Summary of tissue samples required at screening	
	Principal study	Principal + ancillary study
Exome analysis	10 X 5µm + 11 X 4µm	10 X 5µm + 11 X 4µm + 6 X 4µm
IHC principal study		
IHC ancillary study		

Table 6. Summary of Biopsy tissues samples

8.3.2. Immunomonitoring study (Ancillary study):

In total, four blood sampling time will be required to study the immune response analysis:

- At the inclusion, before first treatment is administrated (Cycle 1 day1) (Day 0)
- At Visit 2 (Cycle 2 day 1 before treatment of Cycle 2 is administrated), (Day 15)
- At Visit 5 (Cycle 5 day 1 before treatment of Cycle 5 is administrated), (Day 90)
- At Visit 12 (Cycle 12 day1 before treatment of Cycle 12 is administrated), (Day 180), unless progression diseases occur before C12, in that case samples will require to be collected at the end of treatment visit.

* At each time point, the following samples will be collected:

  - 7 EDTA tubes (Purple cap) - 6mL: in total 42 mL
  - 2 Heparin tubes (Without gel, dark green cap) - 6mL: in total 12 mL
  - 1 dry tube (without gel, red cap) - 6mL: in total 6 mL

Each sample should be identified with the following informations:

- name of the study
- identification number of the site
- inclusion number of the patient
- name of the Visit (Visit 1/Visit 2/Visit5/Visit 12 or EOT)
- date and time of collection / name of the study.

● **6 EDTA tubes:** As soon as the 6 tubes are collected, homogenize the tubes by about 10 aversions. Label the tubes with the patient's anonymization number, the type of visit and the date of the visit. The samples will be sending by a carrier dedicated to the study. Samples must be sent within 24 hours to the central laboratory "The biomonitoring platform CIC1431 – Hôpital Jean-Minjoz, 3Boulevard alexandre Flemming, 25030 Besançon " Be careful, the sampling must be done in the morning in order to be removed in the afternoon. The carrier must be informed the day before the pick-up. No removal can be scheduled on Friday or the day before a public holiday.

Samples will be used to:

- Analyze cytokine production by T cells: Th1 cytokines (IFN-γ, IL-2, TNF), Th2 (IL-4, IL-5, IL-13), Th17 (IL17A), Treg (IL-10, TGF) and inflammatory (IL-1, IL-6, IL 15) must be measured in serum samples on PBMC after stimulation with TCR triggering by Luminex (Biorad) technology.

- study evaluation of lymphocyte reactivity to tumor antigens. The reactivity of the hTERT-specific T cells and other tumor antigens will be tested by ELISpot, by proliferation and by ELISA. A pool of 32 peptides containing pan-HLA derived EBV, CMV viral proteins and influenza (CEF Peptide Pool "Plus" set; CTL Europe GmbH) used as positive control.

Those parts would be realized by the bio-monitoring platform from the CHU of Besançon (CIC1431), under the direction of Pr. Olivier ADOTEVI.

● **1 EDTA** tubes will be used for complete blood count. Each local laboratory procedures will be used for this part, as it is a routine exam. Volume of the tube will depend on local practice (4 or 6 mL)

● **2 HEPARINE:** As soon as the 2 tubes are collected, homogenize the tubes by about 10 aversions. Label the tubes with the patient's anonymization number, the type of visit and the date of the visit. The samples will be send by a carrier dedicated to the study. Samples must be sent within 24 hours to Emeric Limagne at the “plateforme de transfert en biologie cancérologie” from the centre Georges-François Leclerc, 1 rue du Pr Marion, 21079 Dijon. Be careful, the sampling must be done in the morning in order to be removed in the afternoon. The carrier must be informed the day before the pick-up. No removal can be scheduled on Friday or the day before a public holiday.

Samples will be used to study, by multiparameter flow cytometry, the phenotype of lymphocyte subpopulations in peripheral blood. The following markers will be tested:

- Activation markers : CD45RA, CD45RO, HLA-DR, CD28, 4-1BB, (on CD8 and CD4 T cells),
- Anergy markers: PD1, BTLA, TIM-3, CTLA-4 (on CD8 and CD4 T cells),
- Immunostaining regulatory T cells: CD3, CD4, CD25, CD127, FoxP3,
- Immunostaining NK cells: CD3, CD56, NKp30, NKp44, NKp46, NKG2D, CD16, CD69, CD86, HLA-DR.

- Immunostaining of PD-L1 expressed by cells in blood.
- MDSC: CD33, HLA-DR, CD14, CD15, Lin-.

● **1 Dry tube** (without gel): As soon as the tube is collected, homogenize the tube by about 10 aversions. Then the tube should be leave at room temperature 30 minutes to allow clotting to take place. The tube should then be centrifuge 10 minutes at 1500g at 20-25°C. The serum will then be split in 3-4 tubes containing 1 mL of serum. Samples should then be stored at -80°C and will be send to the “plateforme de transfer ten biologie cancérologie” at the end of the study.

Serum will allow to performed circulating PD-L1 analysis. Circulated PD-L1 evaluated by ELISA. This part will be realized by CRI INSERM in Dijon, under the direction of Pr. François GHIRINGHELLI.

	**PBMC**	**NFS**	**Whole exome analysis**	**Immuno-phenotyping**	**Serum library**	**Total blood volume**
**C1J1**	X 6 EDTA 6mL (*purple cap)*	X 1 EDTA 6mL or 4mL (*purple cap*	X 1 EDTA 6mL *purple cap*	X 2 HEPARIN 6mL *dark green cap*	X 1 Dry tube 6mL *red cap*	66 ml
**C2J1**	X 6 EDTA 6mL *purple cap*	X 1 EDTA 6mL or 4mL *purple cap*		X 2 HEPARIN 6mL *dark green cap*	X 1 Dry tube 6mL *red cap*	60ml
**C5J1**	X 6 EDTA 6mL *purple cap*	X 1 EDTA 6mL or 4mL *purple cap*		X 2 HEPARIN 6mL *dark green cap*	X 1 Dry tube 6mL *red cap*	60ml
**C12J1**	X 6 EDTA 6mL *purple cap*	X 1 EDTA 6mL or 4mL *purple cap*		X 2 HEPARIN 6mL *dark green cap*	X 1 Dry tube 6mL *red cap*	60ml
**Condition for sending**	room temperature within 24h	NA	room temperature within 24h		at the end of the study within dry ice	
**Shipment Adress**	The biomonitoring platform CIC1431 – Hôpital Jean-Minjoz, 3Boulevard alexandre Flemming, 25030 Besançon	Local analysis	plateforme de transfert en biologie cancérologie centre Georges-François Leclerc, 1 rue du Pr Marion, 21079 Dijon		plateforme de transfert en biologie cancérologie centre Georges-François Leclerc, 1 rue du Pr Marion, 21079 Dijon	

Table 7. Volume of Blood to be drawn From Each Subject

8.3.3. Withdrawal of informed consent for donated biological samples

If a subject withdraws consent to the use of donated samples, the samples will be disposed of/destroyed, and the action documented. As collection of the biological samples is an integral part of the study, then the subject is withdrawn from further study participation.

The Principal Investigator:

- Ensures that biological samples from that subject, if stored at the study site, are immediately identified, disposed of /destroyed, and the action documented
- Ensures the laboratory(ies) holding the samples is/are informed about the withdrawn consent immediately and that samples are disposed/destroyed, the action documented and the signed document returned to the study site
- Ensures that the subject is informed about the sample disposal.

9. DISEASE EVALUATION AND METHODS

The response to immunotherapy may differ from the typical responses observed with cytotoxic chemotherapy including the following (Wolchok et al 2009, Nishino et al 2013):

- Response to immunotherapy may be delayed
- Response to immunotherapy may occur after PD by conventional criteria
- The appearance of new lesions may not represent PD with immunotherapy
- SD while on immunotherapy may be durable and represent clinical benefit.

Based on the above-described unique response to immunotherapy and based on guidelines from regulatory agencies, e.g., European Medicines Agency's "Guideline on the evaluation of anti-cancer medicinal products in man" (EMA/CHMP/205/95/Rev.4) for immune modulating anti-cancer compounds, the study may wish to implement the following in addition to standard RECIST 1.1 criteria:

- RECIST will be modified so that PD must be confirmed at the next scheduled visit, preferably, and no earlier than 4 weeks after the initial assessment of PD in the absence of clinically significant deterioration. Treatment with durvalumab + tremelimumab would continue between the initial assessment of progression and confirmation for progression.

- In addition, subjects may continue to receive durvalumab + tremelimumab beyond confirmed PD in the absence of clinically significant deterioration and if investigators consider that subjects continue to receive benefit from treatment.

Modification of RECIST as described may discourage the early discontinuation of durvalumab + tremelimumab and provide a more complete evaluation of its anti-tumor activity than would be seen with conventional response criteria. Nonetheless, the efficacy analysis will be conducted by programmatically deriving each efficacy endpoint based on RECIST 1.1 criteria.

Of note, clinically significant deterioration is considered to be a rapid tumor progression that necessitates treatment with anti-cancer therapy other than durvalumab + tremelimumab or with symptomatic progression that requires urgent medical intervention (e.g., central nervous system metastasis, respiratory failure due to tumor compression, spinal cord compression).>>

9.1. RECIST 1.1 criteria

Patients will undergo regular tumor assessments until documented disease progression as described by RECIST 1.1 criteria. CT scan performed at the following time point: screening (baseline); Cycle 6; Cycle 12 and Cycle 18

Response criteria are essentially based on a set of measurable lesions identified at baseline as target lesions, and followed until disease progression.

The following paragraphs are a quick reference to the RECIST criteria.

9.1.1. Measurability of tumor lesions at baseline

Definitions

♦ Measurable lesions - lesions that can be accurately measured in at least one dimension with longest diameter ≥20 mm. With spiral CT scan, lesion must be ≥10 mm in at least one dimension.

♦ Non-measurable lesions - all other lesions, including small lesions (longest diameter < 20 mm with conventional techniques or < 10 mm with spiral CT scan) and other non-measurable lesions. These include: bone lesions; leptomeningeal disease; ascites; pleural / pericardial effusion; inflammatory breast disease; lymphangitis cutis / pulmonis; abdominal masses that are not confirmed and followed by imaging techniques; and cystic lesions.

All measurements should be recorded in metric notation by use of a ruler or calipers. All baseline evaluations should be performed as closely as possible to the beginning of treatment and never more than 21 days before the beginning of the treatment.

Tumor response evaluation

Target lesions should be selected on the basis of their size (those with the longest diameter) and their suitability for accurate repetitive measurements (either by imaging techniques or clinically).

A sum of the longest diameter (LD) for all target lesions will be calculated and reported as the baseline sum LD. The baseline sum LD will be used as reference by which to characterize the objective tumour response.

All other lesions (or sites of disease) should be identified as non-target lesions and should also be recorded at baseline. Measurements are not required but the presence or absence of each should be noted throughout follow-up.

9.1.2. Response Criteria

Patient will be considered in response if they are in complete response (CR), Partial response (PR) or stable disease (SD) as described below.

Evaluation of target lesions

Complete Response (CR):	Disappearance of all target lesions.
Partial Response (PR):	At least a 30% decrease in the sum of LD of target lesions taking as reference the baseline sum LD.
Progression (PD):	At least a 20% increase in the sum of LD of target lesions taking as references the smallest sum LD recorded since the treatment started or the appearance of one or more new lesions.
Stable Disease (SD):	Neither sufficient shrinkage to qualify for PR nor sufficient increase to qualify for PD taking as references the smallest sum LD since the treatment started.

Evaluation of non target lesions

Complete Response (CR):	Disappearance of all non-target lesions and normalization of tumor marker level.
Incomplete Response / Stable Disease	Persistence of one or more non-target lesion(s) or/and maintenance of tumor marker level above the normal limits.
Progression (PD):	Appearance of one or more new lesions and/or unequivocal progression of existing non-target lesions (*).

(*) Although a clear progression of "non target" lesions only is exceptional, in such circumstances, the opinion of the treating physician should prevail and the progression status should be confirmed later on by the review panel (or study chair).

Best overall response

If measurable disease is present at different sites in a given patient, the response is assessed globally for all sites. The best overall response is the best response recorded from the start of the treatment until disease progression/recurrence (taking as reference for progressive disease the smallest measurements recorded since the treatment started). The patient's best response assignment will depend on the achievement of both measurement and confirmation criteria.

Target lesions	Non-Target lesions	New Lesions	Overall response
CR	CR	No	CR
CR	Incomplete response / SD	No	PR
PR	Non-PD	No	PR
SD	Non-PD	No	SD
PD	Any	Yes or No	PD
Any	PD	Yes or No	PD
Any	Any	Yes	PD

CGFL CENTRE GEORGES FRANÇOIS LECLERC Ensemble, dépassons le cancer

9.2. Modified irRC methods

- Overall irCR: Complete disappearance of all lesions (whether measurable or not) and no new lesions. All measurable lymph nodes also must have a reduction in short axis to 10 mm or less.

- Overall irPR: Sum of the longest diameters of target and new measurable lesions decreases ≥ 30%. (compared to baseline)

- Overall irSD: Sum of the longest diameters of target and new measurable lesions neither irCR, irPR, (compared to baseline) or irPD (compared to nadir).

- Overall irPD: Sum of the longest diameters of target and new measurable lesions increases ≥ 20% (compared to nadir), confirmed by a repeat, consecutive observations at least 4 weeks (normally it should be done at 6 weeks) from the date first documented

	**RECIST 1.1**	**Modified IrRC**
Target / Index Lesions	Up to 5 total (2/organ) unidimensional	Up to 5 total (2/organ), unidimensional
New lesions	Always represent PD	Incorporated into SOLD if ≥ 5X5mm (non-nodal) or ≥ 10mm (nodal)
Non-Target / Index Lesions	Contribute to define CR, PR, SD and PD	Contribute to define modified IrCR ONLY
CR	Disappearance of all known lesions; confirmed at 4 weeks	Disappearance of all known lesions; confirmed ≥ 4 weeks
PR	At least 30% decrease in SOLD; confirmed at ≥ 4 weeks	At least 30% decrease in SOLD; confirmed at ≥ 4 weeks
SD	Neither PR or PD criteria met	Neither PR or PD criteria met
PD	20% increase in SOLD compared with nadir (and absolute increase of 5mm) andor uniequivocal progressions of non-target lesions and/or appearance of nex lesions.	SOLD of target and new measurable lesions increases ≥ 20 % (compared to nadir), confirmed ≥ 4 weeks (normally done at 6 weeks) from the date first documented

9.3. Progression free survival (PFS)

Progression free survival is defined as the time from enrolment until the date of objective disease progression or death (by any cause in the absence of progression). The date of PFS will be recorded by the investigator and defined according to local standard clinical practice and may involve any of: objective radiological progression, symptomatic progression or death. Progression free survival data will be collected at Months 3 and Months 6.

9.4. Overall survival (OS)

Overall survival (months) is defined as the time from the date of enrolment until death due to any cause. Any patient not known to have died at the time of analysis will be censored based on the last recorded date on which the patient was known to be alive. Overall survival data will be collected at 12 months.

Subjects who have disease control following completion of 12 months of treatment or subjects who are withdrawn from durvalumab + tremelimumab treatment for reasons other than confirmed PD will continue to have objective tumor assessments (see Appendix 3).

10. ASSESSMENT OF SAFETY

The Principal Investigator is responsible for ensuring that all staff involved in the study is familiar with the content of this section.

10.1. Safety Parameters

10.1.1. Definition of adverse events

The International Conference on Harmonization (ICH) Guideline for Good Clinical Practice (GCP) E6 (R1) defines an AE as:

Any untoward medical occurrence in a patient or clinical investigation subject administered a pharmaceutical product and which does not necessarily have a causal relationship with this treatment. An AE can therefore be any unfavorable and unintended sign (including an abnormal laboratory finding), symptom, or disease temporally associated with the use of a medicinal product, whether or not considered related to the medicinal product.

An AE includes but is not limited to any clinically significant worsening of a subject’s pre-existing condition. An abnormal laboratory finding (including ECG finding) that requires an action or intervention by the investigator, or a finding judged by the investigator to represent a change beyond the range of normal physiologic fluctuation, should be reported as an AE.

Adverse events may be treatment emergent (ie, occurring after initial receipt of investigational product) or nontreatment emergent. A nontreatment-emergent AE is any new sign or symptom, disease, or other untoward medical event that begins after written informed consent has been obtained but before the subject has received investigational product.

Elective treatment or surgery or preplanned treatment or surgery (that was scheduled prior to the subject being enrolled into the study) for a documented pre-existing condition, that did not worsen from baseline, is not considered an AE (serious or nonserious). An untoward medical event occurring during the prescheduled elective procedure or routinely scheduled treatment should be recorded as an AE or SAE.

The term AE is used to include both serious and non-serious AEs.

10.1.2. Definition of serious adverse events

A serious adverse event is an AE occurring during any study phase (i.e., screening, run-in, treatment, wash-out, follow-up), at any dose of the study drugs that fulfils one or more of the following criteria:

- Results in death
- Is immediately life-threatening

- Requires in-patient hospitalization or prolongation of existing hospitalization
- Results in persistent or significant disability or incapacity
- Is a congenital abnormality or birth defect in offspring of the subject
- Is an important medical event that may jeopardize the patient or may require medical intervention to prevent one of the outcomes listed above:
- Medical or scientific judgment should be exercised in deciding whether expedited reporting is appropriate in this situation. Examples of medically important events are intensive treatment in an emergency room or at home for allergic bronchospasm, blood dyscrasias, or convulsions that do not result in hospitalizations; or development of drug dependency or drug abuse.

The causality of SAEs (their relationship to all study treatment/procedures) will be assessed by the investigator(s) and communicated to AstraZeneca.

10.1.3. Durvalumab + tremelimumab adverse events of special interest

An adverse event of special interest (AESI) is one of scientific and medical interest specific to understanding of the Investigational Product and may require close monitoring and rapid communication by the investigator to the sponsor. An AESI may be serious or non-serious. The rapid reporting of AESIs allows ongoing surveillance of these events in order to characterize and understand them in association with the use of this investigational product.

AESIs for durvalumab and tremelimumab include but are not limited to events with a potential inflammatory or immune-mediated mechanism and which may require more frequent monitoring and/or interventions such as steroids, immunosuppressants and/or hormone replacement therapy. These AESIs are being closely monitored in clinical studies with durvalumab monotherapy and combination therapy. An immune-related adverse event (irAE) is defined as an adverse event that is associated with drug exposure and is consistent with an immune-mediated mechanism of action and where there is no clear alternate aetiology. Serologic, immunologic, and histologic (biopsy) data, as appropriate, should be used to support an irAE diagnosis. Appropriate efforts should be made to rule out neoplastic, infectious, metabolic, toxin, or other etiologic causes of the irAE.

If the Investigator has any questions in regards to an adverse event (AE) being an irAE, the Investigator should promptly contact the Study Physician.

AESIs observed with durvalumab and tremelimumab include:
- Diarrhea / Colitis and intestinal perforation
- Pneumonitis / ILD
- hepatitis / transaminase increases
- Endocrinopathies (i.e. events of hypophysitis/hypopituitarism, adrenal insufficiency, hyper- and hypothyroidism and type I diabetes mellitus)
- Rash / Dermatitis
- Nephritis / Blood creatinine increases
- Pancreatitis / serum lipase and amylase increases
- Myocarditis

- Myositis / Polymyositis
- Neuropathy / neuromuscular toxicity (e.g. Guillain-Barré, and myasthenia gravis)

Other inflammatory responses that are rare / less frequent with a potential immune-mediated aetiology include, but are not limited to, pericarditis, sarcoidosis, uveitis and other events involving the eye,skin, haematological and rheumatological events, vasculitis, non-infectious meningitis and non-infectious encephalitis.

Further information on these risks (e.g. presenting symptoms) can be found in the current version of the durvalumab and tremelimumab Investigator Brochure. For durvalumab and tremelimumab, AESIs will comprise the following:

Pneumonitis

AEs of pneumonitis are also of interest for AstraZeneca, as pneumonitis has been observed with use of anti-PD-1 mAbs (but not with anti-PD-L1 mAbs). Initial work-up should include a high-resolution CT scan, ruling out infection, and pulse oximetry. Pulmonary consultation is highly recommended. Guidelines for the management of patients with immune-related AEs (irAEs) including pneumonitis are provided in Table 1.

Infusion reactions

AEs of infusion reactions (also termed infusion-related reactions) are of special interest to AstraZeneca and are defined, for the purpose of this protocol, as all AEs occurring from the start of IP infusion up to 48 hours after the infusion start time. For all infusion reactions, SAEs should be reported to AstraZeneca Patient safety as described in Section 10.3.

Hypersensitivity reactions

Hypersensitivity reactions as well as infusion-related reactions have been reported with anti-PD-L1 and anti-PD-1 therapy (Brahmer et al 2012). As with the administration of any foreign protein and/or other biologic agents, reactions following the infusion of mAbs can be caused by various mechanisms, including acute anaphylactic (IgE-mediated) and anaphylactoid reactions against the mAbs and serum sickness. Acute allergic reactions may occur, may be severe, and may result in death. Acute allergic reactions may include hypotension, dyspnea, cyanosis, respiratory failure, urticaria, pruritus, angioedema, hypotonia, arthralgia, bronchospasm, wheeze, cough, dizziness, fatigue, headache, hypertension, myalgia, vomiting, and unresponsiveness. Guidelines for the management of patients with hypersensitivity (including anaphylactic reaction) and infusion-related reactions are provided in Table 1.

Hepatic function abnormalities (hepatotoxicity)

Hepatic function abnormality is defined as any increase in ALT or AST to greater than 3 × ULN and concurrent increase in total bilirubin to be greater than 2 × ULN. Concurrent findings are those that derive from a single blood draw or from separate blood draws taken within 8 days of each other. Follow-up investigations and inquiries will be initiated promptly by the investigational site to determine whether the findings are reproducible and/or whether there is objective evidence that clearly supports causation by a disease (eg, cholelithiasis and bile duct obstruction with distended gallbladder) or an agent other than the IP are provided in Table 1.

Gastrointestinal disorders

Diarrhea/colitis is the most commonly observed treatment emergent SAE when tremelimumab is used as monotherapy. In rare cases, colon perforation may occur that requires surgery (colectomy) or can lead to a fatal outcome if not properly managed. Guidelines on management of diarrhea and colitis in patients receiving tremelimumab are provided in Table 1.

Endocrine disorders

Immune-mediated endocrinopathies include hypophysitis, adrenal insufficiency, and hyper- and hypothyroidism. Guidelines for the management of patients with immune-mediated endocrine events are provided in Table 1.

Pancreatic disorders

Immune-mediated pancreatitis includes autoimmune pancreatitis, and lipase and amylase elevation. Guidelines for the management of patients with immune-mediated pancreatic disorders are provided in Table 1.

Neurotoxicity

Immune-mediated nervous system events include encephalitis, peripheral motor and sensory neuropathies, Guillain-Barré, and myasthenia gravis. Guidelines for the management of patients with immune-mediated neurotoxic events are provided in Table 1.

Nephritis

Consult with Nephrologist. Monitor for signs and symptoms that may be related to changes in renal function (e.g. routine urinalysis, elevated serum BUN and creatinine, decreased creatinine clearance, electrolyte imbalance, decrease in urine output, proteinuria, etc)

Patients should be thoroughly evaluated to rule out any alternative etiology (e.g., disease progression, infections etc.)

Steroids should be considered in the absence of clear alternative etiology even for low grade events (Grade 2) , in order to prevent potential progression to higher grade event. Guidelines for the management of patients with immune-mediated neurotoxic events are provided in Table 1.

10.1.4. Immune-related adverse events

Based on the mechanism of action of durvalumab and tremelimumab leading to T-cell activation and proliferation, there is a possibility of observing irAEs during the conduct of this study. Potential irAEs may be similar to those seen with the use of ipilimumab, BMS-936558 (anti-PD-1 mAb), and BMS-936559 (anti-PD-L1 mAb) and may include immune-mediated enterocolitis, dermatitis, hepatitis (hepatotoxicity), pneumonitis, and endocrinopathies (Hodi et al 2010, Brahmer et al 2012). These AEs are inflammatory in nature and can affect any organ. With anti-PD-L1 and anti-CTLA-4 combination therapy, the occurrence of overlapping or increasing cumulative toxicities that include irAEs could potentially occur at higher frequencies than with either durvalumab or tremelimumab monotherapy. Patients should be monitored for signs and symptoms of irAEs. In the absence of an alternate etiology (eg, infection or PD), an immune-related etiology should be considered for signs or symptoms of enterocolitis, dermatitis, pneumonitis, hepatitis, and endocrinopathy. In addition to the dose

modification guidelines provided in Table 1, it is recommended that irAEs are managed according to the general treatment guidelines outlined for ipilimumab. These guidelines recommend the following:

- Patients should be evaluated to identify any alternative etiology.
- In the absence of a clear alternative etiology, all events of an inflammatory nature should be considered immune related.
- Symptomatic and topical therapy should be considered for low-grade events.
- Systemic corticosteroids should be considered for a persistent low-grade event or for a severe event.
- More potent immunosuppressives should be considered for events not responding to systemic steroids (eg, infliximab or mycophenolate).
- If the Investigator has any questions in regards to an AE being an irAE, the Investigator should immediately contact the Study Physician. Assessment of safety parameters

10.1.5. Assessment of severity

Assessment of severity is one of the responsibilities of the investigator in the evaluation of AEs and SAEs. Severity will be graded according to <<the NCI CTCAE v4.03.
The determination of severity for all other events not listed in the CTCAE should be made by the investigator based upon medical judgment and the severity categories of Grade 1 to 5 as defined below:

Grade 1 (mild)	An event that is usually transient and may require only minimal treatment or therapeutic intervention. The event does not generally interfere with usual activities of daily living.
Grade 2 (moderate)	An event that is usually alleviated with additional specific therapeutic intervention. The event interferes with usual activities of daily living, causing discomfort but poses no significant or permanent risk of harm to the subject.
Grade 3 (severe)	An event that requires intensive therapeutic intervention. The event interrupts usual activities of daily living, or significantly affects the clinical status of the subject.
Grade 4 (life threatening)	An event, and/or its immediate sequelae, that is associated with an imminent risk of death or with physical or mental disabilities that affect or limit the ability of the subject to perform activities of daily living (eating, ambulation, toileting, etc).
Grade 5 (fatal)	Death (loss of life) as a result of an event.

It is important to distinguish between serious criteria and severity of an AE. Severity is a measure of intensity whereas seriousness is defined by the criteria in Section 9.2.1. A Grade 3 AE need not

necessarily be considered an SAE. For example, a Grade 3 headache that persists for several hours may not meet the regulatory definition of an SAE and would be considered a nonserious event, whereas a Grade 2 seizure resulting in a hospital admission would be considered an SAE.

10.1.6. Assessment of relationship

Relationship to Investigational Product

The investigator is required to provide an assessment of relationship of AEs and SAEs to the investigational product.

- An event will be considered "not related" to use of the investigational product if any of the following tests are met:
- An unreasonable temporal relationship between administration of the investigational product and the onset of the event (eg, the event occurred either before, or too long after, administration of the investigational product for it to be considered product-related)
- A causal relationship between the investigational product and the event is biologically implausible (eg, death as a passenger in an automobile accident)
- A clearly more likely alternative explanation for the event is present (eg, typical adverse reaction to a concomitant drug and/or typical disease-related event)

Individual AE/SAE reports will be considered "related" to use of the investigational product
if the "not related" criteria are not met. "Related" implies that the event is considered to be "associated with the use of the drug" meaning that there is "a reasonable possibility" that the event may have been caused by the product under investigation (ie, there are facts, evidence, or arguments to suggest possible causation).

Relationship to Protocol Procedures

The investigator is also required to provide an assessment of relationship of SAEs to protocol procedures on the SAE Report Form. This includes non-treatment emergent SAEs (ie, SAEs that occur prior to the administration of investigational product) as well as treatment emergent SAEs. A protocol-related SAE may occur as a result of a procedure or intervention required during the study (eg, blood collection, washout of an existing medication). The following guidelines should be used by investigators to assess the relationship of SAEs to the protocol: Protocol related: The event occurred due to a procedure/intervention that was described in the protocol for which there is no alternative etiology present in the subject's medical record. Not protocol related: The event is related to an etiology other than the procedure/intervention that was described in the protocol (the alternative etiology must be documented in the study subject's medical record).

10.2. Recording of adverse events and serious adverse events

Adverse events will be recorded in the eCRF using a recognized medical term or diagnosis that accurately reflects the event. Adverse events will be assessed by the investigator for severity, relationship to the investigational product, possible etiologies, and whether the event meets criteria of an SAE and therefore requires immediate notification to AstraZeneca/MedImmune Patient Safety.

The following variables will be collected for each AE:

- AE (verbatim)
- The date when the AE started and stopped
- Changes in NCI CTCAE grade and the maximum CTC grade attained
- Whether the AE is serious or not
- Investigator causality rating against durvalumab (yes or no) <<and/or comparator/combination drug (yes/no)>>
- Action taken with regard to durvalumab /comparator/combination agent
- Outcome

In addition, the following variables will be collected for SAEs as applicable:

- Date AE met criteria for serious AE
- Date Investigator became aware of serious AE
- AE is serious due to criteria
- Date of hospitalization
- Date of discharge
- Probable cause of death
- Date of death
- Autopsy performed
- Description of AE
- Causality assessment in relation to Study procedure(s)
- Causality assessment in relation to FOLFOX

Events, which are unequivocally due to disease progression, should not be reported as an AE during the study.

10.2.1. Study recording period and follow-up for adverse events and serious adverse events

Adverse events and serious adverse events will be recorded from time of signature of informed consent, throughout the treatment period and including the follow-up period (90 days after the last dose of durvalumab + tremelimumab or until the initiation of alternative anticancer therapy).

During the course of the study all AEs and SAEs should be proactively followed up for each subject. Every effort should be made to obtain a resolution for all events, even if the events continue after discontinuation/study completion.

If a subject discontinues from treatment for reasons other than disease progression, and therefore continues to have tumor assessments, drug or procedure-related SAEs must be captured until the patient is considered to have confirmed PD and will have no further tumor assessments.

The investigator is responsible for following all SAEs until resolution, until the subject returns to baseline status, or until the condition has stabilized with the expectation that it will remain chronic, even if this extends beyond study participation.

Follow-up of unresolved adverse events

Any AEs that are unresolved at the subject's last visit in the study are followed up by the investigator for as long as medically indicated, but without further recording in the eCRF. After 90 days, only subjects with ongoing investigational product-related SAEs will continue to be followed for safety.

AstraZeneca/MedImmune retains the right to request additional information for any subject with ongoing AE(s)/SAE(s) at the end of the study, if judged necessary.

Post study events

After the subject has been permanently withdrawn from the study, there is no obligation for the investigator to actively report information on new AE or SAEs occurring in former study subjects after the 90-day safety follow-up period for patients treated with durvalumab + tremelimumab. However, if an investigator learns of any SAEs, including death, at any time after the subject has been permanently withdrawn from study, and he/she considers there is a reasonable possibility that the event is related to study treatment, the investigator should notify the study sponsor and AstraZeneca/MedImmune Drug Safety.

10.2.2. Reporting of serious adverse events

All SAEs will be reported, whether or not considered causally related to the investigational product, or to the study procedure(s). The reporting period for SAEs is the period immediately following the time that written informed consent is obtained through 90 days after the last dose of durvalumab + tremelimumab or until the initiation of alternative anticancer therapy. The investigator and/or Sponsor are responsible for informing the Ethics Committee and/or the Regulatory Authority of the SAE as per local requirements.

**** Send SAE report IMMEDIATELY and accompanying cover page by way of email to UNICANCER:***

R and D UNICANCER – Pharmaco Vigilance
101 rue Tolbiac – 75654 PARIS cedex 13
Tel: 01 44 23 04 73
Fax: 01 44 23 55 70
Email: pv-rd@unicancer.fr

If a non-serious AE becomes serious, this and other relevant follow-up information must also be provided to AstraZeneca and the FDA.

Serious adverse events that do not require expedited reporting to the FDA still need to be reported to AstraZeneca preferably using the MedDRA coding language for serious adverse events. This information should be reported on a monthly basis and under no circumstance less frequently than quarterly.

10.2.2.1. Reporting of deaths

All deaths that occur during the study, or within the protocol-defined 90-day post-last dose of durvalumab + tremelimumab (or until the initiation of alternative anticancer therapy) safety follow-up period must be reported as follows:

- Death that is clearly the result of disease progression should be documented but should not be reported as an SAE.
- Where death is not due (or not clearly due) to progression of the disease under study, the AE causing the death must be reported to as a SAE within **24 hours** (see Section 10.3.2 for further details). The report should contain a comment regarding the co-involvement of progression of disease, if appropriate, and should assign main and contributory causes of death.
- Death with an unknown cause should always be reported as a SAE.
- Death that occur following the protocol-defined 90-day post-last-dose of MEDI4736 safety follow-up period will be documented <<as events for survival analysis>>, but will not be reported as an SAE.

10.2.3. Other events requiring reporting

10.2.3.1. Overdose

An overdose is defined as a subject receiving a dose of durvalumab + tremelimumab in excess of that specified in the Investigator's Brochure, unless otherwise specified in this protocol.

Any overdose of a study subject with durvalumab + tremelimumab, with or without associated AEs/SAEs, is required to be reported within 24 hours of knowledge of the event to the sponsor and AstraZeneca/MedImmune Patient Safety or designee using the designated Safety e-mailbox (see Section 10.3.2 for contact information). If the overdose results in an AE, the AE must also be recorded as an AE (see Section 10.3). Overdose does not automatically make an AE serious, but if the consequences of the overdose are serious, for example death or hospitalization, the event is serious and must be recorded and reported as an SAE (see Section 10.3 and Section 10.3.2). There is currently no specific treatment in the event of an overdose of durvalumab or tremelimumab.

The investigator will use clinical judgment to treat any overdose.

10.2.3.2. Hepatic function abnormality

Hepatic function abnormality (as defined in Section 10.1.3.) in a study subject, with or without associated clinical manifestations, is required to be reported as "hepatic function abnormal" ***within 24 hours of knowledge of the event*** to the sponsor and AstraZeneca/MedImmune Patient Safety using the designated Safety e-mailbox (see Section 10.3.2 for contact information), unless a definitive underlying diagnosis for the abnormality (e.g., cholelithiasis or bile duct obstruction) that is unrelated to investigational product has been confirmed.

- If the definitive underlying diagnosis for the abnormality has been established and is unrelated to investigational product, the decision to continue dosing of the study subject will be based on the clinical judgment of the investigator.

- If no definitive underlying diagnosis for the abnormality is established, dosing of the study subject must be interrupted immediately. Follow-up investigations and inquiries must be initiated by the investigational site without delay.

Each reported event of hepatic function abnormality will be followed by the investigator and evaluated by the sponsor and AstraZeneca/MedImmune.

10.2.3.3. Pregnancy

10.2.3.4. Maternal exposure

If a patient becomes pregnant during the course of the study, the IPs should be discontinued immediately.

Pregnancy itself is not regarded as an AE unless there is a suspicion that the IP under study may have interfered with the effectiveness of a contraceptive medication. Congenital abnormalities or birth defects and spontaneous miscarriages should be reported and handled as SAEs. Elective abortions without complications should not be handled as AEs. The outcome of all pregnancies (spontaneous miscarriage, elective termination, ectopic pregnancy, normal birth, or congenital abnormality) should be followed up and documented even if the patient was discontinued from the study.

If any pregnancy occurs in the course of the study, then the Investigator or other site personnel should inform the appropriate AstraZeneca representatives within 1 day, ie, immediately, but **no later than 24 hours** of when he or she becomes aware of it.

The designated AstraZeneca representative will work with the Investigator to ensure that all relevant information is provided to the AstraZeneca Patient Safety data entry site within 1 to 5 calendar days for SAEs and within 30 days for all other pregnancies.

The same timelines apply when outcome information is available.

10.2.4. Paternal exposure

Male patients should refrain from fathering a child or donating sperm during the study and for 180 days after the last dose of durvalumab + tremelimumab combination therapy or 90 days after the last dose of durvalumab monotherapy, whichever is the longer time period.

Pregnancy of the patient’s partner is not considered to be an AE. However, the outcome of all pregnancies (spontaneous miscarriage, elective termination, ectopic pregnancy, normal birth, or congenital abnormality) occurring from the date of the first dose until 90 days after the last dose should, if possible, be followed up and documented.

Where a report of pregnancy is received, prior to obtaining information about the pregnancy, the Investigator must obtain the consent of the patient’s partner. Therefore, the local study team should

adopt the generic ICF template in line with local procedures and submit it to the relevant Ethics Committees (ECs)/Institutional Review Boards (IRBs) prior to use.

11. STATISTICAL METHODS AND SAMPLE SIZE DETERMINATION

11.1. Description of analysis sets for the step 1

Safety and pharmacokinetic analyses will be performed on the first 9 patients. Toxicities during the first 2 cycles will be considered.

11.2. Description of analysis sets for the step 2 (Simon's phase II trial)

11.2.1. Safety analysis set

Safety and pharmacokinetic analyses will be performed on the safety-evaluable population. The safety-evaluable population is defined as all subjects treated with at least one dose of investigational product. Subjects who are not treated with any investigational product will be identified and described separately from the safety-evaluable population.

Erroneously treated patients will be accounted in the group for the treatment they actually received.

11.2.2. Efficacy analysis set

Efficacy analyses will be performed in modified intent-to-treat population (ITT) population ie, all patients following the major inclusion criteria and with a 3 months evaluation. Analyses will be repeated in the intent to treat principle i.e including all randomized patients whatever eligibility criteria and treatment received patients and in the per protocol population (patients who had received all the planned doses).

11.3. Methods of statistical analyses

Continuous variables will be summarized using descriptive statistics, i.e. number of subjects with available data (N), mean, median, standard deviation (S.D.), 25% - 75% quartiles (Q1-Q3) and range. Continuous variables could be transformed as categorical variable using median or using conventional cut-off from bibliography or clinical practice. If required comparison using Chi square (or exact Fisher test) or Student T test (or Wilcoxon Mann and Whitney) tests will be done.

Categorical variables will be described by and percentages. The number of missing data will be described.

Phase I

Safety analyses will be performed on the first 9 patients. Toxicities during the first 2 cycles will be considered. If the same grade 3-4 adverse event is reported for more than 3 patient over 9, the trial could prematurely stop after reunion of the independent committee.

Data on safety will be completed during the second part of the study.

Phase II

11.3.1. Safety Analyses

Analysis of safety endpoint(s)

* Safety analyses will be performed on the safety-evaluable population, defined as all subjects treated with at least one dose of investigational product.
* Toxicities and grades will be described at each cycle.
* The following data will be given:

- The number and percentage of patients with at least one adverse event
- The number and percentage of patients with at least one grade 3 or 4 adverse event
- The number and percentage of patients with at least one serious adverse event
- The number and percentage of patients with at least one adverse event leading to treatment premature stop
- Time until grade 3-4 toxicity will be determined using the Kaplan Meier method. Patients without toxicities will be censored.

11.3.2. Efficacy Analyses set

Efficacy analyses will be performed in modified intent-to-treat population (ITT) population ie, all patients following the major inclusion criteria and with a 3 months evaluation. Analyses will be repeated in the intent to treat principle i.e including all enrolled patients whatever eligibility criteria and treatment received patients and in the per protocol population (patients who had received all the planned doses).

The primary objective will be evaluated at 3 months among MSS patients only. According to the Simon's design, at 3 months, on the first evaluable 43 patients (mITT population): if 26 or more patients respond to treatment the treatment will be considered of interest for evaluation in a phase III trial. Response rates will be evaluated using RECIST criteria. Response will be considered for patient with Complet response (CR), partial response (PR) and stable disease (SD). Overall survival and progression free survival (OS and PFS) will be estimated using Kaplan Meier method. Median survivals will be reported with 95% confidence intervals (CI).

Median follow-up will be estimated using the reverse Kaplan Meier method.
Analysis of survival and response among MSI patients will be performed in an exploratory manner, taken into account the small sample size in this population. All statistical analysis will be performed with Stata 11 or SAS 9.3.

11.3.3. Exploratory Analyses

Analysis of immunogenicity will be done as the standard practice used at the "plateforme de transfer en biologie cancérologie" (PTBC) from Dijon.

11.3.4. Interim analyses

2 interims analyses are planned: after the inclusion of 9 patients for the step 1 safety analysis and after 16 patients for step 2 of efficacy analysis (Simon's design).

STEP 1: An interim analysis is plan after the inclusion of the patient 9 from step with at least two cycles of treatment performed. The purpose of this interim analysis is to stop the study if a to high numbers of toxicity is observed. If the same grade 3-4 adverse event is reported during the first 2 cycles for more than 3 patients over 9, the trial could prematurely be stopped. The independent data monitoring committee will also meet in order to analysis all the toxicity observed on the 9 first patient with a minimum of 2 cycles of treatement.

STEP2: The efficacy of the treatment scheme will be determined in a 2-step phase II study. A second interim analysis will be performed after 16 subjects evaluable for the primary objective (3 months evaluation). On these 16 first evaluable subjects, if 9 or more are in response to treatment, the study will continue and the additional 27 subjects could be enrolled. If 8 or less subjects are in response, the study could be stopped because of futility, after decision of the independent committee. The study can't stop in favor of efficacy. Inclusions will not be stop during this interim analysis in order to maintain the enrollment dynamic.

11.4. Determination of sample size

Step 1: Nine patients will be included in the **safety** analyses. If the same grade 3-4 adverse event is reported for more than 1 patient over 3, the trial could prematurely stop after reunion of the independent committee. Patients from the safety population will be included in the efficacy set.

Step 2: The **efficacy** will be determined using a Simon 2 steps phase II design.

<u>The hypotheses are the following:</u>

The primary objective will be evaluated among MSS patients

A PFS of 3 months is not considered of interest. A PFS of 6 months is expected. This is equivalent to assume that a PFS of 50% at 3 months is insufficient and a PFS of 70.7% is expected. With α=10%, β=10% (90% power), 20% of non-evaluable patients, 52 patients with MSS disease are needed including patients of the safety population (9 patients)

The prevalence of MSS disease is around 90%-95%, thus 5 additional patients will be included

Overall the study will include 57 patients.

12. ETHICAL AND REGULATORY REQUIREMENTS

12.1. Ethical conduct of the study

The study will be performed in accordance with ethical principles that have their origin in the Declaration of Helsinki and are consistent with ICH/Good Clinical Practice, and applicable regulatory requirements Subject data protection.

12.2. Ethics and regulatory review

Before carrying out biomedical research on humans, the sponsor is required to submit the project to the opinion of one of the competent institutional review board where the coordinating investigator operates.

The request of an opinion of the biomedical research project is sent to the committee by the sponsor.

Applications for substantial modifications of the initial projects are also addressed by the sponsor for an opinion from the committee.

12.3. Informed consent

Prior to the implementation of the biomedical research on a person, subject should be fully informed by the investigator during the consultation and after a period of reflection sufficient written informed consent form should be collected.

The information for the trial participants should include all elements defined in the Public Health Act of August 9, 2004 and must be written in a simple and understandable language for the patient.

The consent form must be dated and signed personally by the research participant and investigator (original archived by the investigator, a copy will be given to the research participant).

In the case of research aimed to carry out analyzes of genomics or proteomics, the informed consent form should specify the type of research to be conducted and the patient must have the ability to accept or decline conservation of biological samples for a scientific research purposes.

12.4. Changes to the protocol and informed consent form

Study procedures will not be changed without the agreement of the coordinator of the project Dr Isambert Nicolas.

If there are any substantial changes to the study protocol, then this changes will be documented in a study protocol amendment and where required in a new version of the study protocol (Revised Clinical Study Protocol)

The amendment is to be approved by the relevant EC and if applicable, also the national regulatory authority approval, before implementation. Local requirements are to be followed for revised protocols.

The Centre Georges-François Leclerc will distribute any subsequent amendments and new versions of the protocols to each PI.

If a protocol amendment requires change to the informed consent form, The centre Georges-François Leclerc and the Ethic comity are to approve the revised informed consent form before the revised form is used.

12.5. Audits and inspections

Authorised representatives of Centre Georges François Leclerc or AstraZeneca, a regulatory authority, or an EC may perfrom audits or inspections at the centre, includiong source data verification. The purpose of an audit or inspection is to systematically and independently examine all study related activities and documents, to determine wheter these activities were conducted, and data were recorded, analysed, and accurately reported according to the protocol, GCP, ICH guidelines, and any applicable regulatory requirements. The investigator will contact the Centre de Recherche Clinique from the Centre Georges François Leclerc or its representative immediately if contacted by a regulatory agency about an inspection at the centre.

13. STUDY MANAGEMENT

This study will be managed by the Centre of clinical research (C.R.C) of the Centre Georges François Leclerc.

13.1. Training of study site personnel

Before the first patient is entered into the study, a representative of the CRC from the Centre Georges-François Leclerc will review and discuss the requirements of the Clinical Study Protocol and related documents with the investigational staff and also traim them in any study specific procedures, including how to administer the questionnaires, and the Clinsight and Tenalea system utilized.

The PI will ensure that appropriate training relevant to the study is given to all of these staff, and that any new information relevant to the performance of this study is forwarded to the staff involved.

The PI will maintain a record of all individuals involved in the study; medical, nursing and other staff on the list of delegation of task.

13.2. Monitoring of the study

During the study a representative from the Clinical Research Center form the Centre Georges-François Leclerc will have regular contacts with the study site, including visit to:

Provide information and support to the Investigators

Confirm the facilities remain acceptable

Confirm that the investigational team is adhering to the protocol, that data are being accurately and timely recorded in the eCRFs, that biological samples are handled in accordance with the lavboratory Manual and that study drug accountability checks are being performed.

Performed source data verification (a comparaison of the data in the eCRFs with the patient’s medical records at the hospital practice, and other records relevant to the study) including verification of

informed consent of participating patients. This will require direct access to all original records for each patient.

Ensure withdrawal of informed consent to the use of the patient's biological samples is reported and biological samples are identified and disposed of/destroyed accordingly, and the action is documented, and reported to the patient.

The representative from the Clinical Research Center form the Centre Georges-François Leclerc will be available between visits if the Investigator(s) or other staff at the center needs information and advice about the study conduct.

13.3. Study timetable and end of study

Source data will be considered by all data available in the patient record. Also please refer to the Clinical Study Agreement for the location of source data.

13.4. Study timetable and end of study

The study is expected to start in Q1 2017 and to be completed by last patient last visit by Q2 2020.

The end of the study is defined as "the last visit of the last patient undergoing the study"

The end of the clinical part of the study is defined as "the date of the last patient stopping study treatment"

The study may be terminated at individual study sites if the study procedures are not being performed according to GCP, or if recruitment is slow. The sponsor may also terminate the entire study prematurely if concerns for safety arise within this study.

14. DATA MANAGEMENT

14.1 Documentation of Essential Documents/Supplements at Study Center during the study

An "investigator file" will be established at the study center at the beginning of the trial. The Investigator/Institution must maintain the trial documents as specified in the Guideline for Essential documents for the conduct of a clinical trial (ICH E6(R2), EMA/CHMP/ICH/135/1995) and the applicable regulatory requirement(s). The Investigator/Institution must take measures to prevent accidental or premature destruction of these documents.

14.2 Data protection

In agreement with the General European Data Protection Regulation (GDPR), study participants will be informed through an information notice and consent form of the following rights:

- the collected data nature and purpose as well as retention period for these data;

- the possibility to stop study participation at any time and the retention by the sponsor of the collected data (unless indicated otherwise);
- the right of access, rectification, opposition, limitation, erasure and portability of data collected in the context of the research. These rights may be exercised at any time during research either by making a request to the doctor who follows the participant in the context of the research (and who will contact the sponsor) or to the sponsor Data Protection Officer;
- the possibility to make a complaint to CNIL when a problem and/or disagreement arises.

The sponsor (through the clinical research technician or investigators) commits to respond to a request on data access with a maximum delay of 1 month. In addition, only the sponsor authorized staff (investigators, clinical research associate, clinical research technician) and representatives of the health authorities will be able to access this information.

14.3 Data processing and retention of research data and documents

Rationale of data collection and CNIL declaration

Data collected during this study are for scientific research and public interest purposes.

This study falls within the framework of the CNIL MR01 reference methodology registered for Center Georges François Leclerc, for the following reasons:

- collection of health data for research purposes;
- validation by an ethics committee (CPP) before research initiation;
- use of anonymized data (identification by a monogram and inclusion number);
- information and individual consent of the participants;
- data access exclusively by professionals (health and sponsor) involved in the study.

The fact that this study falls within the framework of MR01 as well as the reasons will be notified in the sponsor's treatment register.

14.4 Data flow process and security

Data will be collected on an electronic case report form (e-CRF) under investigator responsibility, by a member of his team (clinical research associate, clinical research technician...) on:

- an e-CRF hosted (and backed up daily) by a provider and protected by a password changing every 3 months.

Data extraction in SAS version 9.4 or R or STATA will be requested to allow the use of appropriate statistical analysis software and subsequent data analysis.

Data file and the corresponding formats will be read and saved according to the standard operating procedures of CGFL. The data will be saved on the CGFL internal network, in a study dedicated directory, accessible only to the statistician. Internal CGFL network is secured by a firewall that blocks any external intrusion. A proxy server also controls web browsing and anti-virus software examines all files and pages copied from external servers to CGFL. Each person wishing to connect to the CGFL

network must first identify using an identifier provided by the CGFL IT Services and with a personalized password upon initial login.

There are no plans to transfer data outside European Union.

14.5 Data processing, verification and validation procedures (data management)

As data will be entered in e-CRF, controls will be programmed according to the data-management manual and will require correction as soon as data is entered.

Depending on specifications, data validation will eventually be carried out for statistical analysis, and correction requests will be issued to investigator or to study clinical research technician, which will complete and correct the data, according to request.

Database will be frozen if no anomalies are detected in consistency checks and following a decision by the project manager in agreement with methodologist.

14.6 Case Report Forms

An electronic CRF (e-CRF) will be used to record clinical data and is an integral part of the trial. The investigator must reflect patient's status at each phase during the course of the trial. The investigator must ensure accuracy, the completeness and the consistency of the data entered in the e-CRF. Patients must not be identified on the e-CRF by name, but by patient's identification number. The e-CRF is specifically designed to record the data required by this protocol. They must be kept up-to-date so that they always reflect the latest observations of the patients enrolled in the study. The eCRF must be signed electronically by the investigator.

The methodology and biostatistic unit (Dr Aurélie Bertaut) will be on charge of the data management. Queries will be sent every 3 months to the investigational and designated investigator or site staff. Any necessary correction of the data base will be made by the data-manager of the unit.

At the end of the study, when every queries will have been answered, the database will be locked and transmitted to the statistician.

14.7 Archiving

In agreement with the 8 November 2006 Ministerial Order, fixing the period of conservation of data and documents related to biomedical research on a medicinal product for human use by the sponsor and investigator at the end of the research:

- all the documents (different protocol versions, CRF, investigator's brochure, consents, letters, etc.) on paper will be archived, in each center, and by sponsor for at least 15 years.
- all study related informatics data will be kept until final report completion and will be archived for at least 15 years.

Study governance and oversight

The safety of all AstraZeneca clinical studies is closely monitored on an ongoing basis by AstraZeneca representatives in consultation with Patient Safety. Issues identified will be addressed; for instance, this could involve amendments to the study protocol and letters to Investigators.

15. INVESTIGATIONAL PRODUCT AND OTHER TREATMENTS

15.1. Identity of investigational product(s)

Table 9. List of investigational products for this study

Investigational product	Dosage form and strength	Manufacturer
Durvalumab	1500 mg, solution, IV	MedImmune
Tremelimumab	75 mg, solution, IV	MedImmune

16. LIST OF REFERENCES

Andorsky et al 2011
Andorsky DJ, Yamada RE, Said J, Pinkus GS, Betting DJ, and Timmerman JM. Programmed death ligand 1 is expressed by non-hodgkin lymphomas and inhibits the activity of tumor-associated T cells. Clin Cancer Res. 2011. 17(13):4232-4244

Antonia et al 2014b
Antonia S, Ou SI, Khleif SN, Brahmer J, Blake-Haskins A, Robbins PB, et al. Clinical activity and safety of MEDI4736, an anti-programmed cell death ligand-1 (PD-L1) antibody in patients with NSCLC. Poster presented at the European Society for Medical Oncology (ESMO) Meeting; 2014 Sep 26-30; Madrid, Spain.

Antonia et al 2014a
Antonia S, Goldberg S, Balmanoukian A, Narwal R, Robbins P, D'Angelo G, et al. A Phase 1 open-label study to evaluate the safety and tolerability of MEDI4736, an anti-programmed cell death-ligand 1 (PD-L1) antibody, in combination with tremelimumab in patients with advanced non-small cell lung cancer (NSCLC). Poster presented at European Society of Medical Oncology (ESMO) Meeting; 2014 Sep 26-30; Madrid, Spain.

Antonia et al 2015
Antonia et al. Phase ib study of medi4736 in combination with treme in patients with advanced NSCLC. Chicago, Il ASCO May30-June4,ASCO 2015, poster #3014.

Berry et al 1991
Berry G, Kitchin RM, Mock PA. A comparison of 2 simple hazard ratio estimators based on the logrank test. Stat Med 1991;10(5):749-55.

Bograd et al 2011
Bograd AJ, Suzuki K, Vertes E, Colovos C, Morales EA, Sadelain M, et al. Immune responses and immunotherapeutic interventions in malignant pleural mesothelioma. Cancer Immunol Immunother. 2011;60:1509-27.

Bonomi 2010
Bonomi PD. Implications of key trials in advanced nonsmall cell lung cancer. Cancer 2010;116(5):1155-64.

Brahmer et al 2012
Brahmer JR, Tykodi SS, Chow LQM, Hwu WJ, Topalian SL, Hwu P, et al. Safety and activity of anti-PD-L1 antibody in patients with advanced cancer. N Engl J Med 2012;366(26):2455-65.

Brahmer et al 2014
Brahmer JR, Horn L, Gandhi L, Spigel DR, Antonia SJ, Rizvi NA et al. Nivolumab (anti-PD-1, BMS-936558, ONO-4538) in patients (pts) with advanced non-small-cell lung cancer (NSCLC): Survival and clinical activity by subgroup analysis [ASCO abstract 8112]. J Clin Oncol 2014;32(15)(Suppl).

Brusa et al 2013
Brusa D, Serra S, Coscia M, Rossi D, D'Arena G, Laurenti L, et al. The PD-1/PD-L1 axis contributes to T-cell dysfunction in chronic lymphocytic leukemia. Haematologica. 2013 Jun; 98(6):953-963.

Burt et al 2011
Burt BM, Rodig SJ, Tilleman TR, Elbardissi AW, Bueno R, Sugarbaker DJ. Circulating and tumor-infiltrating myeloid cells predict survival in human pleural mesothelioma. Cancer. 2011;117(22):5234-44.

Ciuleanu et al 2009
Ciuleanu T, Brodowicz T, Zielinski C, Kim JH, Krzakowski M, Laack E, et al. Maintenance pemetrexed plus best supportive care versus placebo plus best supportive care for non-small-cell lung cancer: a randomized, double-blind, phase 3 study. Lancet 2009;374(9699):1432-40.

Collett 2003
Collett D. Modelling survival data in medical research: 2nd ed. Chapman and Hall/CRC; 2003.

D'Addario et al 2010
D'Addario G, Fruh M, Reck M, Baumann P, Klepetko W, Felip E, et al. Metastatic non-small-cell lung cancer: ESMO clinical practice guidelines for diagnosis, treatment and follow-up. Ann Oncol 2010;21(Suppl 5):116-9.

Drake et al 2013
Drake CG, McDermott DF, Sznol M, Choueiri TK, Kluger HM, Powderly JD et al. Survival, safety, and response duration results of nivolumab (Anti-PD-1; BMS-936558; ONO-4538) in a phase I trial in patients with previously treated metastatic renal cell carcinoma (mRCC): Long-term patient follow-up [abstract 4514]. J Clin Oncol 2013;31(Suppl).

Dunn et al 2004
Dunn GP, Old LJ, Schreiber RD. The three Es of cancer immunoediting. Annu Rev Immunol 2004;22:329-60.

Ellis et al 2008
Ellis S, Carroll KJ, Pemberton K. Analysis of duration of response in oncology trials. Contemp Clin Trials 2008;29(4):456-65.

Forde et al 2014
Forde PM, Kelly RJ, Brahmer JR. New strategies in lung cancer: translating immunotherapy into clinical practice. Clin Cancer Res 2014;20(5):1067-73.

Gail and Simon 1985
Gail M, Simon R. Tests for qualitative interactions between treatment effects and patient subsets. Biometrics 1985;41(2):361–72.

GLOBOCAN 2012
GLOBOCAN. Lung cancer, estimated incidence, mortality and prevalence worldwide in 2012. International Agency for Research on Cancer, World Health Organization; Lyon, 2012. Available at http://globocan.iarc.fr/Pages/fact_sheets_cancer.aspx. Accessed on 16 April 2015.

Gong et al 2009
Gong AY, Zhou R, Hu G, Li X, Splinter PL, O'Hara SP, et al. MicroRNA-513 regulates B7-H1 translation and is involved in IFN-gamma-induced B7-H1 expression in cholangiocytes. J Immunol 2009;182(3):1325-33.

Hamanshi et al 2007
Hamanishi J, Mandai M, Iwasaki M, Okazaki T, Tanaka Y, Yamaguchi K, et al. Programmed cell death 1 ligand 1 and tumor-infiltrating CD8+ T lymphocytes are prognostic factors of human ovarian cancer. Proc Nat Acad Sci U S A. 2007;104(9):3360-5.

Herbst et al 2013
Herbst RS, Gordon MS, Fine GD, Sosman JA, Soria JC, Hamid O, et al. A study of MPDL3280A, an engineered PD-L1 antibody in patients with locally advanced or metastatic tumours [abstract 3000]. J Clin Oncol 2013;31(Suppl 15).

Hirano et al 2005
Hirano F, Kaneko K, Tamura H, Dong H, Wang S, Ichikawa M, et al. Blockade of B7-H1 and PD-1 by monoclonal antibodies potentiates cancer therapeutic immunity. Cancer Res 2005;65(3):1089-96.

Hodi et al 2010
Hodi FS, O'Day SJ, McDermott DF, Weber RW, Sosman JA, Haanan JB, et al. Improved survival with ipilimumab in patients with metastatic melanoma [published erratum appears in N Engl J Med 2010;363(13):1290]. N Engl J Med 2010;363(8):711-23.

Hodi et al 2010
Hodi FS, O'Day SJ, McDermott DF, Weber RW, Sosman JA, Haanan JB, et al. Improved survival with ipilimumab in patients with metastatic melanoma [published erratum appears in N Engl J Med 2010;363(13):1290]. N Engl J Med 2010;363(8):711-23.

Hodi et al 2014
Hodi FS, Sznol M, Kluger HM, McDermott DF, Carvajal RD, Lawrence DP et al. Long-term survival of ipilimumab-naive patients (pts) with advanced melanoma (MEL) treated with nivolumab (anti-PD-1, BMS-936558, ONO-4538) in a phase I trial [ASCO abstract 9002]. J Clin Oncol 2014; 32(15)(Suppl).

Howlander et al 2014
Howlander N, Noone AM, Krapcho M, Garshell J, Neyman N, Altekruse SF, et al. SEER cancer statistics review, 1975-2011. Bethesda (MD): National Cancer Institute,. Available from: URL:http://seer.cancer.gov/csr/1975_2011/ based on November 2013 SEER data submission (posted to the SEER web site, April 2014).

IASLC Staging Manual in Thoracic Oncology
Goldstraw P, ed. Staging manual in thoracic oncology. 7th ed. IASLC 2010. (Available on request)

Iwai et al 2002
Iwai Y, Ishida M, Tanaka Y, Okazaki T, Honjo T, Minato N. Involvement of PD-L1 on tumour cells in the escape from host immune system and tumour immunotherapy by PD-L1 blockade. Proc Natl Acad Sci USA 2002;99(19):12293-7.

Iwai et al 2002
Iwai Y, Ishida M, Tanaka Y, Okazaki T, Honjo T, Minato N. Involvement of PD-L1 on tumour cells in the escape from host immune system and tumour immunotherapy by PD-L1 blockade. Proc Natl Acad Sci USA 2002;99(19):12293-7.

Keir et al 2008
Keir ME, Butte MJ, Freeman GJ, Sharpe AH. PD-1 and its ligands in tolerance and immunity. Annu Rev Immunol. 2008;26:677-704.

Kirkwood et al 2010
Kirkwood JM, Lorigan P, Hersey P, Hauschild A, Robert C, McDermott D, et al. Phase II trial of tremelimumab (CP-675,206) in patients with advanced refractory or relapsed melanoma. Clin Cancer Res 2010;16(3):1042-8.

Kitano et al 2014
Kitano S, Postow MA, Ziegler CG, Kuk D, Panageas KS, Cortez C, et al. Computational algorithm-driven evaluation of monocytic myeloid-derived suppressor cell frequency for prediction of clinical outcomes. Cancer Immunol Res 2014;2(8):812-21.

Klein et al 2007
Klein JP, Logan B, Harhoff M, Andersen PK. Analyzing survival curves at a fixed point in time. Stat Med 2007;26(24):4505-19.

Korn et al 2008
Korn EL, Liu PY, Lee SJ, Chapman JA, Niedzwiecki D, Suman VJ, et al. Meta-analysis of phase II cooperative group trials in metastatic stage IV melanoma to determine progression-free and overall survival benchmarks for future phase II trials. J Clin Oncol 2008;26(4):527-34.

Krambeck et al 2007
Krambeck AE, Dong H, Thompson RH, Kuntz SM, Lohse CM, Leibovich BC, et al. Survivin and B7-H1 are collaborative predictors of survival and represent potential therapeutic targets for patients with renal cell carcinoma. Clin Cancer Res. 2007;13(6):1749-56.

Kresowik and Griffith 2009
Kresowik TP, Griffith TS. Bacillus Calmette–Guerin immunotherapy for urothelial carcinoma of the bladder. Immunother. 2009;1(2):281-8.

Lan and DeMets 1983
Lan KKG, DeMets DL. Discrete sequential boundaries for clinical trials. Biometrika 1983;70(3):659-63.

Loos et al 2008
Loos M, Giese NA, Kleeff J, Giese T, Gaida MM, Bergmann F, et al. Clinical significance and regulation of the costimulatory molecule B7-H1 in pancreatic adenocarcinoma. Cancer Lett. 2008;268(1):98-109.

McDermott 2009
McDermott DF. Immunotherapy of metastatic renal cell carcinoma. Cancer. 2009;115(10 suppl):2298–305.

Meyer et al 2014
Meyer C, Cagnon L, Costa-Nunes Cm, Baumgaaertner P, Montandon N, Leyvraz L, et al. Frequencies of circulating MDSC correlate with clinical outcome of melanoma patients treated with ipilimumab. Cancer Immunol Immunother 2014;63(3):247-57.

Mu et al 2011
Mu CY, Huang JA, Chen Y, Chen C, Zhang XG. High expression of PD-L1 in lung cancer may contribute to poor prognosis and tumor cells immune escape through suppressing tumor infiltrating dendritic cells maturation. Med Oncol. 2011 Sep;28(3):682-8.

Mu et al 2011
Mu CY, Huang JA, Chen Y, Chen C, Zhang XG. High expression of PD-L1 in lung cancer may contribute to poor prognosis and tumour cells immune escape through suppressing tumour infiltrating dendritic cells maturation. Med Oncol 2011;28(3):682-8.

Nakano et al 2001
Nakano O, Sato M, Naito Y, Suzuki K, Orikasa S, Aizawa M, et al. Proliferative activity of intratumoral $CD8^+$ T-lymphocytes as a prognostic factor in human renal cell carcinoma: clinicopathologic demonstration of antitumor immunity. Cancer Res. 2001;61:5132-6.

Narwal et al 2013
Narwal R, Roskos LK, Robbie GJ (2013) Population Pharmacokinetics of Sifalimumab, an Investigational Anti-Interferonalpha Monoclonal Antibody, in Systemic Lupus Erythematosus. Clin Pharmacokinet 52:1017–1027

Ng et al 2006
Ng CM, Lum BL, Gimenez V, et al. Rationale for fixed dosing of pertuzumab in cancer patients based on population pharmacokinetic analysis. Pharm Res. 2006;23(6):1275–84.

NCCN 2014
National Comprehensive Cancer Network Clinical Practice Guidelines in Oncology (NCCN Guidelines®). Non-Small Cell Lung Cancer. Version 4.2014. www.nccn.org.

Nishino et al 2013
Nishino M, Biobbie-Hurder A, Gargano M, Suda M, Ramaiya NH, Hodi FS. Developing a common language for tumor response to immunotherapy: immune-related response criteria using unidimensional measurements. Clin Cancer Res 2013;19(14):3936-43.

Okudaira et al 2009
Okudaira K, Hokari R, Tsuzuki Y, Okada Y, Komoto S, Watanabe C, et al. Blockade of B7-H1 or B7-DC induces an anti-tumour effect in a mouse pancreatic cancer model. Int J Oncol 2009;35(4):741-9.

Pardoll 2012
Pardoll DM. The blockade of immune checkpoints in cancer immunotherapy. Nat Rev Cancer 2012;12(4):252-64.

Paz-Ares et al 2013
Paz-Ares LG, de Marinis F, Dediu M, Thomas M, Pujol JL, Bidoli P, et al. PARAMOUNT: Final overall survival results of the phase III study of maintenance pemetrexed versus placebo immediately after induction treatment with pemetrexed plus cisplatin for advanced nonsquamous non-small-cell lung cancer. J Clin Oncol 2013;31(23):2895-902.

Peggs et al 2009
Peggs KS, Quezada SA, Allison JP. Cancer immunotherapy: co-stimulatory agonists and coinhibitory antagonists. Clin Exp Immunol 2009;157:9-19.

Pisters and LeChevalier 2005
Pisters KM, LeChevalier T. Adjuvant chemotherapy in completely resected non-small-cell lung cancer. J Clin Oncol 2005;23(14):3270-8.

Reck et al 2014
Reck M, Popat S, Reinmuth N, De Ruysscher D, Kerr KM, Peters S; ESMO Guidelines Working Group. Metastatic non-small-cell lung cancer (NSCLC): ESMO clinical practice guidelines for diagnosis, treatment and follow-up. Ann Oncol 2014;25(Suppl 3):iii27-39.

Ribas et al 2013
Ribas A, Kefford R, Marshall MA, Punt CJA, Haanen JB, Marmol M, et al. Phase III randomized clinical trial comparing tremelimumab with standard-of-care chemotherapy in patients with advanced melanoma. J Clin Oncol 2013;31:616-22.

Sandler et al 2006
Sandler A, Gray R, Perry MC, Brahmer J, Schiller JH, Dowlati A, et al. Paclitaxel-carboplatin alone or with bevacizumab for non-small-cell lung cancer. N Engl J Med 2006;355(24):2524-50.

Scagliotti et al 2008
Scagliotti GV, Parikh P, von Pawel J, Biesma B, Vansteenkiste J, Manegold C, et al. Phase III study comparing cisplatin plus gemcitabine with cisplatin plus pemetrexed in chemotherapy-naive patients with advanced-stage non-small-cell lung cancer. J Clin Oncol 2008;26(21)3543-51.

Schadendorf et al 2013
Schadendorf D, Hodi FS, Robert C et al. Pooled analysis of long-term survival data from phase II and phase III trials of ipilimumab in metastatic or locally advanced, unresectable melanoma. Presented at: European Cancer Congress 2013 (ECCO-ESMO-ESTRO); September 27-October 1, 2013; Amsterdam, The Netherlands. Abstract 24.

Schiller et al 2002
Schiller JH, Harringdon D, Belani CP, Langer C, Sandler A, Krook J, et al. Comparison of four chemotherapy regimens for advanced non-small-cell lung cancer. N Engl J Med 2002;10(346):92-8.

Schwarzenbach et al 2014
Schwarzenbach H, Nishida N, Calin GA, Pantel K. Clinical evidence of circulating cell-free microRNAs in cancer. Nat Rev Clin Oncol 2014;11(3):145-56.

Selke and Siegmund 1983
Selke T, Siegmund D. Sequential analysis of the proportional hazards model. Biometrika 1983;70:315-26.

Sun and Chen 2010
Sun X, Chen C. Comparison of Finkelstein's Method with the conventional approach for interval-censored data analysis. Stat Biopharm Res 2010;2(1):97-108.

Tarhini and Kirkwood 2008
Tarhini AA, Kirkwood JM. Tremelimumab (CP-675,206): a fully human anticytotoxic T lymphocyte-associated antigen 4 monoclonal antibody for treatment of patients with advanced cancers. Expert Opin Biol Ther 2008;8(10):1583-93.

Topalian et al 2012
Topalian SL, Hodi FS, Brahmer JR, Gettinger SN, Smith DC, McDermott DF, et al. Safety, activity, and immune correlates of anti-PD-1 antibody in cancer. N Engl J Med 2012;366(26):2443-54.

Topalian et al 2014
Topalian SL, Sznol M, McDermott DF, Kluger HM, Carvajal RD, Sharfman WH et al. Survival, durable tumor remission, and long-term safety in patients with advanced melanoma receiving nivolumab. J Clin Oncol, 2014 Apr 1; 32(10):1020-30.

Wang et al 2009
Wang DD, Zhang S, Zhao H, et al. Fixed dosing versus body size based dosing of monoclonal antibodies in adult clinical trials. J Clin Pharmacol. 2009;49(9):1012–24.

Wang et al 2013
Wang Z, Han J, Cui Y, Fan K, Zhou X. Circulating microRNA-21 as noninvasive predictive biomarker for response in cancer immunotherapy. Med Hypotheses 2013;81(1):41-3.

Wang et al 2014
Wang E, Kang D, et al. Population pharmacokinetic and pharmacodynamics analysis of tremelimumab in patients with metastatic melanoma. J Clin Pharmacol. 2014 Oct; 54(10):1108-16.

Weber et al 2012
Weber J, Kähler KC, Hauschild A. Management of immune-related adverse events and kinetics of response with ipilimumab. J Clin Oncol 2012;30(21):2691-7.

Whitehead and Whitehead 1991
Whitehead A, Whitehead J. A general parametric approach to the meta-analysis of randomized clinical trials. Stat Med 1991;10(11):1665-77.

Wolchok et al 2013
Wolchok JD, Kluger H, Callahan MK, Postow MA, Rizvi NA, Lesokhin AM, et al. Nivolumab plus ipilimumab in advanced melanoma. N Engl J Med 2013;369(2):122-33.

Yuan et al 2011
Yuan J, Adamow M, Ginsberg BA, Rasalan TS, Ritter E, Gallardo HF, et al. Integrated NY-ESO-1 antibody and CD8+ T-cell responses correlated with clinical benefit in advanced melanoma patients treated with ipilimumab. Proc Natl Acad Sci USA 2011;108(40):16723-8.

Zhang et al 2008
Zhang C, Wu S, Xue X, Li M, Qin X, Li W, et al. Anti-tumour immunotherapy by blockade of the

Zhang et al 2012
Zhang S, Shi R, Li C, et al. Fixed dosing versus body size-based dosing of therapeutic peptides and proteins in adults. J Clin Pharmacol. 2012;52(1):18–28.

Appendix 1. Dose modification and toxicity management guidelines for immune-mediated, infusion related, and non immuned-mediated reactions for the combination of durvalumab and tremelimumab

Dosing Modification and Toxicity Management Guidelines for Immune-Mediated, Infusion-Related, and Non-Immune–Mediated Reactions (MEDI4736 Monotherapy or Combination Therapy with Tremelimumab or Tremelimumab Monotherapy) 17 October 2019 Version (CTCAE v5.0)

General Considerations regarding Immune-Mediated Reactions

Dose Modifications	Toxicity Management
Drug administration modifications of study drug/study regimen will be made to manage potential immune-related AEs based on severity of treatment-emergent toxicities graded per NCI CTCAE v5.0.<br>In addition to the criteria for permanent discontinuation of study drug/study regimen based on CTC grade/severity (table below), permanently discontinue study drug/study regimen for the following conditions:<br>• Inability to reduce corticosteroid to a dose of ≤10 mg of prednisone per day (or equivalent) **within 12 weeks** of the start of the immune-mediated adverse event (imAE)<br>• Grade 3 recurrence of a previously experienced treatment-related imAE following resumption of dosing<br>**Grade 1** No dose modification<br>**Grade 2** Hold study drug/study regimen dose until Grade 2 resolution toGrade ≤1.<br>If toxicity worsens, then treat as Grade 3 or Grade 4.<br>Study drug/study regimen can be resumed once event stabilizes to Grade ≤1 after completion of steroid taper.<br>Patients with endocrinopathies who may require prolonged or continued steroid replacement can be retreated with study drug/study regimen on the following conditions:<br>1. The event stabilizes and is controlled.<br>2. The patient is clinically stable as per Investigator or treating physician's clinical judgement.<br>3. Doses of prednisone are at ≤10 mg/day or equivalent.	It is recommended that management of immune-mediated adverse events (imAEs) follows the guidelines presented in this table:<br>– It is possible that events with an inflammatory or immune mediated mechanism could occur in nearly all organs, some of them not noted specifically in these guidelines.<br>– Whether specific immune-mediated events (and/or laboratory indicators of such events) are noted in these guidelines or not, patients should be thoroughly evaluated to rule out any alternative etiology (e.g., disease progression, concomitant medications, and infections) to a possible immune-mediated event. In the absence of a clear alternative etiology, all such events should be managed as if they were immune related. General recommendations follow.<br>– Symptomatic and topical therapy should be considered for low-grade (Grade 1 or 2, unless otherwise specified) events.<br>– For persistent (>3 to 5 days) low-grade (Grade 2) or severe (Grade ≥3) events, promptly start prednisone 1 to 2 mg/kg/day PO or IV equivalent.<br>– Some events with high likelihood for morbidity and/or mortality – e.g., myocarditis, or other similar events even if they are not currently noted in the guidelines – should progress rapidly to high dose IV corticosteroids (methylprednisolone at 2 to 4 mg/kg/day) even if the event is Grade 2, and if clinical suspicion is high and/or there has been clinical confirmation. Consider, as necessary, discussing with the study physician, and promptly pursue specialist consultation.<br>– If symptoms recur or worsen during corticosteroid tapering (28 days of taper), increase the corticosteroid dose (prednisone dose [e.g., up to 2 to 4 mg/kg/day PO or IV equivalent]) until stabilization or improvement of symptoms, then resume corticosteroid tapering at a slower rate (>28 days of taper).<br>– More potent immunosuppressives such as TNF inhibitors (e.g., infliximab; also refer to the individual sections of the imAEs for specific type of immunosuppressive) should be considered for events not responding to

Dosing Modification and Toxicity Management Guidelines for Immune-Mediated, Infusion-Related, and Non-Immune–Mediated Reactions (MEDI4736 Monotherapy or Combination Therapy with Tremelimumab or Tremelimumab Monotherapy) 17 October 2019 Version (CTCAE v5.0)

General Considerations regarding Immune-Mediated Reactions

Dose Modifications	Toxicity Management
**Grade 3** Depending on the individual toxicity, study drug/study regimen may be permanently discontinued. Please refer to guidelines below.<br>**Grade 4** Permanently discontinue study drug/study regimen.<br>Note: For asymptomatic amylase or lipase levels of >2X ULN, hold study drug/study regimen, and if complete work up shows no evidence of pancreatitis, study drug/study regimen may be continued or resumed.<br>Note: Study drug/study regimen should be permanently discontinued in Grade 3 events with high likelihood for morbidity and/or mortality – e.g., myocarditis, or other similar events even if they are not currently noted in the guidelines. Similarly, consider whether study drug/study regimen should be permanently discontinued in Grade 2 events with high likelihood for morbidity and/or mortality – e.g., myocarditis, or other similar events even if they are not currently noted in the guidelines – when they do not rapidly improve to Grade <1 upon treatment with systemic steroids and following full taper<br>Note: There are some exceptions to permanent discontinuation of study drug for Grade 4 events (i.e., hyperthyroidism, hypothyroidism, Type 1 diabetes mellitus).	systemic steroids. Progression to use of more potent immunosuppressives should proceed more rapidly in events with high likelihood for morbidity and/or mortality – e.g., myocarditis, or other similar events even if they are not currently noted in the guidelines – when these events are not responding to systemic steroids.<br>– With long-term steroid and other immunosuppressive use, consider need for *Pneumocystis jirovecii* pneumonia (PJP, formerly known as *Pneumocystis carinii* pneumonia) prophylaxis, gastrointestinal protection, and glucose monitoring.<br>– Discontinuation of study drug/study regimen is not mandated for Grade 3/Grade 4 inflammatory reactions attributed to local tumor response (e.g., inflammatory reaction at sites of metastatic disease and lymph nodes). Continuation of study drug/study regimen in this situation should be based upon a benefit-risk analysis for that patient.

AE Adverse event; CTC Common Toxicity Criteria; CTCAE Common Terminology Criteria for Adverse Events; imAE immune-mediated adverse event; IV intravenous; NCI National Cancer Institute; PO By mouth.

Pediatric Considerations regarding Immune-Mediated Reactions	
**Dose Modifications**	**Toxicity Management**
The criteria for permanent discontinuation of study drug/study regimen based on CTC grade/severity is the same for pediatric patients as it is for adult patients, as well as to permanently discontinue study drug/study regimen if unable to reduce corticosteroid ≤ a dose equivalent to that required for corticosteroid replacement therapy **within 12 weeks** of the start of the immune-mediated event	– All recommendations for specialist consultation should occur with a pediatric specialist in the specialty recommended.<br>– The recommendations for dosing of steroids (i.e., mg/kg/day) and for IV IG and plasmapheresis that are provided for adult patients should also be used for pediatric patients.<br>– The infliximab 5 mg/kg IV dose recommended for adults is the same as recommended for pediatric patients ≥ 6 years old. For dosing in children younger than 6 years old, consult with a pediatric specialist.<br>– For pediatric dosing of mycophenolate mofetil, consult with a pediatric specialist.<br>– With long-term steroid and other immunosuppressive use, consider need for PJP prophylaxis, gastrointestinal protection, and glucose monitoring.

Specific Immune-Mediated Reactions

Adverse Events	Severity Grade of the Event (NCI CTCAE version 5.0)	Dose Modifications	Toxicity Management
**Pneumonitis/Interstitial Lung Disease (ILD)**	**Any Grade**	**General Guidance**	**For Any Grade:**<br>– Monitor patients for signs and symptoms of pneumonitis or ILD (new onset or worsening shortness of breath or cough). Patients should be evaluated with imaging and pulmonary function tests, including other diagnostic procedures as described below.<br>– Initial work-up may include clinical evaluation, monitoring of oxygenation via pulse oximetry (resting and exertion), laboratory work-up, and high- resolution CT scan.
	**Grade 1**<br>(asymptomatic, clinical or diagnostic observations only; intervention not indicated)	No dose modifications required. However, consider holding study drug/study regimen dose as clinically appropriate and during diagnostic work-up for other etiologies.	**For Grade 1 (radiographic changes only):**<br>– Monitor and closely follow up in 2 to 4 days for clinical symptoms, pulse oximetry (resting and exertion), and laboratory work-up and then as clinically indicated.<br>– Consider Pulmonary and Infectious Disease consults.
	**Grade 2**<br>(symptomatic; medical intervention indicated; limiting instrumental ADL)	Hold study drug/study regimen dose until Grade 2 resolution to Grade ≤1.<br>• If toxicity worsens, then treat as Grade 3 or Grade 4.<br>• If toxicity improves to Grade ≤1, then the decision to reinitiate study drug/study regimen will be based upon treating physician's clinical judgment and after completion of steroid taper.	**For Grade 2 (mild to moderate new symptoms):**<br>– Monitor symptoms daily and consider hospitalization.<br>– Promptly start systemic steroids (e.g., prednisone 1 to 2 mg/kg/day PO or IV equivalent).<br>– Reimage as clinically indicated.<br>– If no improvement within 3 to 5 days, additional workup should be considered and prompt treatment with IV methylprednisolone 2 to 4 mg/kg/day started<br>– If still no improvement within 3 to 5 days despite IV methylprednisolone at 2 to 4 mg/kg/day, promptly start immunosuppressive therapy such as TNF inhibitors (e.g., infliximab at 5 mg/kg every 2 weeks). Caution: It is

			important to rule out sepsis and refer to infliximab label for general guidance before using infliximab.<br>– Once the patient is improving, gradually taper steroids over ≥28 days and consider prophylactic antibiotics, antifungals, or anti-PJP treatment (refer to current NCCN guidelines for treatment of cancer-related infections)[a]<br>– Consider Pulmonary and Infectious Disease consults.<br>– Consider, as necessary, discussing with study physician.
	**Grade 3 or 4**<br>(Grade 3: severe symptoms; limiting self-care ADL; oxygen indicated)<br><br>(Grade 4: life-threatening respiratory compromise; urgent intervention indicated [e.g., tracheostomy or intubation])	Permanently discontinue study drug/study regimen.	**For Grade 3 or 4 (severe or new symptoms, new/worsening hypoxia, life-threatening):**<br>– Promptly initiate empiric IV methylprednisolone 1 to 4 mg/kg/day or equivalent.<br>– Obtain Pulmonary and Infectious Disease consults; consider, as necessary, discussing with study physician.<br>– Hospitalize the patient.<br>– Supportive care (e.g., oxygen).<br>– If no improvement within 3 to 5 days, additional workup should be considered and prompt treatment with additional immunosuppressive therapy such as TNF inhibitors (e.g., infliximab at 5 mg/kg every 2 weeks' dose) started. Caution: rule out sepsis and refer to infliximab label for general guidance before using infliximab.<br>– Once the patient is improving, gradually taper steroids over ≥28 days and consider prophylactic antibiotics, antifungals, and, in particular, anti-PJP treatment (refer to current NCCN guidelines for treatment of cancer-related infections).[a]
**Diarrhea/Colitis**<br>**Large intestine perforation/Intestine perforation**	**Any Grade**	**General Guidance**	**For Any Grade:**<br>– Monitor for symptoms that may be related to diarrhea/enterocolitis (abdominal pain, cramping, or changes in bowel habits such as increased frequency over baseline or blood in stool) or related to bowel perforation (such as sepsis, peritoneal signs, and ileus).<br>– When symptoms or evaluation indicate a perforation is suspected, consult a surgeon experienced in abdominal surgery immediately without any delay.

		– Patients should be thoroughly evaluated to rule out any alternative etiology (e.g., disease progression, other medications, or infections), including testing for clostridium difficile toxin, etc.<br>– Steroids should be considered in the absence of clear alternative etiology, even for low-grade events, in order to prevent potential progression to higher grade event, including perforation.<br>– Use analgesics carefully; they can mask symptoms of perforation and peritonitis.
**Grade 1**<br>(Diarrhea: stool frequency of <4 over baseline per day)<br>(Colitis: asymptomatic; clinical or diagnostic observations only; intervention not indicated)	No dose modifications.	**For Grade 1:**<br>– Monitor closely for worsening symptoms.<br>– Consider symptomatic treatment, including hydration, electrolyte replacement, dietary changes (e.g., American Dietetic Association colitis diet), and loperamide. Use probiotics as per treating physician's clinical judgment.
**Grade 2**<br>(Diarrhea: stool frequency of 4 to 6 over baseline per day; limiting instrumental ADL)<br>(Colitis: abdominal pain; mucus or blood in stool)	Hold study drug/study regimen until resolution to Grade ≤1<br>• If toxicity worsens, then treat as Grade 3 or Grade 4.<br>• If toxicity improves to Grade ≤1, then study drug/study regimen can be resumed after completion of steroid taper.	**For Grade 2:**<br>– Consider symptomatic treatment, including hydration, electrolyte replacement, dietary changes (e.g., American Dietetic Association colitis diet), and loperamide and/or budesonide.<br>– Promptly start prednisone 1 to 2 mg/kg/day PO or IV equivalent.<br>– If event is not responsive within 3 to 5 days or worsens despite prednisone at 1 to 2 mg/kg/day PO or IV equivalent, GI consult should be obtained for consideration of further workup, such as imaging and/or colonoscopy, to confirm colitis and rule out perforation, and prompt treatment with IV methylprednisolone 2 to 4 mg/kg/day started.<br>– If still no improvement within 3 to 5 days despite 2 to 4 mg/kg IV methylprednisolone, promptly start immunosuppressives

(Perforation: invasive intervention not indicated)		such as infliximab at 5 mg/kg once every 2 weeks[a]. **Caution:** it is important to rule out bowel perforation and refer to infliximab label for general guidance before using infliximab.<br>– Consider, as necessary, discussing with study physician if no resolution to Grade ≤1 in 3 to 4 days.<br>– Once the patient is improving, gradually taper steroids over ≥28 days and consider prophylactic antibiotics, antifungals, and anti-PJP treatment (refer to current NCCN guidelines for treatment of cancer-related infections).[a]
**Grade 3 or 4**<br>(Grade 3 Diarrhea: stool frequency of ≥7 over baseline per day; limiting self care ADL; Grade 4 Diarrhea: life threatening consequences)<br>(Grade 3 Colitis: severe abdominal pain, fever; ileus; peritoneal signs; Grade 4 Colitis: life-threatening consequences, urgent intervention indicated)<br>(Grade 3 Perforation: invasive intervention indicated;<br>Grade 4 Perforation: life-threatening consequences; urgent intervention indicated)	**Grade 3**<br>Permanently discontinue study drug/study regimen for Grade 3 if toxicity does not improve to Grade ≤1 within 14 days; study drug/study regimen can be resumed after completion of steroid taper.<br>**Grade 4**<br>Permanently discontinue study drug/study regimen.	**For Grade 3 or 4:**<br>– Promptly initiate empiric IV methylprednisolone 2 to 4 mg/kg/day or equivalent.<br>– Monitor stool frequency and volume and maintain hydration.<br>– Urgent GI consult and imaging and/or colonoscopy as appropriate.<br>– If still no improvement within 3 to 5 days of IV methylprednisolone 2 to 4 mg/kg/day or equivalent, promptly start further immunosuppressives (e.g., infliximab at 5 mg/kg once every 2 weeks). **Caution**: Ensure GI consult to rule out bowel perforation and refer to infliximab label for general guidance before using infliximab. If perforation is suspected, consult a surgeon experienced in abdominal surgery immediately without any delay .<br>– Once the patient is improving, gradually taper steroids over ≥28 days and consider prophylactic antibiotics, antifungals, and anti-PJP treatment (refer to current NCCN guidelines for treatment of cancer-related infections).[a]

**Hepatitis (elevated LFTs)**	**Any Grade**	**General Guidance**	**For Any Grade**
Infliximab should not be used for management of immune-related hepatitis.<br><br>**PLEASE SEE shaded area immediately below this section to find guidance for management of "Hepatitis (elevated LFTS)" in HCC patients**			– Monitor and evaluate liver function test: AST, ALT, ALP, and TB.<br>– Evaluate for alternative etiologies (e.g., viral hepatitis, disease progression, concomitant medications).
	**Grade 1**<br>**(**AST or ALT >ULN and ≤3.0×ULN if baseline normal, 1.5-3.0×baseline if baseline abnormal; and/or TB >ULN and ≤1.5×ULN if baseline normal, >1.0-1.5×baseline if baseline abnormal)	• No dose modifications.<br>• If it worsens, then treat as Grade 2.	– Continue LFT monitoring per protocol.
	**Grade 2**<br>(AST or ALT >3.0×ULN and ≤5.0×ULN if baseline normal, >3-5×baseline if baseline abnormal; and/or TB >1.5×ULN and ≤3.0×ULN if baseline normal, >1.5-3.0×baseline if baseline abnormal)	• Hold study drug/study regimen dose until resolution to Grade≤1.<br>• If toxicity worsens, then treat as Grade 3.<br>• If toxicity improves to Grade≤1, resume study drug/study regimen after completion of steroid taper.	**For Grade 2:**<br>– Regular and frequent checking of LFTs (e.g., every 1 to 2 days) until elevations of these are improving or resolved.<br>– If no resolution to ≤Grade 1 in 1 to 2 days, consider, as necessary, discussing with study physician.<br>– If event is persistent (>3 to 5 days) or worsens, promptly start prednisone 1 to 2 mg/kg/day PO or IV equivalent.<br>– If still no improvement within 3 to 5 days despite 1 to 2 mg/kg/day of prednisone PO or IV equivalent, consider additional work up and start prompt treatment with IV methylprednisolone 2 to 4 mg/kg/day.<br>– If still no improvement within 3 to 5 days despite 2 to 4 mg/kg/day of IV methylprednisolone, promptly start immunosuppressives (i.e., mycophenolate mofetil).[a] Discuss with study physician if mycophenolate mofetil is not available. **Infliximab should NOT be used.**<br>– Once the patient is improving, gradually taper steroids over ≥28 days and consider prophylactic antibiotics, antifungals,

		and anti-PJP treatment (refer to current NCCN guidelines for treatment of cancer-related infections).[a]
**Grade 3**<br>(AST or ALT >5.0×ULN and ≤20×ULN if baseline normal, >5-20× baseline if baseline abnormal; and/or TB >3.0×ULN and ≤10.0×ULN if baseline normal, >3.0-10.0× baseline if baseline abnormal)	For elevations in transaminases ≤8×ULN, or elevations in TB ≤5×ULN:<br>• Hold study drug/study regimen dose until resolution to Grade≤1<br>• Resume study drug/study regimen if elevations downgrade to Grade≤1 within 14 days and after completion of steroid taper.<br>• Permanently discontinue study drug/study regimen if the elevations do not downgrade to Grade≤1 within 14 days.<br>For elevations in transaminases >8×ULN or elevations in bilirubin >5×ULN, permanently discontinue study drug/study regimen.	**For Grade 3 or 4:**<br>– Promptly initiate empiric IV methylprednisolone at 1 to 4 mg/kg/day or equivalent.<br>– If still no improvement within 3 to 5 days despite 1 to 4 mg/kg/day methylprednisolone IV or equivalent, promptly start treatment with immunosuppressive therapy (i.e., mycophenolate mofetil). Discuss with study physician if mycophenolate is not available. **Infliximab should NOT be used.**<br>– Request Hepatology consult, and perform abdominal workup and imaging as appropriate.<br>– Once the patient is improving, gradually taper steroids over ≥28 days and consider prophylactic antibiotics, antifungals, and anti-PJP treatment (refer to current NCCN guidelines for treatment of cancer-related infections).[a]
**Grade 4**<br>(AST or ALT >20×ULN if baseline normal, >20×baseline if baseline abnormal; and/or TB >10×ULN if baseline normal, >10.0×baseline if baseline abnormal)	Permanently discontinue study drug/study regimen for any case meeting Hy's law criteria (AST and/or ALT >3×ULN + bilirubin >2×ULN without initial findings of cholestasis [i.e., elevated alkaline P04] and in the absence of any alternative cause).[b]	

**Hepatitis (elevated LFTs)**<br>Infliximab should not be used for management of immune-related hepatitis.<br>**THIS shaded area is guidance *only* for management of "Hepatitis (elevated LFTs)" in HCC patients**<br>See instructions at bottom of shaded area if transaminase rise is not isolated but (at any time) occurs in setting of either **increasing bilirubin or signs of DILI/liver decompensation**	**Any Elevations of AST, ALT, or TB as Described Below**	**General Guidance**	**For Any Elevations Described:**<br>– Monitor and evaluate liver function test: AST, ALT, ALP, and TB.<br>– Evaluate for alternative etiologies (e.g., viral hepatitis, disease progression, concomitant medications, worsening of liver cirrhosis [e.g., portal vein thrombosis]).<br>– For HBV+ patients: evaluate quantitative HBV viral load, quantitative HBsAg, or HBeAg<br>– For HCV+ patients: evaluate quantitative HCV viral load<br>– Consider consulting hepatologist/Infectious Disease specialist regarding change/implementation in/of antiviral medications for any patient with an elevated HBV viral load >2000 IU/ml<br>– Consider consulting hepatologist/Infectious Disease specialist regarding change/implementation in/of antiviral HCV medications if HCV viral load increased by ≥2-fold<br>– For HCV+ with HBcAB+: Evaluate for both HBV and HCV as above
	**Isolated AST or ALT >ULN and ≤5.0×ULN, whether normal or elevated at baseline**	• No dose modifications.<br>• If ALT/AST elevations represents significant worsening based on investigator assessment, then treat as described for elevations in the row below.<br>For all transaminase elevations, see instructions at bottom of shaded area if transaminase rise is not isolated but (at any time) occurs in setting of either **increasing bilirubin or signs of DILI/liver decompensation**	

	**Isolated AST or ALT >5.0×ULN and ≤8.0×ULN, if normal at baseline**<br><br>**Isolated AST or ALT >2.0×baseline and ≤12.5×ULN, if elevated >ULN at baseline**	• Hold study drug/study regimen dose until resolution to AST or ALT ≤5.0×ULN.<br>• If toxicity worsens, then treat as described for elevations in the rows below.<br>If toxicity improves to AST or ALT ≤5.0×ULN, resume study drug/study regimen after completion of steroid taper.	– Regular and frequent checking of LFTs (e.g., every 1 to 3 days) until elevations of these are improving or resolved.<br>– Recommend consult hepatologist; consider abdominal ultrasound, including Doppler assessment of liver perfusion.<br>– Consider, as necessary, discussing with study physician.<br>– If event is persistent (>3 to 5 days) or worsens, and investigator suspects toxicity to be immune-mediated AE, recommend to start prednisone 1 to 2 mg/kg/day PO or IV equivalent.<br>– If still no improvement within 3 to 5 days despite 1 to 2 mg/kg/day of prednisone PO or IV equivalent, consider additional workup and treatment with IV methylprednisolone 2 to 4 mg/kg/day.<br>– If still no improvement within 3 to 5 days despite 2 to 4 mg/kg/day of IV methylprednisolone, consider additional abdominal workup (including liver biopsy) and imaging (i.e., liver ultrasound), and consider starting immunosuppressives (i.e., mycophenolate mofetil).[a] Discuss with study physician if mycophenolate mofetil is not available. **Infliximab should NOT be used.**
	**Isolated AST or ALT >8.0×ULN and ≤20.0×ULN, if normal at baseline**<br><br>**Isolated AST or ALT >12.5×ULN and ≤20.0×ULN, if elevated >ULN at baseline**	• Hold study drug/study regimen dose until resolution to AST or ALT ≤5.0×ULN.<br>• Resume study drug/study regimen if elevations downgrade to AST or ALT ≤5.0×ULN within 14 days and after completion of steroid taper.<br>• Permanently discontinue study drug/study regimen if the elevations do not downgrade to AST or ALT ≤5.0×ULN within 14 days<br>Permanently discontinue study drug/study regimen for any case meeting Hy's law criteria, in the absence of any alternative cause.[b]	– Regular and frequent checking of LFTs (e.g., every 1-2 days) until elevations of these are improving or resolved.<br>– Consult hepatologist (unless investigator is hepatologist); obtain abdominal ultrasound, including Doppler assessment of liver perfusion; and consider liver biopsy.<br>– Consider, as necessary, discussing with study physician.<br>– If investigator suspects toxicity to be immune-mediated, promptly initiate empiric IV methylprednisolone at 1 to 4 mg/kg/day or equivalent.<br>– If no improvement within 3 to 5 days despite 1 to 4 mg/kg/day methylprednisolone IV or equivalent, obtain liver biopsy (if it has not been done already) and promptly start treatment with immunosuppressive therapy (mycophenolate mofetil). Discuss with study physician if mycophenolate is not available. **Infliximab should NOT be used.**

			– Once the patient is improving, gradually taper steroids over ≥28 days and consider prophylactic antibiotics, antifungals, and anti-PCP treatment (refer to current NCCN guidelines for treatment of cancer-related infections).[a]
	**Isolated AST or ALT >20×ULN, whether normal or elevated at baseline**	Permanently discontinue study drug/study regimen.	**Same as above**<br>**(except would recommend obtaining liver biopsy early)**
**If transaminase rise is not isolated but (at any time) occurs in setting of either increasing total/direct bilirubin (≥1.5×ULN, if normal at baseline; or 2×baseline, if >ULN at baseline) or signs of DILI/liver decompensation (e.g., fever, elevated INR):**<br>- **Manage dosing for each level of transaminase rise as instructed for the next highest level of transaminase rise**<br>- **For example, manage dosing for second level of transaminase rise (i.e., AST or ALT >5.0×ULN and ≤8.0×ULN, if normal at baseline, or AST or ALT >2.0×baseline and ≤12.5×ULN, if elevated >ULN at baseline) as instructed for the third level of transaminase rise (i.e., AST or ALT >8.0×ULN and ≤20.0×ULN, if normal at baseline, or AST or ALT >12.5×ULN and ≤20.0×ULN, if elevated >ULN at baseline)**<br>- **For the third and fourth levels of transaminase rises, permanently discontinue study drug/study regimen**			
**Nephritis or renal dysfunction**<br>(elevated serum creatinine)	**Any Grade**	**General Guidance**	**For Any Grade:**<br>– Consult with nephrologist.<br>– Monitor for signs and symptoms that may be related to changes in renal function (e.g., routine urinalysis, elevated serum BUN and creatinine, decreased creatinine clearance, electrolyte imbalance, decrease in urine output, or proteinuria).<br>– Patients should be thoroughly evaluated to rule out any alternative etiology (e.g., disease progression or infections).<br>– Steroids should be considered in the absence of clear alternative etiology even for low-grade events (Grade 2), in order to prevent potential progression to higher grade event.

**Grade 1**<br>(serum creatinine > ULN to 1.5 × ULN)	No dose modifications.	**For Grade 1:**<br>– Monitor serum creatinine weekly and any accompanying symptoms.<br>• If creatinine returns to baseline, resume its regular monitoring per study protocol.<br>• If creatinine worsens, depending on the severity, treat as Grade 2, 3, or 4.<br>– Consider symptomatic treatment, including hydration, electrolyte replacement, and diuretics.<br>– If baseline serum creatinine is elevated above normal, and there is a rise to > 1 to 1.5 × baseline, consider following recommendations in this row.
**Grade 2**<br>(serum creatinine >1.5 to 3.0 × baseline; >1.5 to 3.0 × ULN)	Hold study drug/study regimen until resolution to Grade ≤1 or baseline.<br>• If toxicity worsens, then treat as Grade 3 or 4.<br>• If toxicity improves to Grade ≤1 or baseline, then resume study drug/study regimen after completion of steroid taper.	**For Grade 2:**<br>– Consider symptomatic treatment, including hydration, electrolyte replacement, and diuretics.<br>– Carefully monitor serum creatinine every 2 to 3 days and as clinically warranted.<br>– Consult nephrologist and consider renal biopsy if clinically indicated.<br>– If event is persistent (>3 to 5 days) or worsens, promptly start prednisone 1 to 2 mg/kg/day PO or IV equivalent.<br>– If event is not responsive within 3 to 5 days or worsens despite prednisone at 1 to 2 mg/kg/day PO or IV equivalent, additional workup should be considered and prompt treatment with IV methylprednisolone at 2 to 4 mg/kg/day started.<br>– Once the patient is improving, gradually taper steroids over ≥28 days and consider prophylactic antibiotics, antifungals, and anti-PJP treatment (refer to current NCCN guidelines for treatment of cancer-related infections).[a]<br>– When event returns to baseline, resume study drug/study regimen and routine serum creatinine monitoring per study protocol.

	**Grade 3 or 4**<br>(Grade 3: serum creatinine >3.0 × baseline; >3.0 to 6.0 × ULN)<br><br>(Grade 4: serum creatinine >6.0 × ULN)	Permanently discontinue study drug/study regimen.	**For Grade 3 or 4:**<br>– Carefully monitor serum creatinine on daily basis.<br>– Consult nephrologist and consider renal biopsy if clinically indicated.<br>– Promptly start prednisone 1 to 2 mg/kg/day PO or IV equivalent.<br>– If event is not responsive within 3 to 5 days or worsens despite prednisone at 1 to 2 mg/kg/day PO or IV equivalent, additional workup should be considered and prompt treatment with IV methylprednisolone 2 to 4 mg/kg/day started.<br>– Once the patient is improving, gradually taper steroids over ≥28 days and consider prophylactic antibiotics, antifungals, and anti-PJP treatment (refer to current NCCN guidelines for treatment of cancer-related infections).[a]
**Rash or Dermatitis (including Pemphigoid)**	**Any Grade**<br>(refer to NCI CTCAE v 5.0 for definition of severity/grade depending on type of skin rash)	**General Guidance**	**For Any Grade:**<br>– Monitor for signs and symptoms of dermatitis (rash and pruritus).<br>– IF THERE IS ANY BULLOUS FORMATION, THE STUDY PHYSICIAN SHOULD BE CONTACTED **AND STUDY DRUG DISCONTINUED IF SUSPECT STEVENS-JOHNSON SYNDROME OR TOXIC EPIDERMAL NECROLYSIS.**
	**Grade 1**	No dose modifications.	**For Grade 1:**<br>– Consider symptomatic treatment, including oral antipruritics (e.g., diphenhydramine or hydroxyzine) and topical therapy (e.g., urea cream).
	**Grade 2**	For persistent (>1 to 2 weeks) Grade 2 events, hold scheduled study drug/study regimen until resolution to Grade ≤1 or baseline.<br>• If toxicity worsens, then treat as Grade 3.	**For Grade 2:**<br>– Obtain Dermatology consult.<br>– Consider symptomatic treatment, including oral antipruritics (e.g., diphenhydramine or hydroxyzine) and topical therapy (e.g., urea cream).<br>– Consider moderate-strength topical steroid.

		• If toxicity improves to Grade ≤1 or baseline, then resume drug/study regimen after completion of steroid taper.	– If no improvement of rash/skin lesions occurs within 3 to 5 days or is worsening despite symptomatic treatment and/or use of moderate strength topical steroid, consider, as necessary, discussing with study physician and promptly start systemic steroids such as prednisone 1 to 2 mg/kg/day PO or IV equivalent. If > 30% body surface area is involved, consider initiation of systemic steroids promptly.<br>– Consider skin biopsy if the event is persistent for >1 to 2 weeks or recurs.
	**Grade 3 or 4**	**For Grade 3:**<br>Hold study drug/study regimen until resolution to Grade ≤1 or baseline.<br>If temporarily holding the study drug/study regimen does not provide improvement of the Grade 3 skin rash to Grade ≤1 or baseline within 30 days, then permanently discontinue study drug/study regimen.<br>**For Grade 4 (or life-threatening):**<br>Permanently discontinue study drug/study regimen.	**For Grade 3 or 4 (or life-threatening):**<br>– Consult Dermatology.<br>– Promptly initiate empiric IV methylprednisolone 1 to 4 mg/kg/day or equivalent.<br>– Consider hospitalization.<br>– Monitor extent of rash [Rule of Nines].<br>– Consider skin biopsy (preferably more than 1) as clinically feasible.<br>– Once the patient is improving, gradually taper steroids over ≥28 days and consider prophylactic antibiotics, antifungals, and anti-PJP treatment (refer to current NCCN guidelines for treatment of cancer-related infections).[a]<br>– Consider, as necessary, discussing with study physician.
**Endocrinopathy**<br>(e.g., hyperthyroidism, thyroiditis, hypothyroidism, Type 1 diabetes mellitus,	**Any Grade**<br>(depending on the type of endocrinopathy, refer to NCI CTCAE	**General Guidance**	**For Any Grade:**<br>– Consider consulting an endocrinologist for endocrine events.<br>– Consider, as necessary, discussing with study physician.<br>– Monitor patients for signs and symptoms of endocrinopathies. Non-specific symptoms include headache, fatigue, behavior changes, changed mental status, vertigo, abdominal pain,

hypophysitis, hypopituitarism, and adrenal insufficiency; exocrine event of amylase/lipase increased also included in this section)	v5.0 for defining the CTC grade/severity)		unusual bowel habits, polydipsia, polyuria, hypotension, and weakness.<br>– Patients should be thoroughly evaluated to rule out any alternative etiology (e.g., disease progression including brain metastases, or infections).<br>– Depending on the suspected endocrinopathy, monitor and evaluate thyroid function tests: TSH, free T3 and free T4 and other relevant endocrine and related labs (e.g., blood glucose and ketone levels, HgA1c).<br>– For asymptomatic elevations in serum amylase and lipase >ULN and <3×ULN, corticosteroid treatment is not indicated as long as there are no other signs or symptoms of pancreatic inflammation.<br>– If a patient experiences an AE that is thought to be possibly of autoimmune nature (e.g., thyroiditis, pancreatitis, hypophysitis, or diabetes insipidus), the investigator should send a blood sample for appropriate autoimmune antibody testing.
	**Grade 1**	No dose modifications.	**For Grade 1 (including those with asymptomatic TSH elevation):**<br>– Monitor patient with appropriate endocrine function tests.<br>– For suspected hypophysitis/hypopituitarism, consider consultation of an endocrinologist to guide assessment of early-morning ACTH, cortisol, TSH and free T4; also consider gonadotropins, sex hormones, and prolactin levels, as well as cosyntropin stimulation test (though it may not be useful in diagnosing early secondary adrenal insufficiency).<br>– If TSH < 0.5 × LLN, or TSH >2 × ULN, or consistently out of range in 2 subsequent measurements, include free T4 at subsequent cycles as clinically indicated and consider consultation of an endocrinologist.
	**Grade 2**	For Grade 2 endocrinopathy other than hypothyroidism and Type 1 diabetes mellitus, hold study drug/study regimen dose until patient is clinically stable.<br>• If toxicity worsens, then treat as Grade 3 or Grade 4.	**For Grade 2 (including those with symptomatic endocrinopathy):**<br>– Consult endocrinologist to guide evaluation of endocrine function and, as indicated by suspected endocrinopathy and as clinically indicated, consider pituitary scan.<br>– For all patients with abnormal endocrine work up, except those with isolated hypothyroidism or Type 1 DM, and as guided by an endocrinologist, consider short-term corticosteroids (e.g., 1

	Study drug/study regimen can be resumed once event stabilizes and after completion of steroid taper.<br><br>Patients with endocrinopathies who may require prolonged or continued steroid replacement (e.g., adrenal insufficiency) can be retreated with study drug/study regimen on the following conditions:<br>1. The event stabilizes and is controlled.<br>2. The patient is clinically stable as per investigator or treating physician's clinical judgement.<br>3. Doses of prednisone are ≤10 mg/day or equivalent.	to 2 mg/kg/day methylprednisolone or IV equivalent) and prompt initiation of treatment with relevant hormone replacement (e.g., hydrocortisone, sex hormones).<br>– Isolated hypothyroidism may be treated with replacement therapy, without study drug/study regimen interruption, and without corticosteroids.<br>– Isolated Type 1 diabetes mellitus (DM) may be treated with appropriate diabetic therapy, without study drug/study regimen interruption, and without corticosteroids.<br>– Once patients on steroids are improving, gradually taper immunosuppressive steroids (as appropriate and with guidance of endocrinologist) over ≥28 days and consider prophylactic antibiotics, antifungals, and anti-PJP treatment (refer to current NCCN guidelines for treatment of cancer-related infections).[a]<br>– For patients with normal endocrine workup (laboratory assessment or MRI scans), repeat laboratory assessments/MRI as clinically indicated.
**Grade 3 or 4**	For Grade 3 or 4 endocrinopathy other than hypothyroidism and Type 1 diabetes mellitus, hold study drug/study regimen dose until endocrinopathy symptom(s) are controlled.<br><br>Study drug/study regimen can be resumed once event stabilizes and after completion of steroid taper.<br><br>Patients with endocrinopathies who may require prolonged or continued steroid replacement (e.g., adrenal insufficiency) can be retreated with study drug/study regimen on the following conditions:<br>1. The event stabilizes and is controlled.	**For Grade 3 or 4:**<br>– Consult endocrinologist to guide evaluation of endocrine function and, as indicated by suspected endocrinopathy and as clinically indicated, consider pituitary scan. Hospitalization recommended.<br>– For all patients with abnormal endocrine work up, except those with isolated hypothyroidism or Type 1 DM, and as guided by an endocrinologist, promptly initiate empiric IV methylprednisolone 1 to 2 mg/kg/day or equivalent, as well as relevant hormone replacement (e.g., hydrocortisone, sex hormones).<br>– For adrenal crisis, severe dehydration, hypotension, or shock, immediately initiate IV corticosteroids with mineralocorticoid activity.<br>– Isolated hypothyroidism may be treated with replacement therapy, without study drug/study regimen interruption, and without corticosteroids.<br>– Isolated Type 1 diabetes mellitus may be treated with appropriate diabetic therapy, without study drug/study regimen interruption, and without corticosteroids.

		2. The patient is clinically stable as per investigator or treating physician's clinical judgement.<br>3. Doses of prednisone are ≤10 mg/day or equivalent.	– Once patients on steroids are improving, gradually taper immunosuppressive steroids (as appropriate and with guidance of endocrinologist) over ≥28 days and consider prophylactic antibiotics, antifungals, and anti-PJP treatment (refer to current NCCN guidelines for treatment of cancer-related infections).[a]
**Neurotoxicity**<br>(to include but not be limited to limbic encephalitis and autonomic neuropathy, excluding Myasthenia Gravis and Guillain-Barre)	**Any Grade**<br>(depending on the type of neurotoxicity, refer to NCI CTCAE v5.0 for defining the CTC grade/severity)	**General Guidance**	**For Any Grade:**<br>– Patients should be evaluated to rule out any alternative etiology (e.g., disease progression, infections, metabolic syndromes, or medications).<br>– Monitor patient for general symptoms (headache, nausea, vertigo, behavior change, or weakness).<br>– Consider appropriate diagnostic testing (e.g., electromyogram and nerve conduction investigations).<br>– Perform symptomatic treatment with Neurology consult as appropriate.<br>–
	**Grade 1**	No dose modifications.	**For Grade 1:**<br>– See "Any Grade" recommendations above.<br>– Treat mild signs/symptoms as Grade 1 (e.g. loss of deep tendon reflexes or paresthesia)
	**Grade 2**	For acute motor neuropathies or neurotoxicity, hold study drug/study regimen dose until resolution to Grade ≤1.<br>For sensory neuropathy/neuropathic pain, consider holding study drug/study regimen dose until resolution to Grade ≤1.<br>If toxicity worsens, then treat as Grade 3 or 4.	**For Grade 2:**<br>– Consider, as necessary, discussing with the study physician.<br>– Obtain Neurology consult.<br>– Sensory neuropathy/neuropathic pain may be managed by appropriate medications (e.g., gabapentin or duloxetine).<br>– Promptly start systemic steroids prednisone 1 to 2 mg/kg/day PO or IV equivalent.<br>– If no improvement within 3 to 5 days despite 1 to 2 mg/kg/day prednisone PO or IV equivalent, consider additional workup and promptly treat with additional immunosuppressive therapy (e.g., IV IG).

		Study drug/study regimen can be resumed once event improves to Grade ≤1 and after completion of steroid taper.	
	**Grade 3 or 4**	**For Grade 3:**<br>Hold study drug/study regimen dose until resolution to Grade ≤1.<br>Permanently discontinue study drug/study regimen if Grade 3 imAE does not resolve to Grade ≤1 within 30 days.<br>**For Grade 4:**<br>Permanently discontinue study drug/study regimen.	**For Grade 3 or 4:**<br>– Consider, as necessary, discussing with study physician.<br>– Obtain Neurology consult.<br>– Consider hospitalization.<br>– Promptly initiate empiric IV methylprednisolone 1 to 2 mg/kg/day or equivalent.<br>– If no improvement within 3 to 5 days despite IV corticosteroids, consider additional workup and promptly treat with additional immunosuppressants (e.g., IV IG).<br>– Once stable, gradually taper steroids over ≥28 days.
**Peripheral neuromotor syndromes**<br>(such as Guillain-Barre and myasthenia gravis)	**Any Grade**	**General Guidance**	**For Any Grade:**<br>– The prompt diagnosis of immune-mediated peripheral neuromotor syndromes is important, since certain patients may unpredictably experience acute decompensations that can result in substantial morbidity or in the worst case, death. Special care should be taken for certain sentinel symptoms that may predict a more severe outcome, such as prominent dysphagia, rapidly progressive weakness, and signs of respiratory insufficiency or autonomic instability.<br>– Patients should be evaluated to rule out any alternative etiology (e.g., disease progression, infections, metabolic syndromes or medications). It should be noted that the diagnosis of immune-mediated peripheral neuromotor syndromes can be particularly challenging in patients with underlying cancer, due to the multiple potential confounding effects of cancer (and its treatments) throughout the neuraxis. Given the importance of prompt and accurate diagnosis, it is essential to have a low threshold to obtain a Neurology consult.

		– Neurophysiologic diagnostic testing (e.g., electromyogram and nerve conduction investigations, and "repetitive stimulation" if myasthenia is suspected) are routinely indicated upon suspicion of such conditions and may be best facilitated by means of a Neurology consultation.<br>– It is important to consider that the use of steroids as the primary treatment of Guillain-Barre is not typically considered effective. Patients requiring treatment should be started with IV IG and followed by plasmapheresis if not responsive to IV IG.
**Grade 1**<br>(Guillain-Barre [GB]: mild symptoms)<br>(Myasthenia gravis [MG]: asymptomatic or mild symptoms; clinical or diagnostic observations only; intervention not indicated)	No dose modifications.	**For Grade 1:**<br>– Consider, as necessary, discussing with the study physician.<br>– Care should be taken to monitor patients for sentinel symptoms of a potential decompensation as described above.<br>– Obtain a Neurology consult.
**Grade 2**<br>(GB: moderate symptoms; limiting instrumental ADL)<br>(MG: moderate; minimal, local or noninvasive intervention indicated; limiting age-	Hold study drug/study regimen dose until resolution to Grade ≤1.<br>Permanently discontinue study drug/study regimen if it does not resolve to Grade ≤1 within 30 days or if there are signs of respiratory insufficiency or autonomic instability.	**For Grade 2:**<br>– Consider, as necessary, discussing with the study physician.<br>– Care should be taken to monitor patients for sentinel symptoms of a potential decompensation as described above.<br>– Obtain a Neurology consult<br>– Sensory neuropathy/neuropathic pain may be managed by appropriate medications (e.g., gabapentin or duloxetine).<br>*MYASTHENIA GRAVIS:*<br>○ Steroids may be successfully used to treat myasthenia gravis. It is important to consider that steroid therapy (especially with high doses) may result in transient worsening of myasthenia and

appropriate instrumental ADL)		should typically be administered in a monitored setting under supervision of a consulting neurologist.<br>○ Patients unable to tolerate steroids may be candidates for treatment with plasmapheresis or IV IG. Such decisions are best made in consultation with a neurologist, taking into account the unique needs of each patient.<br>○ If myasthenia gravis-like neurotoxicity is present, consider starting AChE inhibitor therapy in addition to steroids. Such therapy, if successful, can also serve to reinforce the diagnosis.<br>*GUILLAIN-BARRE:*<br>○ It is important to consider here that the use of steroids as the primary treatment of Guillain-Barre is not typically considered effective.<br>○ Patients requiring treatment should be started with IV IG and followed by plasmapheresis if not responsive to IV IG.
**Grade 3 or 4**<br>(Grade 3 GB: severe symptoms; limiting self care ADL;<br>Grade 4 GB: life-threatening consequences; urgent intervention indicated; intubation)<br>(Grade 3 MG: severe or medically significant but not immediately life-threatening; hospitalization or	**For Grade 3:**<br>Hold study drug/study regimen dose until resolution to Grade ≤1.<br>Permanently discontinue study drug/study regimen if Grade 3 imAE does not resolve to Grade ≤1 within 30 days or if there are signs of respiratory insufficiency or autonomic instability.<br>**For Grade 4:**<br>Permanently discontinue study drug/study regimen.	**For Grade 3 or 4 (severe or life-threatening events):**<br>– Consider, as necessary, discussing with study physician.<br>– Recommend hospitalization.<br>– Monitor symptoms and obtain Neurology consult.<br>*MYASTHENIA GRAVIS:*<br>○ Steroids may be successfully used to treat myasthenia gravis. They should typically be administered in a monitored setting under supervision of a consulting neurologist.<br>○ Patients unable to tolerate steroids may be candidates for treatment with plasmapheresis or IV IG.<br>○ If myasthenia gravis-like neurotoxicity present, consider starting AChE inhibitor therapy in addition to steroids. Such therapy, if successful, can also serve to reinforce the diagnosis.<br>*GUILLAIN-BARRE:*

	prolongation of existing hospitalization indicated; limiting self care ADL; Grade 4 MG: life-threatening consequences; urgent intervention indicated)		○ It is important to consider here that the use of steroids as the primary treatment of Guillain-Barre is not typically considered effective.<br>○ Patients requiring treatment should be started with IV IG and followed by plasmapheresis if not responsive to IV IG.
**Myocarditis**	**Any Grade**	**General Guidance**<br>Discontinue drug permanently if biopsy-proven immune-mediated myocarditis.	**For Any Grade:**<br>– The prompt diagnosis of immune-mediated myocarditis is important, particularly in patients with baseline cardiopulmonary disease and reduced cardiac function.<br>– Consider, as necessary, discussing with the study physician.<br>– Monitor patients for signs and symptoms of myocarditis (new onset or worsening chest pain, arrhythmia, shortness of breath, peripheral edema). As some symptoms can overlap with lung toxicities, simultaneously evaluate for and rule out pulmonary toxicity as well as other causes (e.g., pulmonary embolism, congestive heart failure, malignant pericardial effusion). A Cardiology consultation should be obtained early, with prompt assessment of whether and when to complete a cardiac biopsy, including any other diagnostic procedures.<br>– Initial work-up should include clinical evaluation, BNP, cardiac enzymes, ECG, echocardiogram (ECHO), monitoring of oxygenation via pulse oximetry (resting and exertion), and additional laboratory work-up as indicated. Spiral CT or cardiac MRI can complement ECHO to assess wall motion abnormalities when needed.<br>– Patients should be thoroughly evaluated to rule out any alternative etiology (e.g., disease progression, other medications, or infections)
	**Grade 1**<br>(asymptomatic or mild symptoms*; clinical or	No dose modifications required unless clinical suspicion is high, in which case hold study drug/study regimen dose	**For Grade 1 (no definitive findings):**<br>- Monitor and closely follow up in 2 to 4 days for clinical symptoms, BNP, cardiac enzymes, ECG, ECHO, pulse

diagnostic observations only; intervention not indicated)<br><br>*Treat myocarditis with mild symptoms as Grade 2.	during diagnostic work-up for other etiologies. If study drug/study regimen is held, resume after complete resolution to Grade 0.	oximetry (resting and exertion), and laboratory work-up as clinically indicated.<br>- Consider using steroids if clinical suspicion is high.
**Grade 2, 3 or 4**<br>(Grade 2: Symptoms with moderate activity or exertion)<br><br>(Grade 3: Severe with symptoms at rest or with minimal activity or exertion; intervention indicated; new onset of symptoms*)<br><br>(Grade 4: Life-threatening consequences; urgent intervention indicated (e.g., continuous IV therapy or mechanical hemodynamic support))	- If Grade 2 -- Hold study drug/study regimen dose until resolution to Grade 0. If toxicity rapidly improves to Grade 0, then the decision to reinitiate study drug/study regimen will be based upon treating physician's clinical judgment and after completion of steroid taper. If toxicity does not rapidly improve, permanently. discontinue study drug/study regimen.<br>If Grade 3-4, permanently discontinue study drug/study regimen.	**For Grade 2-4:**<br>– Monitor symptoms daily, hospitalize.<br>– Promptly start IV methylprednisolone 2 to 4 mg/kg/day or equivalent after Cardiology consultation has determined whether and when to complete diagnostic procedures including a cardiac biopsy.<br>– Supportive care (e.g., oxygen).<br>– If no improvement within 3 to 5 days despite IV methylprednisolone at 2 to 4 mg/kg/day, promptly start immunosuppressive therapy such as TNF inhibitors (e.g., infliximab at 5 mg/kg every 2 weeks). Caution: It is important to rule out sepsis and refer to infliximab label for general guidance before using infliximab.<br>– Once the patient is improving, gradually taper steroids over ≥28 days and consider prophylactic antibiotics, antifungals, or anti-PJP treatment (refer to current NCCN guidelines for treatment of cancer-related infections).[a]

	* Consider "new onset of symptoms" as referring to patients with prior episode of myocarditis.		
**Myositis/Polymyositis ("Poly/myositis")**	**Any Grade**	**General Guidance**	**For Any Grade:**<br>– Monitor patients for signs and symptoms of poly/myositis. Typically, muscle weakness/pain occurs in proximal muscles including upper arms, thighs, shoulders, hips, neck and back, but rarely affects the extremities including hands and fingers; also difficulty breathing and/or trouble swallowing can occur and progress rapidly. Increased general feelings of tiredness and fatigue may occur, and there can be new-onset falling, difficulty getting up from a fall, and trouble climbing stairs, standing up from a seated position, and/or reaching up.<br>– If poly/myositis is suspected, a Neurology consultation should be obtained early, with prompt guidance on diagnostic procedures. Myocarditis may co-occur with poly/myositis; refer to guidance under Myocarditis. Given breathing complications, refer to guidance under Pneumonitis/ILD. Given possibility of an existent (but previously unknown) autoimmune disorder, consider Rheumatology consultation.<br>– Consider, as necessary, discussing with the study physician.<br>– Initial work-up should include clinical evaluation, creatine kinase, aldolase, LDH, BUN/creatinine, erythrocyte sedimentation rate or C-reactive protein level, urine myoglobin, and additional laboratory work-up as indicated, including a number of possible rheumatological/antibody tests (i.e., consider whether a rheumatologist consultation is indicated and could guide need for rheumatoid factor, antinuclear antibody, anti-smooth muscle, antisynthetase [such as anti-Jo-1], and/or signal-recognition particle antibodies). Confirmatory testing may include electromyography, nerve conduction studies, MRI of the muscles, and/or a muscle biopsy. Consider Barium swallow for evaluation of dysphagia or dysphonia.

		Patients should be thoroughly evaluated to rule out any alternative etiology (e.g., disease progression, other medications, or infections).
**Grade 1**<br>(mild pain)	- No dose modifications.	**For Grade 1:**<br>– Monitor and closely follow up in 2 to 4 days for clinical symptoms and initiate evaluation as clinically indicated.<br>– Consider Neurology consult.<br>– Consider, as necessary, discussing with the study physician.
**Grade 2**<br>(moderate pain associated with weakness; pain limiting instrumental activities of daily living [ADLs])	Hold study drug/study regimen dose until resolution to Grade ≤1.<br>- Permanently discontinue study drug/study regimen if it does not resolve to Grade ≤1 within 30 days or if there are signs of respiratory insufficiency.	**For Grade 2:**<br>– Monitor symptoms daily and consider hospitalization.<br>– Obtain Neurology consult, and initiate evaluation.<br>– Consider, as necessary, discussing with the study physician.<br>– If clinical course is rapidly progressive (particularly if difficulty breathing and/or trouble swallowing), promptly start IV methylprednisolone 2 to 4 mg/kg/day systemic steroids along with receiving input from Neurology consultant<br>– If clinical course is *not* rapidly progressive, start systemic steroids (e.g., prednisone 1 to 2 mg/kg/day PO or IV equivalent); if no improvement within 3 to 5 days, continue additional work up and start treatment with IV methylprednisolone 2 to 4 mg/kg/day<br>– If after start of IV methylprednisolone at 2 to 4 mg/kg/day there is no improvement within 3 to 5 days, consider start of immunosuppressive therapy such as TNF inhibitors (e.g., infliximab at 5 mg/kg every 2 weeks). Caution: It is important to rule out sepsis and refer to infliximab label for general guidance before using infliximab.<br>– Once the patient is improving, gradually taper steroids over ≥28 days and consider prophylactic antibiotics, antifungals, or anti-PJP treatment (refer to current NCCN guidelines for treatment of cancer-related infections).[a]
**Grade 3 or 4**<br>(Grade 3: pain associated with severe weakness; limiting self-care ADLs	**For Grade 3:**<br>Hold study drug/study regimen dose until resolution to Grade ≤1.	**For Grade 3 or 4 (severe or life-threatening events):**<br>– Monitor symptoms closely; recommend hospitalization.<br>– Obtain Neurology consult, and complete full evaluation.<br>– Consider, as necessary, discussing with the study physician.

Grade 4: life-threatening consequences; urgent intervention indicated)	Permanently discontinue study drug/study regimen if Grade 3 imAE does not resolve to Grade ≤1 within 30 days or if there are signs of respiratory insufficiency.<br><br>**For Grade 4:**<br>- Permanently discontinue study drug/study regimen.	– Promptly start IV methylprednisolone 2 to 4 mg/kg/day systemic steroids <u>along with receiving input</u> from Neurology consultant.<br>– If after start of IV methylprednisolone at 2 to 4 mg/kg/day there is no improvement within 3 to 5 days, consider start of immunosuppressive therapy such as TNF inhibitors (e.g., infliximab at 5 mg/kg every 2 weeks). Caution: It is important to rule out sepsis and refer to infliximab label for general guidance before using infliximab.<br>– Consider whether patient may require IV IG, plasmapheresis.<br>– Once the patient is improving, gradually taper steroids over ≥28 days and consider prophylactic antibiotics, antifungals, or anti-PJP treatment (refer to current NCCN guidelines for treatment of cancer-related infections).[a]

[a]ASCO Educational Book 2015 “Managing Immune Checkpoint Blocking Antibody Side Effects” by Michael Postow MD.

[b]FDA Liver Guidance Document 2009 Guidance for Industry: Drug Induced Liver Injury – Premarketing Clinical Evaluation.

AChE Acetylcholine esterase; ADL Activities of daily living; AE Adverse event; ALP Alkaline phosphatase test; ALT Alanine aminotransferase; AST Aspartate aminotransferase; BUN Blood urea nitrogen; CT Computed tomography; CTCAE Common Terminology Criteria for Adverse Events; ILD Interstitial lung disease; imAE immune-mediated adverse event; IG Immunoglobulin; IV Intravenous; GI Gastrointestinal; LFT Liver function tests; LLN Lower limit of normal; MRI Magnetic resonance imaging; NCI National Cancer Institute; NCCN National Comprehensive Cancer Network; PJP *Pneumocystis jirovecii* pneumonia (formerly known as *Pneumocystis carinii* pneumonia); PO By mouth; T3 Triiodothyronine; T4 Thyroxine; TB Total bilirubin; TNF Tumor necrosis factor; TSH Thyroid-stimulating hormone; ULN Upper limit of normal.

Infusion-Related Reactions

Severity Grade of the Event (NCI CTCAE version 5.0)	Dose Modifications	Toxicity Management
**Any Grade**	General Guidance	**For Any Grade:**<br>– Manage per institutional standard at the discretion of investigator.<br>– Monitor patients for signs and symptoms of infusion-related reactions (e.g., fever and/or shaking chills, flushing and/or itching, alterations in heart rate and blood pressure, dyspnea or chest discomfort, or skin rashes) and anaphylaxis (e.g., generalized urticaria, angioedema, wheezing, hypotension, or tachycardia).
**Grade 1 or 2**	**For Grade 1:**<br>The infusion rate of study drug/study regimen may be decreased by 50% or temporarily interrupted until resolution of the event.<br>**For Grade 2:**<br>The infusion rate of study drug/study regimen may be decreased 50% or temporarily interrupted until resolution of the event.<br>Subsequent infusions may be given at 50% of the initial infusion rate.	**For Grade 1 or 2:**<br>– Acetaminophen and/or antihistamines may be administered per institutional standard at the discretion of the investigator.<br>– Consider premedication per institutional standard prior to subsequent doses.<br>– Steroids should not be used for routine premedication of Grade ≤2 infusion reactions.
**Grade 3 or 4**	**For Grade 3 or 4:**<br>Permanently discontinue study drug/study regimen.	**For Grade 3 or 4:**<br>– Manage severe infusion-related reactions per institutional standards (e.g., IM epinephrine, followed by IV diphenhydramine and ranitidine, and IV glucocorticoid).

CTCAE Common Terminology Criteria for Adverse Events; IM intramuscular; IV intravenous; NCI National Cancer Institute.

Non–Immune-Mediated Reactions

Severity Grade of the Event (NCI CTCAE version 5.0)	Dose Modifications	Toxicity Management
**Any Grade**	Note: Dose modifications are not required for AEs not deemed to be related to study treatment (i.e., events due to underlying disease) or for laboratory abnormalities not deemed to be clinically significant.	Treat accordingly, as per institutional standard.
**Grade 1**	No dose modifications.	Treat accordingly, as per institutional standard.
**Grade 2**	Hold study drug/study regimen until resolution to ≤Grade 1 or baseline.	Treat accordingly, as per institutional standard.
**Grade 3**	Hold study drug/study regimen until resolution to ≤Grade 1 or baseline.<br>For AEs that downgrade to ≤Grade 2 within 7 days or resolve to ≤Grade 1 or baseline within 14 days, resume study drug/study regimen administration. Otherwise, discontinue study drug/study regimen.	Treat accordingly, as per institutional standard.
**Grade 4**	Discontinue study drug/study regimen (Note: For Grade 4 labs, decision to discontinue should be based on accompanying clinical signs/symptoms, the Investigator's clinical judgment, and consultation with the Sponsor.).	Treat accordingly, as per institutional standard.

Note: As applicable, for early phase studies, the following sentence may be added: "Any event greater than or equal to Grade 2, please discuss with Study Physician."

AE Adverse event; CTCAE Common Terminology Criteria for Adverse Events; NCI National Cancer Institute.

Appendix 2. Durvalumab DOSE CALCULATIONS

For durvalumab dosing done depending on subject weight:

1. Cohort dose: X mg/kg

2. Subject weight: Y kg

3. Dose for subject: XY mg = X (mg/kg) × Y (kg)

4. Dose to be added into infusion bag:

Dose (mL) = XY mg / 50 (mg/mL)

where 50 mg/mL is durvalumab nominal concentration.

The corresponding volume of durvalumab should be rounded to the nearest tenth mL (0.1 mL). Dose adjustments for each cycle are only needed for greater than 10% change in weight.

5. The theoretical number of vials required for dose preparation is the next greatest whole number of vials from the following formula:

Number of vials = Dose (mL) / 10.0 (mL/vial)

Example:

1. Cohort dose: 10 mg/kg

2. Subject weight: 30 kg

3. Dose for subject: 300 mg = 10 (mg/kg) × 30 (kg)

4. Dose to be added into infusion bag:

Dose (mL) = 300 mg / 50 (mg/mL) = 6.0 mL

5. The theoretical number of vials required for dose preparation:

Number of vials = 6.0 (mL) / 10.0 (mL/vial) = 1 vials

Appendix 3. Durvalumab DOSE VOLUME CALCULATIONS

For durvalumab flat dosing:

1. Cohort dose: X g

2. Dose to be added into infusion bag:

Dose (mL) = X g × 1000 / 50 (mg/mL)

where 50 mg/mL is durvalumab nominal concentration.

The corresponding volume of durvalumab should be rounded to the nearest tenth mL (0.1 mL).

3. The theoretical number of vials required for dose preparation is the next greatest whole number of vials from the following formula:

Number of vials = Dose (mL) / 10.0 (mL/vial)

Example:

1. Cohort dose: 1.5 g

2. Dose to be added into infusion bag:

Dose (mL) = 1.5 g ×1000 / 50 (mg/mL) = 30.0 mL

3. The theoretical number of vials required for dose preparation:

Number of vials = 30.0 (mL) / 10.0 (mL/vial) = 3 vials

Appendix 4. Tremelimumab DOSE CALCULATIONS

For tremelimumab dosing done depending on subject weight:
1. Cohort dose: X mg/kg

2. Subject weight: Y kg

3. Dose for subject: XY mg = X (mg/kg) × Y (kg)

4. Dose to be added into infusion bag:

Dose (mL) = XY mg / 20 (mg/mL)

where 20 mg/mL is tremelimumab nominal concentration.

The corresponding volume of tremelimumab should be rounded to the nearest tenth mL (0.1 mL). Dose adjustments for each cycle are only needed for greater than 10% change in weight.

5. The theoretical number of vials required for dose preparation is the next greatest whole number of vials from the following formula:

Number of vials = Dose (mL) / 20.0 (mL/vial)

Example:

1. Cohort dose: 1 mg/kg

2. Subject weight: 30 kg

3. Dose for subject: 30 mg = 1 (mg/kg) × 30 (kg)

4. Dose to be added into infusion bag:

Dose (mL) = 30 mg / 20 (mg/mL) = 1.5 mL

5. The theoretical number of vials required for dose preparation:

Number of vials = 1.5 (mL) / 20.0 (mL/vial) = 1 vials

Appendix 5. Tremelimumab DOSE VOLUME CALCULATIONS

For tremelimumab flat dosing:

1. Cohort dose: X mg

2. Dose to be added into infusion bag:

Dose (mL) = X mg / 20 (mg/mL)

where 20 mg/mL is tremelimumab nominal concentration

The corresponding volume of tremelimumab should be rounded to the nearest tenth mL (0.1 mL).

3. The theoretical number of vials required for dose preparation is the next greatest whole number of vials from the following formula:

Number of vials = Dose (mL) / 20 (mL/vial)

Example:

1. Cohort dose: 75 mg

2. Dose to be added into infusion bag:

Dose (mL) = 75 mg / 20 (mg/mL) = 3.8 mL

3. The theoretical number of vials required for dose preparation:

Number of vials = 3.8 (mL) / 20 (mL/vial) = 1 vial

Section/Topic	Item No	Checklist item	Reported on page No
Title and abstract			
	1a	Identification as a randomised trial in the title	NA
	1b	Structured summary of trial design, methods, results, and conclusions (for specific guidance see CONSORT for abstracts)	1
Introduction			
Background and objectives	2a	Scientific background and explanation of rationale	4
	2b	Specific objectives or hypotheses	4
Methods			
Trial design	3a	Description of trial design (such as parallel, factorial) including allocation ratio	33
	3b	Important changes to methods after trial commencement (such as eligibility criteria), with reasons	NA
Participants	4a	Eligibility criteria for participants	34-38
	4b	Settings and locations where the data were collected	39
Interventions	5	The interventions for each group with sufficient details to allow replication, including how and when they were actually administered	39
Outcomes	6a	Completely defined pre-specified primary and secondary outcome measures, including how and when they were assessed	38
	6b	Any changes to trial outcomes after the trial commenced, with reasons	NA
Sample size	7a	How sample size was determined	38-39
	7b	When applicable, explanation of any interim analyses and stopping guidelines	NA

Randomisation:			
Sequence generation	8a	Method used to generate the random allocation sequence	NA
	8b	Type of randomisation; details of any restriction (such as blocking and block size)	NA
Allocation concealment mechanism	9	Mechanism used to implement the random allocation sequence (such as sequentially numbered containers), describing any steps taken to conceal the sequence until interventions were assigned	NA
Implementation	10	Who generated the random allocation sequence, who enrolled participants, and who assigned participants to interventions	NA
Blinding	11a	If done, who was blinded after assignment to interventions (for example, participants, care providers, those assessing outcomes) and how	NA
	11b	If relevant, description of the similarity of interventions	NA
Statistical methods	12a	Statistical methods used to compare groups for primary and secondary outcomes	NA
	12b	Methods for additional analyses, such as subgroup analyses and adjusted analyses	49-51 to 55
Results			
Participant flow (a diagram is strongly recommended)	13a	For each group, the numbers of participants who were randomly assigned, received intended treatment, and were analysed for the primary outcome	Extended data figure 1b
	13b	For each group, losses and exclusions after randomisation, together with reasons	Extended data figure 1b
Recruitment	14a	Dates defining the periods of recruitment and follow-up	5
	14b	Why the trial ended or was stopped	6

Baseline data	15	A table showing baseline demographic and clinical characteristics for each group	Extended Data Table 1
Numbers analysed	16	For each group, number of participants (denominator) included in each analysis and whether the analysis was by original assigned groups	Extended data figure 1b
Outcomes and estimation	17a	For each primary and secondary outcome, results for each group, and the estimated effect size and its precision (such as 95% confidence interval)	6-7
	17b	For binary outcomes, presentation of both absolute and relative effect sizes is recommended	NA
Ancillary analyses	18	Results of any other analyses performed, including subgroup analyses and adjusted analyses, distinguishing pre-specified from exploratory	7 to 13
Harms	19	All important harms or unintended effects in each group (for specific guidance see CONSORT for harms)	NA
Discussion			
Limitations	20	Trial limitations, addressing sources of potential bias, imprecision, and, if relevant, multiplicity of analyses	17
Generalisability	21	Generalisability (external validity, applicability) of the trial findings	NA
Interpretation	22	Interpretation consistent with results, balancing benefits and harms, and considering other relevant evidence	15 to 17
Other information			
Registration	23	Registration number and name of trial registry	3 and 33
Protocol	24	Where the full trial protocol can be accessed, if available	Supplementary information
Funding	25	Sources of funding and other support (such as supply of drugs), role of funders	18
