# Supplementary material for: First-line durvalumab and tremelimumab with chemotherapy in RAS-mutated metastatic colorectal cancer: a phase 1b/2 trial
Source: Nat Med. 2023 Aug 10;29(8):2087–98. doi: 10.1038/s41591-023-02497-z (PMC10427431; doi:10.1038/s41591-023-02497-z)
Supplement: Supplementary file 2 — Reporting Summary [file 41591_2023_2497_MOESM2_ESM.pdf]

nature portfolio

Corresponding author(s): François Ghiringhelli

Last updated by author(s): 12/12/2022

Reporting Summary

Nature Portfolio wishes to improve the reproducibility of the work that we publish. This form provides structure for consistency and transparency in reporting. For further information on Nature Portfolio policies, see our Editorial Policies and the Editorial Policy Checklist.

Statistics

For all statistical analyses, confirm that the following items are present in the figure legend, table legend, main text, or Methods section.

n/a	Confirmed	
☐	☒	The exact sample size (*n*) for each experimental group/condition, given as a discrete number and unit of measurement
☐	☒	A statement on whether measurements were taken from distinct samples or whether the same sample was measured repeatedly
☐	☒	The statistical test(s) used AND whether they are one- or two-sided<br>*Only common tests should be described solely by name; describe more complex techniques in the Methods section.*
☐	☒	A description of all covariates tested
☐	☒	A description of any assumptions or corrections, such as tests of normality and adjustment for multiple comparisons
☐	☐	A full description of the statistical parameters including central tendency (e.g. means) or other basic estimates (e.g. regression coefficient) AND variation (e.g. standard deviation) or associated estimates of uncertainty (e.g. confidence intervals)
☐	☒	For null hypothesis testing, the test statistic (e.g. *F*, *t*, *r*) with confidence intervals, effect sizes, degrees of freedom and *P* value noted<br>*Give P values as exact values whenever suitable.*
☒	☐	For Bayesian analysis, information on the choice of priors and Markov chain Monte Carlo settings
☒	☐	For hierarchical and complex designs, identification of the appropriate level for tests and full reporting of outcomes
☒	☐	Estimates of effect sizes (e.g. Cohen's *d*, Pearson's *r*), indicating how they were calculated

Our web collection on statistics for biologists contains articles on many of the points above.

Software and code

Policy information about availability of computer code

Data collection	no software
Data analysis	Statistical analyses were performed using the R software version 4.0.3 (http://www.R-project.org/) and graphs were drawn using GraphPad Prism V.9.0.2.

For manuscripts utilizing custom algorithms or software that are central to the research but not yet described in published literature, software must be made available to editors and reviewers. We strongly encourage code deposition in a community repository (e.g. GitHub). See the Nature Portfolio guidelines for submitting code & software for further information.

Data

Policy information about availability of data

All manuscripts must include a data availability statement. This statement should provide the following information, where applicable:

- Accession codes, unique identifiers, or web links for publicly available datasets
- A description of any restrictions on data availability
- For clinical datasets or third party data, please ensure that the statement adheres to our policy

Clinical data are available in the Extended Data Table 1. RNAseq and scRNAseq data were uploaded on GEO.

Human research participants

Policy information about studies involving human research participants and Sex and Gender in Research.

Reporting on sex and gender	Sex and gender are described in Table 1. We did not take sex into account in this study because this is not a relevant issue in this disease.
Population characteristics	These data are presented in Extended Data Table 1
Recruitment	The patients were recruited by 9 cancer research centers in France
Ethics oversight	The protocol was approved by the french reglementary autority (CPP) as appropiated and mansion in the protocol

Note that full information on the approval of the study protocol must also be provided in the manuscript.

Field-specific reporting

Please select the one below that is the best fit for your research. If you are not sure, read the appropriate sections before making your selection.

☒ Life sciences ☐ Behavioural & social sciences ☐ Ecological, evolutionary & environmental sciences

For a reference copy of the document with all sections, see nature.com/documents/nr-reporting-summary-flat.pdf

Life sciences study design

All studies must disclose on these points even when the disclosure is negative.

Sample size	57
Data exclusions	Exclusion criteria are mentioned in the methods and in the full protocol
Replication	No replication in a clinical trial
Randomization	no
Blinding	no

Behavioural & social sciences study design

All studies must disclose on these points even when the disclosure is negative.

Study description	*Briefly describe the study type including whether data are quantitative, qualitative, or mixed-methods (e.g. qualitative cross-sectional, quantitative experimental, mixed-methods case study).*
Research sample	*State the research sample (e.g. Harvard university undergraduates, villagers in rural India) and provide relevant demographic information (e.g. age, sex) and indicate whether the sample is representative. Provide a rationale for the study sample chosen. For studies involving existing datasets, please describe the dataset and source.*
Sampling strategy	*Describe the sampling procedure (e.g. random, snowball, stratified, convenience). Describe the statistical methods that were used to predetermine sample size OR if no sample-size calculation was performed, describe how sample sizes were chosen and provide a rationale for why these sample sizes are sufficient. For qualitative data, please indicate whether data saturation was considered, and what criteria were used to decide that no further sampling was needed.*
Data collection	*Provide details about the data collection procedure, including the instruments or devices used to record the data (e.g. pen and paper, computer, eye tracker, video or audio equipment) whether anyone was present besides the participant(s) and the researcher, and whether the researcher was blind to experimental condition and/or the study hypothesis during data collection.*
Timing	*Indicate the start and stop dates of data collection. If there is a gap between collection periods, state the dates for each sample cohort.*
Data exclusions	*If no data were excluded from the analyses, state so OR if data were excluded, provide the exact number of exclusions and the rationale behind them, indicating whether exclusion criteria were pre-established.*

Non-participation	*State how many participants dropped out/declined participation and the reason(s) given OR provide response rate OR state that no participants dropped out/declined participation.*
Randomization	*If participants were not allocated into experimental groups, state so OR describe how participants were allocated to groups, and if allocation was not random, describe how covariates were controlled.*

Ecological, evolutionary & environmental sciences study design

All studies must disclose on these points even when the disclosure is negative.

Study description	*Briefly describe the study. For quantitative data include treatment factors and interactions, design structure (e.g. factorial, nested, hierarchical), nature and number of experimental units and replicates.*
Research sample	*Describe the research sample (e.g. a group of tagged Passer domesticus, all Stenocereus thurberi within Organ Pipe Cactus National Monument), and provide a rationale for the sample choice. When relevant, describe the organism taxa, source, sex, age range and any manipulations. State what population the sample is meant to represent when applicable. For studies involving existing datasets, describe the data and its source.*
Sampling strategy	*Note the sampling procedure. Describe the statistical methods that were used to predetermine sample size OR if no sample-size calculation was performed, describe how sample sizes were chosen and provide a rationale for why these sample sizes are sufficient.*
Data collection	*Describe the data collection procedure, including who recorded the data and how.*
Timing and spatial scale	*Indicate the start and stop dates of data collection, noting the frequency and periodicity of sampling and providing a rationale for these choices. If there is a gap between collection periods, state the dates for each sample cohort. Specify the spatial scale from which the data are taken*
Data exclusions	*If no data were excluded from the analyses, state so OR if data were excluded, describe the exclusions and the rationale behind them, indicating whether exclusion criteria were pre-established.*
Reproducibility	*Describe the measures taken to verify the reproducibility of experimental findings. For each experiment, note whether any attempts to repeat the experiment failed OR state that all attempts to repeat the experiment were successful.*
Randomization	*Describe how samples/organisms/participants were allocated into groups. If allocation was not random, describe how covariates were controlled. If this is not relevant to your study, explain why.*
Blinding	*Describe the extent of blinding used during data acquisition and analysis. If blinding was not possible, describe why OR explain why blinding was not relevant to your study.*

Did the study involve field work? ☐ Yes ☐ No

Field work, collection and transport

Field conditions	*Describe the study conditions for field work, providing relevant parameters (e.g. temperature, rainfall).*
Location	*State the location of the sampling or experiment, providing relevant parameters (e.g. latitude and longitude, elevation, water depth).*
Access & import/export	*Describe the efforts you have made to access habitats and to collect and import/export your samples in a responsible manner and in compliance with local, national and international laws, noting any permits that were obtained (give the name of the issuing authority, the date of issue, and any identifying information).*
Disturbance	*Describe any disturbance caused by the study and how it was minimized.*

Reporting for specific materials, systems and methods

We require information from authors about some types of materials, experimental systems and methods used in many studies. Here, indicate whether each material, system or method listed is relevant to your study. If you are not sure if a list item applies to your research, read the appropriate section before selecting a response.

Materials & experimental systems

n/a	Involved in the study
☐	☒ Antibodies
☒	☐ Eukaryotic cell lines
☒	☐ Palaeontology and archaeology
☒	☐ Animals and other organisms
☐	☒ Clinical data
☒	☐ Dual use research of concern

Methods

n/a	Involved in the study
☒	☐ ChIP-seq
☐	☒ Flow cytometry
☒	☐ MRI-based neuroimaging

Antibodies

Antibodies used

anti-CD183-FITC (clone G025H7), (Beckman Coulter)
anti-CD197-PE (clone G043H7), (Beckman Coulter)
anti-CD196-PE-Cy7 (clone B-R35), (Beckman Coulter)
anti-CD278-APC (clone ISA-3), (Beckman Coulter)
anti-CD45RA-AlexaFluor700 (clone 2H4LDH11LDB9 (2H4)), (Beckman Coulter)
anti-HLA-DR-APC-AlexaFluor750 (clone Immu-357), (Beckman Coulter)
anti-CD4-PacBlue (clone 13B8.2) (Beckman Coulter)
anti-CD8-KromeOrange (clone B9.11) (Beckman Coulter)
anti-CCR4-PerCP-Cy5.5 (BioLegend, clone L291H4)
anti-CD28-BV605 (BD Biosciences, clone CD28.2).
anti-PD1-APC (clone PD1.3), (Beckman Coulter)
anti-HLA-DR-KromeOrange (clone Immu-357) (Beckman Coulter)
anti-CD80-APC-AlexaFluor750 (BD Biosciences, clone L307.4)
anti-CD127-BV605 (BioLegend, clone A019D5).
anti-CD25-PE (clone B1.49.9), (Beckman Coulter)
anti-CD39-PE-Cy5 (clone BA54), (Beckman Coulter)
anti-PD1-PE-Cy7 (clone PD1.3), (Beckman Coulter)
anti-CCR4-PerCP-Cy5.5 (BioLegend, clone L291H4),
anti-Tim3-BV605 (BioLegend, clone F38-282).
anti-CD159a-PE (clone Z199), (Beckman Coulter)
anti-PD1-PE-Cy5 (clone PD1.3) (Beckman Coulter)
anti-CD335-PE-Cy7 (clone BAB281), (Beckman Coulter)
anti-CD314-APC (clone ON72), (Beckman Coulter)
anti-CD56-APC-AlexaFluor750 (clone N901), (Beckman Coulter)
anti-CD16-PacBlue (clone 3G8) (Beckman Coulter)
anti-Tim3-FITC (Miltenyi Biotec, clone REA635),
anti-NKG2C-AlexaFluor700 (R&D Systems, clone 134591)
anti-CD3-BV605 (BioLegend, clone UCHT1).
anti-CD33-FITC (clone D3HL60.251), (Beckman Coulter)
anti-CD39-PE (clone BA54), (Beckman Coulter)
anti-CD3-Pe-Cy5 (clone UCTH1), (Beckman Coulter)
anti-CD19-PE-Cy5 (clone J3-119), (Beckman Coulter)
anti-CD20-PE-Cy5 (clone B9E9), (Beckman Coulter)
anti-CD56-PE-Cy5 (clone N901), (Beckman Coulter)
anti-PD-L1-APC (clone PDL1.3.1), (Beckman Coulter)
anti-HLA-DR-APC-AlexaFluor750 (clone Immu-357), (Beckman Coulter)
anti-CD15-PacBlue (clone 80H5), (Beckman Coulter)
anti-CD14-KromeOrange (clone RMO52) (Beckman Coulter)
anti-CD11b-BV605 (BioLegend, clone ICRF44).
anti-TCRαβ-PE Clone IP26A, Beckman Coulter
anti-TCRγδ (Clone IMMU510, Beckman Coulter)
anti-human CD8 (1/100, clone C8/144B, M7103, Agilent)
anti-human PD-L1 (1/200, clone QR1, C-P0001-01, Diagomics)

Validation

Dose of mAb were described in the material and methods and used at dose recommanded by the provider.

Eukaryotic cell lines

Policy information about cell lines and Sex and Gender in Research

Cell line source(s)

State the source of each cell line used and the sex of all primary cell lines and cells derived from human participants or vertebrate models.

Authentication

Describe the authentication procedures for each cell line used OR declare that none of the cell lines used were authenticated.

Mycoplasma contamination

Confirm that all cell lines tested negative for mycoplasma contamination OR describe the results of the testing for mycoplasma contamination OR declare that the cell lines were not tested for mycoplasma contamination.

Commonly misidentified lines (See ICLAC register) | *Name any commonly misidentified cell lines used in the study and provide a rationale for their use.*

Palaeontology and Archaeology

Specimen provenance | *Provide provenance information for specimens and describe permits that were obtained for the work (including the name of the issuing authority, the date of issue, and any identifying information). Permits should encompass collection and, where applicable, export.*

Specimen deposition | *Indicate where the specimens have been deposited to permit free access by other researchers.*

Dating methods | *If new dates are provided, describe how they were obtained (e.g. collection, storage, sample pretreatment and measurement), where they were obtained (i.e. lab name), the calibration program and the protocol for quality assurance OR state that no new dates are provided.*

☐ Tick this box to confirm that the raw and calibrated dates are available in the paper or in Supplementary Information.

Ethics oversight | *Identify the organization(s) that approved or provided guidance on the study protocol, OR state that no ethical approval or guidance was required and explain why not.*

Note that full information on the approval of the study protocol must also be provided in the manuscript.

Animals and other research organisms

Policy information about studies involving animals; ARRIVE guidelines recommended for reporting animal research, and Sex and Gender in Research

Laboratory animals | *For laboratory animals, report species, strain and age OR state that the study did not involve laboratory animals.*

Wild animals | *Provide details on animals observed in or captured in the field; report species and age where possible. Describe how animals were caught and transported and what happened to captive animals after the study (if killed, explain why and describe method; if released, say where and when) OR state that the study did not involve wild animals.*

Reporting on sex | *Indicate if findings apply to only one sex; describe whether sex was considered in study design, methods used for assigning sex. Provide data disaggregated for sex where this information has been collected in the source data as appropriate; provide overall numbers in this Reporting Summary. Please state if this information has not been collected. Report sex-based analyses where performed, justify reasons for lack of sex-based analysis.*

Field-collected samples | *For laboratory work with field-collected samples, describe all relevant parameters such as housing, maintenance, temperature, photoperiod and end-of-experiment protocol OR state that the study did not involve samples collected from the field.*

Ethics oversight | *Identify the organization(s) that approved or provided guidance on the study protocol, OR state that no ethical approval or guidance was required and explain why not.*

Note that full information on the approval of the study protocol must also be provided in the manuscript.

Clinical data

Policy information about clinical studies
All manuscripts should comply with the ICMJE guidelines for publication of clinical research and a completed CONSORT checklist must be included with all submissions.

Clinical trial registration | NCT03202758

Study protocol | The study protocol was added as supplementary data

Data collection | 57 patients with unresectable metastatic RAS-mutated CRC were included from 9 hospitals in France between August 2017 and December 2019. Data base was locked down on 21 december 2021.

Outcomes |
Phase Ib Objective:
To determine the safety of the combination of Durvalumab (Anti-PDL-1) + Tremelimumab (Anti-CTLA-4) + FOLFOX

Phase II Objectives:
To determine efficacy of the combination of Durvalumab (Anti-PD-L1) + Tremelimumab (Anti-CTLA-4) + FOLFOX in terms of PFS in patients with colorectal MSS disease.

Secondary objective
Phase II Secondary Objective:
To determine efficacy of the combination of Durvalumab (Anti-PDL1) + Tremelimumab (Anti-CTLA-4) + FOLFOX in terms of response to treatment and overall survival in patients with colorectal MSS disease.
- To determine efficacy of the combination of Durvalumab (Anti-PD-L1) + Tremelimumab (Anti-CTLA-4) + FOLFOX in terms of PFS,

response to treatment and overall survival in patients with colorectal MSI disease.

Exploratory Studies :

 To evaluate quality of life at each cycle,
 To determine genetically characterized for MSI status,
 To determine NRAS, KRAS and Braf status,
 To study the immune cells infiltration into the tumor,
 To analyze PD-1, PD-L1, CTLA-4 expression with Ventana assay system
 To determine double labelling of Th1, Th2, Th17, Follicular helper T cells and exhausted T cells,
 To perform identification of tumor-specific mutations,
 To determine candidate of neoantigens and also prediction for proteasomal processing and HLA class I binding will be assess,
 Analyze immune response before and after treatment start,
 To assess local immune response before and after therapy,
 To study evaluation of lymphocyte reactivity to tumor antigens,
 To analyze cytokine production by T cells.

Dual use research of concern

Policy information about dual use research of concern

Hazards

Could the accidental, deliberate or reckless misuse of agents or technologies generated in the work, or the application of information presented in the manuscript, pose a threat to:

No	Yes	
☐	☐	Public health
☐	☐	National security
☐	☐	Crops and/or livestock
☐	☐	Ecosystems
☐	☐	Any other significant area

Experiments of concern

Does the work involve any of these experiments of concern:

No	Yes	
☐	☐	Demonstrate how to render a vaccine ineffective
☐	☐	Confer resistance to therapeutically useful antibiotics or antiviral agents
☐	☐	Enhance the virulence of a pathogen or render a nonpathogen virulent
☐	☐	Increase transmissibility of a pathogen
☐	☐	Alter the host range of a pathogen
☐	☐	Enable evasion of diagnostic/detection modalities
☐	☐	Enable the weaponization of a biological agent or toxin
☐	☐	Any other potentially harmful combination of experiments and agents

ChIP-seq

Data deposition

☐ Confirm that both raw and final processed data have been deposited in a public database such as GEO.

☐ Confirm that you have deposited or provided access to graph files (e.g. BED files) for the called peaks.

Data access links
May remain private before publication.
For "Initial submission" or "Revised version" documents, provide reviewer access links. For your "Final submission" document, provide a link to the deposited data.

Files in database submission
Provide a list of all files available in the database submission.

Genome browser session
(e.g. UCSC)
Provide a link to an anonymized genome browser session for "Initial submission" and "Revised version" documents only, to enable peer review. Write "no longer applicable" for "Final submission" documents.

Methodology

Replicates
Describe the experimental replicates, specifying number, type and replicate agreement.

Sequencing depth | *Describe the sequencing depth for each experiment, providing the total number of reads, uniquely mapped reads, length of reads and whether they were paired- or single-end.*

Antibodies | *Describe the antibodies used for the ChIP-seq experiments; as applicable, provide supplier name, catalog number, clone name, and lot number.*

Peak calling parameters | *Specify the command line program and parameters used for read mapping and peak calling, including the ChIP, control and index files used.*

Data quality | *Describe the methods used to ensure data quality in full detail, including how many peaks are at FDR 5% and above 5-fold enrichment.*

Software | *Describe the software used to collect and analyze the ChIP-seq data. For custom code that has been deposited into a community repository, provide accession details.*

Flow Cytometry

Plots

Confirm that:

☒ The axis labels state the marker and fluorochrome used (e.g. CD4-FITC).

☒ The axis scales are clearly visible. Include numbers along axes only for bottom left plot of group (a 'group' is an analysis of identical markers).

☒ All plots are contour plots with outliers or pseudocolor plots.

☒ A numerical value for number of cells or percentage (with statistics) is provided.

Methodology

Sample preparation

Blood sampling for cytometry analysis occurred during screening, at cycle 1 days 1 and 15, at cycle 3 day 1 and at cycle 6 day 15 and at treatment discontinuation. At each time point, 7 panels of 10 markers were assessed.
*For blood count analysis:
- Antibodies for blood count analysis: Multicolour flow cytometry was performed using Beckman Coulter's custom design service and its dry coating technology, custom tubes containing anti-CD16-FITC (clone 3G8), anti-CD56-PE (clone N901), anti-CD19-PE-Cy5.5 (clone J3-119), anti-CD14-PE-Cy7 (clone RMO52), anti-CD4-APC (clone 13B8.2), anti-CD8-AlexaFluor700 (Clone B9.11), anti-CD3-APC-AlexaFluor750 (clone UCTH1), anti-CD15-PacificBlue (clone 80H5) and anti-CD45-KromeOrange (clone J.33) were produced.
- Staining protocol: 100 µL of total heparinized blood was added to DURAClone tube, vortexed immediately for 15s and incubated for 15 min at room temperature in the dark. Two millilitres of red blood lysis solution (VersaLyse solution, A09777, Beckman Coulter) containing 50 µL of the fixative agent IOTest 3 Fixative solution (A07800, Beckman Coulter) was added, inverted and incubated for 15 min in the dark. Then, 100µL of counting beads (Flow-Count Fluorospheres, 7547053, Beckman Coulter) were added before acquisition a Canto II cytometer (BD Biosciences).
*For immune cell populations identification : To decipher the peripheral immune system, we performed 5 panels to identify and characterize the different lymphocyte and myeloid subpopulations.
- Antibodies for T cell analysis (first panel): Using Beckman Coulter's custom design service and its dry coating technology, custom tubes containing anti-CD183-FITC (clone G025H7), anti-CD197-PE (clone G043H7), anti-CD196-PE-Cy7 (clone B-R35), anti-CD278-APC (clone ISA-3), anti-CD45RA-AlexaFluor700 (clone 2H4LDH11LDB9 (2H4)), anti-HLA-DR-APC-AlexaFluor750 (clone Immu-357), anti-CD4-PacBlue (clone 13B8.2) and anti-CD8-KromeOrange (clone B9.11) were produced. Liquid antibodies were also used: anti-CCR4-PerCP-Cy5.5 (BioLegend, clone L291H4) and anti-CD28-BV605 (BD Biosciences, clone CD28.2).
- Antibodies for T cell analysis (second panel): Using Beckman Coulter's custom design service and its dry coating technology, custom tubes containing anti-CD183-FITC (clone G025H7), anti-CD197-PE (clone G043H7), anti-CD196-PE-Cy7 (clone B-R35), anti-PD1-APC (clone PD1.3), anti-CD45RA-AlexaFluor700 (clone 2H4LDH11LDB9 (2H4)), anti-CD4-PacBlue (clone 13B8.2) and anti-HLA-DR-KromeOrange (clone Immu-357) were produced. Liquid antibodies were also used: anti-CD80-APC-AlexaFluor750 (BD Biosciences, clone L307.4) and anti-CD127-BV605 (BioLegend, clone A019D5).
- Antibodies for Treg cell analysis: Using Beckman Coulter's custom design service and its dry coating technology, custom tubes containing anti-CD25-PE (clone B1.49.9), anti-CD39-PE-Cy5 (clone BA54), anti-PD1-PE-Cy7 (clone PD1.3), anti-CD278-APC (clone ISA-3), anti-CD45RA-AlexaFluor700 (clone 2H4LDH11LDB9 (2H4)), anti-CD4-PacBlue (clone 13B8.2) and anti-CD8-KromeOrange (clone B9.11) were produced. Liquid antibodies were also used: anti-CCR4-PerCP-Cy5.5 (BioLegend, clone L291H4), anti-CD80-APC-AlexaFluor750 (BD Biosciences, clone L307.4) and anti-Tim3-BV605 (BioLegend, clone F38-282).
- Antibodies for NK cell analysis: Using Beckman Coulter's custom design service and its dry coating technology, custom tubes containing anti-CD159a-PE (clone Z199), anti-PD1-PE-Cy5 (clone PD1.3) anti-CD335-PE-Cy7 (clone BAB281), anti-CD314-APC (clone ON72), anti-CD56-APC-AlexaFluor750 (clone N901), anti-CD16-PacBlue (clone 3G8) and anti-CD45- KromeOrange (clone J33) were produced. Liquid antibodies were also used: anti-Tim3-FITC (Miltenyi Biotec, clone REA635), anti-NKG2C-AlexaFluor700 (R&D Systems, clone 134591) and anti-CD3-BV605 (BioLegend, clone UCHT1).
- Antibodies for myeloid cell analysis: Multicolour flow cytometry was also performed using Beckman Coulter's custom design service and its dry coating technology, custom tubes containing anti-CD33-FITC (clone D3HL60.251), anti-CD39-PE (clone BA54), anti-CD3-Pe-Cy5 (clone UCTH1), anti-CD19-PE-Cy5 (clone J3-119), anti-CD20-PE-Cy5 (clone B9E9), anti-CD56-PE-Cy5 (clone N901), anti-PD-L1-APC (clone PDL1.3.1), anti-HLA-DR-APC-AlexaFluor750 (clone Immu-357), anti-CD15-PacBlue (clone 80H5), anti-CD14-KromeOrange (clone RMO52) and a mortality marker DRAQ7 were produced. The following liquid antibody was added to the custom tubes: anti-CD11b-BV605 (BioLegend, clone ICRF44).
- Staining protocol: 100 µL of total heparinized blood was added to each DURAClone tube containing liquid antibodies, vortexed immediately for 15s and incubated for 15 min at room temperature in the dark. Two millilitres of red blood lysis

solution (VersaLyse solution, A09777, Beckman Coulter) containing 50 µL of the fixative agent IOTest 3 Fixative solution (A07800, Beckman Coulter) was added, inverted and incubated for 15 min in the dark. After centrifugation and washing with 3 mL of PBS 1X, cells were resuspended in 150 µL PBS 1X before acquisition on a Canto II cytometer (BD Biosciences).
*For lymphocyte function analysis:
- Antibodies used: Using Beckman Coulter's custom design service and its dry coating technology, custom tubes containing anti-IFNγ-FITC (clone 45.15), anti-CD25-PE (clone B1.49.9), anti-CD4-PE-Cy5.5 (clone 13B8.2), anti-IL-4-PE-Cy7 (clone MP4-25D2), anti-Foxp3-AlexaFluor647 (Clone 259D), anti-TNFα-AlexaFluor700 (Clone IPM2), anti-CD3-APC-AlexaFluor750 (clone UCHT1), anti-IL-17A-PacBlue (clone BL168) and anti-CD8-KromeOrange (clone B9.11) were produced. Liquid antibody was also used: anti-IL-2-BV605 (BioLegend, clone MQ1-17H12).
- Staining procedure: 100 µL of total heparinized blood was added to a DURactive 1 tube containing Phorbol-Myristate-acetate, Ionomycin and Brefledin A (C11101, Beckman Coulter) for 3 hours at 37°C in the dark. After activation, 25 µL of PerFix-NC R1 buffer (PerFix-NC kit, B31168, Beckman Coulter) was added on vortex and incubated for 15 min at room temperature. Then, 2 mL of PBS 1X was added, and after centrifugation the pellet was resuspended in 25 µL of FBS (Dutscher) and 300 µL of PerFix-NC R2 buffer was added. A 325 µL aliquot was transferred to a DURAClone tube containing the liquid antibody, vortexed immediately for 15s and incubated for 1h at room temperature in the dark. PBS 1X (3 mL) was added to the tubes, incubated for 5 min at room temperature in the dark before centrifugation for 5 min at 500g. After supernatant removal, the cells were resuspended in 3 mL of 1X PerFix-NC R3 buffer before another 5 min centrifugation at 500g. The pellet was dried and resuspended in 150 µL of 1X R3 buffer.

Instrument

Data collection was done on a BD flow cytometer, the model is a FACSCanto IVD 10 (3 lasers, 10 parameters).

Software

The data acquisition was done on the Diva software (BD) and the verification and validation of the compensations was done on the Kaluza analysis software (Beckman Coulter).

Cell population abundance

NA

Gating strategy

The gating strategy used to validate compensations of whole blood analyses is as follows: leucocytes were determined by SSC-A vs FSC-A and doublet exclusion performed using FSC-A vs FSC-H and SSC-A vs SSC-H. Then, we validated compensations of each fcs file and we exported a new fcs file for a cleaned and compensated leukocyte population. An unsupervised analysis with R software was then performed by a bioinformatician .
If necessary, we can provide you with a gating strategy for each panel.

☐ Tick this box to confirm that a figure exemplifying the gating strategy is provided in the Supplementary Information.

Magnetic resonance imaging

Experimental design

Design type

Indicate task or resting state; event-related or block design.

Design specifications

Specify the number of blocks, trials or experimental units per session and/or subject, and specify the length of each trial or block (if trials are blocked) and interval between trials.

Behavioral performance measures

State number and/or type of variables recorded (e.g. correct button press, response time) and what statistics were used to establish that the subjects were performing the task as expected (e.g. mean, range, and/or standard deviation across subjects).

Acquisition

Imaging type(s)

Specify: functional, structural, diffusion, perfusion.

Field strength

Specify in Tesla

Sequence & imaging parameters

Specify the pulse sequence type (gradient echo, spin echo, etc.), imaging type (EPI, spiral, etc.), field of view, matrix size, slice thickness, orientation and TE/TR/flip angle.

Area of acquisition

State whether a whole brain scan was used OR define the area of acquisition, describing how the region was determined.

Diffusion MRI ☐ Used ☐ Not used

Preprocessing

Preprocessing software

Provide detail on software version and revision number and on specific parameters (model/functions, brain extraction, segmentation, smoothing kernel size, etc.).

Normalization

If data were normalized/standardized, describe the approach(es): specify linear or non-linear and define image types used for transformation OR indicate that data were not normalized and explain rationale for lack of normalization.

Normalization template

Describe the template used for normalization/transformation, specifying subject space or group standardized space (e.g. original Talairach, MNI305, ICBM152) OR indicate that the data were not normalized.

Noise and artifact removal

Describe your procedure(s) for artifact and structured noise removal, specifying motion parameters, tissue signals and

Noise and artifact removal | *physiological signals (heart rate, respiration).*

Volume censoring | *Define your software and/or method and criteria for volume censoring, and state the extent of such censoring.*

Statistical modeling & inference

Model type and settings | *Specify type (mass univariate, multivariate, RSA, predictive, etc.) and describe essential details of the model at the first and second levels (e.g. fixed, random or mixed effects; drift or auto-correlation).*

Effect(s) tested | *Define precise effect in terms of the task or stimulus conditions instead of psychological concepts and indicate whether ANOVA or factorial designs were used.*

Specify type of analysis: ☐ Whole brain ☐ ROI-based ☐ Both

Statistic type for inference
(See Eklund et al. 2016) | *Specify voxel-wise or cluster-wise and report all relevant parameters for cluster-wise methods.*

Correction | *Describe the type of correction and how it is obtained for multiple comparisons (e.g. FWE, FDR, permutation or Monte Carlo).*

Models & analysis

n/a	Involved in the study
☐	☐ Functional and/or effective connectivity
☐	☐ Graph analysis
☐	☐ Multivariate modeling or predictive analysis

Functional and/or effective connectivity | *Report the measures of dependence used and the model details (e.g. Pearson correlation, partial correlation, mutual information).*

Graph analysis | *Report the dependent variable and connectivity measure, specifying weighted graph or binarized graph, subject- or group-level, and the global and/or node summaries used (e.g. clustering coefficient, efficiency, etc.).*

Multivariate modeling and predictive analysis | *Specify independent variables, features extraction and dimension reduction, model, training and evaluation metrics.*
